# ﻿Review and guide to the isopods (Crustacea, Isopoda) of littoral and sublittoral marine habitats in the Southern California Bight

**DOI:** 10.3897/zookeys.1162.100390

**Published:** 2023-05-16

**Authors:** Timothy D. Stebbins, Regina Wetzer

**Affiliations:** 1 Research and Collections Branch, Natural History Museum of Los Angeles County, 900 Exposition Boulevard, Los Angeles, California 90007, USA Natural History Museum of Los Angeles County Los Angeles United States of America; 2 City of San Diego Marine Biology Laboratory (retired), Public Utilities Department, San Diego, California 92101, USA City of San Diego Marine Biology Laboratory San Diego United States of America

**Keywords:** Baja California, intertidal, isopod crustaceans, keys to species, northeastern Pacific, southern California, subtidal

## Abstract

The isopod crustaceans reported from or expected to occur in littoral and sublittoral marine habitats of the Southern California Bight (SCB) in the northeastern Pacific Ocean are reviewed. A total of 190 species, representing 105 genera in 42 families and six suborders are covered. Approximately 84% of these isopods represent described species with the remaining 16% comprising well-documented “provisional” but undescribed species. Cymothoida and Asellota are the most diverse of the six suborders, accounting for ca. 36% and 29% of the species, respectively. Valvifera and Sphaeromatidea are the next most speciose suborders with between 13–15% of the species each, while the suborder Limnorioidea represents fewer than 2% of the SCB isopod fauna. Finally, the mostly terrestrial suborder Oniscidea accounts for ca. 5% of the species treated herein, each which occurs at or above the high tide mark in intertidal habitats. A key to the suborders and superfamilies is presented followed by nine keys to the SCB species within each of the resultant groups. Figures are provided for most species. Bathymetric range, geographic distribution, type locality, habitat, body size, and a comprehensive list of references are included for most species.

## ﻿Introduction

**Figure 1.**
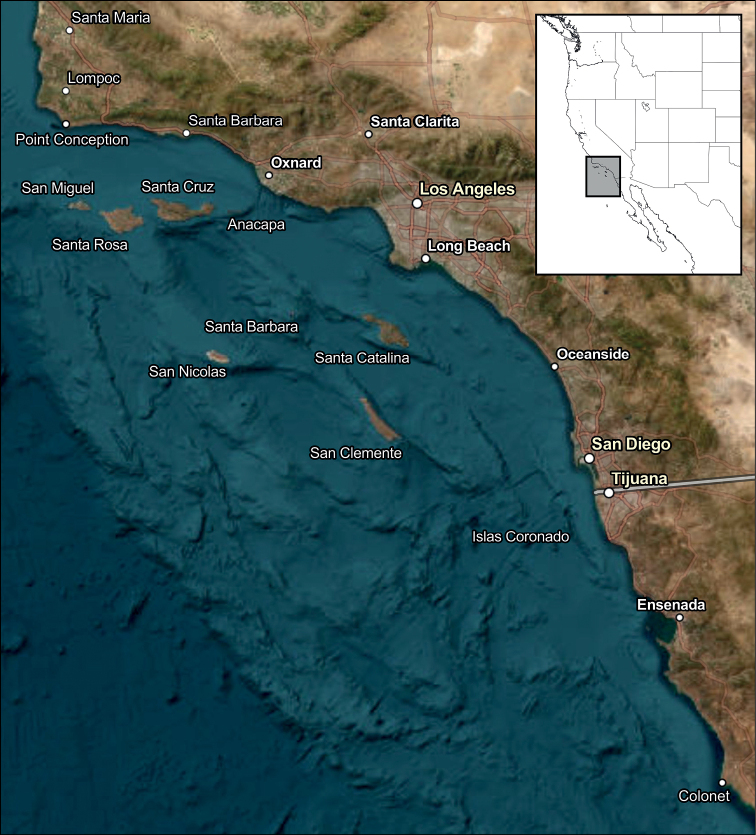
Southern California Bight region from Point Conception, California, USA to Punta Colonet, Baja California, Mexico, including the eight Channel Islands and Islas Coronado.

The Southern California Bight (SCB) is an important ecological region and economic resource that extends more than 600 km from Point Conception, California, USA to Cabo Colonet, Baja California, Mexico in the northeastern Pacific Ocean (Fig. 1). Because of its diverse and productive coastal ecosystems (e.g., rocky and sandy beach intertidal habitats, marshes, bays, lagoons, and estuaries, nearshore kelp forests and reefs, and offshore soft-bottom and hard-bottom benthic habitats of the continental shelf, slope, basins, and submarine canyons), and its proximity to dense human populations and associated pollutant inputs, the SCB is the focus of some of the largest and most comprehensive ocean monitoring programs in the world (Schiff et al. 2002, 2016, 2019). A key component of these programs is documenting changes in bottom dwelling invertebrate communities over space and time. In the SCB these include long-term localized programs conducted by four major wastewater discharge agencies (see City of San Diego 2018; Los Angeles County Sanitation Districts 2020; City of Los Angeles 2021; Orange County Sanitation District 2022), as well as broader regional programs that have been conducted every 4 or 5 years since 1994 (e.g., Allen et al. 1998, 2002, 2007, 2011; Bergen et al. 1998; Ranasinghe et al. 2003, 2007, 2012; Gillett et al. 2017, 2022; Walther et al. 2017; Wisenbaker et al. 2021). Key to the success of these and other programs and surveys is assuring taxonomic standardization of the resultant macroinvertebrate data sets. Fortunately, the SCB has also been home to the Southern California Association of Marine Invertebrate Taxonomists (SCAMIT) since 1982, whose members have ensured the production of accurate and reliable information for the region’s macrobenthic invertebrate fauna since that time.

Isopoda (Crustacea: Peracarida) is a diverse and ancient order of crustaceans comprising more than 10,600 living marine, freshwater and terrestrial species known worldwide (see Boyko et al. 2008 onwards). Several recent reviews have focused on the diversity and distribution of the major groups of isopods throughout the world. These include global reviews of the biodiversity of freshwater isopods (Wilson 2008a), the marine isopods exclusive of the epicarideans and asellotes (Poore and Bruce 2012), the bopyrid and cryptoniscid isopods that are ectoparasitic on other crustaceans (McDermott et al. 2010; Williams and Boyko 2012), and the cymothoid isopods that are parasites of marine and freshwater fishes (Smit et al. 2014). Also see Smit et al. (2019) for a review of the current state of knowledge for the various parasitic crustacean taxa, including the isopods. Overall, the isopods are well represented in the SCB with more than 130 species listed in SCAMIT (2021).

There are a number of general monographs, natural history guides, taxonomic keys, and other works relevant to the coastal marine invertebrates of the northeastern Pacific Ocean that contain useful information on SCB isopods even though many are focused on regions further to the north or south and in the Gulf of California (e.g., Richardson 1905a; Ricketts and Calvin 1952, 1968; Menzies and Barnard 1959; Johnson and Snook 1967; Schultz 1969; Kozloff 1974, 1983, 1996; Miller 1975; Allen 1976; Brusca and Brusca 1978; Brusca 1980; Lee and Miller 1980; Ricketts et al. 1985; Hinton 1987; Wetzer and Brusca 1997; Wilson 1997; O’Clair and O’Clair 1998; Sept 2002, 2019; Brusca et al. 2004, 2007; Kerstitch and Bertsch 2007). Additional information is included in several species checklists (e.g., Leistikow and Wägele 1999; Espinosa-Pérez and Hendrickx 2001a, 2006; Schmalfuss 2003; Campos and Villarreal 2008). However, except for treatments of the southern California coastal shelf and submarine canyons from more than 50 years ago (Menzies and Barnard 1959; Schultz 1966), a more recent survey of the offshore benthos of the Santa Maria Basin and Western Santa Barbara Channel in the northern SCB (Wetzer and Brusca 1997; Wilson 1997), and a guide to the intertidal and supralittoral species of the California and Oregon coasts (Brusca et al. 2007), most information on the coastal isopods of the region remains scattered amongst various taxonomic or ecological contributions. Consequently, there is presently no single comprehensive treatment of the SCB coastal isopod fauna.

The purpose of this guide is to review all macrobenthic species of isopods known or expected to occur in littoral or sublittoral marine habitats of the SCB. Most of these isopods are free-living species that inhabit soft or hard bottom habitats ranging from the upper intertidal to offshore continental shelf and upper slope (depths < 500 m), as well as inland bays and estuaries. Additional species that occur in deeper waters of the lower continental slope, nearshore basins, submarine canyons, and around oceanic islands in the region are also included. Some species are typically associated with more specific microhabitats or niches. For example, these include isopods living on or within sponges (e.g., some sphaeromatids and asellotes), species living commensally with other isopods or echinoderms (e.g., some asellotes and idoteids), species living on or closely associated with kelp or other marine algae (e.g., many idoteids), species that burrow into wood, algal holdfasts or other substrates (e.g., limnoriids and some sphaeromatids), species that are micropredators or temporary parasites of fishes (e.g., aegids, cirolanids, corallanids, gnathiids), and species that are obligate parasites of other crustaceans (epicarideans) or fishes (cymothoids). Finally, although the focus of this review is on marine isopods, halophilic or semi-terrestrial species within the suborder Oniscidea that occur at or just above the high tide line in many SCB intertidal areas are also included.

**Figure 2.**
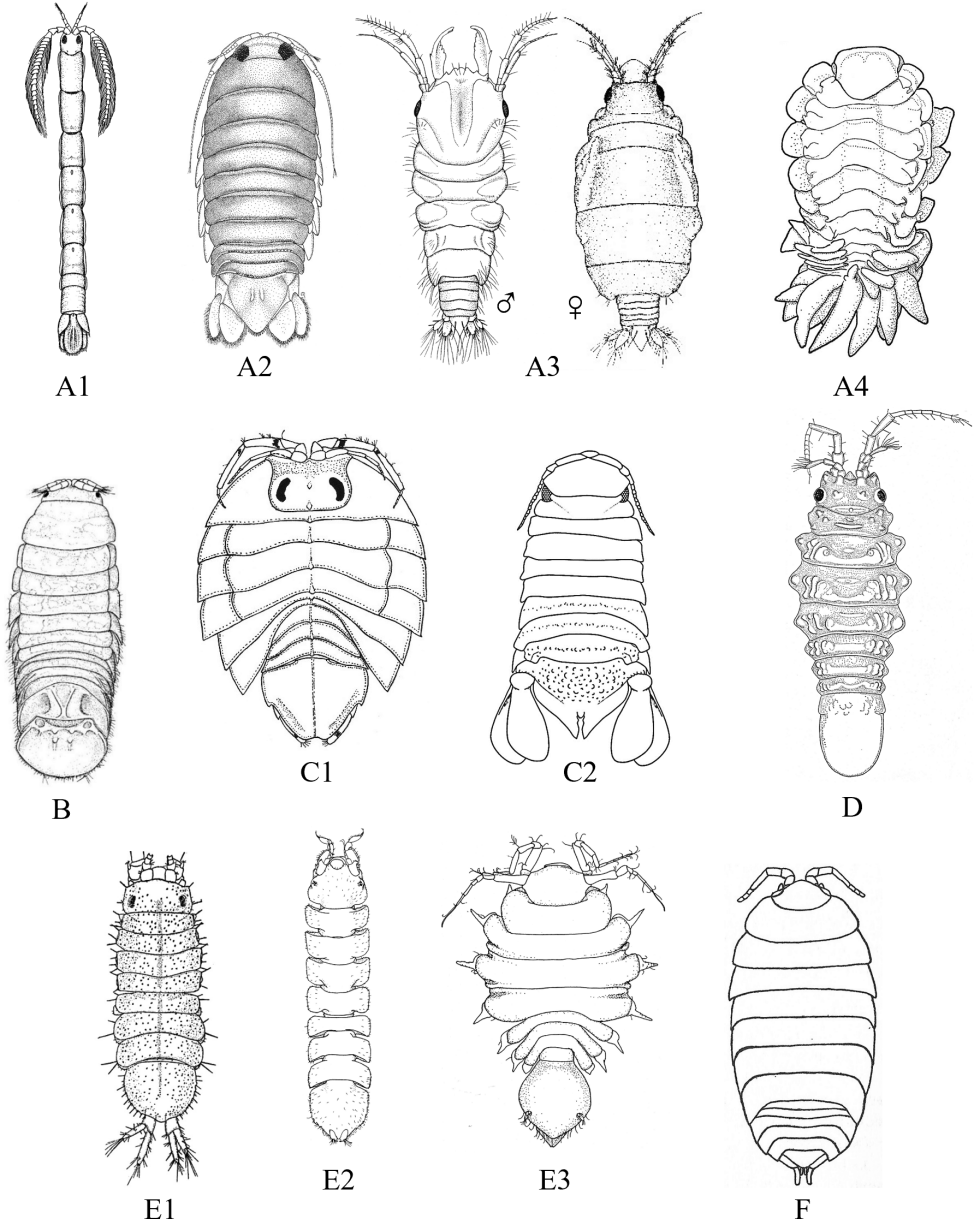
Examples of representative family-level body types for each of the six suborders of marine isopods occurring in the SCB. **A** suborder Cymothoida**A1**Anthuridae (*Haliophasmageminatum*) **A2**Cirolanidae (*Cirolanaharfordi*) **A3**Gnathiidae (*Gnathiatridens*, male and female) **A4**Bopyridae (*Munidionpleuroncodis*) **B** suborder Limnorioidea, Limnoriidae (*Limnoriaquadripunctata*) **C** suborder Sphaeromatidea**C1**Serolidae (*Heteroseroliscarinata*) **C2**Sphaeromatidae (*Dynoideselegans*) **D** suborder Valvifera, Idoteidae (*Synidoteamagnifica*) **E** suborder Asellota**E1**Janiridae (*Ianiropsisanaloga*) **E2**Joeropsididae (*Joeropsisconcava*) **E3**Paramunnidae (*Pleurogoniumcaliforniense*) **F** suborder Oniscidea, Alloniscidae (*Alloniscusperconvexus*).

A key to the suborders and superfamilies of marine isopods occurring in the SCB is provided, which is followed by nine subsequent keys that identify the local isopod fauna to species. Some representative body types for the major groups are illustrated in Fig. 2. Dorsal whole-body illustrations are included for most species. Unless otherwise noted, these figures were reproduced or modified after original species descriptions or other sources as credited in their respective captions and the list of references. Additional figures illustrating enlarged views of specific diagnostic features referred to in the keys are provided where possible. For provisional (undescribed) species where no figures exist, an image of a representative congener is included if available. A series of endnotes to these keys is provided that includes comprehensive additional information useful in the identification or classification of specific taxa, but which does not fit neatly in the annotated list of species. This list follows the endnote section and includes bathymetric and geographic distribution information, type locality, brief habitat notes, maximum body size, and the primary taxonomic, biogeographical, and ecological literature references for each species. Our effort focuses on summarizing and organizing current biodiversity data for the SCB marine isopods and is our best attempt at bringing together their morphological, habitat, and distributional data. Although by no means exhaustive, we recognize that available illustrations in many instances are less than ideal. Presenting primarily dorsal whole-body figures is a means of standardizing taxonomic diversity and aiding in the identification of different species, but we acknowledge it is not always the best depiction of a given taxon. Other views are often readily available, and we encourage the reader to refer to the cited and original descriptions for greater detail.

## ﻿Materials and methods

### ﻿Species review

All species currently recognized by the Southern California Association of Marine Invertebrate Taxonomists (SCAMIT 2021), as well as previously by the organization (SCAMIT 1994–2018) were reviewed and changes (e.g., additions, deletions, synonymies) verified. Additional species were added based on reviews of original species descriptions, taxonomic monographs, species or habitat guides, ecological studies, and published reports of site-specific or regional surveys conducted in the Southern California Bight (SCB) or elsewhere, as well as by personal observations and examination of field or museum specimens by the authors.

### ﻿Classification and terminology

The higher-level classification of crustaceans and isopods has evolved over the past two decades, with 11 isopod suborders currently recognized, of which eight include marine species (e.g., see Martin and Davis 2001; Brandt and Poore 2003; Ahyong et al. 2011; Boyko et al. 2013; Wetzer et al. 2013; Martin et al. 2014; Wetzer 2015). The treatment followed herein for the SCB isopods reflects that presently accepted by the World Register of Marine Species (WoRMS: Boyko et al. 2008 onwards) and SCAMIT (2021), with any differences between these lists addressed in the endnotes or annotated species list following the keys.

The Isopoda Latreille, 1817 can be distinguished from the other peracarid orders, and crustaceans in general, by the following combination of characters (after Brusca and Iverson 1985; Wetzer et al. 1997; Brusca et al. 2007).

1. Body usually dorsoventrally depressed but may be cylindrical or tubular in some suborders (e.g., Anthuridea and Phreatoicidea).
2. Body without a carapace but with a cephalic shield.
3. Head (cephalon) compact, typically with compound eyes, two pairs of antennae (first pair minute in Oniscidea), and mouthparts comprising one pair of mandibles, two pairs of maxillae, and one pair of maxillipeds.
4. Compound eyes sessile (not stalked) but may be on ocular lobes in some Asellota, Cymothoida (i.e., Gnathiidae), and Valvifera.
5. First and second antennae (antennules and antennae, respectively) uniramous, but with minute scales in a few taxa.
6. Mandible usually with a 1- to 3-articulate palp and a multidentate incisor process, left and right lacinia mobilis often differ, molar process highly variable.
7. First and second maxillae (maxillules and maxillae, respectively) without palps.
8. First thoracic appendages (thoracopods) modified as maxillipeds comprising fifth pair of mouthparts.
9. Long thorax of eight segments (thoracomeres), the first (and second in Gnathiidae) fused to the cephalon and bearing the maxillipeds, the remaining seven segments (pereonites) being free and collectively comprising a region called the pereon.
10. Pereon with seven pairs of uniramous legs (pereopods), all generally alike; the exception being the Gnathiidae in which only five pairs of walking legs are present.
11. Abdomen (pleon) relatively short comprising six somites (pleonites), at least one of which is always fused to the telson to form a pleotelson.
12. Six pairs of biramous pleonal appendages, including five pairs of plate-like pleopods specialized for respiration and/or swimming, and one pair of fan-like or stick-like, uniarticulate uropods.
13. Heart located primarily in the pleon.
14. Young isopods develop within a brood pouch (female’s marsupium) and emerge as a manca before appearance of the last pair of pereopods. Mancas have six pairs of pereopods.
15. Biphasic molting in which the posterior half of the body molts before the anterior half.

In terms of reproductive status, isopods can be sexed based on the presence or absence of male or female secondary sex characters. If oostegites (or a marsupium) are present with or without eggs or developing embryos, an individual is obviously female. If oostegites are absent, males can be distinguished by the presence of paired penes (may be fused) on the sternum of pereonite 7 (or pleonite 1) and/or appendices masculinae on the endopods of the second pleopods. Absence of all these characters indicates that the individual is either a juvenile, an immature female, or an immature male that has not yet developed secondary sexual features.

**Figure 3.**
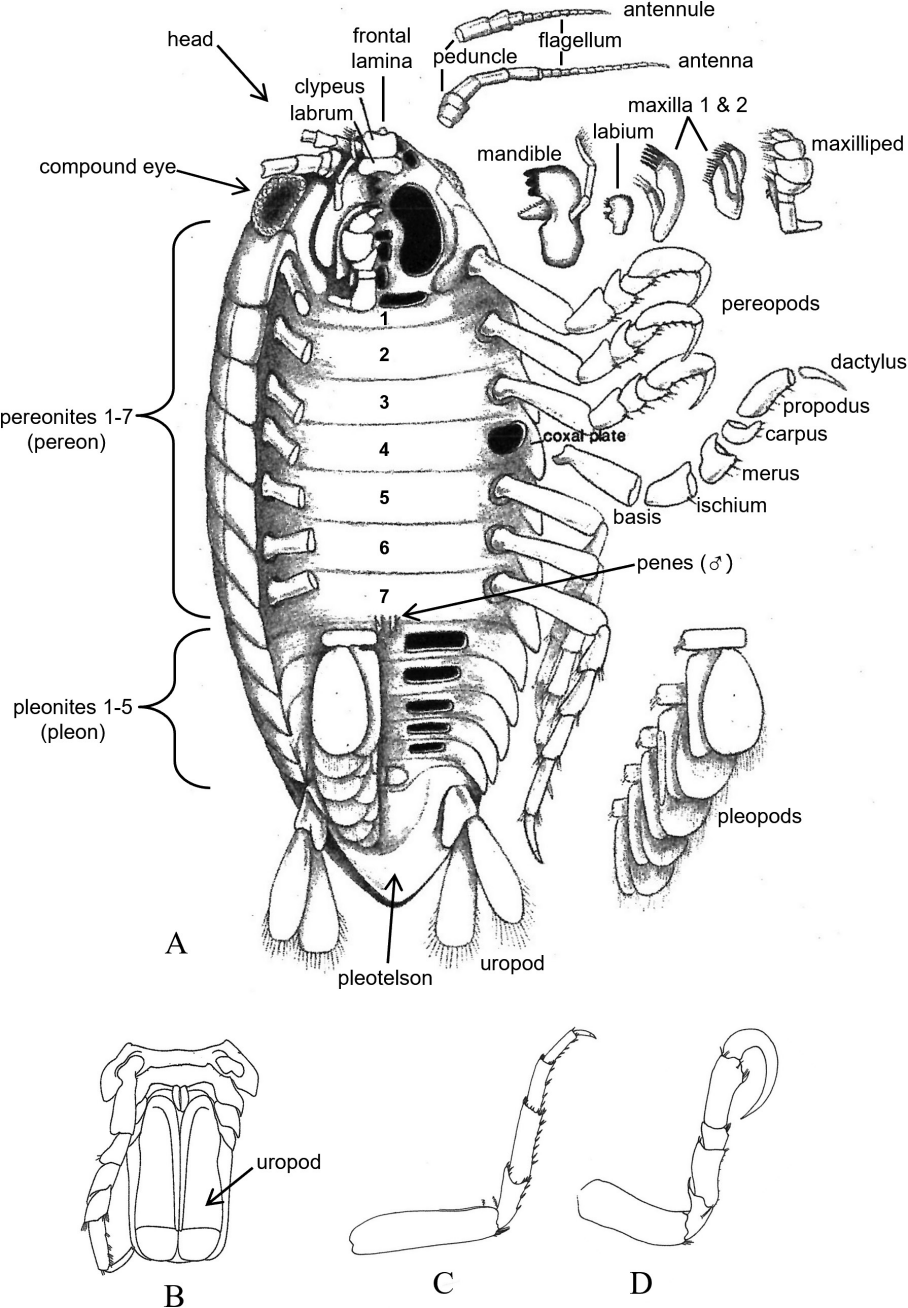
**A** Generalized isopod body plan (modified after Kensley and Schotte 1989: fig. 2) **B** ventral view of valviferan pleon and pleotelson showing opercular uropods (after Poore and Lew Ton 1993: fig. 25) **C** example of ambulatory pereopod (after Brusca 1983a: fig. 11G) **D** example of prehensile pereopod (after Brusca 1983a: fig. 11F).

**Figure 4.**
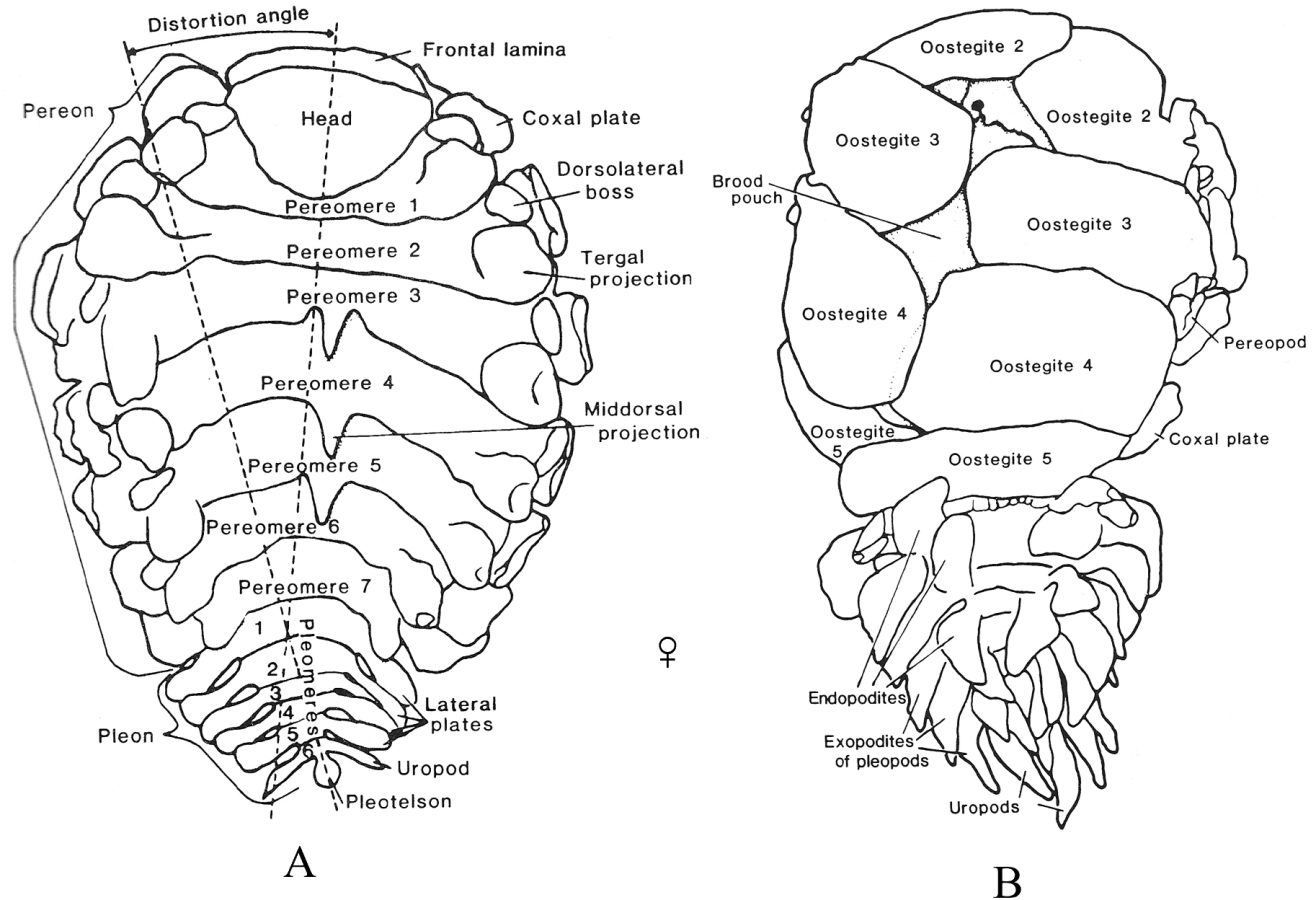
Bopyrid isopod body plan (adult female). **A** dorsal view **B** ventral view. Modified after Markham (1985a: fig. 2).

Terminology in the keys follows that which is typical for isopods in general (see Brusca and Iverson 1985; Kensley and Schotte 1989; Brusca and Wilson 1991; Wetzer et al. 1997; Brusca et al. 2007). The generalized isopod body plan characteristic of most groups is diagrammed in Fig. 3A, while Fig. 4 shows the specialized body plan characteristic of an adult female bopyrid (i.e., representative epicaridean). See Cohen and Poore (1994: fig. 1) for a detailed diagram of a stylized male gnathiid isopod.

In general, the isopod body is divided into three regions: the cephalon (head), pereon, and pleon. The cephalic region is referred to as “head” throughout the following keys. The segments of the pereon are referred to as pereonites 1–7 for most taxa or as pereomeres 1–7 for the epicarideans. Likewise, the segments of the pleon are referred to as pleonites 1–5 and the pleotelson (incorporating the fused pleonite 6 and telson) for most taxa (unless fused into fewer segments) or as pleomeres 1–6 plus telson for the epicarideans. The appendages of the pereon are numbered as pereopods 1–7, while the appendages of the pleon are referred to as pleopods 1–5 plus the uropods for all taxa. The first antennae (antenna 1) and second antennae (antenna 2) are referred to as antennules and antennae, respectively. Additional terminology is defined in glossaries of technical terms provided in Martin et al. (2022). Other useful glossaries are included in Kensley and Schotte (1989) and Wetzer et al. (1997), while Brusca and Wilson (1991) provide an extensive detailed description of isopod morphology. Key references regarding taxonomy, systematics, biogeography, and ecology of various isopod taxa are listed below for each suborder represented in this review, while specific references for each species are provided in the annotated species list.

**Suborder Cymothoida**: Primary references for the superfamily Anthuroidea include Menzies and Barnard (1959), Poore (1975, 1980, 1984a, 1984b, 2001a), Wägele (1984a), Poore and Lew Ton (1986, 1988a, 1988b, 1988c, 1988d), Cadien and Brusca (1993), and Annisaqois and Wägele (2021). Key references for the superfamily Cymothooidea are available for the families Aegidae (e.g., Brusca 1983a; Brusca and France 1992; Bruce 2009a), Cirolanidae (e.g., Bruce 1981; Brusca et al. 1995), Corallanidae (e.g., Delaney 1984, 1989), Cymothoidae (e.g., Brusca 1981; Bruce 1990; Smit et al. 2014; Hata et al. 2017), Gnathiidae (e.g., Cohen and Poore 1994; Smit and Davies 2004; Haney 2006; Tanaka 2007; Wilson et al. 2011), and Tridentellidae (e.g., Bruce 1984; Delaney and Brusca 1985). Within the infraorder Epicaridea, the family Bopyridae has been treated in detail in a long series of papers by Markham since the 1970s (e.g., Markham 1974a, 1975, 1977a, 1985a, 1986, 1992, 2001, 2003, 2004, 2008, 2016, 2020), while Boyko and Williams (2021a, 2021b) recently covered the two known local genera of the family Dajidae. Other valuable recent references for bopyrid and dajid isopods include Boyko et al. (2017), Williams et al. (2019), and Williams and Boyko (2021).

**Suborder Limnoriidea**: Key references for the family Limnoriidae include Menzies (1951a, 1957, 1958), Cookson (1991), and Borges et al. (2014).

**Suborder Sphaeromatidea**: Key references for the family Serolidae include Sheppard (1933), Harrison and Poore (1984), Brandt (1988), Wägele (1994), and Bruce (2009b), while important references for the family Ancinidae include Holmes and Gay (1909), Menzies and Barnard (1959), Schultz (1973), Glynn and Glynn (1974), Bruce (1993), and Shimomura (2008). Key references for the Sphaeromatidae include Menzies (1954), Harrison and Holdich (1982, 1984), Harrison and Ellis (1991), Bruce (1993), Schotte (2005), Wetzer et al. (2013, 2018, 2021), Wall et al. (2015), and Wetzer and Mowery (2017).

**Suborder Valvifera**: Key references for the valviferans include Menzies (1950a, 1951b), Sheppard (1957), Menzies and Barnard (1959), Menzies and Miller (1972), Brusca and Wallerstein (1977), Brusca (1983b, 1984), Brandt (1990), Poore and Lew Ton (1990, 1993), Rafi and Laubitz (1990), Wägele (1991), Poore (2001b, 2015), and King (2003).

**Suborder Asellota**: Key references for the asellotes include Menzies (1951b, 1952), Menzies and Barnard (1959), George and Strömberg (1968), Hessler et al. (1979), Wilson and Hessler (1980, 1981), Poore (1984c), Wilson (1987, 1994, 1997, 2008b), Wilson and Wägele (1994), Serov and Wilson (1995), Just and Wilson (2004, 2007, 2021), Doti and Wilson (2010), and Brix et al. (2021).

**Suborder Oniscidea**: Useful references for the oniscids include Van Name (1936, 1940, 1942), Garthwaite et al. (1985, 1992), Garthwaite and Lawson (1992), Leistikow and Wägele (1999), Schmalfuss (2003), Schmidt and Leistikow (2004), Schmidt (2008), and Sfenthourakis and Taiti (2015).

## ﻿Results

### ﻿Biodiversity

A total of 190 species of isopods are covered in this review of the Southern California Bight (SCB) isopod fauna, representing 105 genera, 42 families, and six suborders (Appendix 1). These include all but two of the 132 species-level taxa currently recognized by SCAMIT (2021)^Endnote 1^. Approximately 84% of these isopods represent described species (see Boyko et al. 2008 onwards), while the remaining 16% represent well-documented “provisional” but undescribed species.

Of the six isopod suborders occurring in SCB coastal and offshore waters, the Cymothoida and Asellota are the most diverse, accounting for ~ 36% and 29% of the species, respectively (Table 1). The Valvifera and Sphaeromatidea are the next most speciose suborders, accounting for ~ 13–15% of species, while the suborder Limnorioidea is represented by only three species (< 2%) in SCB waters. Finally, the suborder Oniscidea is represented by nine species (~ 5%) that occur at the terrestrial-marine interface in several intertidal areas of the SCB. See Brusca et al. (2007) or contact R Wetzer for more complete lists of California oniscid isopods.

**Table 1.** Number of families, genera, and species for each of the six isopod suborders occurring in littoral and sublittoral marine habitats of the Southern California Bight. For suborders represented by more than one superfamily, the breakdown per superfamily is indicated.

Suborder/Superfamily	Number taxa per group		
	Families	Genera	Species
**Suborder Cymothoida**	**15**	**47**	**69**
Superfamily Anthuroidea	5	10	11
Superfamily Cymothooidea	6	18	38
Infraorder Epicaridea			
Superfamily Bopyroidea	2	16	17
Superfamily Cryptoniscoidea	2	3	3
**Suborder Limnoriidea**	**1**	**1**	**3**
**Suborder Sphaeromatidea**	**4**	**12**	**25**
Superfamily Seroloidea	1	1	1
Superfamily Sphaeromatoidea	3	12	24
**Suborder Valvifera**	**4**	**12**	**29**
**Suborder Asellota**	**12**	**26**	**55**
Superfamily Janiroidea	11	25	54
Superfamily Stenetrioidea	1	1	1
**Suborder Oniscoidea**	**6**	**6**	**9**
**Total taxa**	**42**	**105**	**190**

The suborder Cymothoida is represented by 69 species distributed amongst four superfamilies (Anthuroidea, Cymothooidea, Bopyroidea, Cryptnoniscoidea) and 15 families. The superfamily Anthuroidea, characterized by long, thin, cylindrical bodies usually at least 6 × longer than wide, includes 11 species in five families. Most anthuroids are thought to feed on other small invertebrates and occur in the SCB amongst fouling communities in marinas, bays, and harbors, and in both littoral and sublittoral habitats on the outer coast from the low intertidal to soft and hard-bottom benthos of the continental shelf, slope, and submarine canyons (0–1300 m depths).

The superfamily Cymothooidea is represented by six families with 38 species. Five of these families (Aegidae, Cirolanidae, Corallanidae, Cymothoidae, Tridentellidae) are generally similar in body form with sleek symmetrical bodies usually ~ 2–6 × longer than wide, the uropods and pleotelson forming a distinct tail fan, and the pereopods modified from ambulatory to prehensile reflecting their different lifestyles (e.g., Fig. 3C, D). For example, the family Cirolanidae is represented by seven species with mostly ambulatory pereopods, although pereopods 1–3 tend to have well-developed dactyli modified for grasping. In the SCB, these species occur intertidally on both sandy and rocky beaches, as well as in shallow to deep water habitats of the continental shelf, slope, and basins (0–1250 m depths). Corallanidae and Tridentellidae, represented by two species each in the SCB, are similar in shape to the cirolanids, but with the mouthparts and anterior pereopods modified for a predatory lifestyle. For example, the dactyli of pereopods 1–3 are usually as long or longer than the propodi and thus adapted for grasping prey. The two SCB corallanids occur from the intertidal to 138 m on the continental shelf, while the two SCB tridentellids have been reported from offshore depths of 53–360 m. The family Aegidae is represented by seven species in the SCB, all of which are temporary parasites of marine fishes and are characterized by prehensile pereopods 1–3 (dactylus strongly recurved and as long or longer than propodus). These species have been reported from the intertidal to deep waters of the continental shelf, slope, basins, and oceanic islands (0–2534 m depths). Although somewhat similar in general body form to the above four families, species of Cymothoidae are even more highly modified for a parasitic lifestyle with all seven pairs of pereopods being strongly prehensile. All cymothoids are obligate parasites of marine or freshwater fishes, most commonly being found attached in the gill chamber or buccal region of their host. Nine species of cymothoids are recognized herein as parasitizing a wide range of marine fishes in nearshore to offshore coastal waters of the SCB.

The sixth family of Cymothooidea occurring in SCB waters is Gnathiidae of which the males are highly modified and characterized by only six free pereonites and five pairs of pereopods. Eleven species of gnathiids are reported in the SCB, including three that are awaiting formal description. These 11 species occur in a wide range of habitats from the intertidal to shallow subtidal, and from the benthos of the offshore continental shelf, slopes, and submarine canyons to depths of ~ 1400 m.

Bopyroidea and Cryptoniscoidea comprise the remaining two superfamilies of Cymothoida, both of which are highly modified obligate parasites of other crustaceans. Bopyroidea is represented by 17 species in two families (Bopyridae and Ionidae) in shallow to deep SCB waters. Sixteen of these species are branchial parasites of a wide range of decapod crustaceans (e.g., hermit crabs, shrimp, mud shrimp, ghost shrimp, galatheid crabs, squat lobsters, porcelain crabs, grapsid crabs) while one species is an abdominal parasite of mud shrimp. In contrast, Cryptoniscoidea is represented by only three species in two families. These include Dajidae represented herein by at least two species that are ectoparasites on the dorsal carapace of several species of shrimp, and Hemioniscidae represented by a single species that is an ectoparasite of barnacles.

The suborder Limnoriidea is represented by three species of the family Limnoriidae in SCB waters. All three species occur in shallow waters (0–30 m depth) where they burrow into either wood (2 species) or algal holdfasts (1 species).

The suborder Sphaeromatidea is represented by 25 species in SCB waters distributed between two superfamilies (Seroloidea and Sphaeromatoidea) and four families. The serolids (family Serolidae) presently include only a single recognized species in the SCB, *Heteroseroliscarinata*, which burrows just beneath the sediment surface from shallow waters in bays and harbors, and offshore to depths of ~ 100 m. However, it is possible that shallow vs. deep water populations in the region represent two distinct species (TDS, pers. obs.). In contrast, the superfamily Sphaeromatoidea includes 24 species in three families. Of these, the family Sphaeromatidae is the most diverse, represented herein by a total of 20 species. Most of these species occur from intertidal to shallow subtidal habitats < 30 m depth, although two species, *Discerceisgranulosa* and *Paracerceisgilliana*, have been reported from slightly deeper waters between 37–73 m. The other two sphaeromatoid families, Ancinidae and Tecticipitidae, are represented by only three and one species, respectively. All four of these species occur in intertidal or shallow subtidal habitats (< 30 m depths).

The suborder Valvifera can be distinguished from all other local isopods by the possession of hinged opercular uropods that cover the ventral surface of the pleon and pleotelson enclosing the pleopods (see Fig. 3B). Four families and 29 species of valviferans are represented in SCB waters. Idoteidae is the most diverse of these families, comprising 23 species in the study area. Many of these species, especially within the genera *Colidotea*, *Erichsonella*, *Eusymmerus*, *Idotea*, *Pentidotea*, and *Stenosoma* are most common in intertidal and shallow subtidal habitats associated with various species of kelp or algae. In contrast, the two local species of *Edotia* occur in soft-bottom habitats of the continental shelf between depths of ~ 14–64 m, while three of the four SCB species of *Synidotea* (except *S.harfordi*) occur on the shelf or slope at depths of ~ 30–800 m. The remaining three families of SCB valviferans comprise a total of six species. The family Arcturidae is represented by four species that occur in the low intertidal (1 species) or offshore shelf benthos (3 species) at depths < 100 m. The last two families are each represented by a single species. These include *Cleantioidesoccidentalis* of the Holognathidae in relatively shallow waters (intertidal to ~ 50 m), and *Califarcturustannerensis* of the Thermoarcturidae in deep waters (~ 1200–1300 m).

The suborder Asellota is represented by 55 species in SCB waters distributed between two superfamilies (Janiroidea and Stenetrioidea) and 12 families. However, only ~ 64% of these species are formally described, with the remaining 36% representing provisional species (see Appendix 1). Stenetrioidea includes a single species (*Stenetrium* sp. A) reported from 90–131 m in the Santa Maria Basin. In contrast, Janiroidea is represented by a diverse group of 11 families that occur in a wide range of habitats and depths from the intertidal to nearly 4000 m. Janiridae is the most diverse of these families, comprising 18 species; ca. two-thirds of the janirids occur in shallow waters from the intertidal to depths of ~ 30 m, while the remaining third occur on the continental shelf at depths between ~ 30–200 m. Paramunnidae is the next most diverse family with nine species, two of which occur in the shallow subtidal (9–20 m) and seven on the continental shelf (75–197 m). Munnidae is the third most diverse family with eight species, of which five species occur in intertidal to shallow subtidal habitats, two species occur at mostly shelf depths (12–237 m), and one species occurs in deeper slope waters (500 m). The Munnopsidae is represented by six species that occur at depths between 73–1118 m. The Joeropsididae is represented by five species that occur at depths from the intertidal to 161 m, while the Desmosomatidae includes three species that range in depth from ~ 100–3000 m. The remaining five families are each represented by a single species. These include Dendrotion­idae (*Acanthomunnatannerensis* at ~ 600–800 m), Haplomunnidae (*Haplomunnacaeca* at ~ 4000 m), Lepidocharontidae (*Microcharon* sp. A at 75 m), Nannoniscidae (*Nannonisconuslatipleonus* at ~ 300–500 m), and Pleurocopidae (*Pleurocope* sp. A at < 1 m).

The suborder Oniscidea is represented in this review by nine species distributed between six families. Each of these species typically occurs at or above the high tide mark in its respective habitat. The families Alloniscidae (2 species) and Tylidae (1 species) occur on sandy beaches in the SCB. The Detonidae (3 species) and Halophiloscidae (1 species) both occur in marshes, bays, and estuaries. The Platyarthridae (1 species) is reported to occur on both sandy beaches and at the edges of marshes. The Ligiidae is represented herein by a single species that typically occurs in the spray zone on rocky intertidal shores.

### ﻿Keys to species

Ten keys were constructed to facilitate identification of the 190 species of isopods included in this guide. Key A represents a key to the suborders and main superfamilies, which are then identified to species in Keys B–J. Keys B–E cover the suborder Cymothoida (superfamilies Anthuroidea, Cymothooidea, Bopyroidea, and Cryptoniscoidea). Key B covers the Anthuroidea (5 families, 11 species). Key C covers the families Aegidae, Cirolanidae, Corallanidae, Cymothoidae, and Tridentellidae of Cymothooidea (27 species). Key D covers the remaining cymothooidean family, Gnathiidae (11 species). Key E covers the epicaridean superfamilies Bopyroidea and Cryptoniscoidea (4 families, 20 species). Key F covers the suborder Limnoriidea (1 family, 3 species). Key G covers the superfamilies Seroloidea and Sphaeromatoidea of the suborder Sphaeromatidea (4 families, 25 species). Key H covers the suborder Valvifera (4 families, 29 species). Key I covers the superfamilies Janiroidea and Stenetrioidea of the suborder Asellota (12 families, 55 species). Key J covers the suborder Oniscidea (6 families, 9 species).

### ﻿Key A. Suborders and Superfamilies of SCB Marine Isopods

**Table:** 

1	Adult isopods free-living, but may live commensally with other isopods or invertebrates; females and males with clear bilateral symmetry, typically similar in size; antennae well developed, never vestigial; antennules variable	**2**
–	Adult isopods obligate parasites of other crustaceans; females with slightly to highly distorted or reduced bilateral symmetry; male minute, bilaterally symmetrical, living on body of adult female; antennae vestigial in female; antennules reduced to ≤ 3 articles [Suborder Cymothoida: Superfamilies Bopyroidea and Cryptoniscoidea]	**Key E**
2	Adult isopods with 5 pairs of pereopods (thoracomere 2 fused to cephalon with its appendages modified into pylopods; thoracomere 8 reduced, without legs); adult males with enlarged, forward projecting forceps-like mandibles; females without mandibles [Suborder Cymothoida: Superfamily Cymothooidea (in part), Family Gnathiidae]	**Key D**
–	Adult isopods usually with 7 pairs of distinct pereopods (7^th^ pair may be small and folded against ventral body wall in juveniles); newly released isopods (mancas) from the marsupium with only 6 pairs of pereopods; males without projecting forceps-like mandibles; females with mandibles	**3**
3	Species terrestrial or halophilic, mostly restricted to upper littoral (e.g., high tide line, spray zone) or brackish water habitats along coast; antennules vestigial, minute; pleon always composed of 5 free pleonites plus pleotelson [Suborder Oniscidea: Superfamily Oniscoidea]	**Key J**
–	Species fully marine, occurring in littoral or sublittoral habitats; antennules normal, or not minute if reduced; pleon variable, with or without fused pleonites	**4**
4	Uropods operculate, modified into pair of ventral covers (opercula) enclosing the pleopods (Fig. 3B), but do not confuse with operculate pleopods [Suborder Valvifera]	**Key H**
–	Uropods not modified as ventral opercula, hinged laterally or terminally on pleotelson	**5**
5	Uropods typically flattened and hinged on anterolateral margins of pleotelson (may be greatly reduced)	**6**
–	Uropods styliform and hinged terminally or nearly so on posterior margins of pleotelson [Suborder Asellota: Superfamilies Janiroidea and Stenetrioidea]	**Key I**
6	Adult body elongated, usually > 6 × longer than wide [Suborder Cymothoida: Superfamily Anthuroidea]	**Key B**
–	Adult body not elongated, < 4 × longer than wide (not elongated)	**7**
7	Uropods greatly reduced with small claw-like exopods, generally not visible dorsally; species burrow in wood or algal holdfasts [Suborder Limnoriidea: Superfamily Limnorioidea]	**Key F**
–	Uropods not as above, clearly visible dorsally as expanded, flattened “tail fan” or long caudal processes	**8**
8	Pleon composed of 4 or 5 free pleonites plus pleotelson [Suborder Cymothoida: Superfamily Cymothooidea (in part), Families Aegidae, Cirolanidae, Corallanidae, Cymothoidae, and Tridentellidae]	**Key C**
–	Pleon composed of ≤ 3 free pleonites plus pleotelson [Suborder Sphaeromatidea: Superfamilies Seroloidea and Sphaeromatoidea]	**Key G**

### ﻿Key B. Suborder Cymothoida, Superfamily Anthuroidea

Figs 5, 6

**Figure 5.**
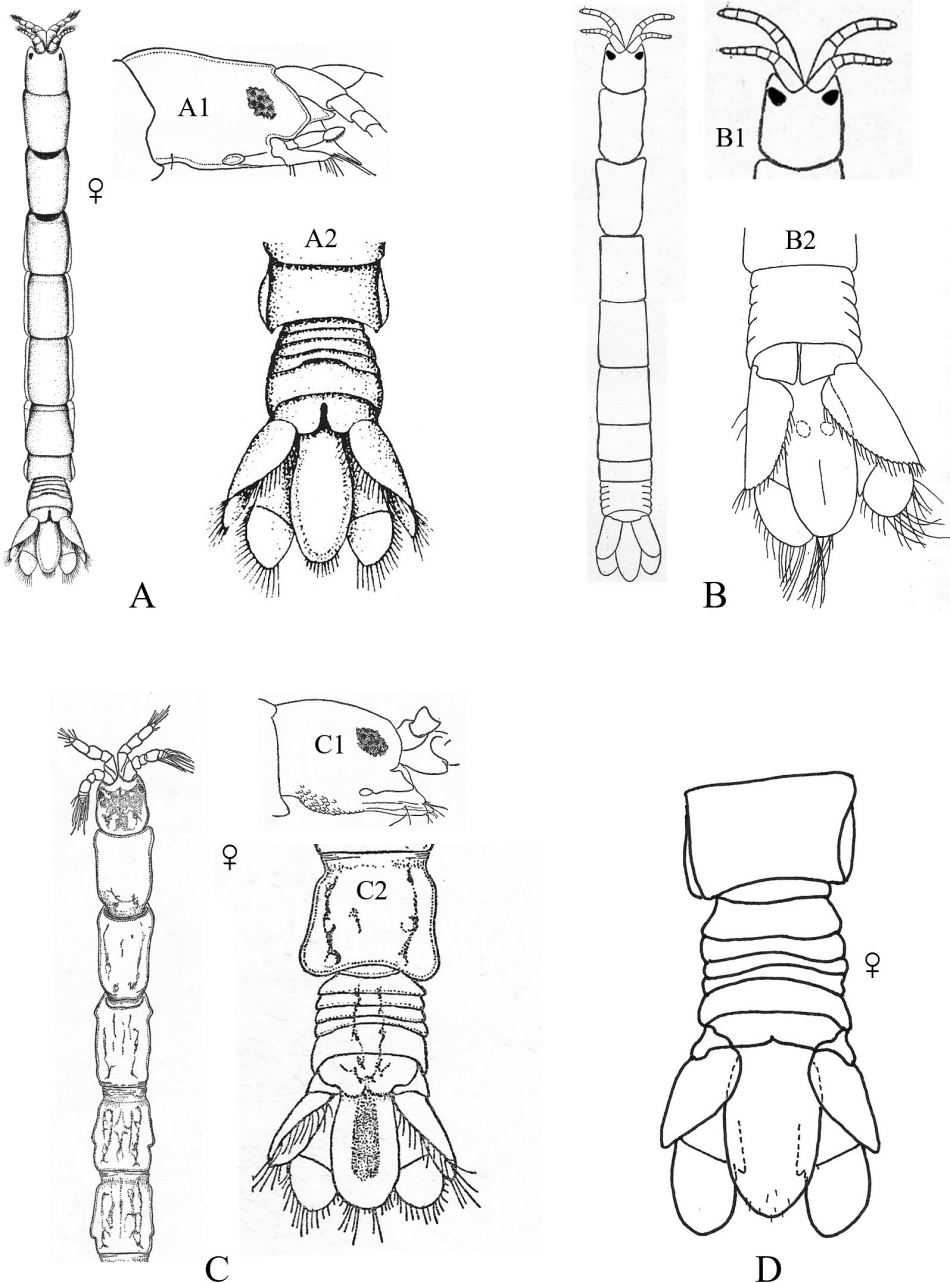
Isopoda, Cymothoida, Anthuroidea, Paranthuridae: **A***Paranthuraelegans***A1** lateral view of head (piercing mouthparts) **A2** dorsal view of pereonite 7, free pleonites 1–5, pleotelson and uropods (after Menzies 1951b; Wetzer and Brusca 1997) **B***Paranthurajaponica***B1** dorsal close-up view of head **B2** dorsal view of fused pleonites 1–5, pleotelson and uropods (after Richardson 1909; Brusca et al. 2007) **C***Califanthurasquamosissima* (male with antenna loaded with aesthetascs) **C1** lateral view of head (piercing mouthparts) **C2** dorsal view of pereonites 6 and 7, dorsally fused pleonites 1–5, pleotelson and uropods (after Menzies 1951b) **D***Colanthurabruscai*, dorsal view of pereonites 6 and 7, free pleonites 1–5, pleotelson and uropods (after G. Poore, personal contribution).

**Figure 6.**
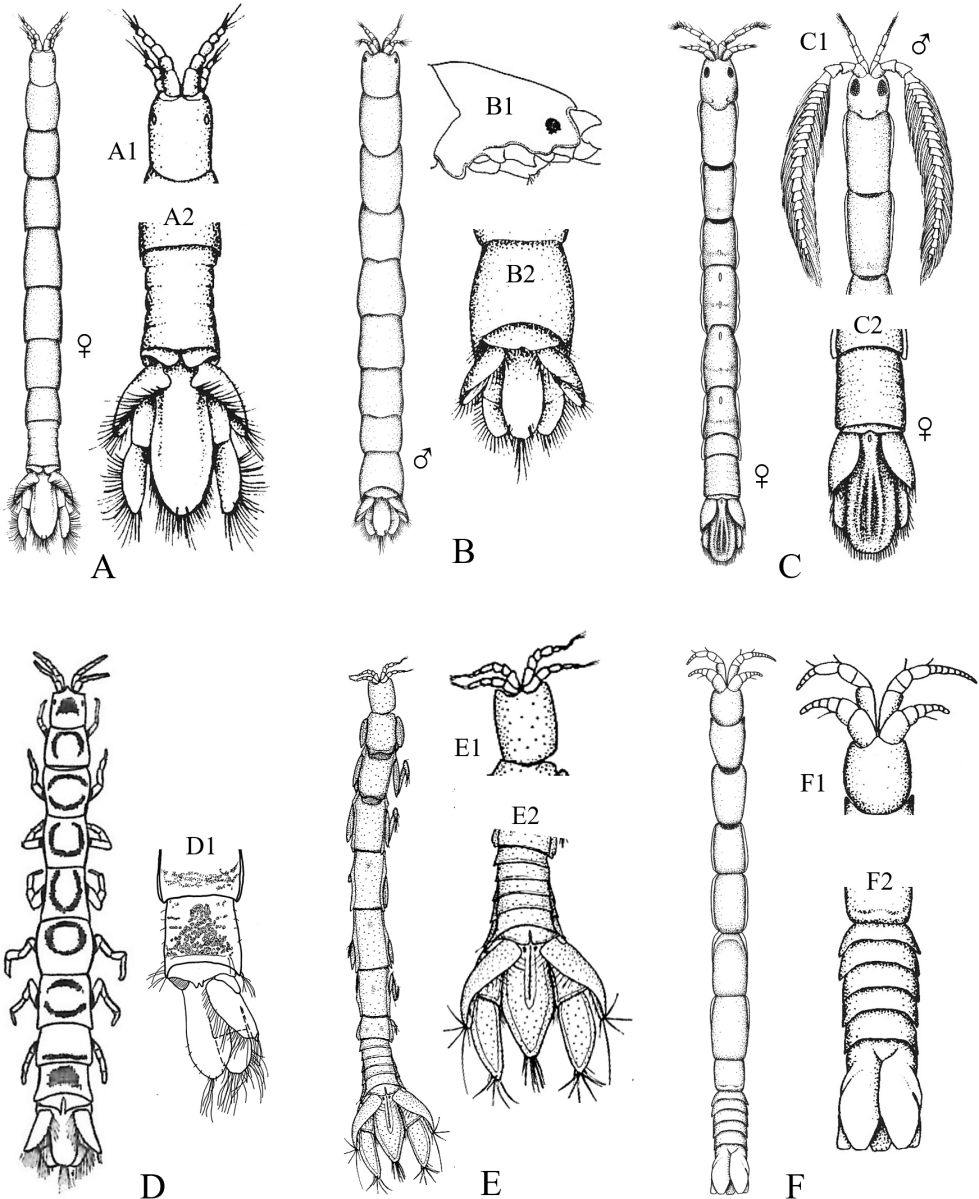
Isopoda, Cymothoida, Anthuroidea, Anthuridae: **A***Amakusanthuracaliforniensis***A1** dorsal close-up view of head **A2** dorsal view of pereonite 7, dorsally fused pleonites 1–5, pleotelson and uropods (after Wetzer and Brusca 1997) **B***Cyathuramunda***B1** lateral view of head (biting mouthparts) **B2** dorsal view of pereonite 7, dorsally fused pleonites 1–5, pleotelson and uropods (after Menzies 1951b) **C***Haliophasmageminatum***C1** dorsal view of head and pereonites 1 and 2 of male **C2** dorsal view of pereonite 7, dorsally fused pleonites 1–5, pleotelson and uropods (after Wetzer and Brusca 1997) **D***Mesanthuraoccidentalis***D1** dorsal view of pereonite 7, dorsally fused pleonites 1–5, pleotelson and right uropod (after Allen 1976). Antheluridae: **E***Ananthuraluna***E1** dorsal close-up view of head **E2** dorsal view of pereonite 7, free pleonites 1–5, pleotelson and uropods (after Schultz 1966). Hyssuridae: **F***Kupellonura* sp. A **F1** dorsal close-up view of head **F2** dorsal view of pereonite 7, free pleonites 1–5, pleotelson and uropods (after Wetzer and Brusca 1997).

**Table:** 

1	Eyes present	**2**
–	Eyes absent	**10**
2	Mouthparts form forward directed cone-like structure under the head, adapted for piercing and sucking	**3** [Paranthuridae]
–	Mouthparts not forming ventral cone-like structure, adapted for biting and chewing	**6**
3	Pereon composed of 7 distinct, well-developed pereonites with 7 pairs of pereopods; pereonite 7 ca. half as long as pereonite 6 and visible laterally	**4**
–	Pereon composed of 6 distinct pereonites with 6 pairs of pereopods (7^th^ pereopods absent); pereonite 7 very short, < 20% as long as pereonite 6, not visible laterally	**5**
4	Pleonites 1–5 free, not fused; pleonite 5 ca. 3 × longer than other pleonites (Fig. 5A)	***Paranthuraelegans***
–	Pleonites 1–5 fused mid-dorsally, but distinct laterally; all pleonites of similar length (Fig. 5B)	***Paranthurajaponica***
5	Pleonites 1–5 free, separated from each other by dorsal integumental folds; pleonite 1 ca. 2 × longer than pleonite 2 (Fig. 5D)	***Colanthurabruscai***
–	Pleonites 1–5 dorsally fused, all pleonites of similar length (Fig. 5C)	***Califanthurasquamosissima***
6	Dorsal surface of pleotelson with median row of spines; uropodal and pleotelsonic margins serrated^Endnote 2^	***Eisothistos* sp. A** [Expanathuridae]
–	Dorsal surface of pleotelson smooth or ridged, without spines; uropodal and pleotelsonic margins not serrated	**7** [Anthuridae]
7	Pleotelson with 3 raised dorsal longitudinal ridges or carinae; uropodal exopods curve up and over base of pleotelson (Fig. 6C)	***Haliophasmageminatum***
–	Pleotelson without dorsal ridges or carinae; uropodal exopods may or may not curve up over pleotelson	**8**
8	Pleonites 1–5 fused only along dorsal midline, segments free laterally and visible dorsally; uropodal exopods ca. half length of endopods and pleotelson, curving up and partially over pleotelson base; uropodal endopods narrow, ca. half as wide as pleotelson (Fig. 6A)	***Amakusanthuracaliforniensis***
–	Pleonites 1–5 completely fused dorsally; uropodal exopods > 50% length of endopods and pleotelson, may or may not cover the pleotelson dorsally; uropodal endopods broad, subequal in width to pleotelson	**9**
9	Dorsal surface of pereon pigmented, with complete or nearly complete dark rings on pereonites 2–6 and posterior transverse band on pereonite 7; uropodal exopods partially cover dorsal surface of pleotelson (Fig. 6D)	***Mesanthuraoccidentalis***
–	Dorsal surface of pereon covered with diffuse pigment splotches, but without pigment rings; uropodal exopods do not cover base of pleotelson (Fig. 6B)	***Cyathuramunda***
10	Uropodal exopods with distinct lateral lobes, exopods overlapping broadly to cover almost entire dorsal surface of pleotelson; uropodal rami nearly reach, but do not exceed, posterior margin of pleotelson; tips of uropodal rami without tufts of stiff setae (Fig. 6F)	***Kupellonura* sp. A** [Hyssuridae]
–	Uropodal exopods without lateral lobes, overlapping only base of pleotelson; uropodal endopod distinctly longer than pleotelson; tips of uropodal rami with tufts of long stiff setae (Fig. 6E)	***Ananthuraluna*** [Antheluridae]

### ﻿Key C. Suborder Cymothoida, Superfamily Cymothooidea (in part): Families Aegidae, Cirolanidae, Corallanidae, Cymothoidae, Tridentellidae

Figs 7–11

**Figure 7.**
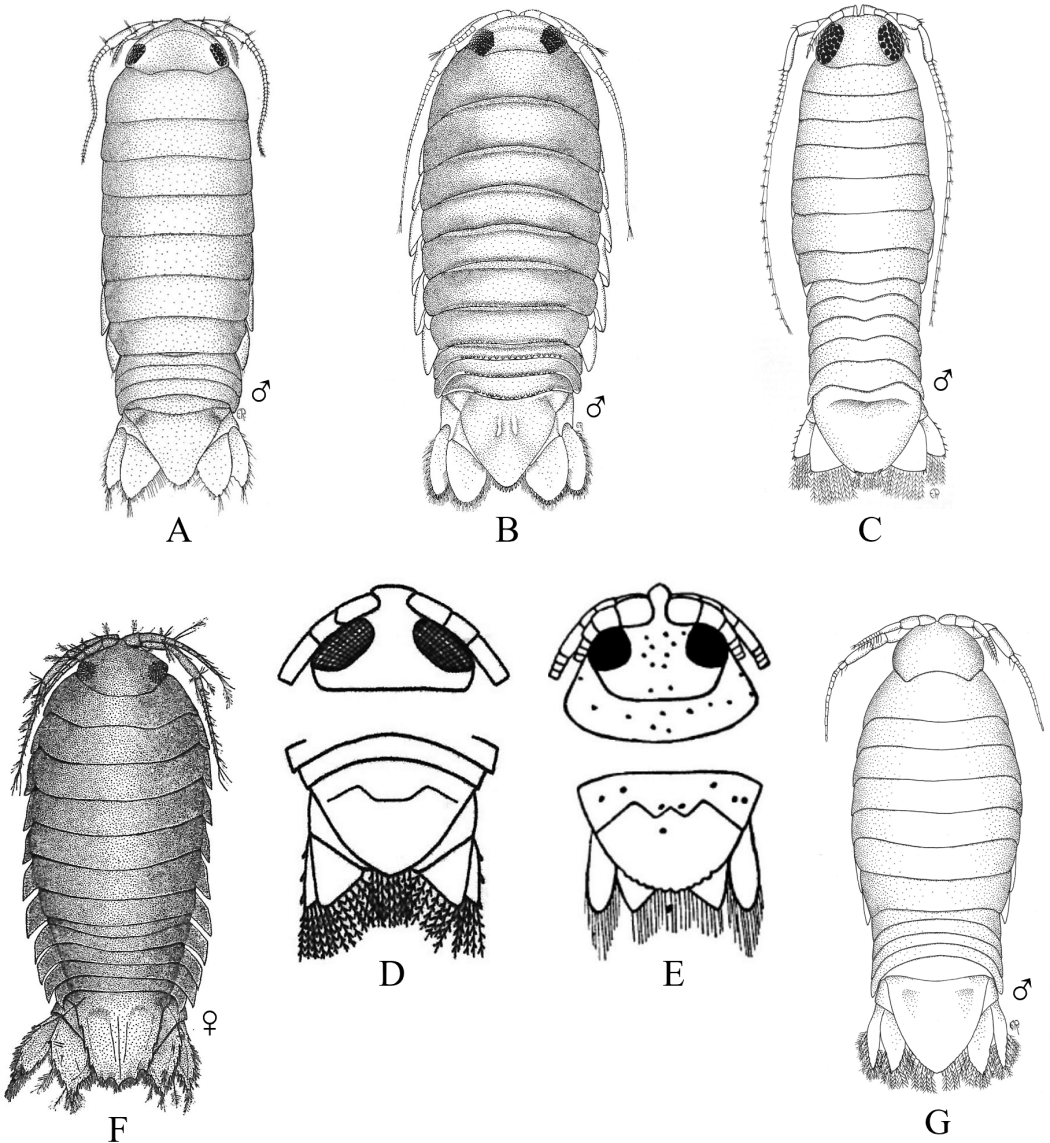
Isopoda, Cymothoida, Cymothooidea, Cirolanidae: **A***Cirolanadiminuta* (after Brusca et al. 1995) **B***Cirolanaharfordi* (after Brusca et al. 1995) **C***Eurydicecaudata* (after Brusca et al. 1995) **D***Excirolanachiltoni* (after Schultz 1969) **E***Excirolanalinguifrons* (after Schultz 1969) **F***Metacirolanajoanneae* (after Schultz 1966) **G***Natatolanacaliforniensis* (after Brusca et al. 1995).

**Figure 8.**
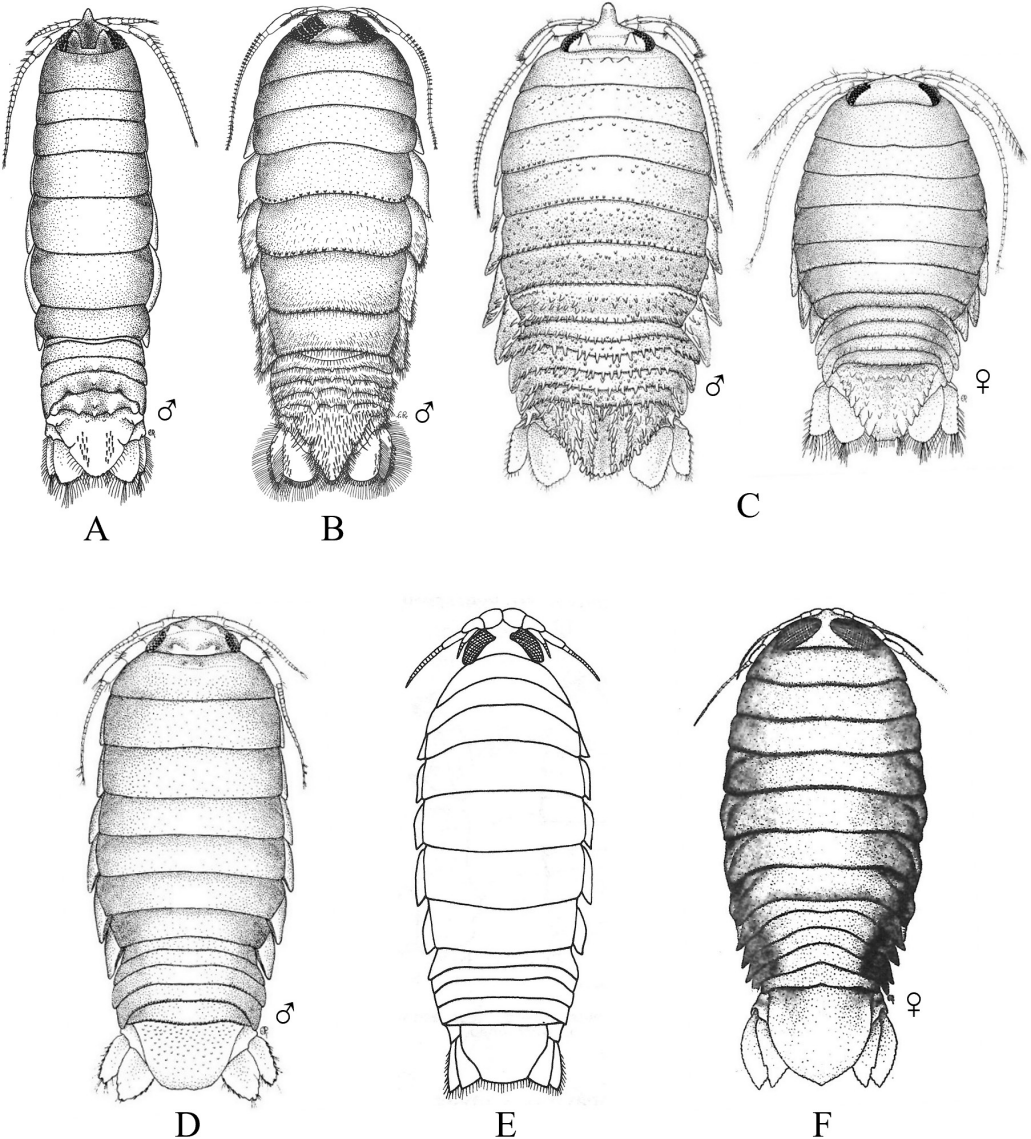
Isopoda, Cymothoida, Cymothooidea, Corallanidae: **A***Excorallanatricornisoccidentalis* (after Delaney 1984) **B***Excorallanatruncata* (after Delaney 1984). Tridentellidae: **C***Tridentellaglutacantha* (after Delaney and Brusca 1985) **D***Tridentellaquinicornis* (after Delaney and Brusca 1985). Aegidae (in part): **E***Aegalecontii* (after Schultz 1969) **F***Aegiochusplebeia* (after Brusca 1983a).

**Figure 9.**
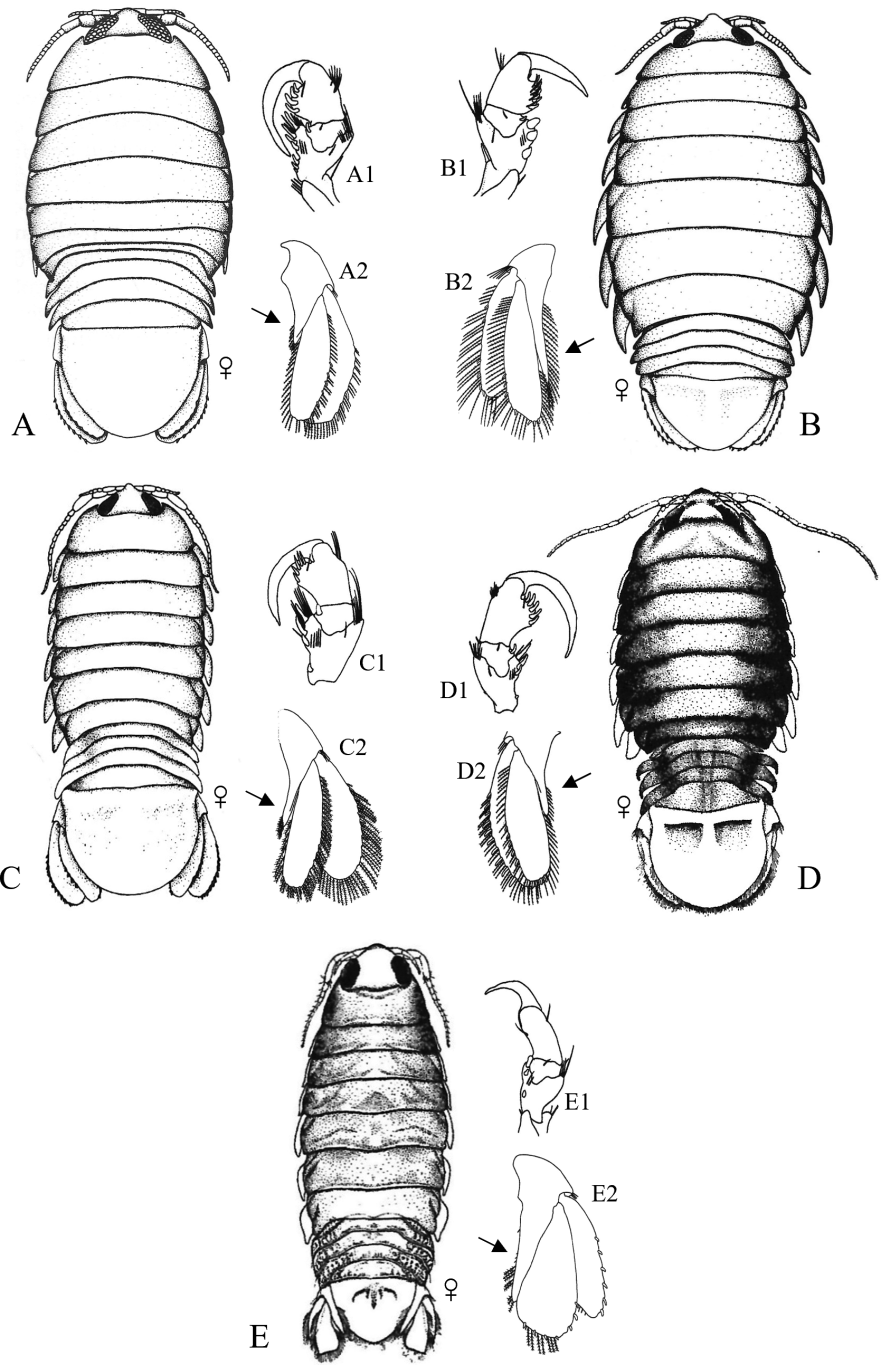
Isopoda, Cymothoida, Cymothooidea, Aegidae (in part): **A***Rocinelaangustata* (after Brusca and France 1992) **B***Rocinelabelliceps* (after Brusca and France 1992) **C***Rocinelalaticauda* (after Brusca and France 1992) **D***Rocinelamurilloi* (after Brusca and Iverson 1985; Brusca and France 1992) **E***Rocinelasignata* (after Brusca and Iverson 1985; Brusca and France 1992) **A1–E1** = pereopods 3 **A2–E2** = uropods with medial process of peduncle indicated by arrows.

**Figure 10.**
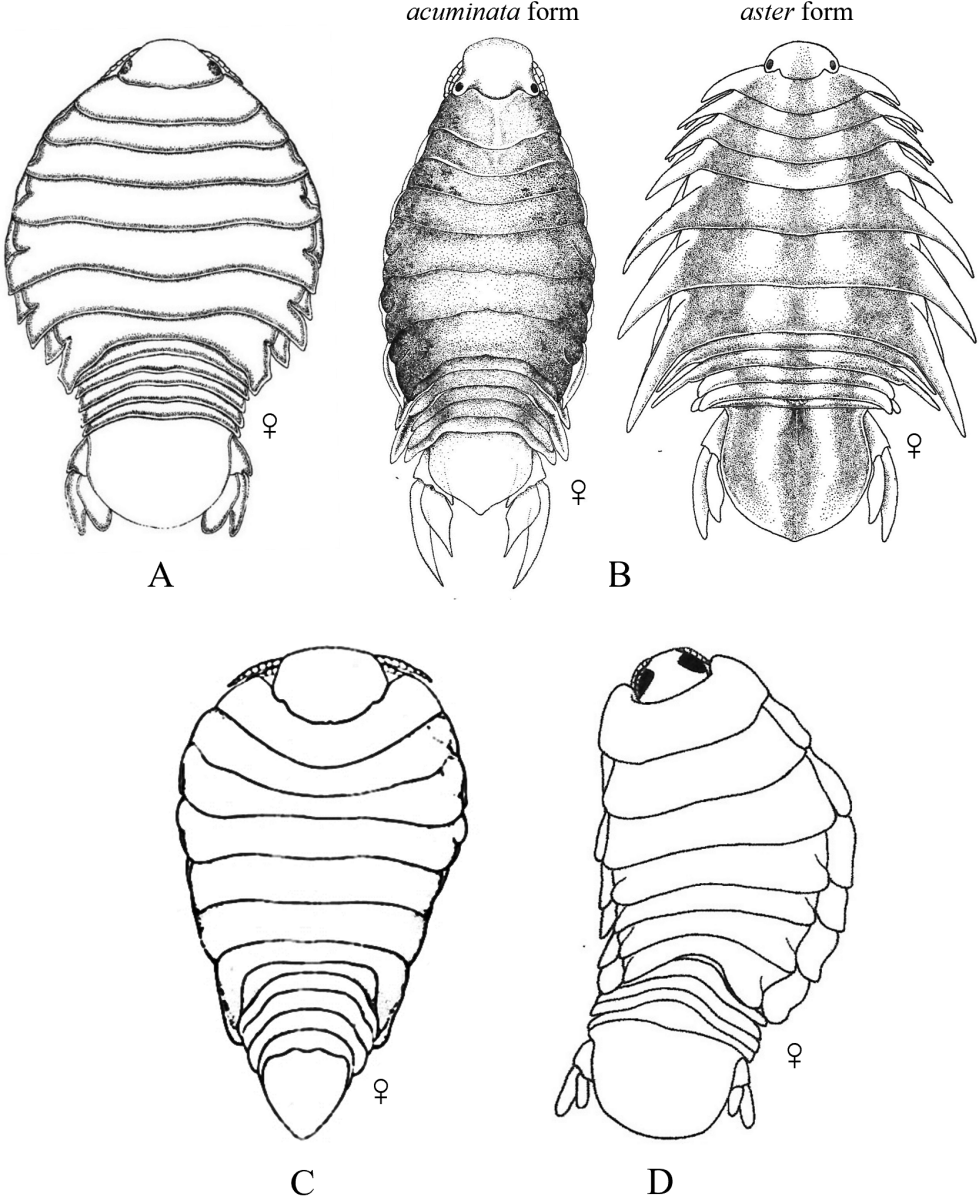
Isopoda, Cymothoida, Cymothooidea, Cymothoidae (in part): **A***Renocilathresherorum* (after Brusca 1981) **B***Nerocilaacuminata* (after Brusca and Iverson 1985) **C***Smenispaconvexa* (after Brusca 1981) **D***Mothocyarosea* (after Brusca et al. 2007).

**Figure 11.**
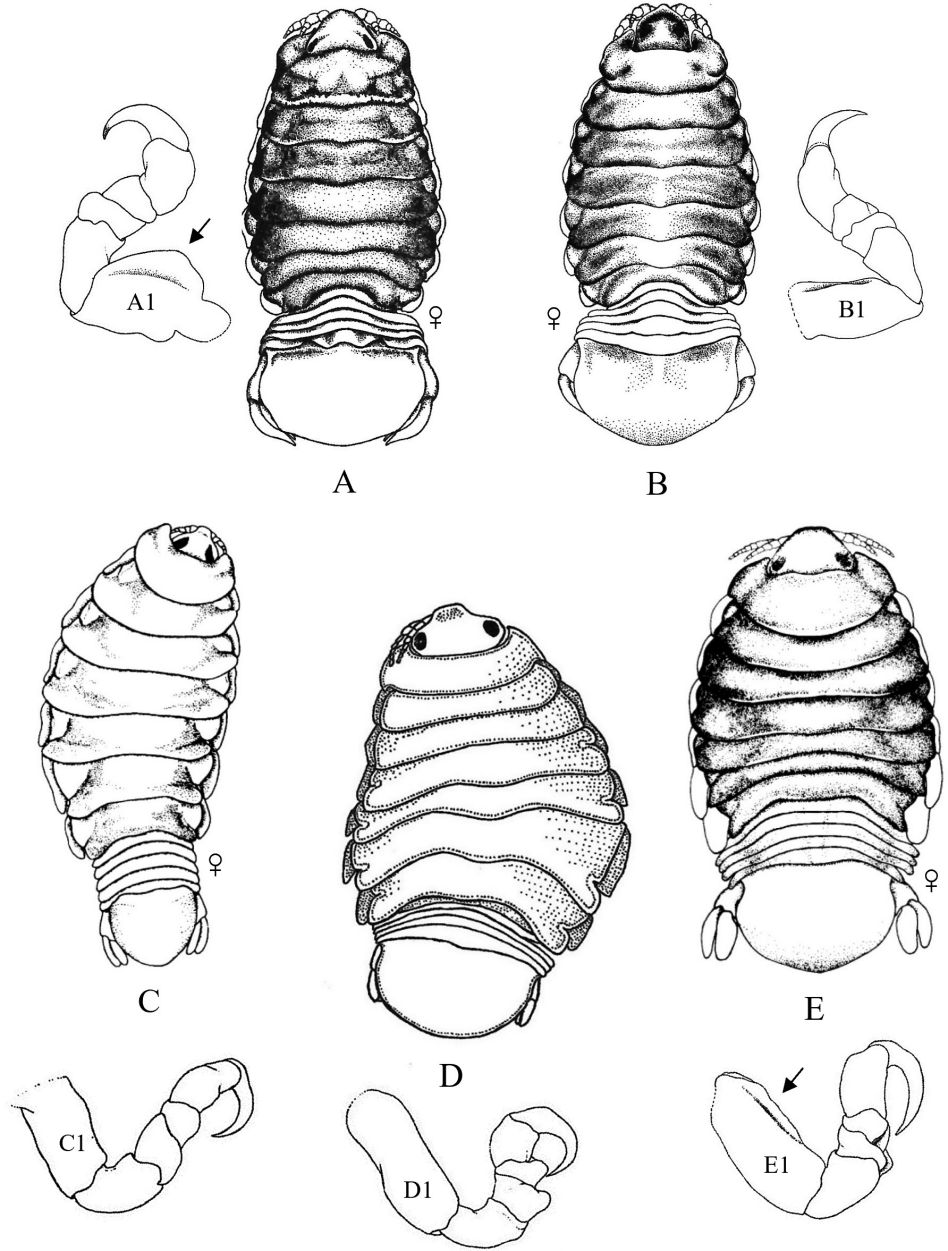
Isopoda, Cymothoida, Cymothooidea, Cymothoidae (in part): **A***Ceratothoagaudichaudii***A1** pereopod 4 with arrow indicating carina (after Brusca 1981) **B***Ceratothoagilberti***B1** pereopod 4 (after Brusca 1981) **C***Elthusacalifornica***C1** pereopod 4 (after Brusca 1981) **D***Elthusamenziesi***D1** pereopod 4 (after Menzies 1962a; Brusca 1981) **E***Elthusavulgaris***E1** pereopod 4 with arrow indicating carina (after Brusca 1981).

**Table:** 

1	Pereopods 1–3 ambulatory with dactylus shorter than propodus (e.g., Fig. 3C)	**2** [Cirolanidae]
–	Pereopods 1–3 prehensile or sub-prehensile with dactylus generally as long as, or longer than propodus and strongly curved (e.g., Fig. 3D)	**8**
2	Eyes absent; head immersed in pereonite 1 with posterior margin appearing deeply concave; pereon with coxae 4–7 produced beyond posterior margins of their respective pereonites, at least 2 or more visible in dorsal view; lateral margins of pleonite 5 obscured by pleonite 4 (Fig. 7G)	***Natatolanacaliforniensis***
–	Eyes present; posterior margin of head not appearing distinctly concave; with or without dorsally visible coxae on pereonites; lateral margins of pleonite 5 may or may not be obscured by pleonite 4	**3**
3	Coxae of pereonites 2–7 well-developed, typically visible in dorsal view and expanded laterally with acute posterior angles; epimeres of pleonites 2–5 well-developed, expanded laterally, with acute posterior angles; pleotelson with squarish to pointed posterior margin and a strong, middorsal longitudinal ridge; margins of pleotelson and uropodal rami notched (Fig. 7F)	***Metacirolanajoanneae***
–	Pereonites, pleonites, pleotelson and uropods not as above	**4**
4	Head with prominent spatulate rostral process separating left and right antennules (Fig. 7D, E)	**5**
–	Head without a prominent rostral process between antennules	**6**
5	Posterior margin of pleotelson broadly rounded and crenulate; antennular peduncle articles 2 and 3 subequal in length (Fig. 7E)	***Excirolanalinguifrons***
–	Posterior margin of pleotelson obtusely rounded and acuminate; antennular peduncle article 3 longer than article 2 (Fig. 7D)	***Excirolanachiltoni***
6	Antennules geniculated, with peduncle article 1 longer than articles 2 or 3, and article 2 arising at right angles to article 1; peduncle of antennae with 4 articles, antennae long and extending beyond pereonite 7; lateral margins of pleonite 5 not obscured by pleonite 4; uropodal rami truncate distally, exopod does not extend to posterior margin of pleotelson (Fig. 7C)	***Eurydicecaudata***
–	Antennules not geniculated; peduncle of antennae with 5 articles; lateral margins of pleonite 5 obscured by pleonite 4; uropodal rami distally rounded or acuminate, extending beyond posterior of pleotelson	**7**
7	Uropodal rami with apical notches and not distally rounded; peduncle articles 1 and 2 of antennules fused; coxae of pereonites 5–7 visible dorsally; pereonites, pleonites and pleotelson without dorsal tubercles, carina, or setae (Fig. 7A)	***Cirolanadiminuta***
–	Uropodal rami rounded distally, without notches; peduncle articles 1 and 2 of antennules not fused; coxae visible dorsally on pereonites 2–7; pleonites 3–5 with row of small tubercles on posterior margins; pleotelson of adult males with two large dorsal submedian tubercles or carinae (Fig. 7B)	***Cirolanaharfordi***
8	Pereopods 4–7 ambulatory (dactylus shorter than propodus)	**9**
–	Pereopods 4–7 prehensile (dactylus generally as long as, or longer than propodus and strongly curved); adults parasitic on fishes	**19** [Cymothoidae]
9	Dorsal surface of pleon tuberculate, with small to medium tubercles present on posterior margins of at least pleonites 3–5	**10**
–	Dorsal surface of pleon without tubercles	**13** [Aegidae]
10	Pleotelson dorsally setose, lateral margins with single incision	**11** [Corallanidae]
–	Pleotelson not dorsally setose, lateral margins without incisions	**12** [Tridentellidae]
11	Male head with 3 large horns or tubercles, including 1 rostral and 2 posterolateral between the eyes (female without tubercles); pereonites 2–7 without dorsal setae or tubercles; pleotelson subtriangular with rounded apex, dorsal surface setose except for median longitudinal area (Fig. 8A)	***Excorallanatricornisoccidentalis***
–	Head of both males and females without horns or tubercles; pereonites 4–7 with dorsal setae and row of small tubercles on posterior margin; pleotelson triangular with subacute apex, entire dorsal surface densely covered with bifid golden setae (Fig. 8B)	***Excorallanatruncata***
12	Body dorsal surface sculptured with low or small tubercles; head of male with 5 low tubercles, including 1 rostral, 1 pair near anterior margin, and 1 pair near posterior margin; male pereonite 1 with 2 small, median tubercles near anterior margin; female lacking tubercles on head and pereon; pleonites 3–5 with small tubercles on posterior margins; pleotelson minutely tuberculate dorsally with widely rounded, slightly crenulate posterior margin (Fig. 8D)	***Tridentellaquinicornis***
–	Body dorsal surface sculptured with large processes and numerous tubercles; male head with 2 dorsal posterolateral horns, frontal margin produced into large, upturned process and smaller ventrally projecting rostrum; pereonite 1 with 3 large dorsal processes; all pereonites and pleonites with numerous dorsal tubercles that increase in size and become more spine-like posteriorly; pleotelson triangular with subtruncate apex, dorsally covered with longitudinal rows of large, spine-like tubercles; females much less spinose than males, lacking large processes on head and pereonite 1 (Fig. 8C)	***Tridentellaglutacantha***
13	Peduncular articles 1 and 2 of antennules greatly expanded (dilated), article 2 with gradual distal process extending 25–50% the distance into article 3; posterior margin of pleotelson truncate, crenulated and fringed with setae (Fig. 8E)	***Aegalecontii***
–	Peduncular articles of antennules not dilated, article 2 without distal process; posterior margin of pleotelson rounded or subacuminate	**14**
14	Eyes large, close-set, nearly touching at midline; pleotelson shield-shaped with subacuminate apex and weekly serrated (notched) posterolateral margins; uropodal rami ovate with subacuminate apices (Fig. 8F)^Endnote 3^	***Aegiochusplebeia***
–	Eyes medium to large, but distinctly separated and not nearly touching medially; posterior margin of pleotelson rounded; uropodal rami with broadly rounded to truncate apices	**15**
15	Medial process of uropodal peduncle very long, extending at least 75% of length of endopod	**16**
–	Medial process of uropodal peduncle extends 50% or less of length of endopod	**17**
16	Propodi of pereopods 1–3 with large, broad, spine-bearing medial lobe; dactyli of pereopods 1–3 longer than propodi; frontal lamina broadly expanded anteriorly, arrowhead or spatulate shaped (Fig. 9B)	***Rocinelabelliceps***
–	Propodi of pereopods 1–3 without expanded medial lobe; dactyli of pereopods 1–3 subequal in length to propodi; frontal lamina thin and narrow (Fig. 9E)	***Rocinelasignata***
17	Medial process of uropodal peduncle extends < 40% of length of endopod; propodi of pereopods 1–3 with 4 stout, recurved acute spines; merus of pereopods 1–3 with 5–8 acute spines (3–5 distal, 2 or 3 proximal) (Fig. 9A)	***Rocinelaangustata***
–	Medial process of uropodal peduncle extends ~ 50% of length of endopod; propodi of pereopods 1–3 with 4–6 acute spines; merus of pereopods 1–3 with 4 acute spines (3 distal, 1 proximal)	**18**
18	Propodi of pereopods 1–3 with 5 thin, straight acute spines; apical article of maxillipedal palp with thin, nearly straight, acute spines (Fig. 9C)	***Rocinelalaticauda***
–	Propodi of pereopods 1–3 with 4–6 stout and recurved acute spines; apical article of maxillipedal palp with stout, recurved acute spines (Fig. 9D)	***Rocinelamurilloi***
19	Pleopods and uropods not setose	**20**
–	Pleopods and uropods heavily setose, adapted for swimming (juvenile cymothoids)^Endnote 4^	**unidentified Cymothoidae**
20	Body very broad and darkly pigmented; pereon at least 2× as wide as pleon with strongly convex lateral margins (widest at pereonite 5); parasite of barspot cardinalfish and Panamic fanged blenny in Eastern Pacific (Fig. 10A)^Endnote 5^	***Renocilathresherorum***
–	Body not as above	**21**
21	Posterior margin of head weakly to strongly trisinuate; pleon not immersed in pereon	**22**
–	Posterior margin of head not trisinuate; pleon partially immersed in pereon	**23**
22	Head not immersed in pereonite 1, posterior border distinctly trisinuate; coxal margins of all or just posterior pereonites with acute or subacute posterolateral angles, coxae may be held close to body (*acuminata* form) or greatly expanded laterally (*aster* form); uropods visible dorsally, extending clearly beyond posterior border of pleotelson; parasite of ~ 40 different species of fishes (Fig. 10B)	***Nerocilaacuminata***
–	Head somewhat immersed in pereonite 1, subquadrate anteriorly with weakly trisinuate posterior border; uropods not visible in dorsal view, typically held concealed under pleotelson and not extending beyond posterior border; parasite of Pacific bumper, pompanos, serranos, carangids, and other fishes (Fig. 10C)	***Smenispaconvexa***
23	Basal articles of antennules expanded and touching or nearly touching	**24**
–	Basal articles of antennules not expanded and touching	**25**
24	Pereopods 4–7 carinate; posterior margin of pleonite 5 trisinuate except in occasional males; parasite of pelagic fishes, including striped mullet off southern California and pompanos and herring off Baja California (Fig. 11A)^Endnote 6^	***Ceratothoagaudichaudii***
–	Pereopods 4–7 not carinate; posterior margin of pleonite 5 smooth, not trisinuate; parasite of mullets and flatfish (Fig. 11B)	***Ceratothoagilberti***
25	Antennules longer than antennae; parasite of California and skipper halfbeaks (Fig. 10D)	***Mothocyarosea***
–	Antennules shorter than antennae	**26**
26	Frontal margin of head broadly rounded or truncate (not produced); bases of pereopods 4–7 with distinct carinae; coxae of pereonites 6 and 7 extending to and usually beyond posterior edge of respective pereonites; pleotelson in adult females nearly 2× as wide as long; parasite of at least 30 species of fishes (Fig. 11E)	***Elthusavulgaris***
–	Frontal margin of head produced; bases of posterior pereopods of females without distinct carinae; coxae of pereonites 6 and 7 not reaching posterior margins of respective pereonites; pleotelson in adult females either as wide as or wider than long	**27**
27	Merus and carpus of pereopod 4 expanded; bases of pleopods with well-developed accessory lamellae; pleotelson in adult females broadly rounded, ~ 1.5–2.0 × wider than long; males with coxal carinae on pereopods 4–7; parasite of wooly sculpin, northern clingfish, and reef finspot (Fig. 11D)	***Elthusamenziesi***
–	Merus and carpus of pereopod 4 not expanded; accessory lamellae of pleopodal bases not well developed; pleotelson in adult females ca. as wide as long; males without carinae on posterior pereopods; parasite of surfperch, smelt, gobies, killifish, and grunion (Fig. 11C)	***Elthusacalifornica***

### ﻿Key D. Suborder Cymothoida, Superfamily Cymothooidea (in part): Family Gnathiidae

Fig. 12

**Figure 12.**
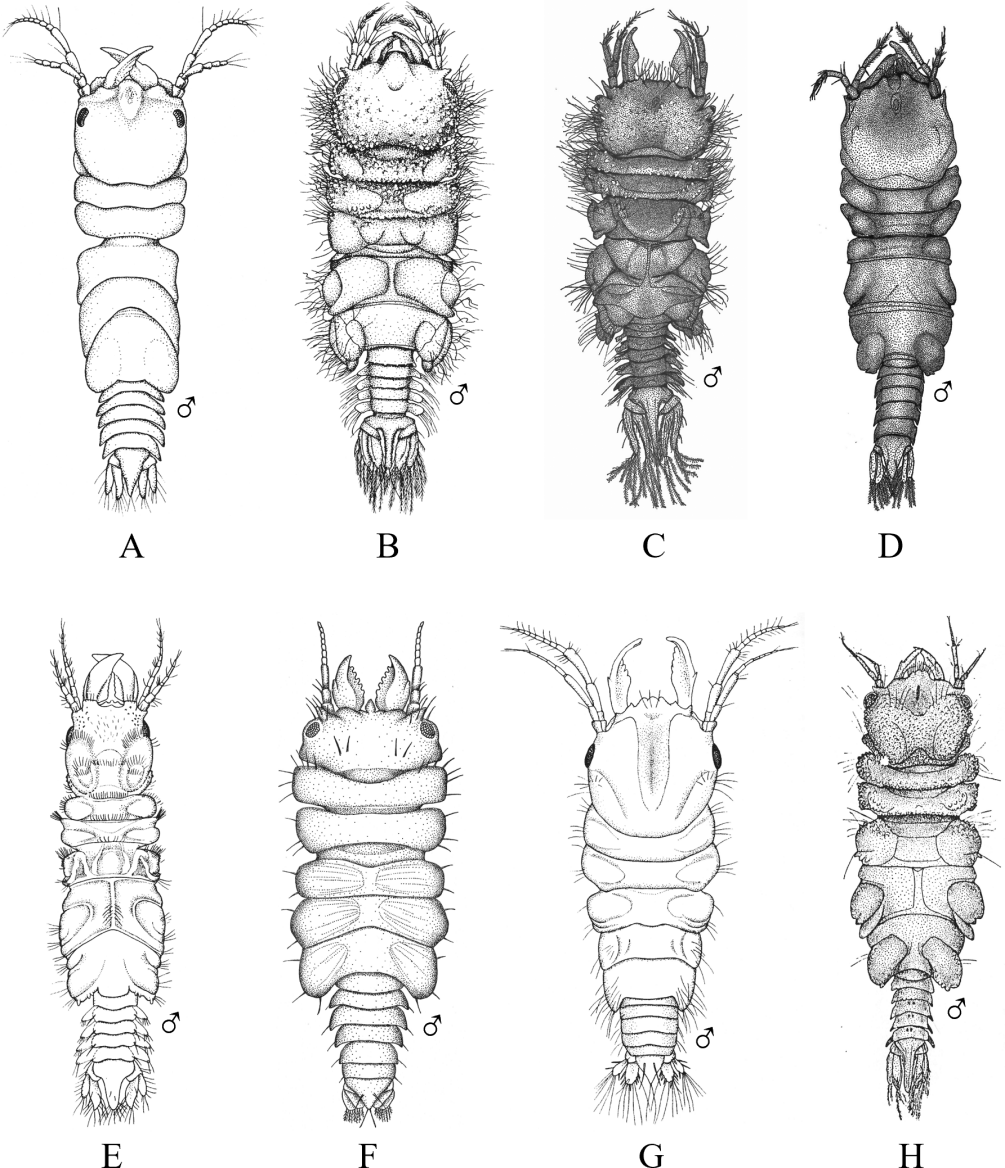
Isopoda, Cymothoida, Cymothooidea, Gnathiidae: **A***Caecognathiacrenulatifrons* (after Wetzer and Brusca 1997) **B***Caecognathiasanctaecrucis* (after Wetzer and Brusca 1997) **C***Gnathiaclementensis* (after Schultz 1966) **D***Gnathiacoronadoensis* (after Schultz 1966) **E***Gnathiaproductatridens* (after Wetzer and Brusca 1997) **F***Gnathiasteveni* (after Menzies 1962a) **G***Gnathiatridens* (after Wetzer and Brusca 1997) **H***Gnathiatrilobata* (after Schultz 1966).

**Table:** 

1	Head with enlarged forceps-like mandibles projecting anteriorly (male gnathiids)	**2**
–	Head not as above, without projecting mandibles; body often sac-like (female and juvenile gnathiids)^Endnote 7^	**Gnathiidae spp.**
2	Eyes present, with or without pigment; telson variable in shape	**3**
–	Eyes absent; frontal margin of head (frons) with 3 central processes, laterals larger than middle process; epimeres single, dorsal, laterally projected; telson distinctly triangular (Fig. 12D)	***Gnathiacoronadoensis***
3	Pleotelson distinctly triangular	**4**
–	Pleotelson arrowhead or T-shaped with base expanded	**8**
4	Pleonal epimeres laterally expanded, highly visible; body with few to numerous setae	**5**
–	Pleonal epimeres not laterally expanded, barely visible; body with relatively few setae	**7**
5	Frontal margin of head forming broad, transverse, minutely crenulated plate (best viewed ventrally); eyes brown to reddish brown; body setosity light, without numerous setae; pleopods without setae (Fig. 12A)	***Caecognathiacrenulatifrons***
–	Frontal margin of head not transverse (lobes or processes present); eyes reddish brown; body not hirsute, but with numerous setae; pleopods with setae	**6**
6	Body mottled with brown pigment; frontal margin of head forming centrally extended narrow lobe with crenulations; mandibles split into 2 articles^Endnote 8^	***Caecognathia* sp. A**
–	Body without pigment; frontal margin of head trilobed with 3 central subequal processes; mandibles of single article only (not split into 2 articles) (Fig. 12G)	***Gnathiatridens***
7	Eyes dark brown, body mottled with brown pigment; frontal margin of head with 3 processes, median process largest and shaped as stepwise pyramid; head with setae and tuberculations (Fig. 12F)	***Gnathiasteveni***
–	Eyes reddish brown; body without pigmentation; frontal margin of head with central 3-dimensional expansion in shape of box, with 2 large setae extending outward centrally; head with setae, but lacking tuberculations^Endnote 8^	***Gnathia* sp. MBC1**
8	Pleotelson distinctly T-shaped	**9**
–	Pleotelson arrowhead-shaped	**10**
9	Eyes sessile, dark brown, lens with tuberculations; body speckled with tiny black dots; frontal margin of head produced into single large lobe; pleopods ovate, paddle-like; body hirsute (Fig. 12B)	***Caecognathiasanctaecrucis***
–	Eyes on distinct ocular peduncles, unpigmented, lens without tuberculations; frontal margin of head with 2 medium–large lateral processes and 4 central subequal processes; pleopods long and narrow; body hirsute (Fig. 12C)	***Gnathiaclementensis***
10	Pleonal epimeres double (dorsal and ventral); eyes golden/amber in color; frontal margin of head with 3 central, subequal processes; body with numerous scattered setae, but not hirsute (Fig. 12H)	***Gnathiatrilobata***
–	Pleonal epimeres single (dorsal only)	**11**
11	Eyes golden or amber in color; head with dorsal carina present; frontal margin of head as 1 broad truncate lobe with medial carina; body hirsute^Endnote 8^	***Caecognathia* sp. SD1**
–	Eyes with red and white checkerboard pattern; head without dorsal carina; frontal margin of head with 3 central subequal processes; body not hirsute, but with numerous setae (Fig. 12E)	***Gnathiaproductatridens***

### ﻿Key E. Suborder Cymothoida, Superfamilies Bopyroidea and Cryptoniscoidea

Figs 13–16

**Figure 13.**
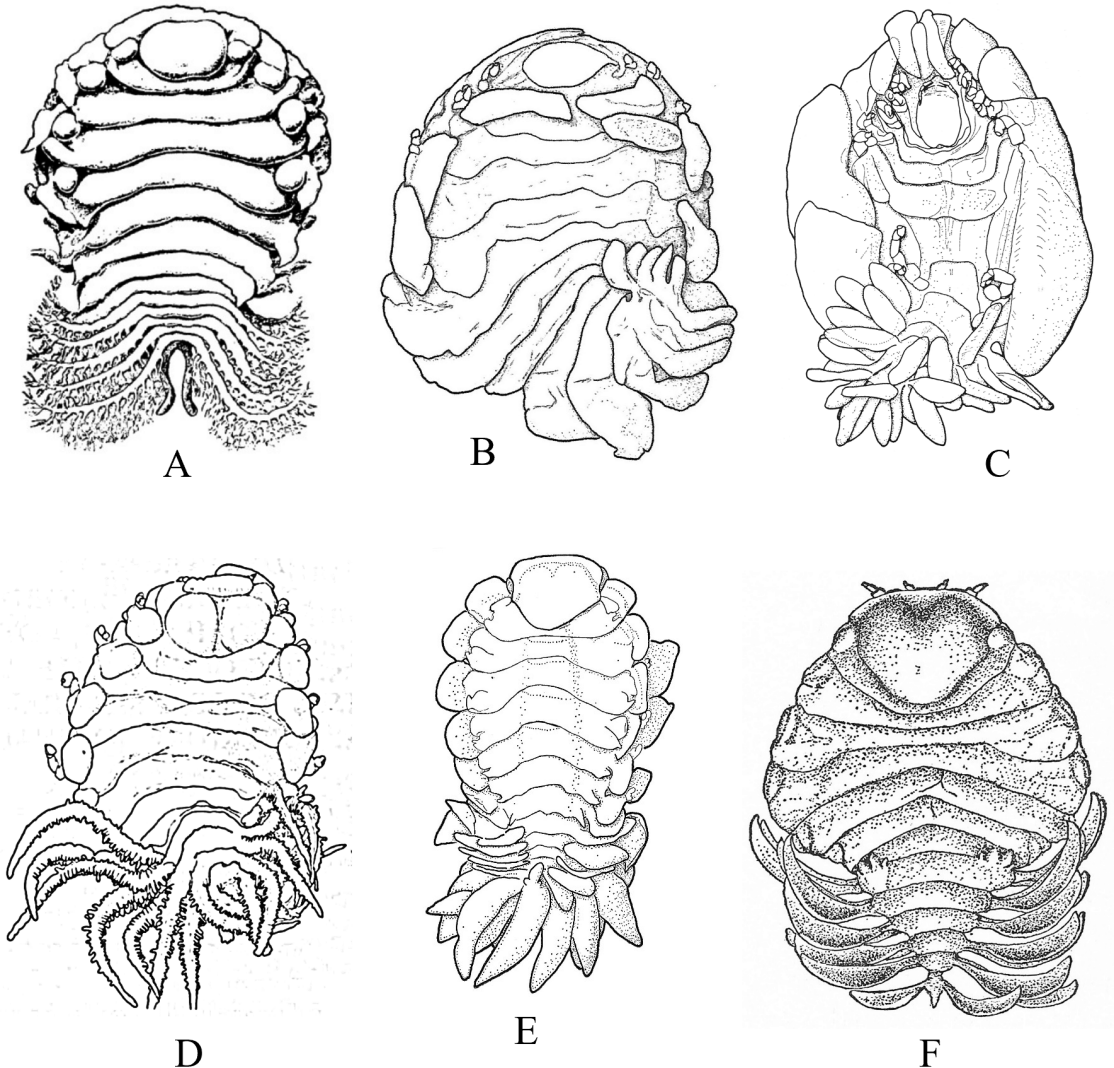
Isopoda, Cymothoida, Epicaridea, Bopyroidea, Ionidae: **A***Ionecornuta* (after Richardson 1905a). Bopyridae (in part): **B***Bathygygegrandis* (after Markham 2016) **C***Anathelgeshyphalus* (after Markham 1974a) **D***Leidyainfelix* (after Markham 2002) **E***Munidionpleuroncodis* (after Markham 1975); **F***Phyllodurusabdominalis* (after Richardson 1905a).

**Figure 14.**
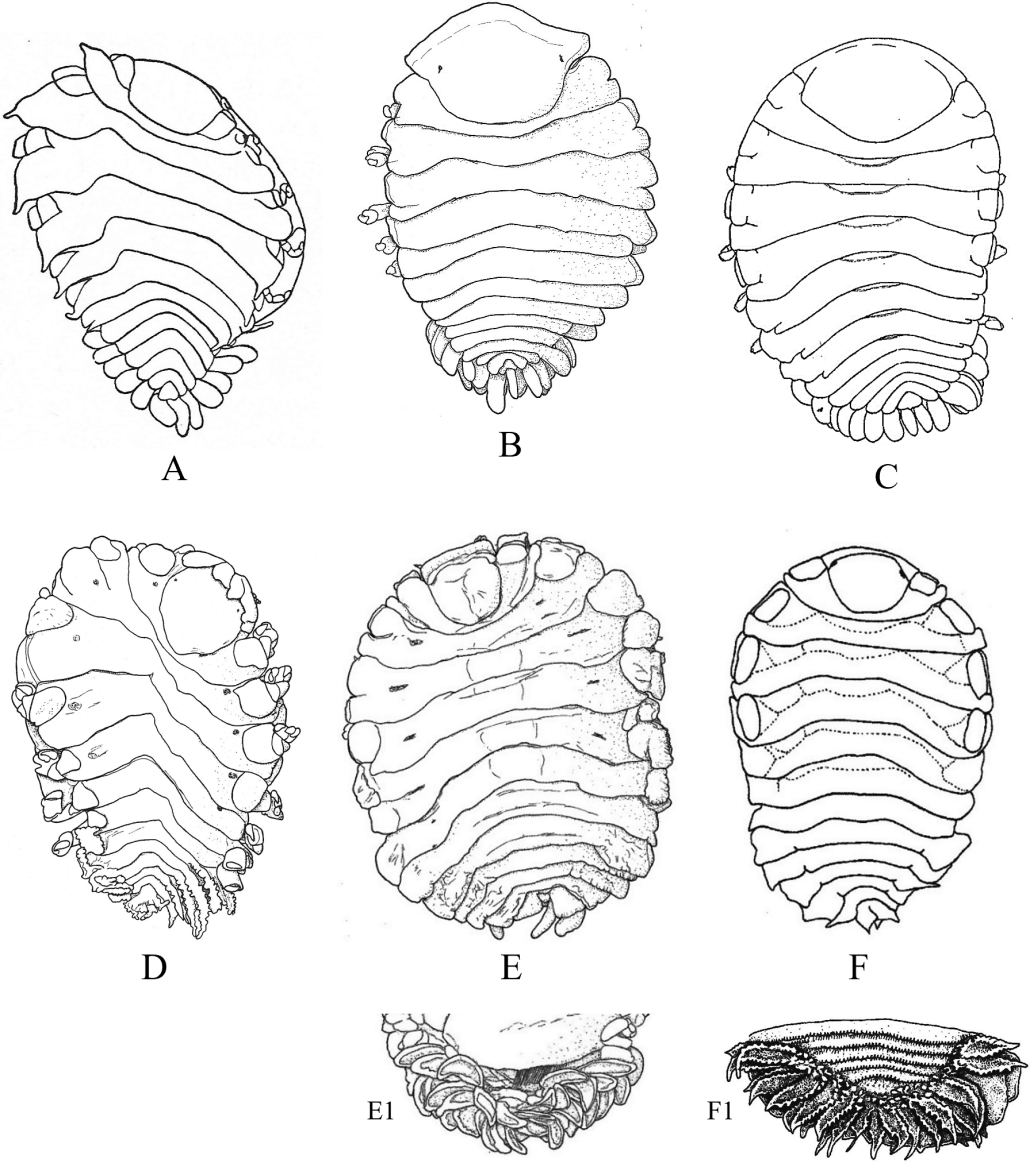
Isopoda, Cymothoida, Epicaridea, Bopyroidea, Bopyridae (in part): **A***Argeiapugettensis* (after Richardson 1905a) **B***Aporobopyrusmuguensis* (after Markham 2008) **C***Aporobopyrusoviformis* (after Shiino 1934) **D***Asymmetrioneambodistorta* (after Markham 1985b) **E***Orthionegriffenis***E1** ventral view of pleon and pleopods (after Markham 2004) **F***Progebiophilusbruscai***F1** ventral view of pleon and pleopods (after Campos and de Campos 1998).

**Figure 15.**
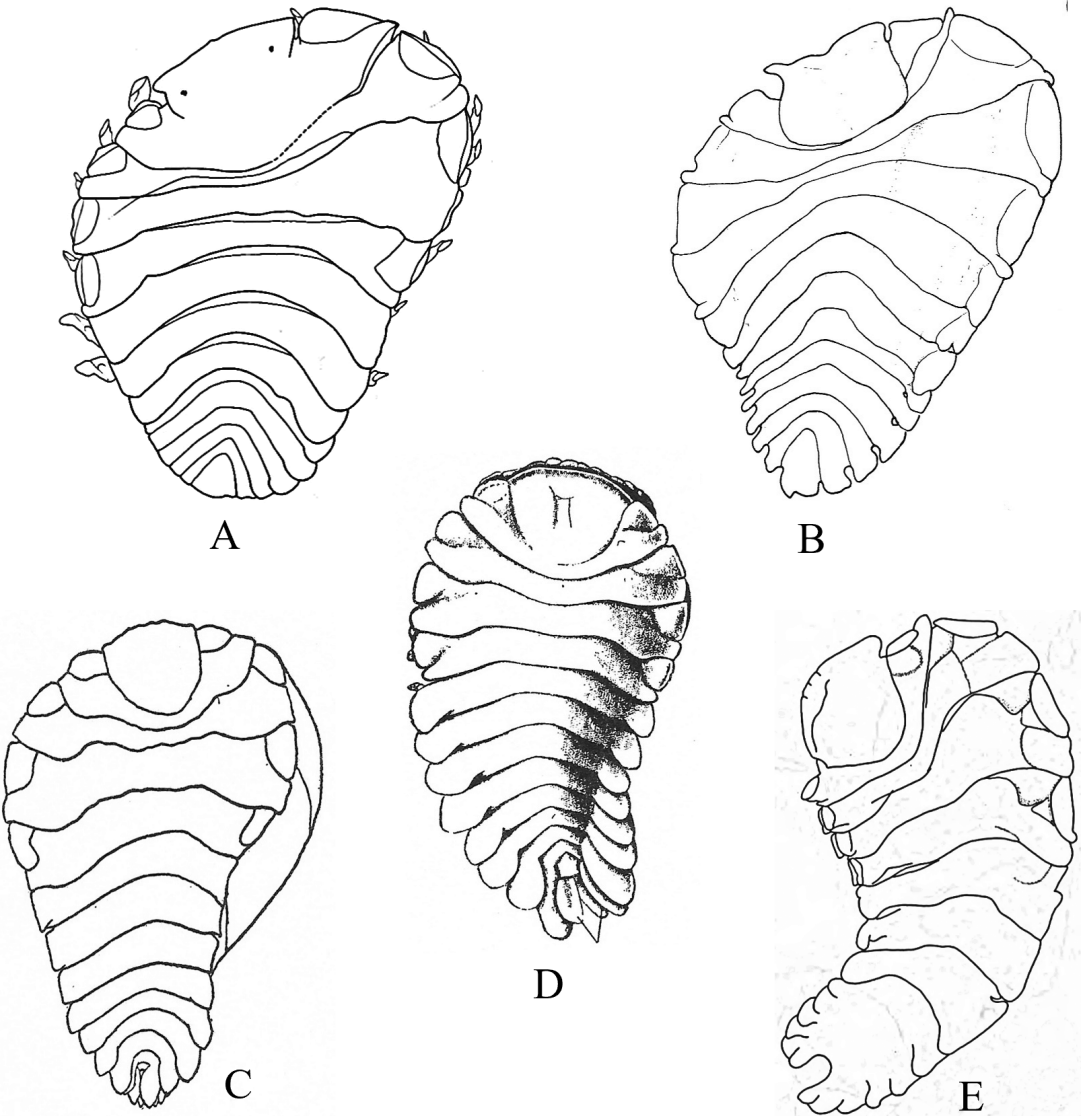
Isopoda, Cymothoida, Epicaridea, Bopyroidea, Bopyridae (in part): **A***Bopyrellacalmani* (after Sassaman et al. 1984) **B***Capitetragoniaalphei* (Caribbean species = representative for *Capitetragonia* sp. A; after Markham 1985a) **C***Eremitionegiardi* (after Richardson 1905a) **D***Pseudionegalacanthae* (after Richardson 1905a) **E***Schizobopyrinastriata* (after Campos and de Campos 1990).

**Figure 16.**
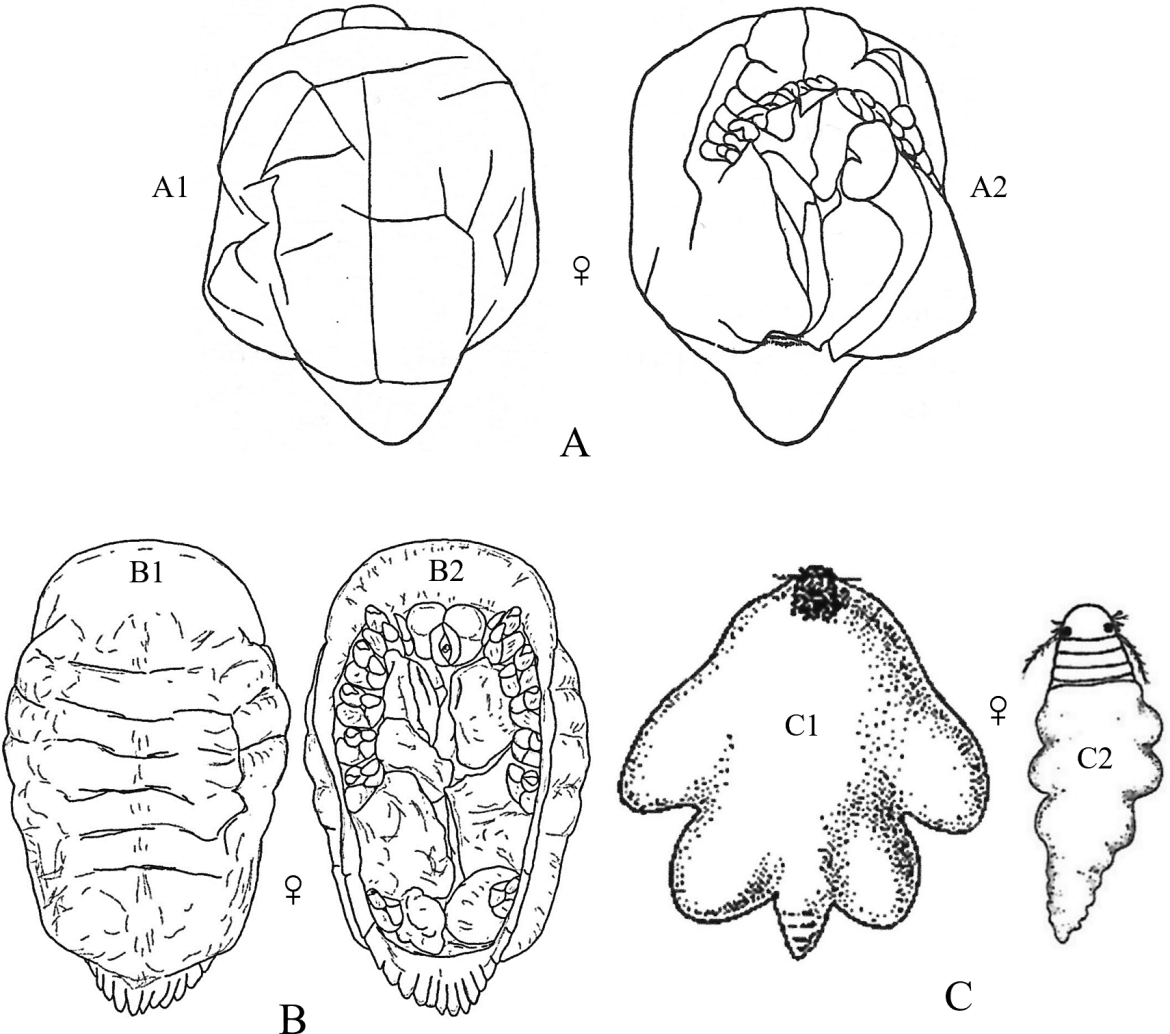
Isopoda, Cymothoida, Epicaridea, Cryptoniscoidea, Dajidae: **A***Holophryxusalaskensis* (adult female) **A1** dorsal view **A2** ventral view (after Richardson 1905b) **B***Zonophryxusprobisowa* (adult female) **B1** dorsal view **B2** ventral view (Peruvian species = representative for unidentified SCB species of *Zonophryxus*; after Boyko and Williams 2021a). Hemioniscidae: **C***Hemioniscusbalani***C1** adult female **C2** juvenile female (after Nierstrasz and Brender à Brandis 1926; Williams and Boyko 2012).

**Table:** 

1	Body of adult female distinctly segmented with clear division of head (cephalon), thoracic (pereon), and abdominal (pleon) regions	**2** [Bopyridae and Ionidae]
–	Body of adult female sac-like, with at most weak segmentation visible	**18** [Dajidae and Hemioniscidae]
2	Adult female body broadly oval; pleon strongly torsioned and reflexed forward over pereon; head without eyes, deeply embedded in pereon, anterior margin covered by oostegites; pleomeres 3–6 fused medially; branchial parasite of deep-water crangonid shrimps of genus *Glyphocrangon* (Fig. 13B)	***Bathygygegrandis***
–	Female body not as above, pleon not strongly reflexed over pereon	**3**
3	Female pleon with pleopods and/or elongated lateral plates (epimeres or pleural lamellae) clearly noticeable in dorsal view	**4**
–	Female pleon without pleopods or elongated lateral plates noticeable in dorsal view	**12**
4	Lateral plates of pleon and/or pleopods conspicuously elongate, foliaceous, or lanceolate, with or without digitated or crenulated margins	**5**
–	Lateral plates of pleon and pleopods relatively short, distal margins of pleopods mostly rounded	**10**
5	Adult female with elongated lateral plates fringed with long, branched processes; branchial parasite of ghost shrimps of genus *Neotrypaea* (Fig. 13A)	***Ionecornuta***
–	Adult female with or without lateral plates, but lateral plates not as above if present	**6**
6	Adult female body nearly oval, with oostegites conspicuously visible dorsally arching over margins of head and pereon; head longer than wide, deeply immersed in pereon, eyes absent; pereomere 6 much longer than other pereo­meres, pereomeres 1–5 concave anteriorly, pereomeres 6 and 7 concave posteriorly; pleon with “triramous appendages” in appearance, but each one consists of a foliaceous lanceolate lateral plate and identical biramous pleopod; branchial parasite of hermit crabs *Parapagurodeslaurentae* and *P.makarovi* (Fig. 13C)	***Anathelgeshyphalus***
–	Adult female body not as above, pereomere 6 subequal in length to other pereomeres; pleon with lateral plates and pleopods not appearing like triramous appendages	**7**
7	Adult females with pair of dorsolateral papillae on pleomere 1; pleon with long, narrow biramous pleopods arising from thin peduncle or stem on each segment, without lateral plates; head slightly wider than long, bilobate, eyes absent; abdominal parasite of mud shrimps of genus *Upogebia* (Fig. 13F)	***Phyllodurusabdominalis***
–	Adult females without dorsolateral papillae on pleomere 1; pleomeres with both elongated lateral plates and pleopods, but morphology of pleopods not as above	**8**
8	Pleon of adult females enclosed by tentacular-like, elongated lanceolate lateral plates and pleopodal exopods, each with deeply digitate margins, ventral surface covered partially by smaller foliate pleopodal endopods with crenulated margins; head completely embedded in pereon, dorsal surface divided into two large sub-oval lobes, eyes absent; branchial parasite of grapsid crab *Pachygrapsuscrassipes* (Fig. 13D)	***Leidyainfelix***
–	Pleon of adult females with lateral plates and pleopods not as above; head not bilobate	**9**
9	Adult female body ~ 2 × longer than wide, pyriform in shape; head slightly wider than long, eyes absent; coxal plates very large, overlapping, and extending well beyond lateral margins of pereomeres; pleon obscured by prominent ovate lateral plates and long, biramous lanceolate pleopods; branchial parasite of pelagic galatheid “red crab” *Pleuroncodesplanipes* (Fig. 13E)	***Munidionpleuroncodis***
–	Adult female body slightly longer than wide; head subcircular, eyes absent; coxal plates prominent but not widely extended on pereomeres 1–4; lateral edges on convex side of pereomeres 5–7 produced into slender points reflexed back over dorsum; pleomeres 1–5 with dentate-margined lanceolate lateral plates and similar biramous pleopods; branchial parasite of hermit crab *Isochelespilosus* (Fig. 14D)	***Asymmetrioneambodistorta***
10	Adult female head somewhat bilobate, eyes absent; pereomeres 1–6 with sharply pointed tergal projections on longer side of body; pleomeres 1–5 with short lateral plates and distally rounded uniramous pleopods; branchial parasite of several genera of crangonid and hippolytid shrimps (e.g., *Crangon*, *Eualus*) (Fig. 14A)	***Argeiapugettensis***
–	Adult females not as above; head not bilobate, with or without eyes; pereon without sharp tergal projections; pleopods biramous	**11**
11	Adult female head wider than long with rounded anterior and posterior margins, eyes absent; narrow and rudimentary coxal plates on pereomeres 3 and 4 on both sides of body; branchial parasite of porcelain crab *Pachychelespubescens* (Fig. 14C)	***Aporobopyrusoviformis***
–	Adult female head subtriangular in shape, anterolateral margins produced into small obtuse projections, eyes present; rudimentary coxal plates of pereomeres 3 and 4 only on longer side of body; branchial parasite of porcelain crabs *Pachycheles* spp. (Fig. 14B)	***Aporobopyrusmuguensis***
12	Adult female body oval to broadly oval, pereon and pleon subequal in width; branchial parasite of mud shrimps of genus *Upogebia*	**13**
–	Adult female body not oval, pleon tapering to much narrower than widest pereomeres	**14**
13	Adult female body broadly oval, almost as wide as long (L:W ratio ~ 1.2); head almost square, deeply embedded in pereomere 1, eyes absent; ventral surface of pleomeres covered by overlapping lanceolate lateral plates and uniramous uropods, middle region covered by similar sized biramous pleopods (Fig. 14E)	***Orthionegriffenis***
–	Adult female body oval, distinctly longer than wide (L:W ratio ~ 1.5); head slightly wider anteriorly than posteriorly, deeply embedded in pereomere 1, eyes present; ventral surface of pleomeres covered by numerous ridges and lanceolate biramous, marginally tuberculate pleopods (Fig. 14F)	***Progebiophilusbruscai***
14	Pleon of adult females composed of 5 medially fused pleomeres and pleotelson, the latter deeply arcuate and embedded in pleomere 5; head roughly triangular, separated from pereon by deep groove, eyes absent; branchial parasite of hippolytid shrimps *Hippolytecaliforniensis* and *Thoralgicola* (Fig. 15E)	***Schizobopyrinastriata***
–	Adult female body not as above; pleomeres distinctly separate, not medially fused; head with or without eyes	**15**
15	Head of adult female partially fused with pereomere 1 and separated by only short lateral notches; frontal margin of head slightly sinuated with anterolateral process usually on just short side of body, small eyes present; pleotelson entirely set within curves of pleomere 5; branchial parasite of snapping shrimps *Alpheopsisequidactylus* and *Synalpheuslockingtoni* (Fig. 15A)	***Bopyrellacalmani***
–	Female head distinctly separate and not fused with pereomere 1, eyes absent; pleotelson not completely set within curves of pleomere 5	**16**
16	Head of adult female squarish with distinct anterolateral horns and a small anteromedial indentation; pleon with 6 distinct pleomeres separated by deep lateral notches; pleopods 1–4 biramous (5^th^ pair absent), uropods absent; branchial parasite of alpheid shrimp *Automate* sp. A (Fig. 15B)^Endnote 9^	***Capitetragonia* sp. A**
–	Female head wider anteriorly than posteriorly, without anterolateral horns; pleon with 5 pleomeres not separated by deep notches and very small 6^th^ segment, 5 pairs of biramous pleopods, and terminal pair of lanceolate uropods	**17**
17	Endopods of pleopods 1–5 much larger than exopods, elongate and pointed, surface rough with irregular rugae; coxal plates of pereomeres 5–7 not developed as lamellae; branchial parasite of hermit crabs of genus *Pagurus* (Fig. 15C)^Endnote 10^	***Eremitionegiardi***
–	Endopods of pleopods 1–5 only slightly larger than exopods, triangular or ovate, surface smooth; coxal plates of pereomeres 5–7 developed as lamellae; branchial parasite of squat lobsters *Galacanthadiomedeae* and *Munidaquadrispina* in the East Pacific (Fig. 15D)	***Pseudionegalacanthae***
18	Adult female body simply an egg sac, without evidence of segmentation; antennae and mouthparts absent; parasite on barnacles of genera *Balanus* and *Chthamalus* (Fig. 16C)	***Hemioniscusbalani*** [Hemioniscidae]
–	Adult female body with weak evidence of segmentation visible dorsally or laterally; antennae and mouthparts present; parasitic on caridean shrimp of families Pasiphaeidae and Pandalidae	**19** [Dajidae]
19	Adult female body typically elongate and symmetrical, but may be irregular with deeper, stouter body in some specimens; head separate, typically hemispherical, visible dorsally or ventrally; segmentation of pereon usually visible laterally by 4 coxal plates; pleonal region posteriorly projected as unsegmented conical prominence; parasite on carapace of pasiphaeid shrimp *Pasiphaeapacifica* (Fig. 16A)^Endnote 11^	***Holophryxusalaskensis***
–	Adult female body ovate, all regions fused and indistinct dorsally; posterior margin of pleon appears notched with row of triangularly shaped processes; parasite on carapace of pandalid shrimps *Pantomusaffinis* and *Plesionikatrispinus* (Fig. 16B)^Endnote 12^	***Zonophryxus* sp.**

### ﻿Key F. Suborder Limnoriidea, Superfamily Limnorioidea: Family Limnoriidae

Fig. 17

**Figure 17.**
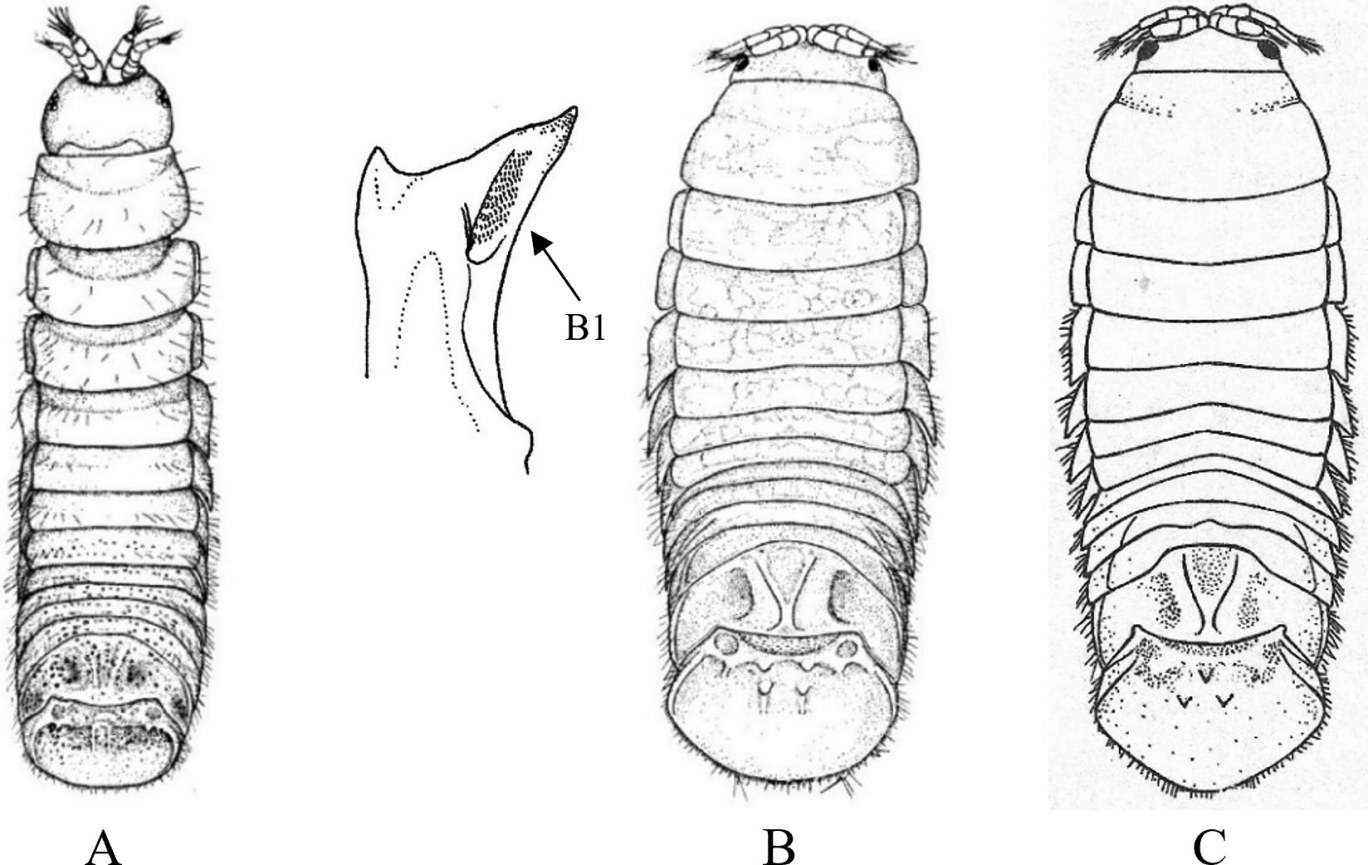
Isopoda, Limnoriidea, Limnorioidea, Limnoriidae: **A***Limnoriaalgarum* (after Menzies 1957) **B***Limnoriaquadripunctata***B1** inner surface of left mandible showing “rasp” (after Menzies 1957) **C***Limnoriatripunctata* (after Allen 1976).

**Table:** 

1	Dorsal surface of pleotelson without symmetrically arranged tubercles; left mandible without rasp or file-like ridges; burrowing in algal holdfasts (Fig. 17A)	***Limnoriaalgarum***
–	Dorsal surface of pleotelson with 3 or 4 symmetrically arranged anterior tubercles; left mandible with rasp or file-like ridges (see Fig. 17B); burrowing in wood	**2**
2	Dorsal surface of pleotelson with 3 anterior tubercles arranged in triangle; lateral and posterior margins of pleotelson tuberculate (Fig. 17C)	***Limnoriatripunctata***
–	Dorsal surface of pleotelson with 2 pairs of anterior tubercles; margins of pleotelson not tuberculate (Fig. 17B)	***Limnoriaquadripunctata***

### ﻿Key G. Suborder Sphaeromatidea, Superfamilies Seroloidea and Sphaeromatoidea

Figs 18–23

**Figure 18.**
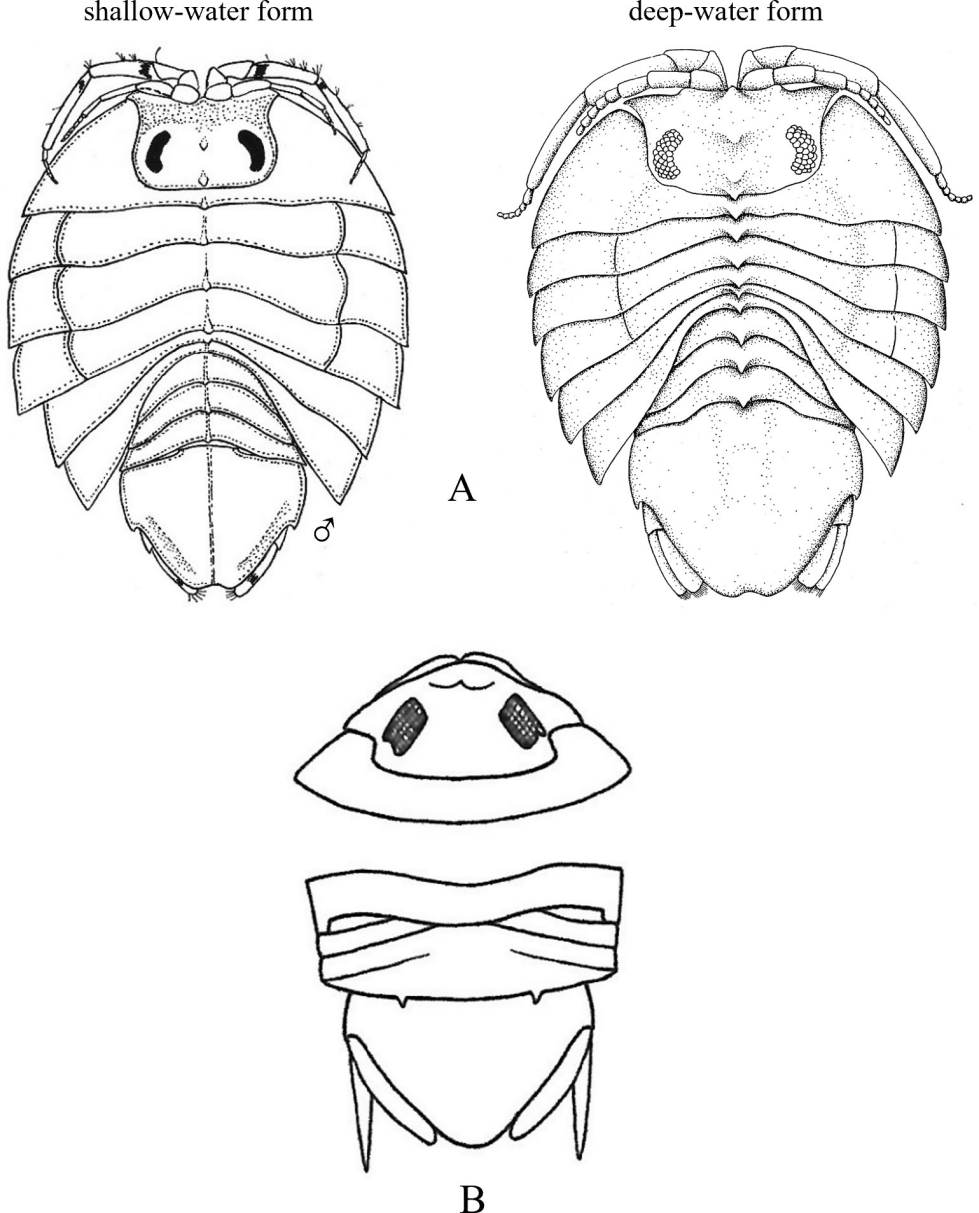
Isopoda, Sphaeromatidea, Seroloidea, Serolidae: **A***Heteroseroliscarinata* (shallow-water form after Menzies and Barnard 1959; deep-water form after Wetzer and Brusca 1997). Tecticipitidae: **B***Tecticepsconvexus* (after Richardson 1905a; Schultz 1969).

**Figure 19.**
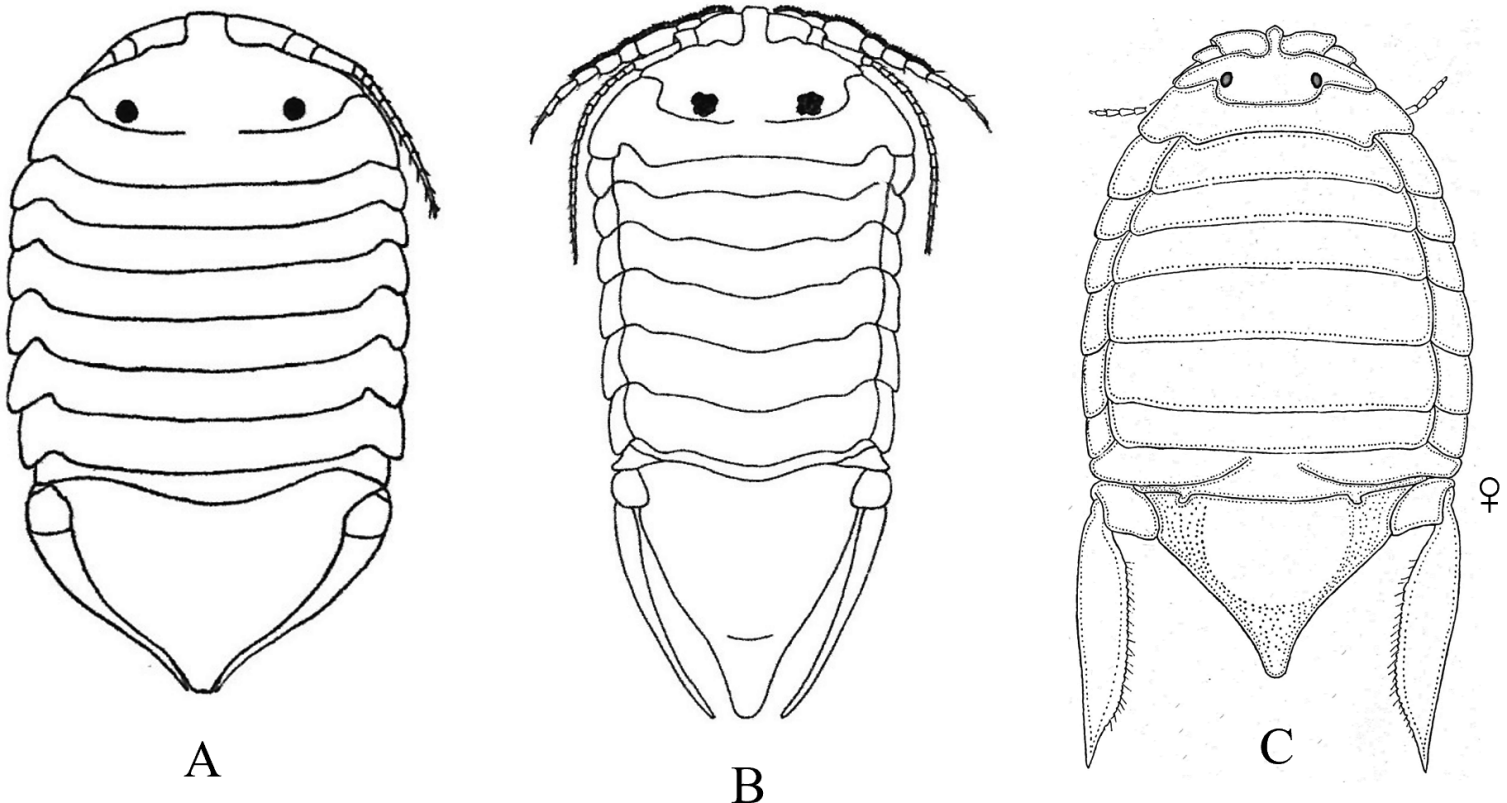
Isopoda, Sphaeromatidea, Sphaeromatoidea, Ancinidae: **A***Ancinusgranulatus* (after Holmes and Gay 1909) **B***Ancinusseticomvus* (after Iverson 1982) **C***Bathycopeadaltonae* (after Menzies and Barnard 1959).

**Figure 20.**
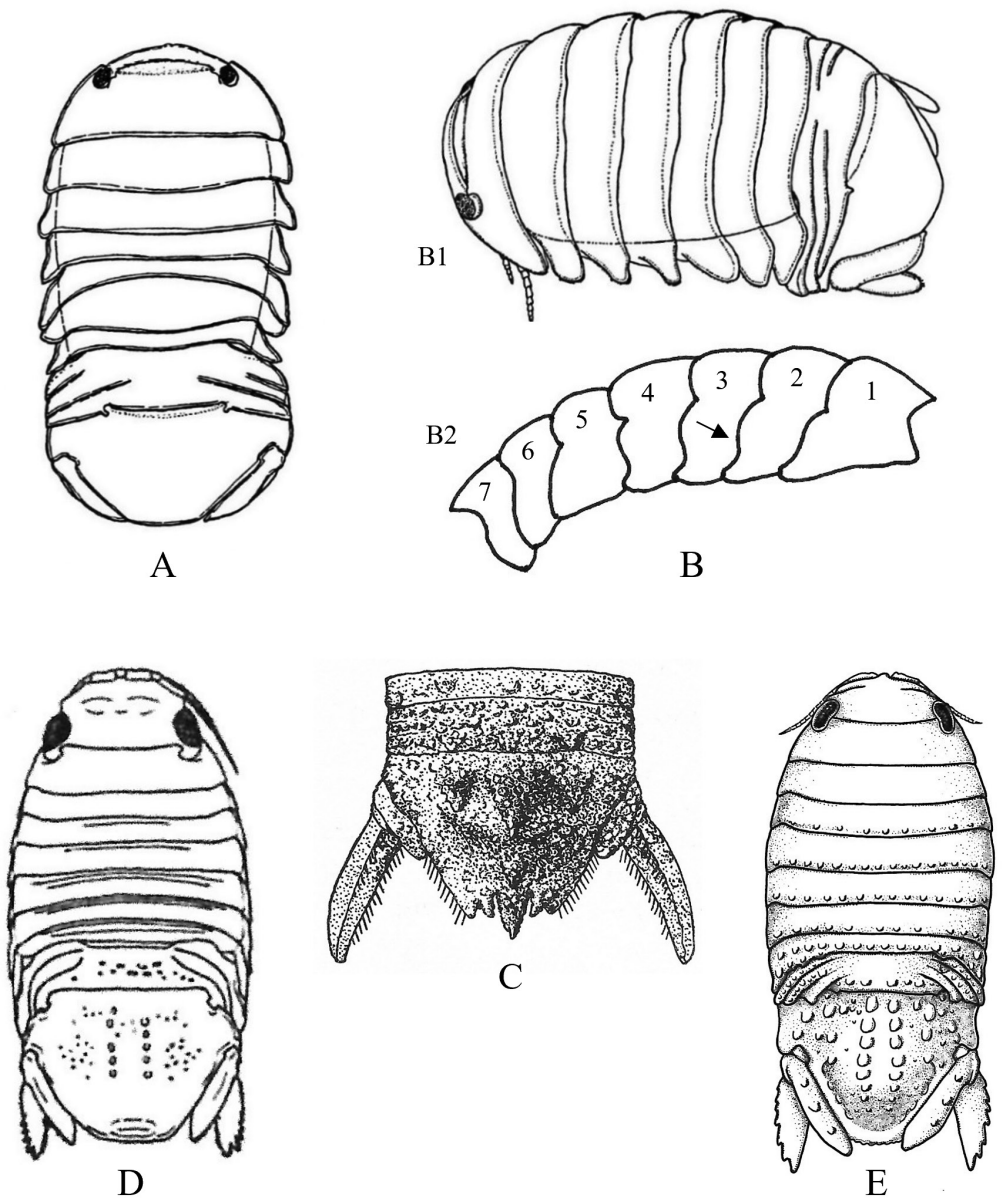
Isopoda, Sphaeromatidea, Sphaeromatoidea, Sphaeromatidae (in part): **A***Gnorimosphaeromanoblei* (after Menzies 1954) **B***Gnorimosphaeromaoregonense***B1** dorsal view (after Menzies 1954) **B2** lateral view of pereonites 1–7 showing S-shaped coxae of anterior pereonites (e.g., arrow) **C***Discerceisgranulosa* (after Richardson 1899, 1905a) **D***Sphaeromaquoianum* (after Harrison and Holdich 1984) **E***Sphaeromawalkeri* (after Kensley and Schotte 1989).

**Figure 21.**
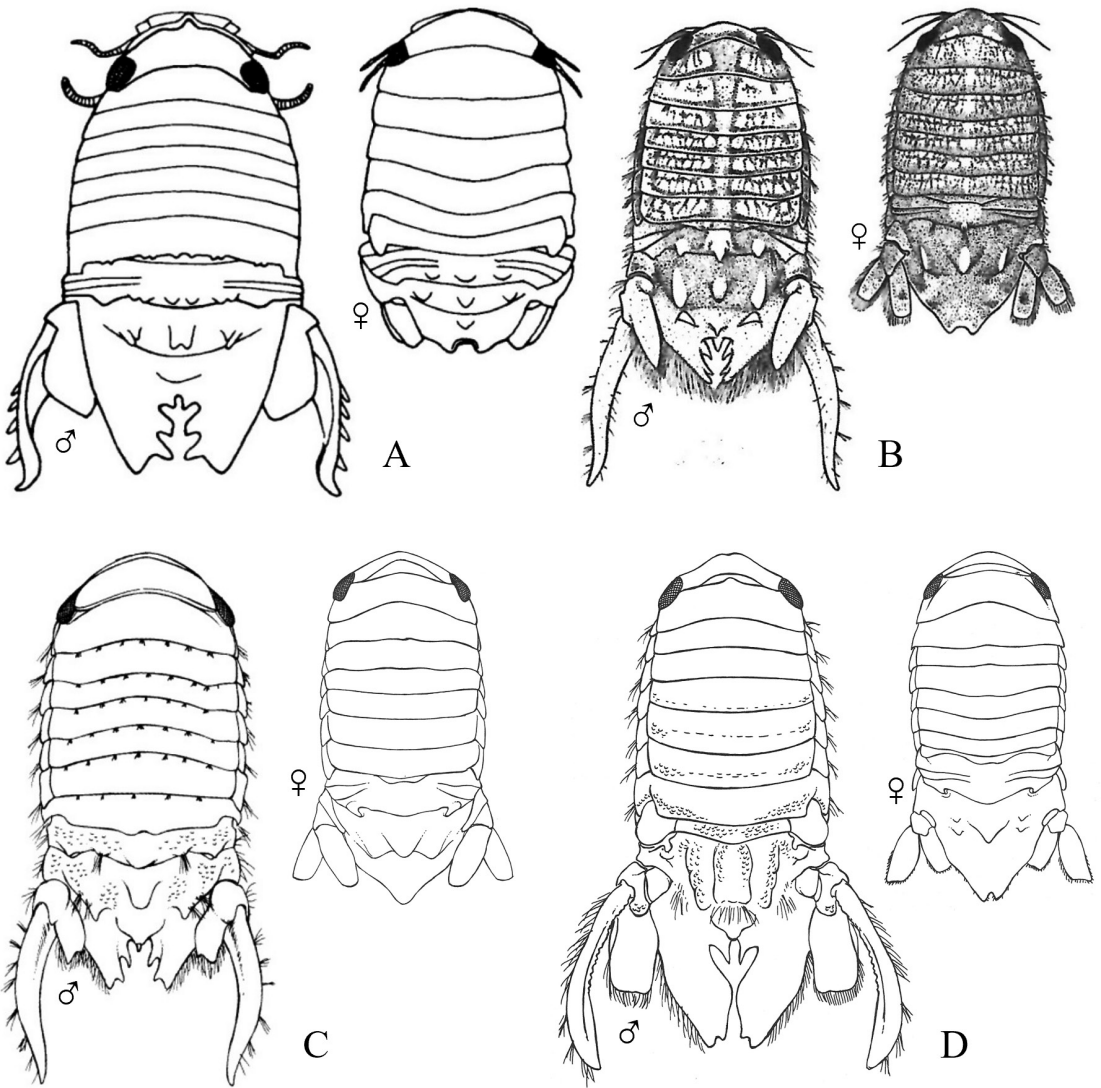
Isopoda, Sphaeromatidea, Sphaeromatoidea, Sphaeromatidae (in part): **A***Paracerceiscordata* (after Schultz 1969) **B***Paracerceisgilliana* (after Allen 1976) **C***Paracerceissculpta* (after Brusca 1980) **D***Paracerceis* sp. A (after Brusca 1980).

**Figure 22.**
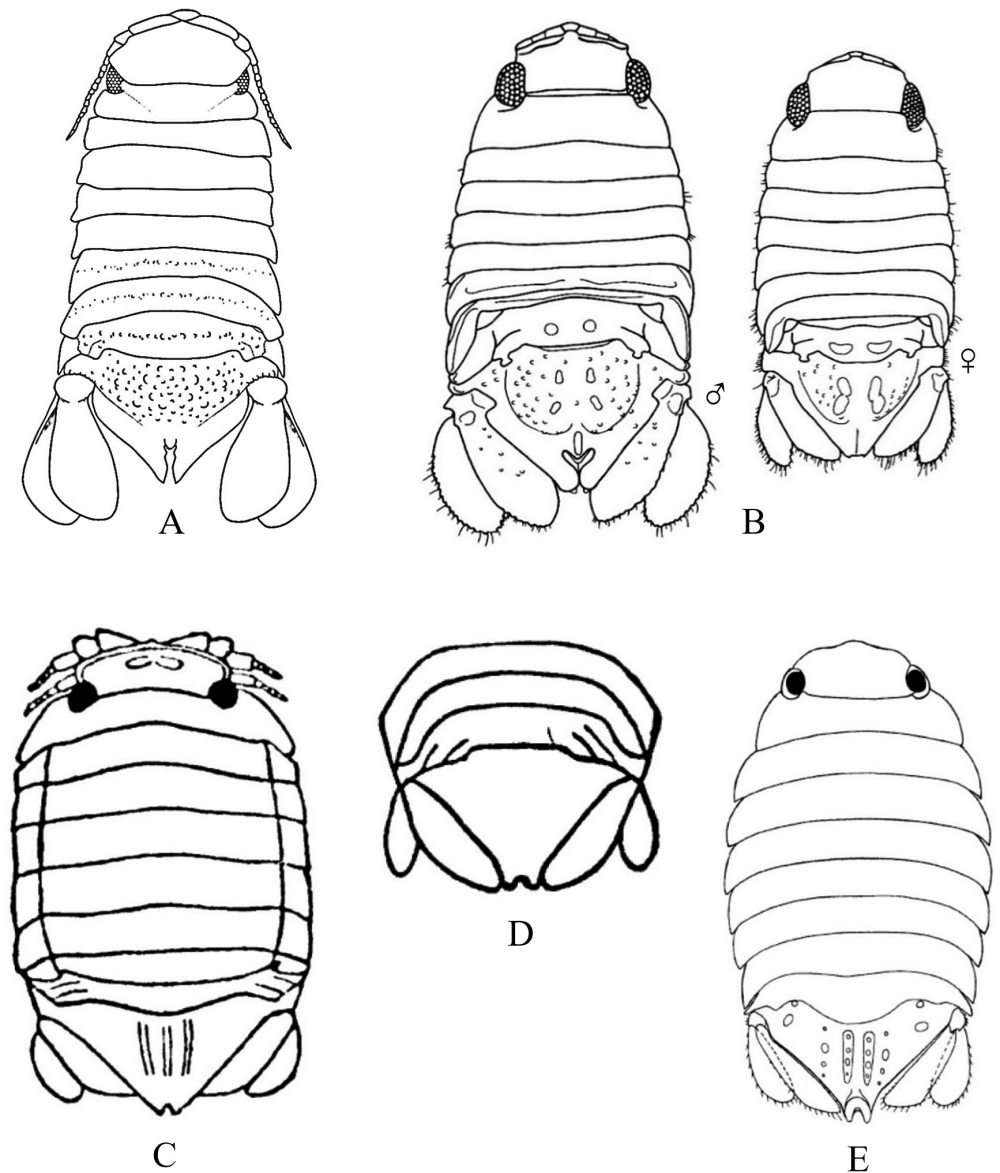
Isopoda, Sphaeromatidea, Sphaeromatoidea, Sphaeromatidae (in part): **A***Dynoideselegans* (after Wetzer and Mowery 2017) **B***Paradelladianae* (after Harrison and Holdich 1982) **C***Dynamenelladilatata* (after Richardson 1899, 1905a) **D***Dynamenellaglabra* (after Richardson 1899, 1905a) **E***Dynamenellasheareri* (after Brusca et al. 2007).

**Figure 23.**
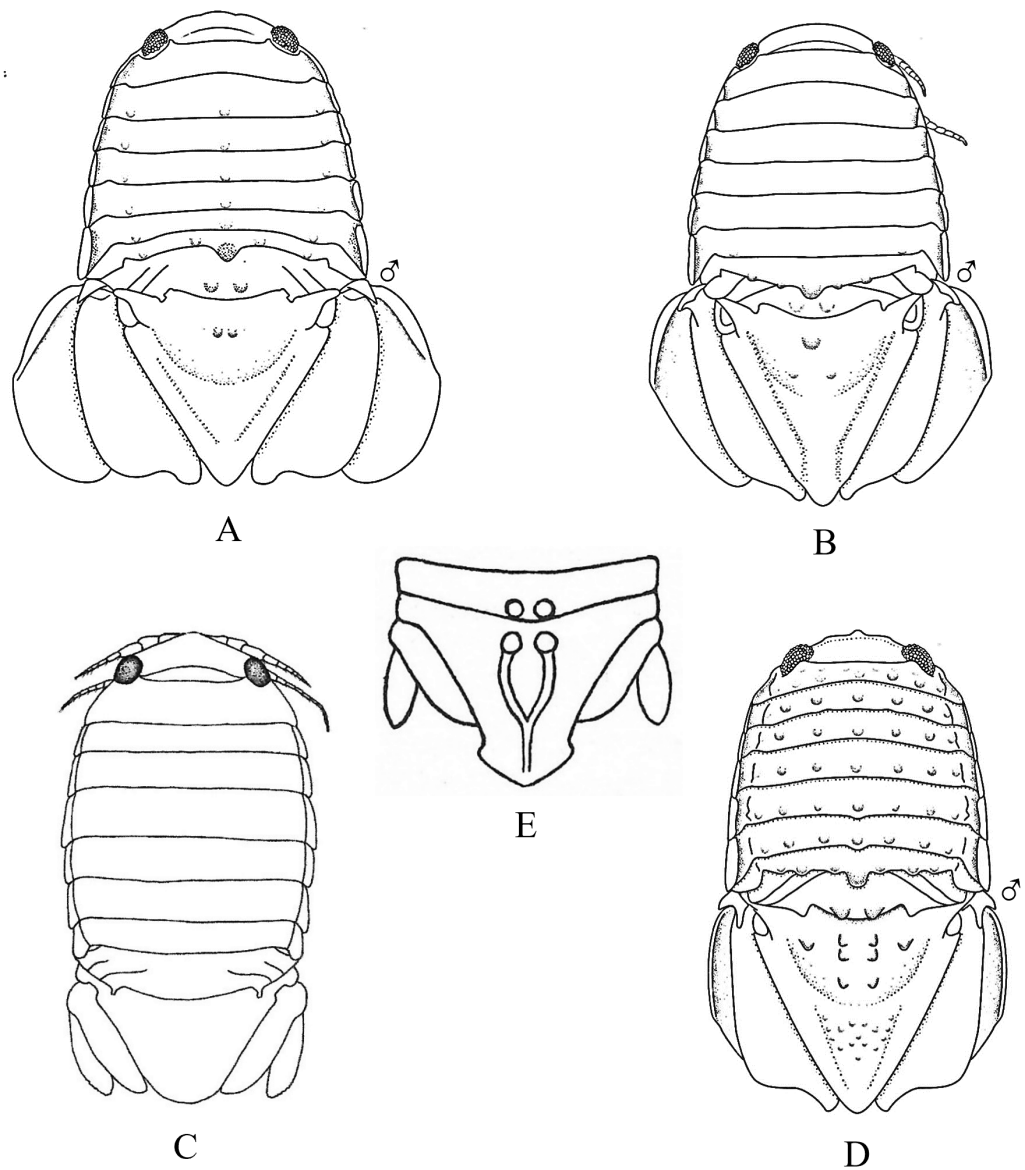
Isopoda, Sphaeromatidea, Sphaeromatoidea, Sphaeromatidae (in part): **A***Exosphaeromaamplicauda* (after Wall et al. 2015) **B***Exosphaeromaaphrodita* (after Wall et al. 2015) **C***Exosphaeromainornata* (after Iverson 1982) **D***Exosphaeromapentcheffi* (after Wall et al. 2015) **E***Exosphaeromarhomburum* (after Richardson 1899, 1905a).

**Table:** 

1	Pleon composed of 3 free pleonites plus pleotelson; body broad, depressed and platter-like; dorsum with distinct medial carinae (Fig. 18A)^Endnote 13^	***Heteroseroliscarinata*** [Serolidae]
–	Pleon composed of 1 or 2 dorsally visible free pleonites plus pleotelson	**2**
2	Pereopod 1 subchelate in both sexes, with broadly expanded propodus and prehensile dactylus (propodus at least 5 × wider than dactylus); pereopod 2 prehensile only in males	**3**
–	Pereopod 1 ambulatory or only weakly prehensile, propodus narrow (propodus < 2 × as wide as dactylus)	**6** [Sphaeromatidae]
3	Head medially fused to pereonite 1; pleopod 5 with both rami lacking transverse pleats or folds; uropods uniramous, lacking exopods	**4** [Ancindae]
–	Head and pereonite 1 not fused; both rami of pleopod 5 with transverse pleats or folds; uropods biramous, exopods slender and spine-like; first segment of pleon with 3 suture lines (fused pleonites 1–4) and 2 small triangular processes on posterior margin; pleotelson with rounded posterior border (Fig. 18B)	***Tecticepsconvexus*** [Tecticipitidae]
4	Lateral margins of head strongly produced; pleon with 2 short posterior projections overhanging anterior margin of pleotelson; pleopod 1 biramous; uropodal ramus narrow proximally, then expands at least twofold for ~ 80% of length before tapering to acute point (Fig. 19C)	***Bathycopeadaltonae***
–	Lateral margins of head weakly produced; pleon without posterior projections; pleopod 1 uniramous; uropodal ramus styliform, widest proximally and tapering to an acute point	**5**
5	Body broad (L:W ratio ~ 1.7) with densely granulated surfaces; pereonite 1 narrower than pereonites 2–7; pleotelson inflated, distinctly wider than long with truncate apex; eyes slightly elevated on swellings; uropods strongly recurved distally (Fig. 19A)^Endnote 14^	***Ancinusgranulatus***
–	Body relatively narrow (L:W ratio > 2.0) with smooth surfaces; pereonite 1 wider than pereonites 2–7; pleotelson not inflated, longer than wide with nearly acute or narrowly rounded apex; eyes not elevated; distal tips of uropods only slightly recurved (Fig. 19B)^Endnote 14^	***Ancinusseticomvus***
6	Endopods of pleopods 4 and 5 without branchial pleats or folds	**7**
–	Endopods of pleopods 4 and 5 with branchial pleats or folds	**8**
7	In lateral view, anterior margins of coxal plates of pereonites 2–4 appear raised and posterior margins not raised, giving the coxae a somewhat S-shaped appearance; species is fully marine (Fig. 20B)^Endnote 15^	***Gnorimosphaeromaoregonense***
–	In lateral view, anterior margins of coxal plates 2–4 are not raised, and coxae do not appear S-shaped; species occurs in brackish or freshwater habitats (Fig. 20A)	***Gnorimosphaeromanoblei***
8	Pleopods 4 and 5 with branchial pleats on both rami	**9**
–	Pleopods 4 and 5 with branchial pleats only on endopods	**19**
9	Uropods of male highly modified, each composed of elongated, cylindrical, or flattened exopod and very short endopod fused to the protopod; both uropodal rami lamellar in females; ovigerous females with 4 pairs of oostegites on pereonites 1–4	**10**
–	Uropods lamellar in both sexes; ovigerous females with 3 pairs, 1 pair, or no pairs of oostegites	**14**
10	Pleotelson of males with pronounced posteromedial tooth that completely fills apical notch and extends posteriorly beyond level of notch opening; dorsum of pleotelson with 3 transverse elevations at base, median elevation terminating in a spine; body surface densely granulated; sexual dimorphism pronounced with female body smooth, lacking ornamentation or setae (Fig. 20C)	***Discerceisgranulosa***
–	Pleotelson of males with highly complex and open apical notch, internal lateral margins of notch with various numbers of teeth forming sinuses of different shapes; body surface not densely granulated; sexual dimorphism pronounced, females without complex pleotelsonic notch, terminal notch either very short and simple or absent; dorsum of pleotelson sculptured with various types of tubercles	**11**
11	Uropods of male with ventrolateral spines on exopods; female pleotelson stout, with 4 dorsal tubercles and wide, but shallow apical notch (Fig. 21A)	***Paracerceiscordata***
–	Uropods of male without spines; female pleotelson not as above	**12**
12	Pleotelson of males long, subequal in length to pereon, with complex medial sinus formed by 2 pairs of teeth, pleotelsonic sinus expanded basally into round foramen overhung by basal knob bearing tall acute spine (spine length ≥ 4 × diameter), sinus then narrowing distally to long thin channel; female pleotelson with small distal notch and 5 dorsal tubercles (1 large medial, 4 smaller lateral); female uropodal exopods with sharply toothed posteromedial margins (Fig. 21D)	***Paracerceis* sp. A**
–	Pleotelson of males relatively short, ca. half length of pereon; pleotelsonic sinus not as above, without round basal foramen, with or without short spine overhanging base of notch; female uropodal exopods without sharply toothed margins	**13**
13	Male pleotelson with short acute spine (i.e., length subequal to diameter) on basal knob overhanging sinus, interior margins of sinus with 2 pairs of lateral teeth; pleotelson of female elongate, acuminate posteriorly, with 3 dorsal tubercles (Fig. 21C)^Endnote 16^	***Paracerceissculpta***
–	Male pleotelson with large dorsal tubercle at base of sinus, interior margins of sinus with 3 pairs of sharp teeth; female pleotelson not as above (Fig. 21B)	***Paracerceisgilliana***
14	Pleotelson apex entire, upturned; dorsal surface of pleotelson with 2 pairs of prominent tubercles and numerous scattered small tubercles; female sculpturing less pronounced than males (see Bruce and Wetzer 2008: fig. 1)	***Pseudosphaeroma* sp.**
–	Pleotelson without upturned apex, sculpturing not as above	**15**
15	Pleotelson of males with deeply slit apical notch or expanded foramen, notch may be reduced to small depression or dorsally visible slit in females; uropodal rami subequal in length, typically extending beyond posterior margin of pleotelson; ovigerous females with 1 or 3 pairs of oostegites	**16**
–	Pleotelson of males and females with shallow terminal notch; uropodal exopod shorter than endopod, rami not extending beyond posterior border of pleotelson; ovigerous females without oostegites	**17**
16	Pleotelsonic sinus (foramen) distinctly heart shaped with median point in males, but greatly reduced in females; dorsum of pleon with 2 rounded submedian tubercles just lateral to midline on pleonite 5, and 4 similarly spaced tubercles on pleotelson; uropodal rami with crenulated margins (at least in males) (Fig. 22B)	***Paradelladianae***
–	Pleotelsonic sinus long and narrow, with prominent rounded tubercle barely overhanging anterior base, sinus walls straight sided and finely crenulate; dorsal surface of pleotelson covered with small tubercles; uropodal rami without crenulated margins (Fig. 22A)	***Dynoideselegans***
17	Frontal margin of head produced as a quadrangular process; antennular articles 1 and 2 dilated; dorsal surface of pleotelson sculptured with 3 longitudinal ridges (Fig. 22C)^Endnote 17^	***Dynamenelladilatata***
–	Frontal margin of head not produced; antennular articles not dilated; dorsal surface of pleotelson smooth or tuberculate	**18**
18	Dorsal surface of pleotelson with many tubercles (Fig. 22E)	***Dynamenellasheareri***
–	Dorsal surface of pleotelson smooth, without tubercles or ridges (Fig. 22D)^Endnote 17^	***Dynamenellaglabra***
19	Uropodal exopods with distinctly serrated outer margins	**20**
–	Uropodal exopods with smooth or lightly crenulated outer margins	**21**
20	Dorsal surface of pleotelson lightly sculptured with 2 longitudinal rows of small tubercles, posterior margin with prominent transverse elevation (Fig. 20D)	***Sphaeromaquoianum***
–	Dorsal surface of pleotelson heavily sculptured with many longitudinal rows of small to large heavy tubercles, posterior margin without prominent transverse elevation (Fig. 20E)	***Sphaeromawalkeri***
21	Dorsal surface of pereonites, pleonites and pleotelson smooth, without tubercles or other ornamentation; apex of pleotelson rounded and truncate (Fig. 23C)^Endnote 18^	***Exosphaeromainornata***
–	Dorsal surface of body with ornamentation; apex of pleotelson acumi­nate^Endnote 18^	**22**
22	Pleotelson with produced rhomboid-shaped apical process and concave lateral margins, sides folded inward, forming kind of funnel-like opening when viewed ventrally; dorsum of pleotelson sculptured with 2 basal tubercles, which are continuous with 2 longitudinal ridges that converge posteriorly into a single median ridge; uropods distinctly shorter than pleotelson (Fig. 23E)^Endnote 19^	***Exosphaeromarhomburum***
–	Pleotelson triangular, dorsum without parallel ridges; uropods subequal in length to pleotelson	**23**
23	Dorsal sculpturing subtle, starting on pereonites 6 and 7; pereonite 6 with row of 7 weak tubercles along posterior margin; pereonite 7 with posteriorly directed median process and weak lateral tubercle on each side along posterior margin; pleon with 2 medium tubercles, 1 on each side of midline; pleotelson with 1 strong anteromedial tubercle and 2 weak tubercles lateral to midline (Fig. 23B)	***Exosphaeromaaphrodita***
–	Dorsal sculpturing distinct on most or all pereonites, comprising either low, weak tubercles or taller, more pronounced tubercles	**24**
24	Dorsal surface of pereonites with low, weak tubercles along posterior margins; pereonite 1 with 1 very weak median tubercle; pereonites 2–4 with 3 tubercles, 1 median and 2 laterals (1 per side); pereonites 5 and 6 with row of 7 tubercles, 1 at midline and 3 laterals per side; pereonite 7 with weak median process and paired lateral tubercles; pleotelson with 2 small anterior tubercles (Fig. 23A)	***Exosphaeromaamplicauda***
–	Dorsal surface of all pereonites sculptured with heavy, strong tubercles giving body appearance of 7 prominent longitudinal rows of tubercles; pereonite 1 with 2 transverse rows of tubercles; pereonites 2–7 each with 1 transverse row of 7 tubercles; pereonite 7 with strong median process and 3 lateral tubercles per side; pleotelson with 3 strong medial tubercles on either side of midline, plus numerous smaller tubercles scattered over dorsal surface (Fig. 23D)	***Exosphaeromapentcheffi***

### ﻿Key H. Suborder Valvifera

Figs 24–30

**Figure 24.**
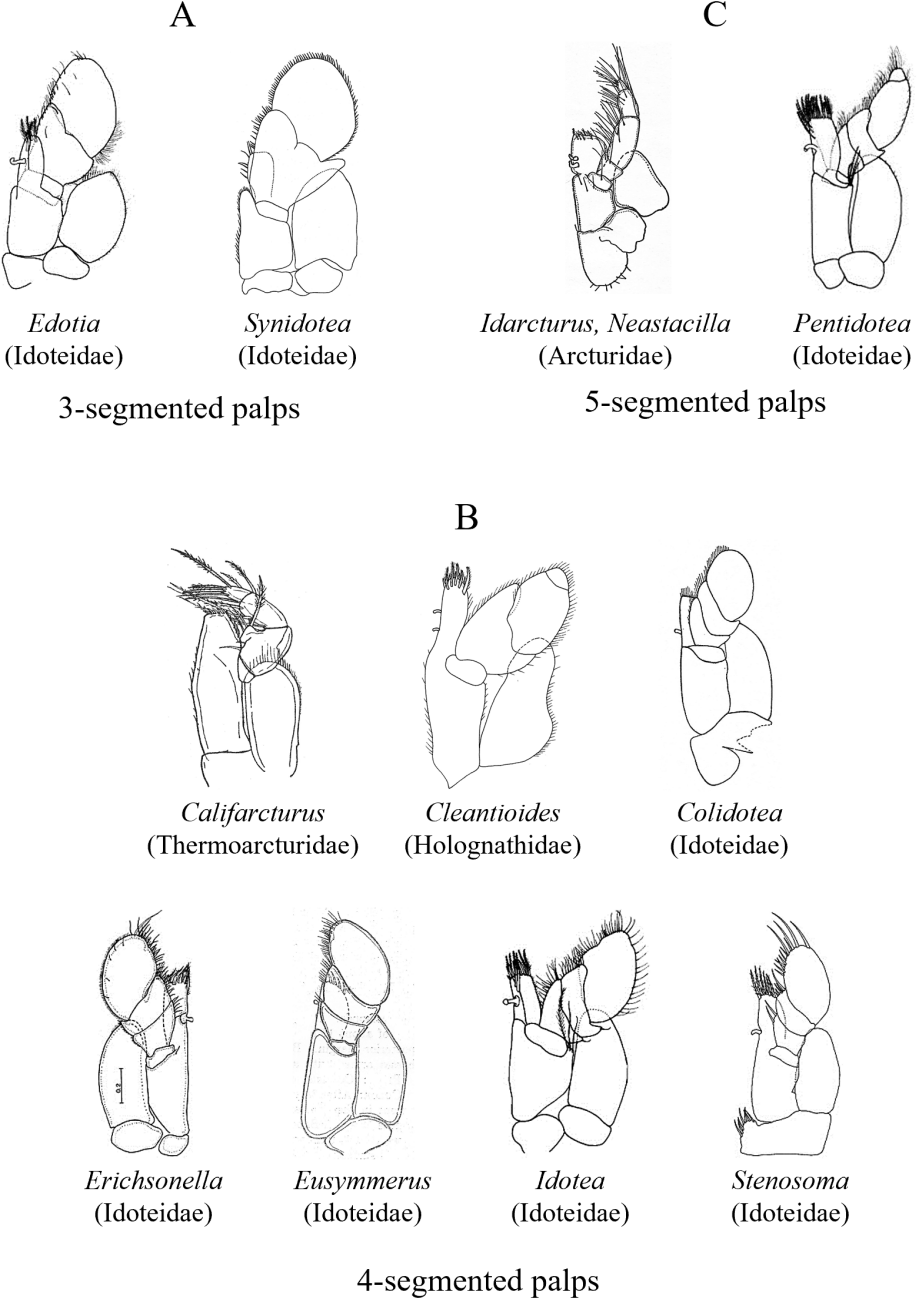
Comparison of maxillipeds and number of articles in maxillipedal palps (3–5) between genera and families of SCB valviferan isopods: **A** 3-article maxillipedal palps **B** 4-article maxillipedal palps **C** 5-article maxillipedal palps.

**Figure 25.**
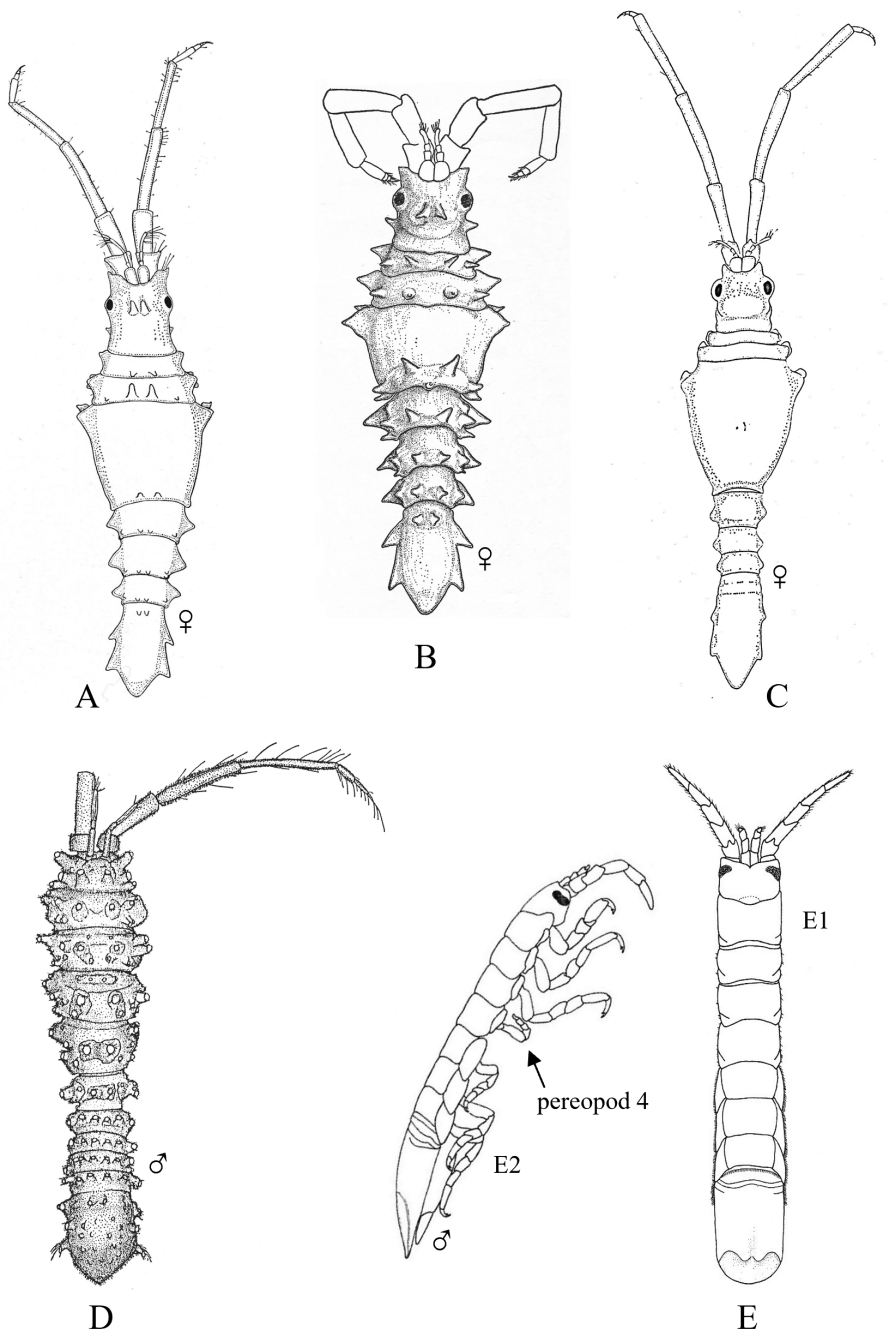
Isopoda, Valvifera, Arcturidae: **A***Idarcturusallelomorphus* (after Menzies and Barnard 1959) **B***Idarcturushedgpethi* (after Menzies 1951b) **C***Neastacillacalifornica* (after Menzies and Barnard 1959). Thermoarcturidae: **D***Califarcturustannerensis* (after Schultz 1966). Holognathidae: **E***Cleantioidesoccidentalis***E1** dorsal view (after Brusca and Wallerstein 1979a) **E2** lateral view of *C.verecundus* from Florida showing reduced size of pereopod 4 characteristic of *Cleantioides* (after Kensley and Clark 1998).

**Figure 26.**
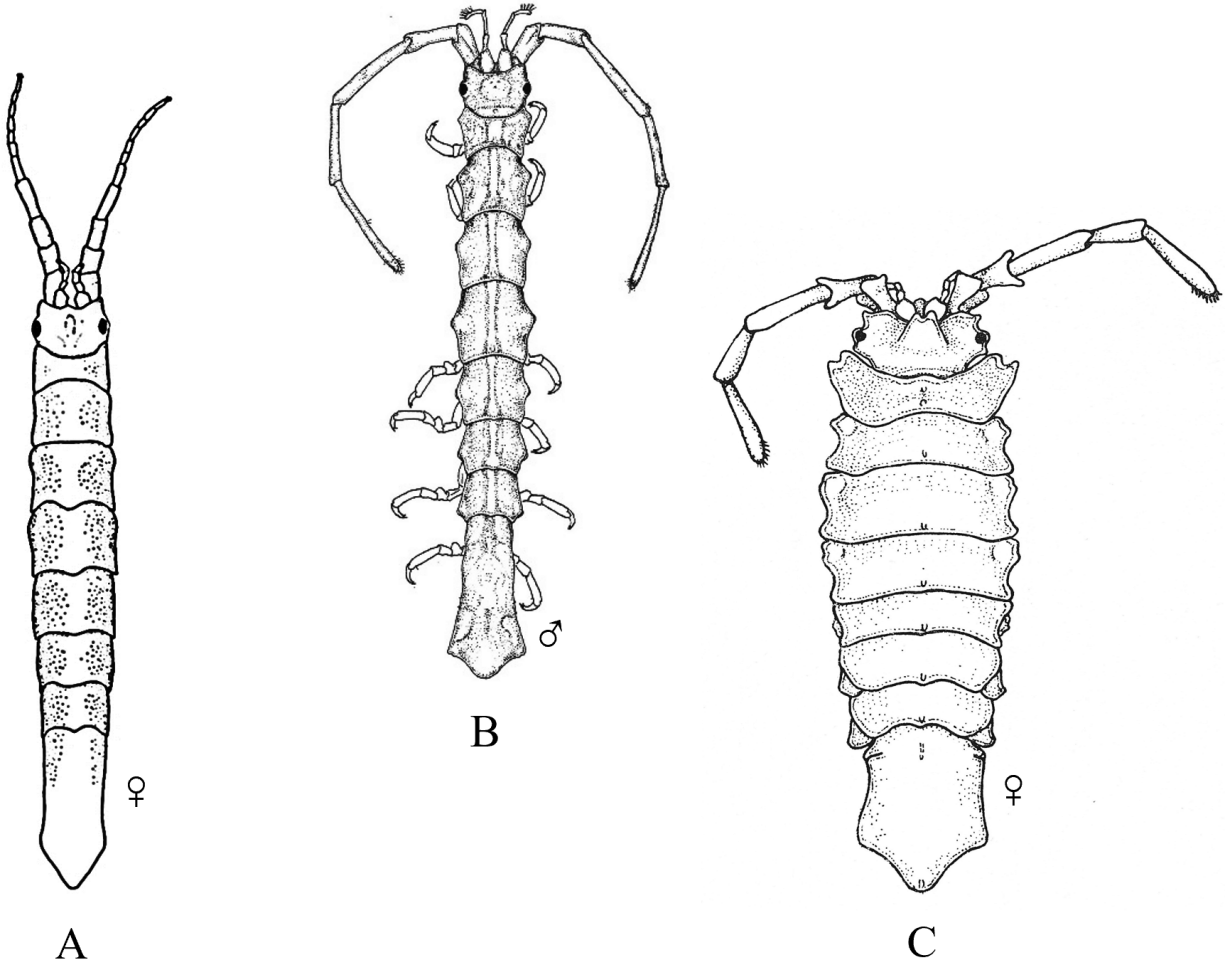
Isopoda, Valvifera, Idoteidae (in part): **A***Stenosomawetzerae* (after Ormsby 1991) **B***Erichsonellacrenulata* (after Menzies 1950b) **C***Eusymmeruspseudoculata* (after Menzies and Bowman 1956).

**Figure 27.**
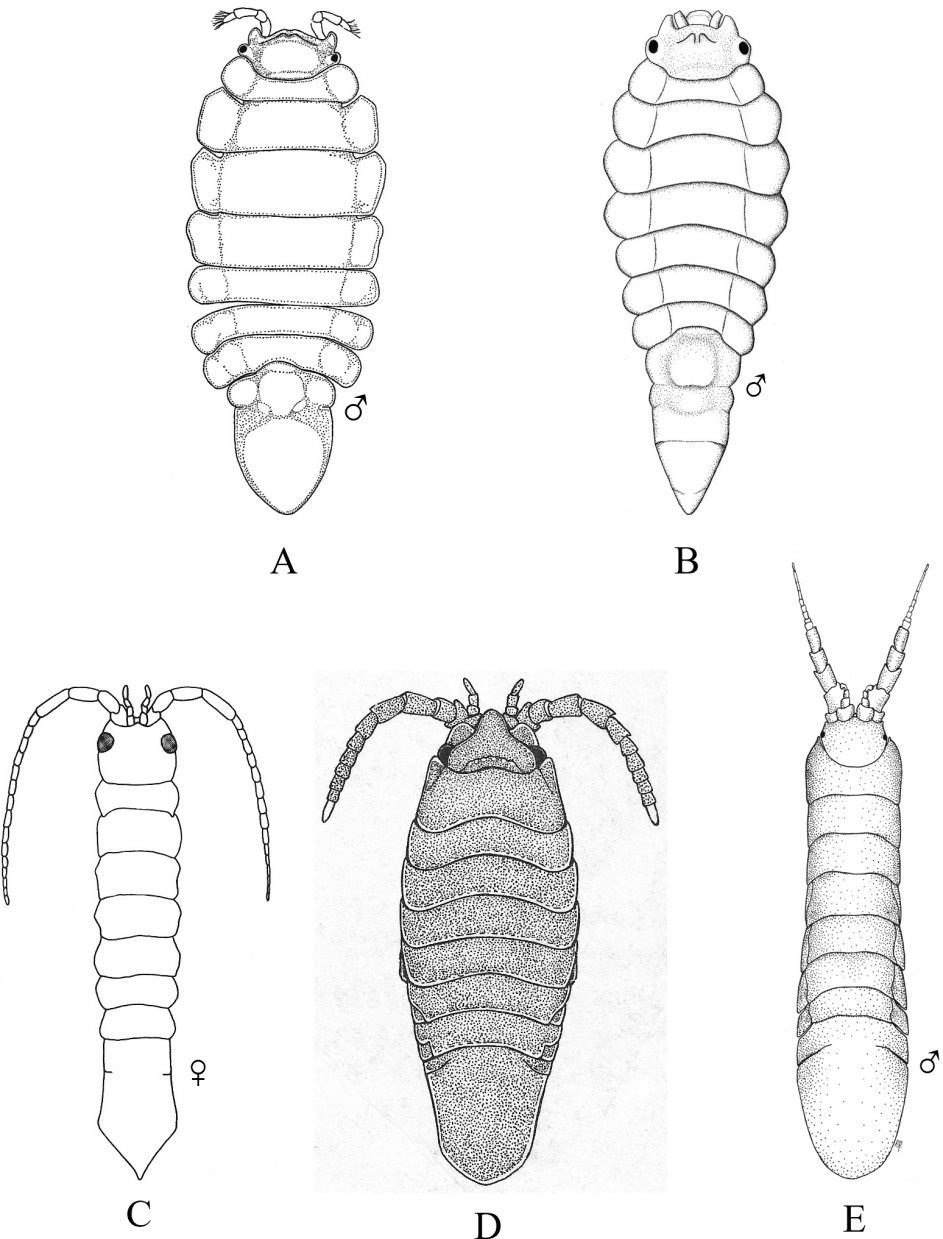
Isopoda, Valvifera, Idoteidae (in part): **A***Edotiasublittoralis* (after Menzies and Barnard 1959) **B***Edotia* sp. B (after Stebbins 2012c) **C***Colidoteafindleyi* (after Brusca and Wallerstein 1977) **D***Colidotearostrata* (after Allen 1976) **E***Colidoteawallersteini* (after Brusca 1983b).

**Figure 28.**
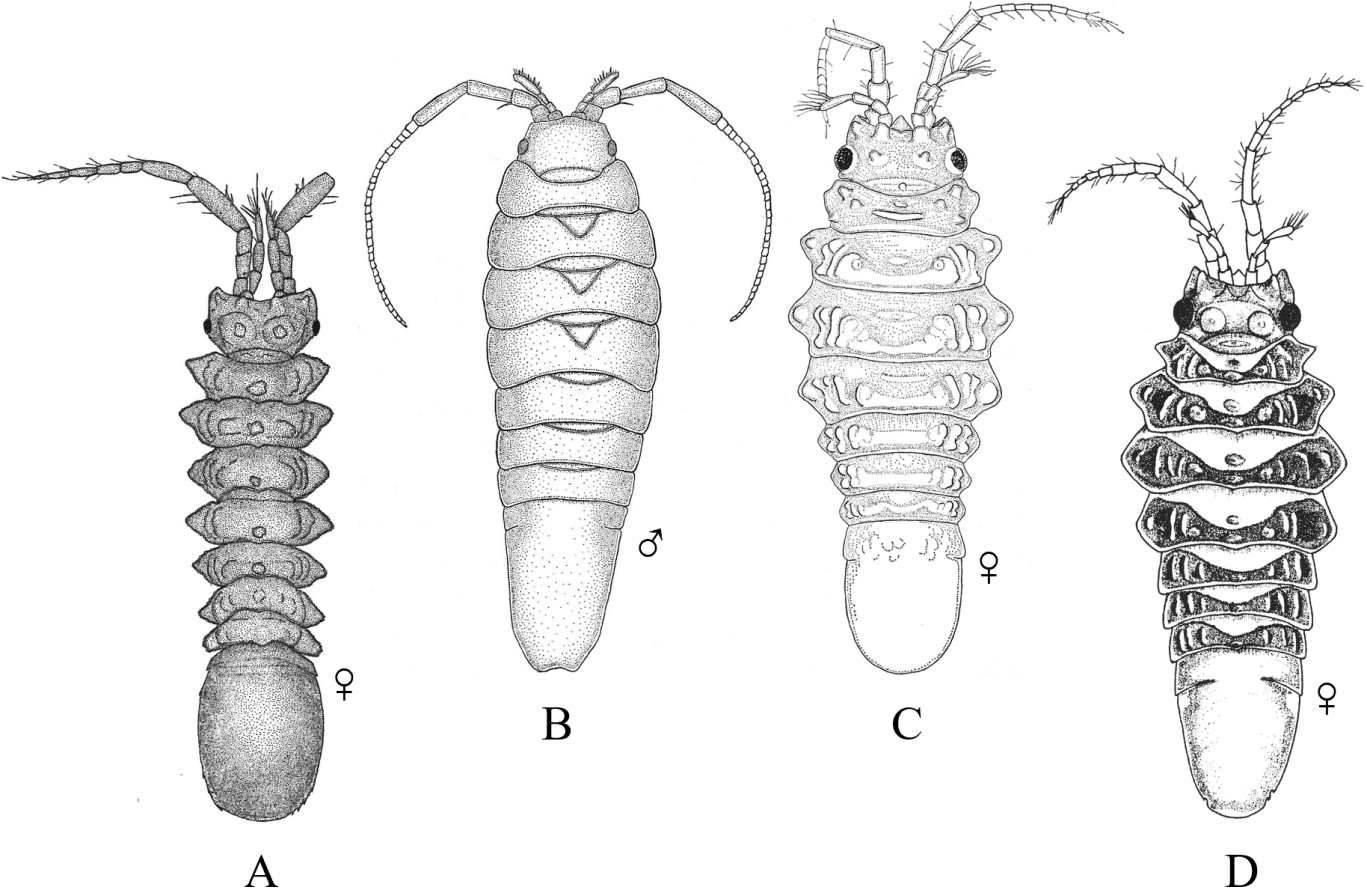
Isopoda, Valvifera, Idoteidae (in part): **A***Synidoteacalcarea* (after Schultz 1966) **B***Synidoteaharfordi* (after Menzies and Miller 1972) **C***Synidoteamagnifica* (after Menzies and Barnard 1959) **D***Synidoteamedia* (after Iverson 1972).

**Figure 29.**
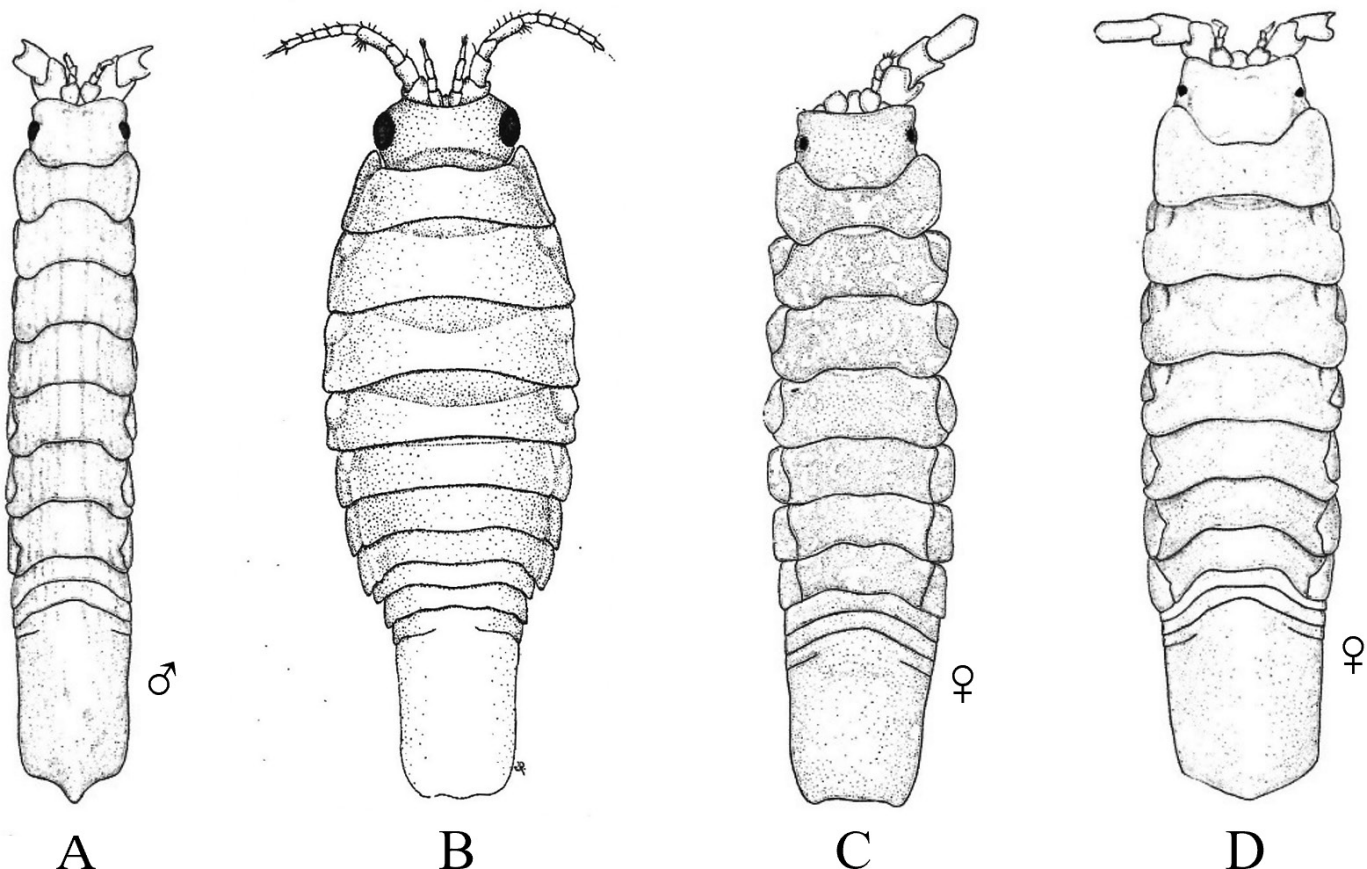
Isopoda, Valvifera, Idoteidae (in part): **A***Idoteafewkesi* (after Menzies 1950a) **B***Idoteametallica* (after Brusca and Wilson 1991) **C***Idotearufescens* (after Menzies 1950a) **D***Idoteaurotoma* (after Menzies 1950a).

**Figure 30.**
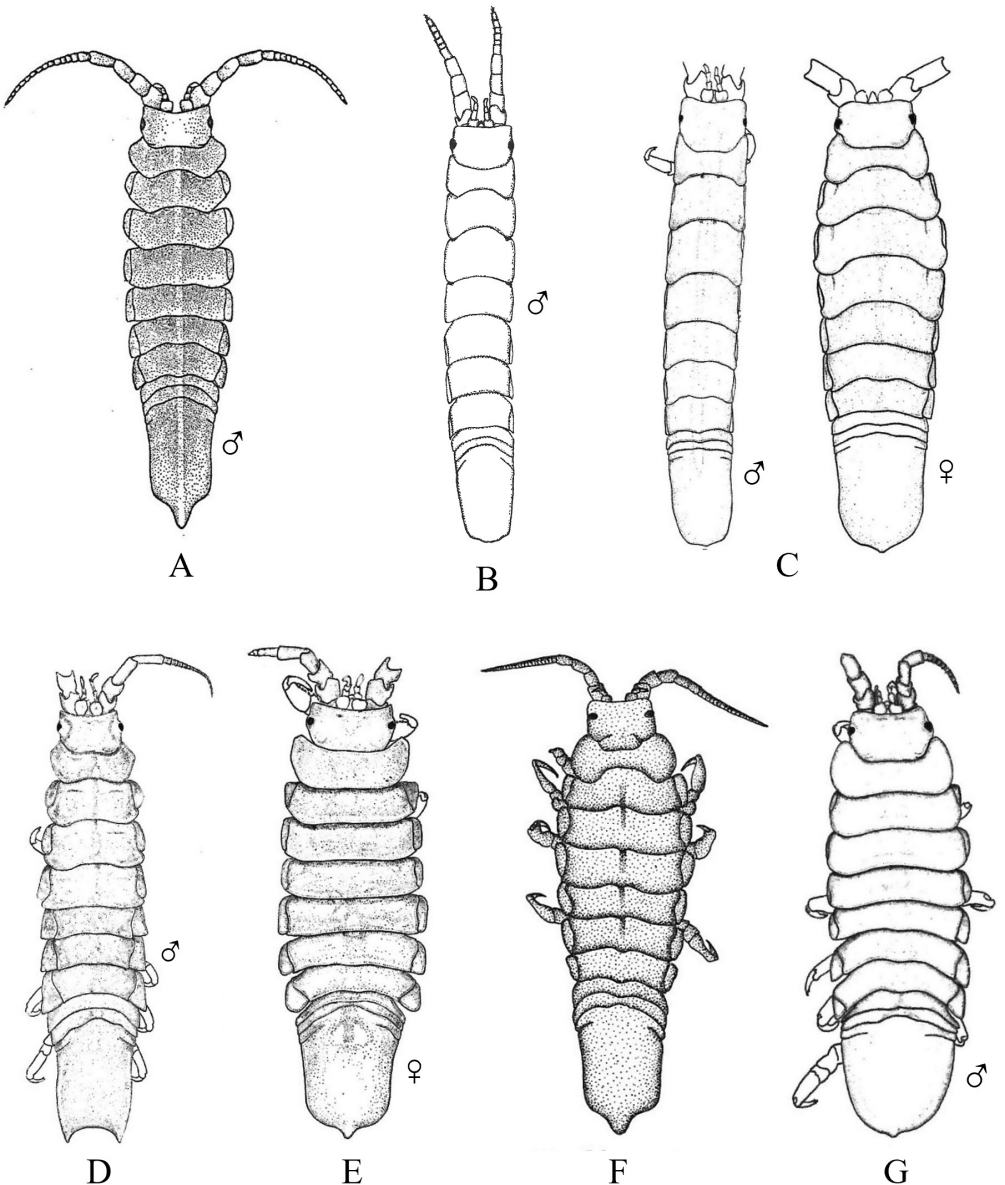
Isopoda, Valvifera, Idoteidae (in part): **A***Pentidoteaaculeata* (after Stafford 1913b) **B***Pentidoteakirchanskii* (after Miller and Lee 1970) **C***Pentidoteamontereyensis* (after Menzies 1950a) **D***Pentidotearesecata* (after Menzies 1950a) **E***Pentidoteaschmitti* (after Menzies 1950a) **F***Pentidoteastenops* (after Brusca and Brusca 1978) **G***Pentidoteawosnesenskii* (after Menzies 1950a).

**Table:** 

1	Eyes absent; head, pereonites and pleonites with transverse rows of submedian, sublateral and lateral tubercles bearing bead-like spheres; pleotelson with short oblique lateral tubercles; maxillipedal palp 4-segmented (Figs 24B, 25D)	***Califarcturustannerensis*** [Thermoarcturidae]
–	Eyes present; body not as above	**2**
2	Head fused with pereonite 1; pereonite 4 much longer (> 2 ×) than other pereonites; pereonite 4 much broader anteriorly than posteriorly in females; pereopods 1–4 slender, fringed with setae, directed anteriorly against ventral body wall (modified for filter feeding); pereopods 5–7 stout, prehensile; maxillipedal palp 5-segmented (Figs 24C, 25A–C)	**3** [Arcturidae]
–	Head distinct from pereonite 1; length of pereonite 4 subequal to other pereonites; anterior pereopods not modified for filter feeding, mostly ambulatory and similar in size	**6**
3	Head incompletely fused with pereonite 1, indicated by distinct lateral incision posterior and ventral to eye (must be viewed laterally); flagellum of antennae with ventral blade-like setae; dorsum of pereonite 4 smooth in males or with medial, bilobed swelling or tubercle in females; dorsum of other pereonites mostly smooth; pleon composed of 3 segments, including 2 pleonites plus the pleotelson (Fig. 25C)	***Neastacillacalifornica***
–	Head completely fused with pereonite 1, lateral margins entire; flagellum of antennae without blade-like setae; dorsum of pereonites 2–7 typically with 2–4 small to large dorsal spines in females or smooth in males; pleon composed of a single fused pleotelson with 2 anterodorsal median spines	**4**
4	Fused head and pereonite 1 with shallow dorsal transverse groove (fusion line) posterior to eyes; antennae robust with peduncular articles 3 and 4 each ~ 5 × longer than wide; pereonites 2–7 with large, broadly triangulate anterolateral or lateral extensions and a transverse row of 4 large posterodorsal spines; pereonites 4–6 also with medium to large posterolateral spines (Fig. 25B)^Endnote 20^	***Idarcturushedgpethi***
–	Fused head and pereonite 1 without dorsal transverse groove; antennae relatively long and narrow with peduncular articles 3 and 4 each > 10 × longer than wide; lateral extensions of pereonites 2–7 not as above, and each one with pair of medial posterodorsal spines (size variable); pereonites 5–7 also with 2 smaller distinct lateral spines	**5**
5	Body color typically golden; pereonites 2–4 anterolateral extensions medium in size, broad at base and distally blunt or obtusely rounded; pereonites 5–7 lateral extensions broadly triangular; anterolateral margins of head and antennal peduncular article 1 acute, but not produced forward as recurved spines (Fig. 25A)^Endnote 20^	***Idarcturusallelomorphus***
–	Body color whitish; pereonites 2–7 anterolateral or lateral extensions narrowly triangular and sharply acute; anterolateral margins of head and peduncular article 1 of antennae distinctly produced forward as sharply acute recurved spines^Endnote 20^	***Idarcturus* sp. A**
6	Pereopod 4 greatly reduced, non-ambulatory; pleon composed of 4 segments with pair of elevated dorsal humps near posterior end; lateral margins of body with dense tufts of setae (Fig. 25E)	***Cleantioidesoccidentalis*** [Holognathidae]
–	Pereopod 4 not reduced, all pereopods ambulatory; pleon composed of 1 or 3 segments; dorsal surface of pleon and lateral margins of body not as above	**7** [Idoteidae]
7	Pleon composed of single fused pleotelson, with or without partial suture lines or indentations	**8**
–	Pleon composed of 3 to 4 segments, including 1 pair of partial sutures	**19**
8	Pleotelson without suture lines	**9**
–	Pleotelson with 1 pair of partial suture lines or indentations	**10**
9	Flagellum of antennae multiarticulate, head with distinct, slightly elongated dorsal tubercle; pereon dorsally smooth (Fig. 26A)	***Stenosomawetzerae***
–	Flagellum of antennae uniarticulate, flagellar article large and clavate; head with large, multilobed or conical tubercle or elevation; pereonites 1–5 with (female) or without (male) middorsal spines (Fig. 26B)	***Erichsonellacrenulata***
10	Antennae shorter than or subequal to antennules, antennal flagellum reduced to single vestigial article; maxillipedal palp of 3 articles; pleonal fusion indicated by 1 pair of lateral grooves instead of distinct incisions	**11**
–	Antennae much longer than antennules, antennal flagellum multiarticulate or comprising a single, large clavate article; maxillipedal palp of 3 or 4 articles; pleotelson with distinct lateral incisions	**12**
11	Pleotelson with dorsal transverse ridge ca. midway between lateral insertions and posterior border, without dorsal swellings; lateral margins of pereonite 4 in females angular, but not forming acute posterolateral projections; propodi of pereopods with 2 large proximal spines along inferior margins (Fig. 27B)^Endnote 21^	***Edotia* sp. B**
–	Pleotelson inflated or bulbous with large dorsal swellings, without a dorsal transverse ridge; lateral margins of pereonite 4 in females form acute posterolateral projections; propodi of pereopods without large proximal spines along inferior margins (Fig. 27A)	***Edotiasublittoralis***
12	Flagellum of antennae uniarticulate, flagellar article large and clavate; head with large, median bifid tubercle projecting anteriorly over frontal margin; anterolateral margins of pereonite 1 produced into bilobed processes (Fig. 26C)	***Eusymmeruspseudoculata***
–	Antennal flagellum multiarticulate; head and pereonite 1 not as above	**13**
13	Maxillipedal palp 4-segmented (Fig. 24B)	**14**
–	Maxillipedal palp 3-segmented (Fig. 24A)	**16**
14	Eyes large, oval to subpyriform in shape, situated at mid-lateral margins of head; lateral margins of pleotelson flare slightly outward to form obtuse posterolateral angles, after which they taper inwards to strongly acuminate apex; coxal plates of pereonites not visible dorsally (Fig. 27C)	***Colidoteafindleyi***
–	Eyes small or large, situated laterally on head adjacent to anterolateral angles of pereonite 1; pleotelson not as above, with rounded or obtuse apex; coxal plates of some posterior pereonites visible dorsally	**15**
15	Eyes large; head not deeply immersed in pereonite 1, dorsal surface with a large, medial, apically rounded rostrum-like process; lateral margins of pereon convex, widest around pereonite 4; coxal plates visible dorsally on pereonites 5–7 (or just pereonites 6 and 7); commensal on sea urchins *Strongylocentrotuspurpuratus* and *Mesocentrotusfranciscanus* with body of live isopods matching purple or dark red color of host urchin, but fading to bluish gray in alcohol (Fig. 27D)	***Colidotearostrata***
–	Eyes very small; head deeply immersed in pereonite 1, without a large dorsal process; lateral margins of pereon not convex, generally straight-sided from pereonites 1–4 and then becoming slightly wider from pereonites 5–7; coxal plates visible dorsally on pereonites 4–7 (Fig. 27E)	***Colidoteawallersteini***
16	Body smooth, but with triangulate anterior median dorsal pattern on pereonites 2–4; lateral margins of pereonites rounded to straight; posterior margin of pleotelson emarginate (Fig. 28B)	***Synidoteaharfordi***
–	Body sculptured with conspicuous tubercles or longitudinal rugae; lateral margins of pereonites angular; pleotelson spatulate, rounded posteriorly^Endnote 22^	**17**
17	Lateral margins of adult body roughly parallel, widest part of pereon subequal in width to pleon; pleotelson widest at midpoint; dorsal sculpturing generally reduced to low, conical tubercles on head and medial row of tubercles along pereonites; eyes small and lightly pigmented (Fig. 28A)	***Synidoteacalcarea***
–	Lateral margins of adult body not parallel, generally widest at pereonites 3 and 4; pleotelson widest anteriorly; dorsal sculpturing variable, pereonites with 3 to 4 longitudinal rugae on lateral areas; eyes large and heavily pigmented	**18**
18	Flange present on basis of pereopods 2–6 in adults, but tiny and difficult to see or not visible in juveniles; anteromedial tubercles of head generally large, highly variable with size, often asymmetrical, becoming broad, flattened, and forward projecting in large specimens; coxae of pereonite 1 not notched laterally; body surface appearing rough, color brownish (Fig. 28C)	***Synidoteamagnifica***
–	Flange absent on basis of pereopods; anteromedial tubercles not as above, typically smaller, narrowly rounded or conical; lateral margins of pereonite 1 deeply notched in lateral view, separating coxal margins into upper and lower lobes; body surface generally smooth and whitish (Fig. 28D)	***Synidoteamedia***
19	Maxillipedal palp 4-segmented in adults, article 4 much larger than article 3 (Fig. 24B)^Endnote 23^	**20**
–	Maxillipedal palp 5-segmented in adults, article 5 much smaller than article 4 (article 5 may not be developed in juveniles) (Fig. 24C)	**23**
20	Eyes large, occupying almost entire lateral margins of head; dorsum of head with curved, sharply defined posterior groove; coxal plates of pereonites 2–7 all wide and extending entire length of respective pereonites; pleotelson broadest anteriorly, tapering to truncate posterior border or apex (Fig. 29B)	***Idoteametallica***
–	Eyes small, situated mid-laterally on head; coxal plates of pereonites narrow; pleotelson with concave or acuminate posterior border or apex	**21**
21	Posterior margin of pleotelson concave with rounded posterolateral corners; coxae 5–7 reach posterior edges of their respective pereonites (Fig. 29C)	***Idotearufescens***
–	Posterior margin of pleotelson acuminate, with or without distinct median projection; only coxae 6 and 7 or just 7 reach posterior edges of respective pereonites	**22**
22	Posterior margin of pleotelson triangular, converging to obtuse point; coxae 6 and 7 reach posterior edges of their pereonites; head with apically blunt frontal process (Fig. 29D)	***Idoteaurotoma***
–	Posterior margin of pleotelson with an elongate median projection; only coxae 7 reach posterior edge of pereonite 7; head with thin, pointed frontal process (Fig. 29A)	***Idoteafewkesi***
23	Posterior margin of pleotelson deeply concave with acute posterolateral corners (Fig. 30D)	***Pentidotearesecata***
–	Posterior margin of pleotelson not concave	**24**
24	Coxae visible dorsally on pereonites 5–7, and occasionally on pereonite 4; posterior margin of pleotelson convex, without median projection; body narrow, with head, pereon and pleotelson subequal in width; apex of frontal process with median notch (Fig. 30B)	***Pentidoteakirchanskii***
–	Coxae visible dorsally on pereonites 2–7; posterior margin of pleotelson with short (sometimes poorly developed) to elongate median projection; frontal process with or without apical notch	**25**
25	Pleonite 1 lateral margins curve and taper posteriorly to form narrow, acutely pointed posterolateral angles (margins not parallel)	**26**
–	Pleonite 1 lateral margins truncate or convex, more-or-less parallel, not curving posteriorly to form acute points	**27**
26	Pleotelson lateral margins concave, posterior margin with median projection; pereonites widely separated laterally, coxae 2–7 reaching posterior edges of pereonites, anterior margins of pereonite 1 separated from head; eyes rectangular or pyriform; apex of frontal process entire, without notch (Fig. 30E)	***Pentidoteaschmitti***
–	Pleotelson lateral margins convex, broadly rounded, posterior margin with small median projection; pereonites not widely separated laterally, only coxae 5–7 reach posterior edges of pereonites; anterior margins of pereonite 1 flush with head; eyes reniform; apex of frontal process entire, without notch (Fig. 30G)	***Pentidoteawosnesenskii***
27	Eyes elongated and thin dorsoventrally; maxilliped with 1–3 coupling hooks; coxae of pereonites 2–7 contiguous with each other (Fig. 30F)	***Pentidoteastenops***
–	Eyes not elongated; maxilliped with 1 coupling hook; coxae of at least some pereonites not contiguous with each other	**28**
28	Pleotelson posterior margin with long median projection; lateral margins of pereonite 1 convex; eyes circular to oval; apex of frontal process notched (Fig. 30A)	***Pentidoteaaculeata***
–	Pleotelson posterior margin with small or poorly developed median projection, sometimes appearing slightly truncate; lateral margins of pereonite 1 barely rounded, almost parallel; eyes with straight anterior and convex posterior borders; apex of frontal process entire, without notch (Fig. 30C)	***Pentidoteamontereyensis***

### ﻿Key I. Suborder Asellota, Superfamilies Janiroidea and Stenetrioidea

Figs 31–41

**Figure 31.**
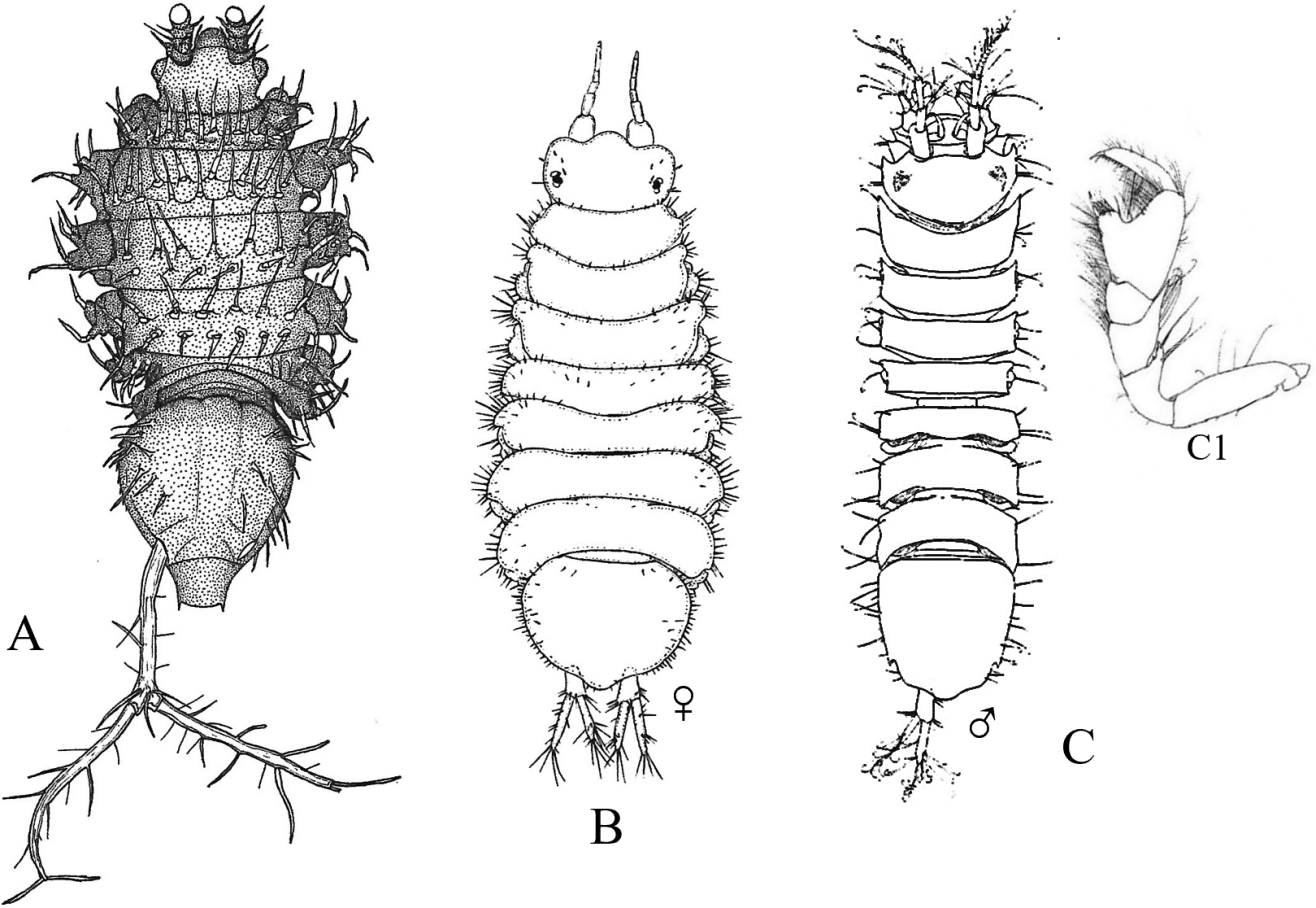
Isopoda, Asellota, Janiroidea, Dendrotionidae: **A***Acanthomunnatannerensis* (after Schultz 1966). Janiridae (in part): **B***Iaiscalifornica* (after Menzies and Barnard 1951). Stenetrioidea, Stenetriidae: **C***Stenetrium* sp. A **C1** pereopod 1 (after Wilson 1997).

**Figure 32.**
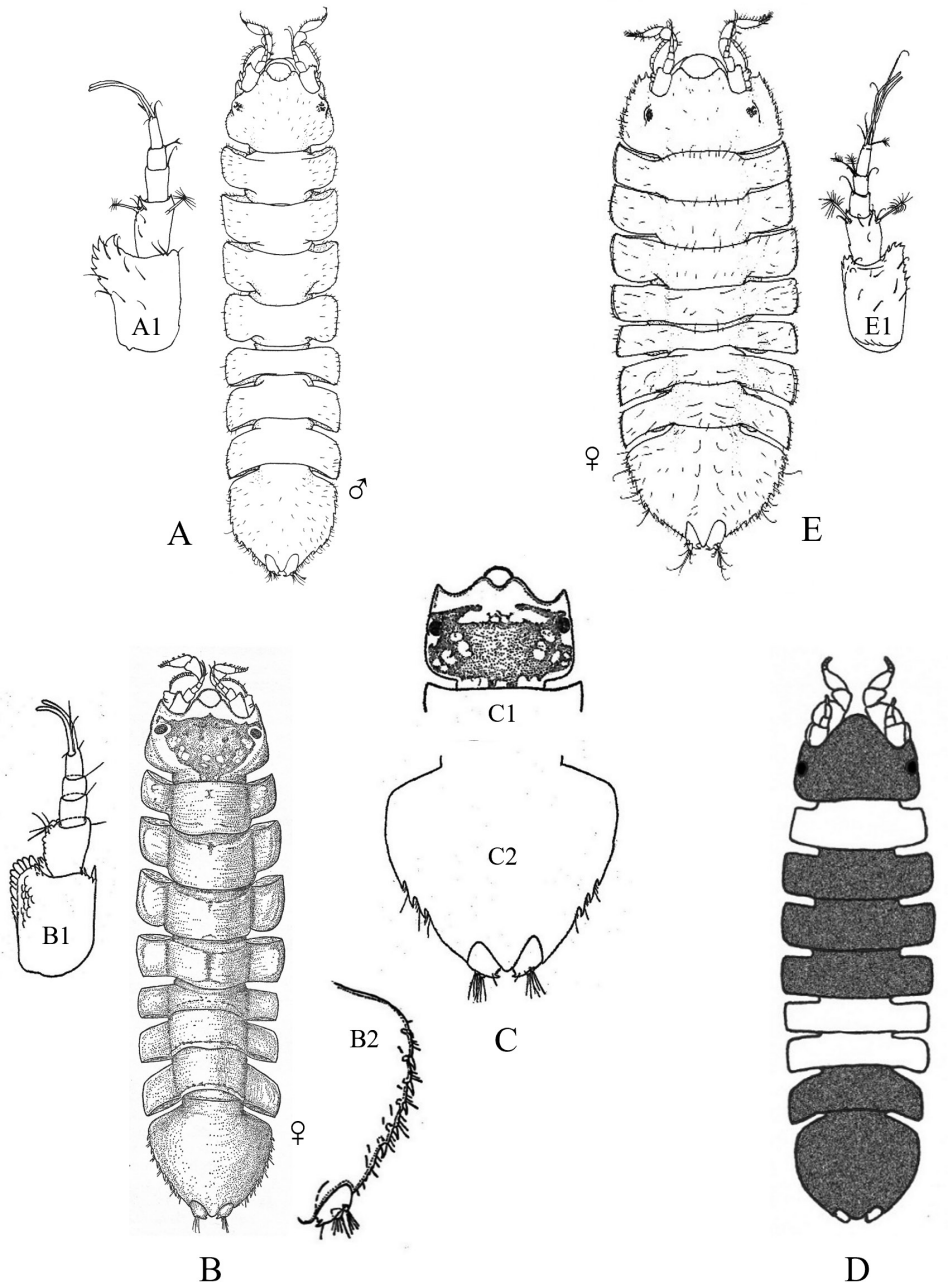
Isopoda, Asellota, Janiroidea, Joeropsididae: **A***Joeropsisconcava***A1** = antennule (after Wilson 1997) **B***Joeropsisdubiadubia***B1** antennule **B2** lateral margin of pleotelson (after Menzies 1951b) **C***Joeropsisdubiapaucispinis***C1** head **C2** pleotelson (after Menzies 1951b) **D***Joeropsislobata* (after Richardson 1905a) **E***Joeropsis* sp. A **E1** antennule (after Wilson 1997).

**Figure 33.**
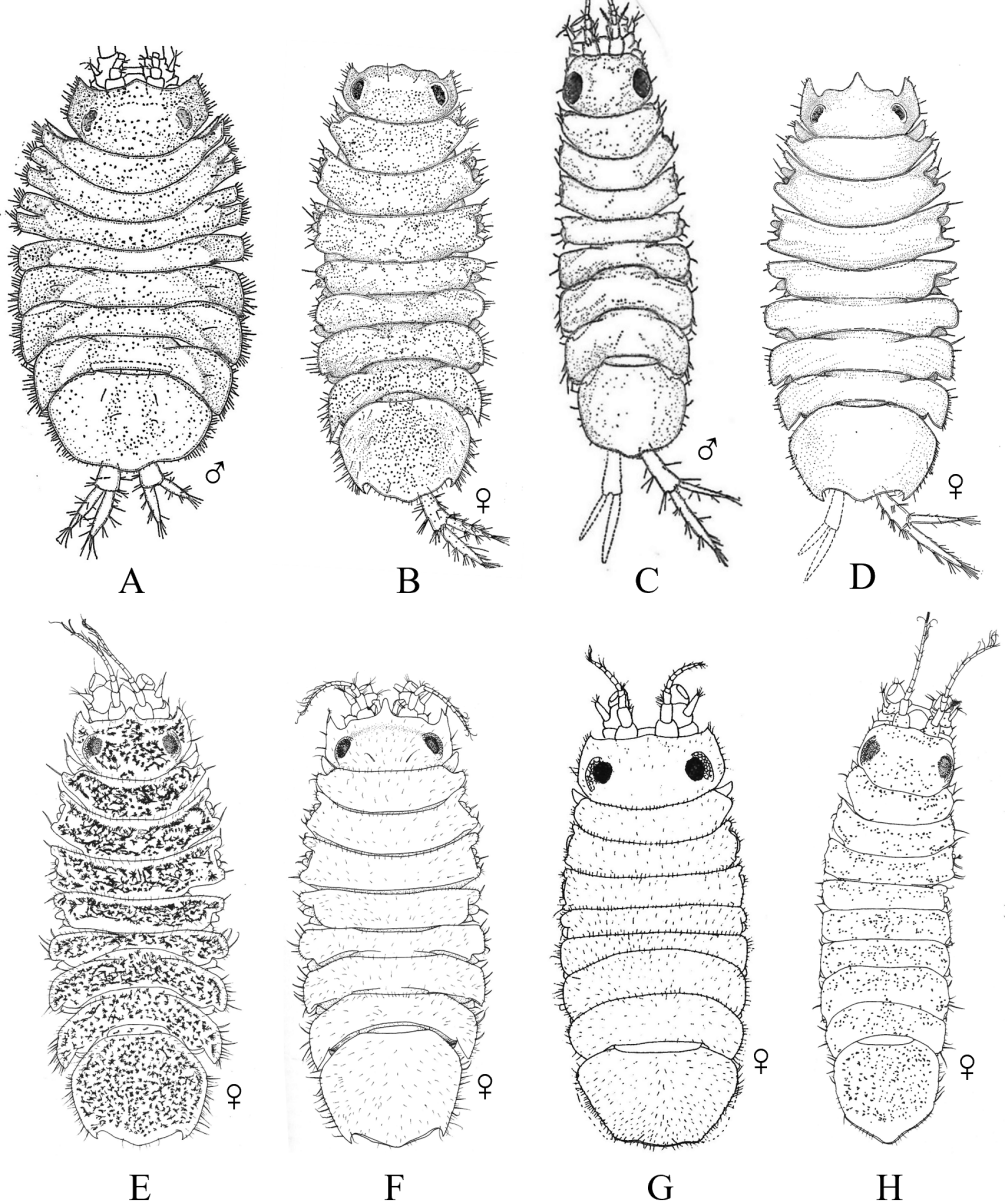
Isopoda, Asellota, Janiroidea, Janiridae (in part): **A***Janiralatadavisi* (after Menzies 1951b) **B***Janiralataoccidentalis* (after Menzies 1951b) **C***Janiralatarajata* (after Menzies 1951b) **D***Janiralatasolasteri* (after Menzies 1951b) **E***Janiralata* sp. A (after Wilson 1997) **F***Janiralata* sp. B (after Wilson 1997) **G***Janiralata* sp. C (after Wilson 1997) **H***Janiralata* sp. D (after Wilson 1997).

**Figure 34.**
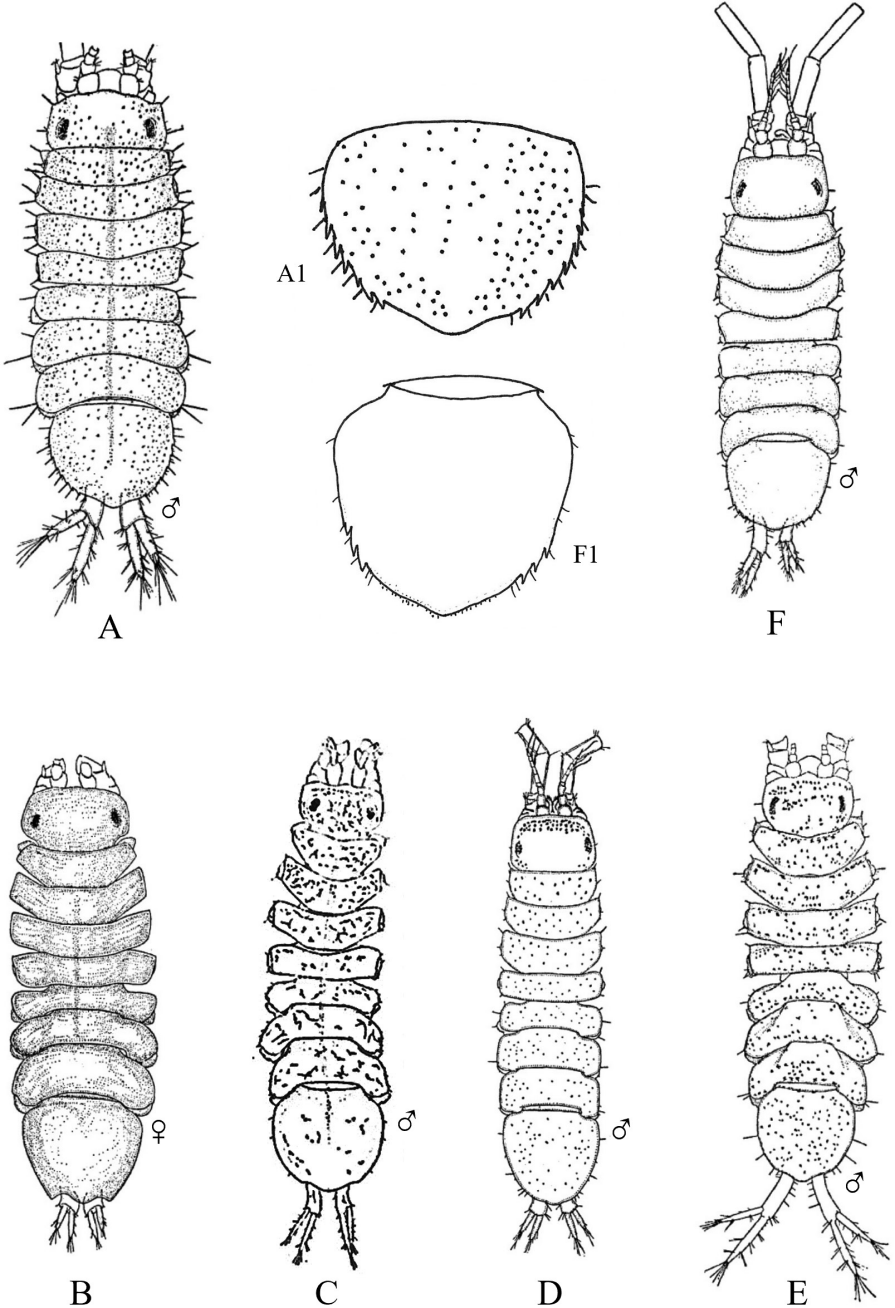
Isopoda, Asellota, Janiroidea, Janiridae (in part): **A***Ianiropsisanaloga***A1** enlarged dorsal view of pleotelson showing lateral spines (after Menzies 1952) **B***Ianiropsisderjugini* (after Menzies 1952) **C***Ianiropsiskincaidi* (after Menzies 1952) **D***Ianiropsisminuta* (after Menzies 1952) **E***Ianiropsismontereyensis* (after Menzies 1952) **F***Ianiropsistridens***F1** enlarged dorsal view of pleotelson showing lateral spines (after Menzies 1952).

**Figure 35.**
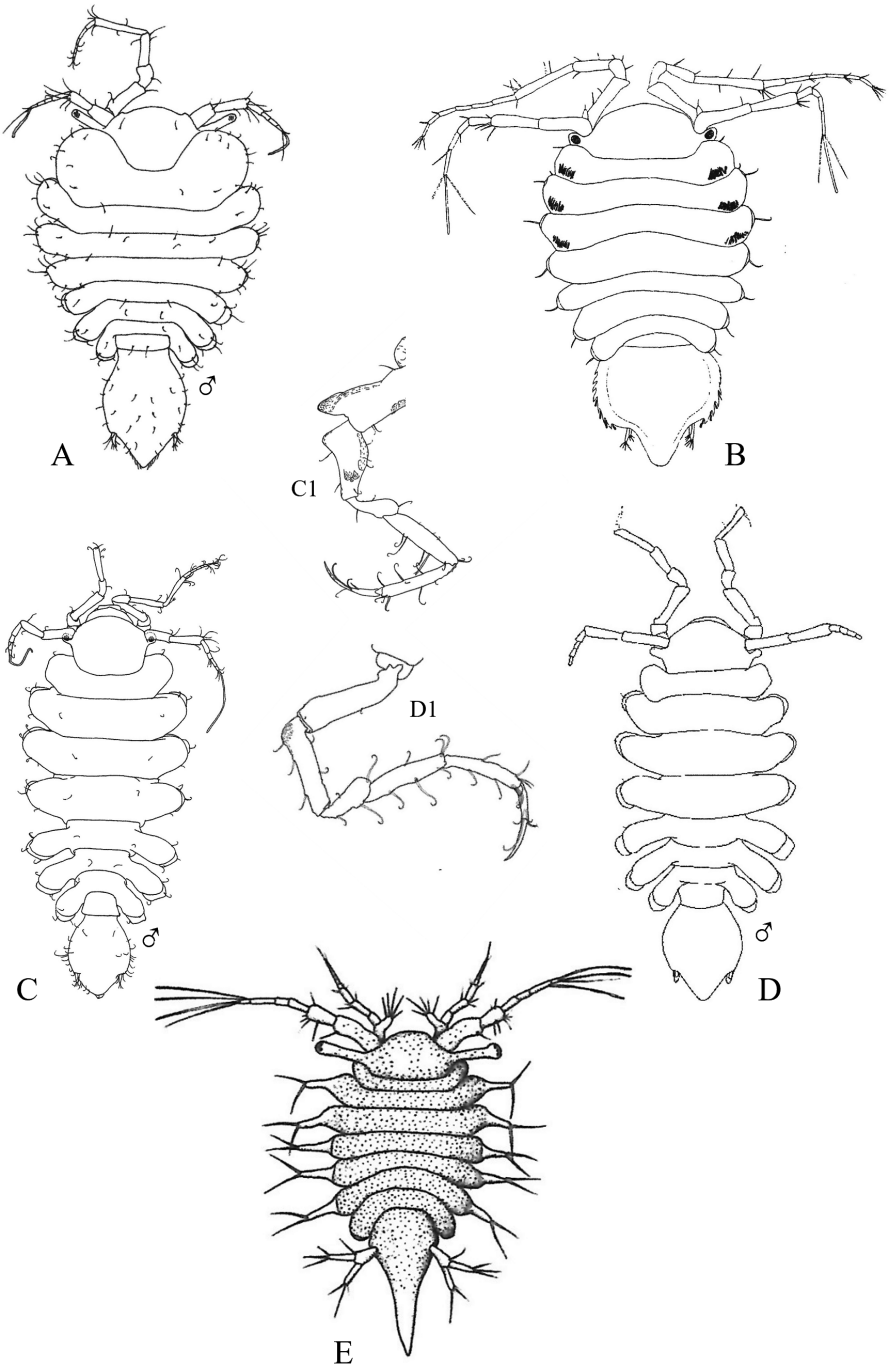
Isopoda, Asellota, Janiroidea, Paramunnidae (in part): **A***Austrosignumlatum* (after Just and Wilson (2021)**B***Boreosignum* sp. A (after Cadien 2008) **C***Munnogoniumerratum*, **C1** male pereopod 2 (after Wilson 1997) **D***Munnogoniumtillerae***D1** male pereopod 2 (after Just and Wilson (2021). Pleurocopidae: **E***Pleurocopefloridensis* (Caribbean species = representative for *Pleurocope* sp. A; after Kensley and Schotte 1989).

**Figure 36.**
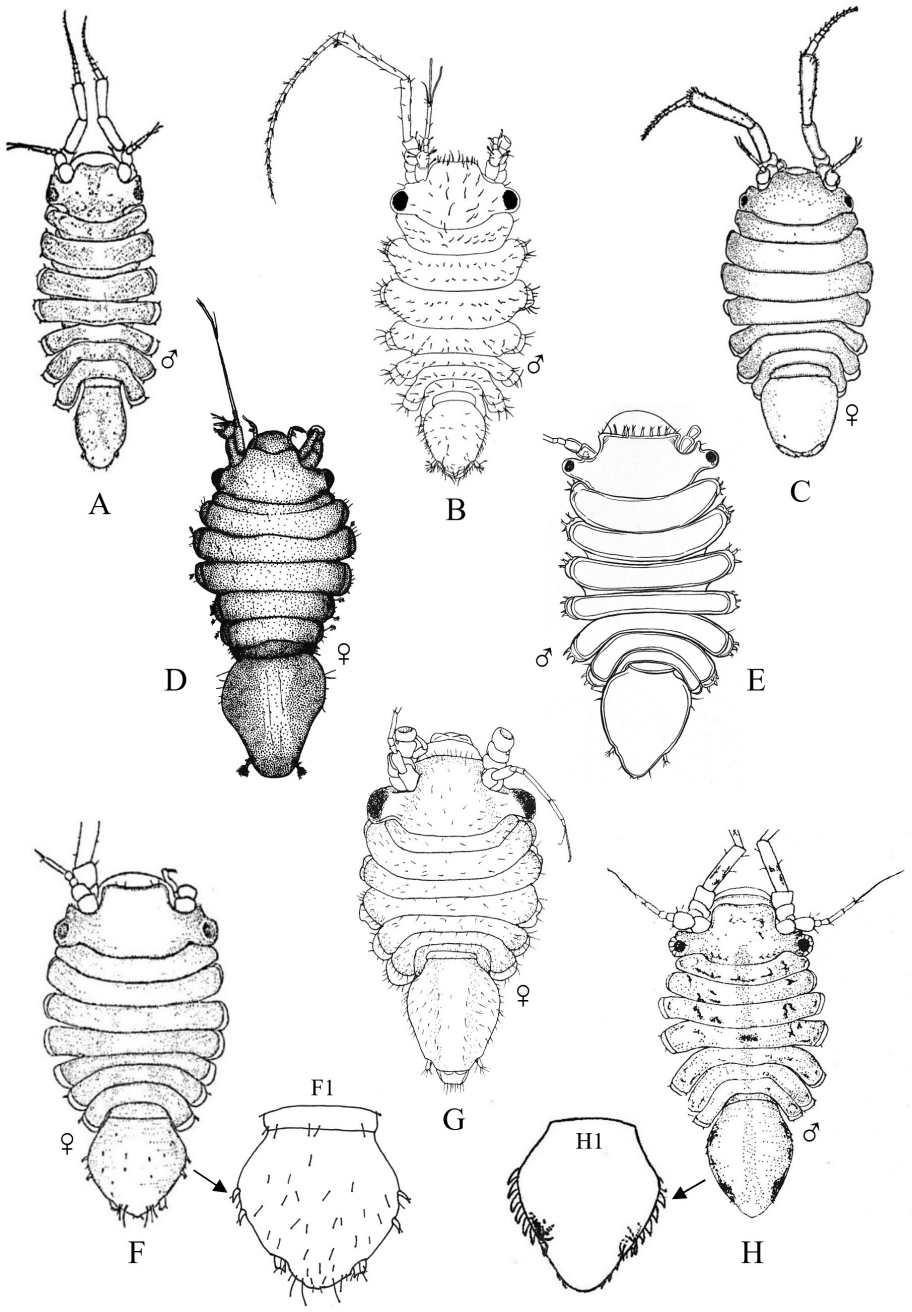
Isopoda, Asellota, Janiroidea, Munnidae: **A***Munnachromatocephala* (after Menzies 1952) **B***Munnafernaldi* (after George and Strömberg 1968) **C***Munnahalei* (after Menzies 1952) **D***Munnamagnifica* (after Schultz 1964) **E***Munnaspinifrons* (after Menzies and Barnard 1959) **F***Munnastephenseni***F1** close-up of pleotelson, arrow indicating sensory setae on left lateral margin (after Menzies 1952) **G***Munna* sp. A (after Wilson 1997) **H***Uromunnaubiquita***H1** close-up of pleotelson, arrow indicating spines or serrations on right lateral margin (after Menzies 1952).

**Figure 37.**
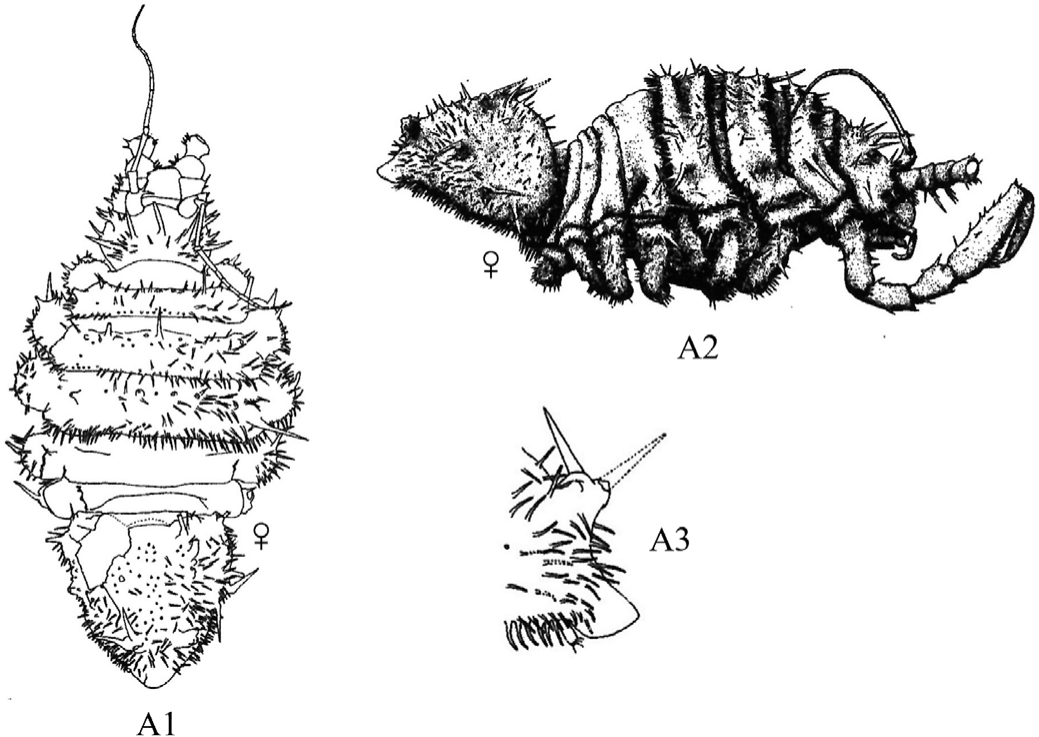
Isopoda, Asellota, Janiroidea, Haplomunnidae: **A***Haplomunnacaeca***A1** dorsal view of brooding female **A2** right lateral view of brooding female **A3** left lateral view of pleotelson tip and 3-spined tubercle (after Wilson 1976).

**Figure 38.**
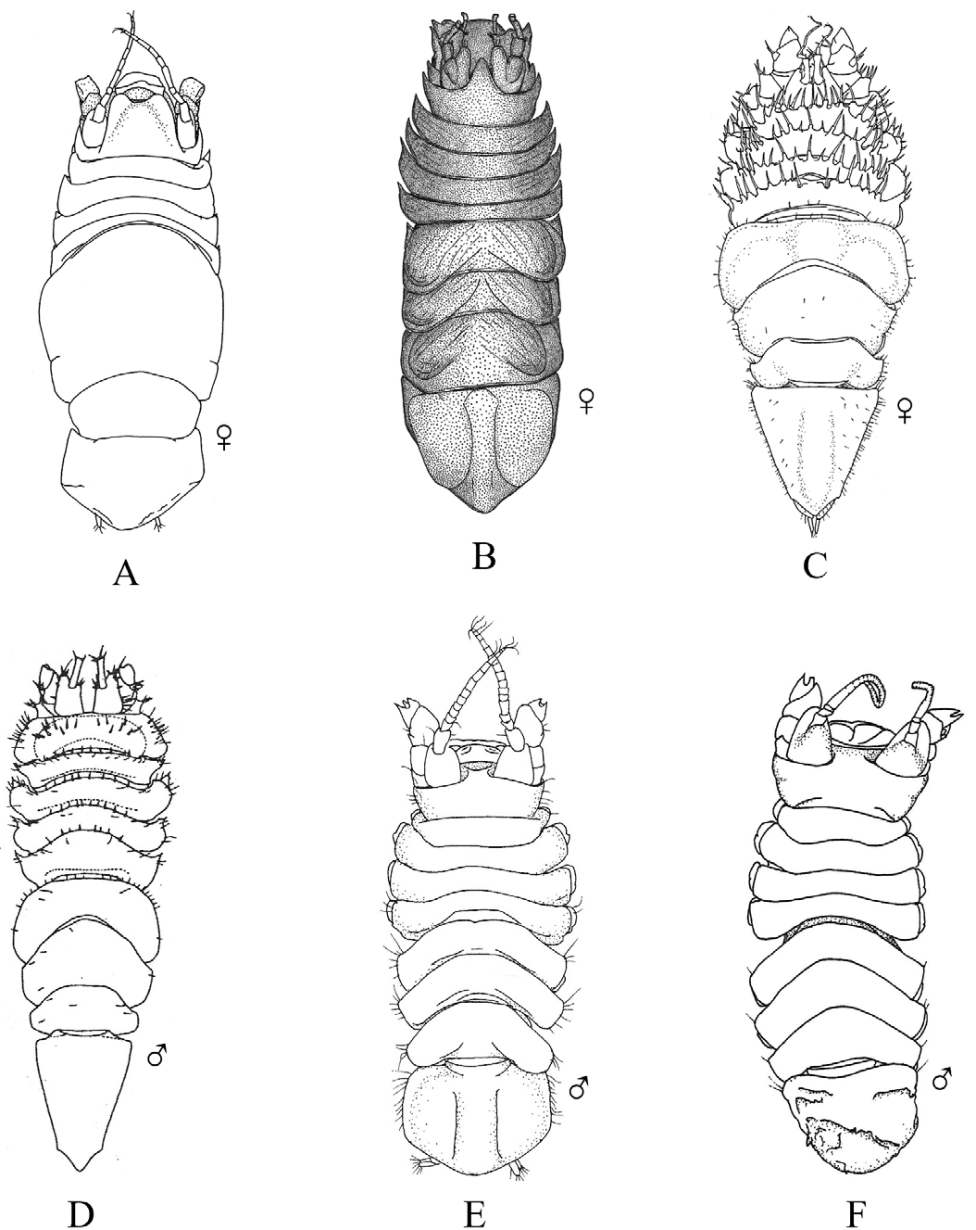
Isopoda, Asellota, Janiroidea, Munnopsidae: **A***Belonectes* sp. A (after Wilson 1997) **B***Eurycopecaliforniensis* (after Schultz 1966) **C***Ilyarachnaacarina* (after Thistle 1979) **D***Ilyarachnaprofunda* (after Thistle 1979) **E***Munnopsurus* sp. A (after Wilson 1997) **F***Munnopsurus* sp. B (after Wilson 1997).

**Figure 39.**
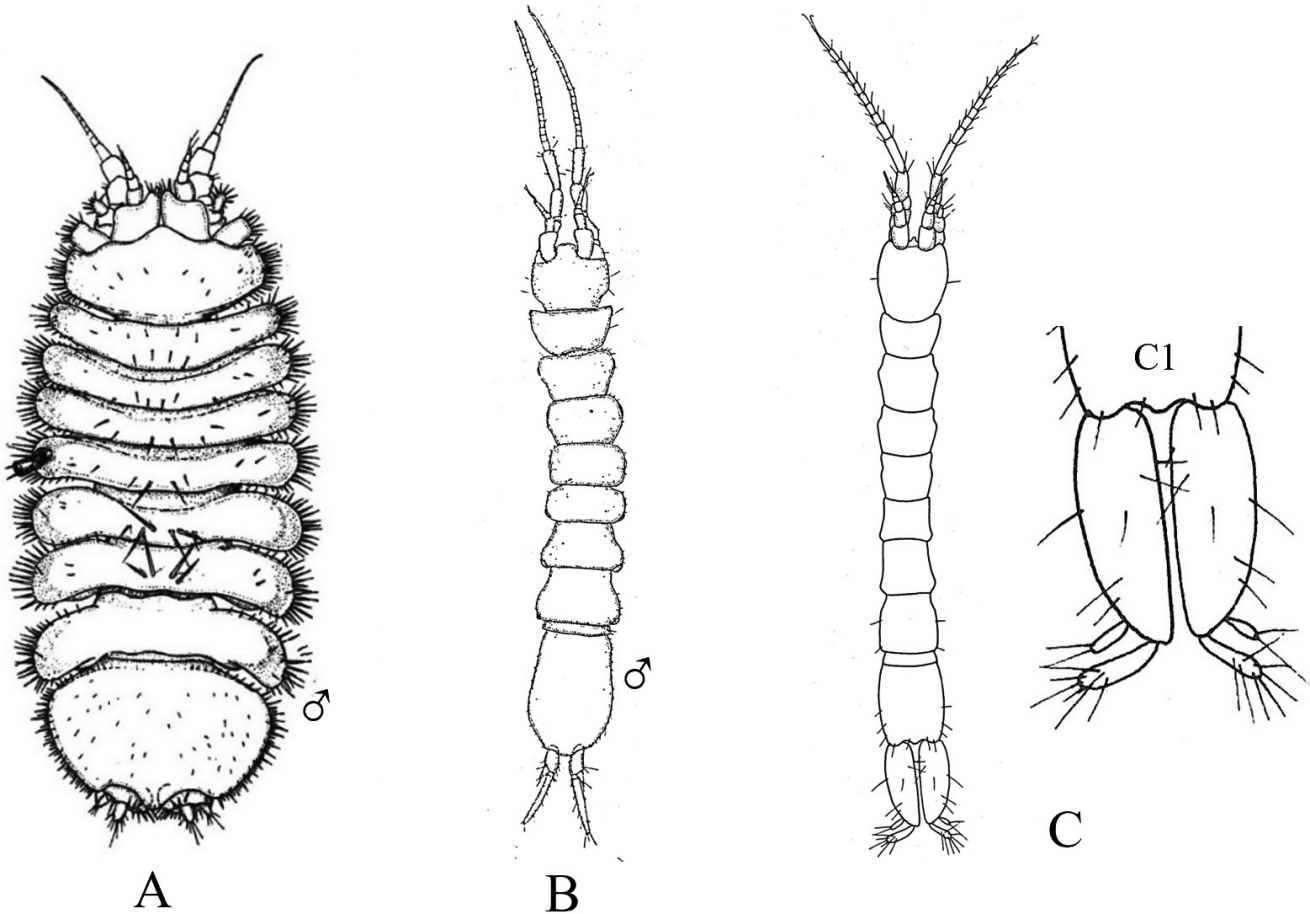
Isopoda, Asellota, Janiroidea, Janiridae (in part): **A***Caecijaerahorvathi* (after Menzies 1951c) **B***Caecianiropsispsammophila* (northern California species = representative for *Caecianiropsis* spp. LA1 and LA2; after Menzies and Pettit 1956). Lepidocharontidae: **C***Microcharonsabulum***C1** close-up of uropods (Caribbean species = representative for *Microcharon* sp. A; after Kensley 1984).

**Figure 40.**
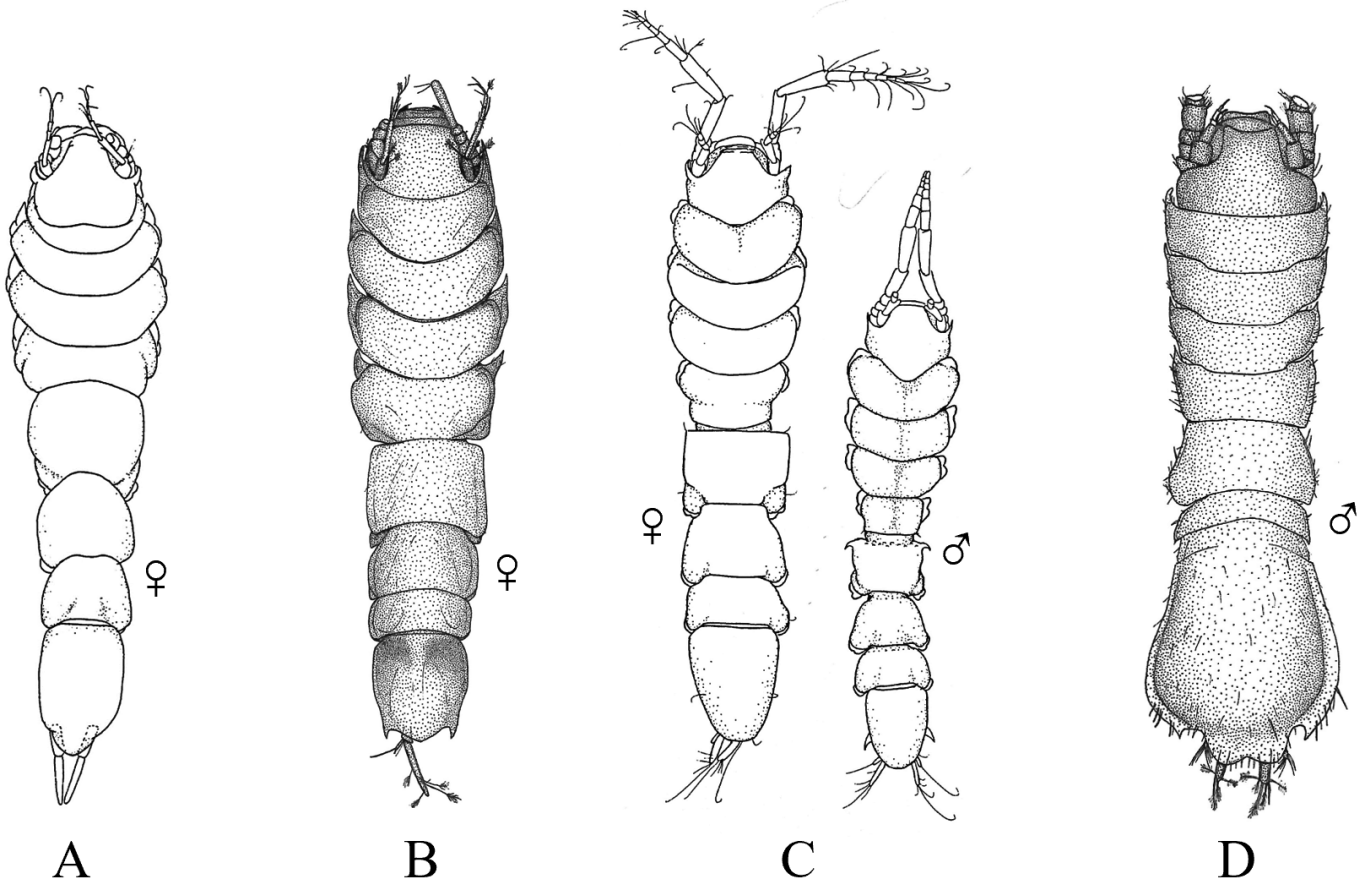
Isopoda, Asellota, Janiroidea, Desmosomatidae: **A***Desmosoma* sp. A (after Wilson 1997) **B***Momedossasymmetrica* (after Schultz 1966) **C***Prochelator* sp. A (after Wilson (1997); Nannoniscidae: **D***Nannonisconuslatipleonus* (after Schultz 1966).

**Figure 41.**
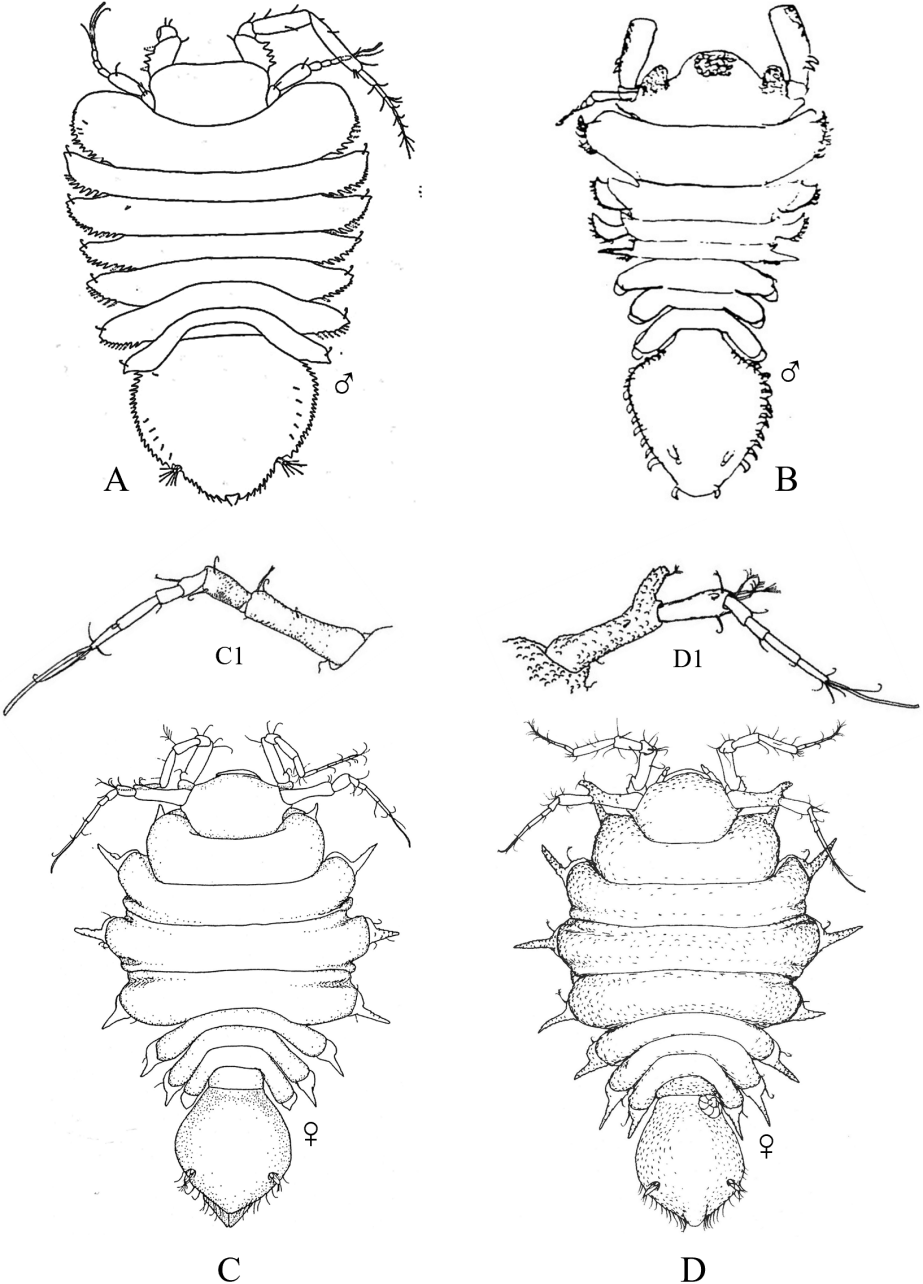
Isopoda, Asellota, Janiroidea, Paramunnidae (in part): **A***Paramunnaquadratifrons* (after Iverson and Wilson 1981) **B***Paramunna* sp. A (after Cadien 1996) **C***Pleurogoniumcaliforniense***C1** antennule (after Wilson 1997) **D***Pleurogonium* sp. A **D1** antennule (after Wilson 1997).

**Table:** 

1	Eyes present	**2**
–	Eyes absent	**36**
2	Body (except head) covered with large, jointed-appearing spines; uropods very long if not broken off, with basal segments and rami each ca. as long as width of pleotelson (Fig. 31A)	***Acanthomunnatannerensis*** [Dendrotionidae]
–	Body and uropods not as above	**3**
3	Eyes sessile, situated dorsolaterally on head but not reaching lateral margins; pleotelson subequal in width to cephalon and most pereonites	**4**
–	Eyes situated laterally on head, bulging outward on short to elongated ocular lobes or peduncle-like projections; pleotelson distinctly narrower than cephalon and widest pereonites	**24**
4	Head with rounded anterolateral margins and prominent rounded median lobe between antennae; body widest at ca. pereonite 6; adult body length < 3 mm; commensal on or in burrows of sphaeromatid *Sphaeromaquoianum* in bays and estuaries (Fig. 31B)	***Iaiscalifornica*** [Janiridae, in part]
–	Head not as above; body lateral margins parallel or gently convex and slightly widest around mid-pereon; maximum adult length varies between ~ 1.3–7.3 mm	**5**
5	Body elongated and unpigmented, lateral margins parallel and covered with long simple setae; head with acute anterolateral angles and lightly pigmented eyes; pleotelson shield-like with small posterolateral spines; male pereopod 1 strongly subchelate with thick projection on inferior margin of propodus, and many long simple setae on dactylus, propodus, carpus, and merus (Fig. 31C)	***Stenetrium* sp. A** [Stenetriidae]
–	Body, head, and male pereopod 1 not as above	**6**
6	Uropods short, inserted in subterminal excavations of pleotelson and not extending much beyond its posterior margin; both antennules and antennae small, flagella lacking or rudimentary; peduncle articles of antennae dilated	**7** [Joeropsididae]
–	Uropods well-developed, extending well beyond posterior margin of pleotelson; both antennules and antennae relatively long with multiarticulate flagella, although flagella often broken off in preserved specimens; peduncle articles of antennae not dilated	**11** [Janiridae, in part]
7	Body without pigment, covered with numerous fine simple setae	**8**
–	Body typically pigmented, not covered with fine setae	**9**
8	Body elongated and parallel-sided (L:W ratio ~ 4.3); head with distinct indentation on lateral margins below eyes; basal article of antennules with large, outwardly produced distolateral projection bearing ~ 6 sharp, flat spines curving toward article 2; pleotelson lateral margins with 7 large posteriorly curved spines, rest of body with small denticulations on lateral margins (Fig. 32A)	***Joeropsis* concava**
–	Body broad and laterally curved (L:W ratio ~ 2.6); margins of head produced forming large lateral plate, with ~ 9 anteriorly curved spines, eyes not reaching lateral margins; basal article of antennules with small distolateral process bearing several lateral curving flat spines; pleotelson with 8 posteriorly curved spines (Fig. 32E)	***Joeropsis* sp. A**
9	Lateral margins of pleotelson without spines; body banded in appearance, with dark brown pigment typically covering head, pereonites 2–4 and 7, and pleotelson; maxillipedal palp article 2 not produced medially; anterior margin of frontal lamina entire, without fringe of small scales (Fig. 32D)	***Joeropsislobata***
–	Lateral margins of pleotelson with 3–7 spines; body typically with diffuse pigment, which may be most pronounced on head and pereonite 4; maxillipedal palp article 2 produced medially; anterior margin of frontal lamina with fringe of small scales	**10**
10	Lateral margins of pleotelson with 5–7 spines; head typically heavily pigmented, pereon and pleon lightly pigmented or without pigments (Fig. 32B)	***Joeropsisdubiadubia***
–	Lateral margins of pleotelson with 2 or 3 spines; head and pereonite 4 both typically heavily pigmented but compare pigment pattern of head with that of *J.dubiadubia* above (Fig. 32C)	***Joeropsisdubiapaucispinis***
11	Pereopod 1 with conspicuous serrations or denticles on proximal third of inferior margin of propodus; maxillipedal palp articles 1–3 as wide as endite	**12**
–	Pereopod 1 without denticles on proximal third of inferior margin of propodus; maxillipedal palp articles 2 and 3 much wider than endite	**19**
12	Pleotelson with distinct distolateral angles or spines; head with pointed anterolateral margins and distinct rostrum	**13**
–	Pleotelson without distolateral spines, posterolateral margins rounded; head with pointed or rounded anterolateral margins, with or without rostrum	**16**
13	Adults with acutely pointed rostrum extending well beyond anterior limits of anterolateral margins of head; body completely devoid of pigment (Fig. 33F)	***Janiralata* sp. B**
–	Adults with rostrum not extending anteriorly beyond anterolateral margins of head; body pigmented	**14**
14	Head with acute rostral point; posterior margin of pleotelson concave laterally with convex median lobe that does not extend beyond posterior limits of distolateral spines; species lives commensally on sun stars (*Solaster* spp.), and its occurrence in southern California is questionable (Fig. 33D)	***Janiralatasolasteri***
–	Head with obtuse rostral point; posterior margin of pleotelson with convex lateral areas and obtusely rounded median point that extends posteriorly distinctly beyond limits of distolateral spines	**15**
15	Body covered with diffuse, anastomosing chromatophores, with pigment extending into region anterior to eyes (Fig. 33E)	***Janiralata* sp. A**
–	Body covered with dense, non-anastomosing chromatophores that are absent from region anterior to eyes (Fig. 33B)	***Janiralataoccidentalis***
16	Head with angular anterolateral margins and short, acute median lobe or slightly rounded median point; eyes medium to large, dorsal, clearly separated from lateral margins of head; body relatively short and broad (L:W ratio < 2.5); pleotelson nearly 2 × as wide as long	**17**
–	Head with rounded anterolateral margins, without distinct median lobe or rostrum; eyes large, almost touching lateral margins of head; body long and narrow (L:W ratio > 3.0); pleotelson ca. as wide as long	**18**
17	Dorsal surface with numerous black chromatophores; pereonites 1–3 with distinct lateral lobes or lappets, each pair separated by narrow V-shaped slit; all lateral margins with conspicuous setal fringe; posterolateral margins of pereonites 5–7 angular; pereonite 5 not shorter medially than other pereonites (Fig. 33A)	***Janiralatadavisi***
–	Dorsal surface without pigment, but with many fine setae; pereonites 2 and 3 with short anterior lappets and indistinct posterior lappets; lateral margins without conspicuous setal fringe; posterolateral margins of pereonites 5–7 rounded; pereonite 5 distinctly shorter medially than other pereonites (Fig. 33G)	***Janiralata* sp. C**
18	Body whitish, without distinct chromatophores; pereopod 1 propodus with ~ 12 or 13 denticles on proximoventral border (Fig. 33C)^Endnote 24^	***Janiralatarajata***
–	Body pigmented with numerous dense chromatophores; pereopod 1 propodus with ~ 18 denticles on proximoventral border (Fig. 33H)^Endnote 24^	***Janiralata* sp. D**
19	Lateral margins of pleotelson with spine-like serrations	**20**
–	Lateral margins of pleotelson without serrations, although setae may be present	**21**
20	Pleotelson with 4–7 spine-like serrations on each side (Fig. 34A)	***Ianiropsisanaloga***
–	Pleotelson with 3 spine-like serrations on each side (Fig. 34F)	***Ianiropsistridens***
21	Pleotelson with distinct posterolateral angles extended beyond uropodal insertions (Fig. 34B)	***Ianiropsisderjugini***
–	Pleotelson without posterolateral angles, posterior margins rounded	**22**
22	Uropods relatively short, not exceeding half-length of pleotelson; frontal margin of head truncate (Fig. 34D)	***Ianiropsisminuta***
–	Uropods much longer than half-length of pleotelson; frontal margin of head with short pointed or blunt medial lobe	**23**
23	Uropods very long, exceeding length of pleotelson; lateral apices of male pleopod 1 bifurcate (Fig. 34E)	***Ianiropsismontereyensis***
–	Uropods not exceeding length of pleotelson; lateral apices of male pleopod 1 not bifurcate (Fig. 34C)	***Ianiropsiskincaidi***
24	Pereonites 1–3, 5 and 6 with long, finger-like lateral processes, each with 1 or 2 terminal setae; pleotelson bulbous anteriorly, then tapers posteriorly to a narrow truncate end; uropods inserted dorsolaterally on anterior half of pleotelson, each uropod ca. as long as pleotelson; eyes tiny, each composed of two lenses at tip of very long and thin lateral peduncle or lobe (Fig. 35E)^Endnote 25^	***Pleurocope* sp. A** [Pleurocopidae]
–	Pereonites, pleotelson and uropods not as above; eyes situated at tips of short to elongated ocular lobes	**25**
25	Antennules and antennae both long, subequal in length; frontal margin of head broadly rounded	**26** [Paramunnidae, in part]
–	Antennae > 2 × longer than antennules; frontal margin of head broadly truncate, with or without medial concavity	**29** [Munnidae]
26	Pereonites 1–3 with dorsal posterolateral setal tufts; pleotelson as wide or wider than pereonite 7, lateral margins of pleotelson serrate or denticulate anterior to uropodal insertions, denticulations with embedded compound setae in cusps (Fig. 35B)	***Boreosignum* sp. A**
–	Pereonites without dorsal setal tufts; pleotelson narrower than pereonite 7, lateral margins convex without lateral serrations or denticulations	**27**
27	Body broadly ovate with pereonites 2 and 3 distinctly wider than pereonites 4 and 5 (body L:W ratio ~ 1.6); head deeply immersed in pereon with pereonite 1 much longer (male) or subequal (female) in length to pereonite 2; eyestalk (ocular lobe) much longer than article 1 of antennules; coxae visible in dorsal view only on pereonites 5–7 (Fig. 35A)	***Austrosignumlatum***
–	Body narrower than above with pereonites 2–5 subequal in width (body L:W ratio > 2.0); head not deeply immersed in pereon, with pereonite 1 shorter than pereonite 2 in both sexes; ocular lobes much shorter than article 1 of antennules; coxae visible in dorsal view on at least pereonites 2–7	**28**
28	Article 1 of antennules shorter than article 2; pereopod 2 in males with robust subtriangular projection on distoventral corner of basis, and proximal projection on ischium; pereopod 2 ventral margins of propodi with short robust setae (Fig. 35C)^Endnote 26^	***Munnogoniumerratum***
–	Article 1 of antennules subequal in length or longer than article 2; pereopod 2 in males not as above, without distinct projections on basis and ischium; pereopod 2 ventral margins of propodi with simple setae only (Fig. 35D)^Endnote 26^	***Munnogoniumtillerae***
29	Pleotelson with 4–6 spines or serrations on ventrolateral margins; male pleopod 1 apically pointed (Fig. 36H)	***Uromunnaubiquita***
–	Pleotelson without spines on lateral margins; male pleopod 1 with apices laterally expanded	**30**
30	Pleotelson with concave posterolateral margins; posterior border of pereonite 7 convex (Fig. 36D)	***Munnamagnifica***
–	Posterolateral margins of pleotelson not concave, although small indentations may be present around uropodal insertions; pereonite 7 with deeply concave posterior border wrapping around anterior portion of pleotelson	**31**
31	Uropods with large acute spine-like protuberances on distal margins; pleotelson posterior margin without subanal channel or shelf visible in dorsal view	**32**
–	Uropods without spine-like protuberances on distal margins; pleotelson with or without subanal shelf visible in dorsal view	**33**
32	Lateral margins of pleotelson with 2 or 3 backwards-curved robust sensory setae on each side; pleotelson broad medially, nearly as wide as long (Fig. 36F)	***Munnastephenseni***
–	Lateral margins of pleotelson without robust sensory setae; pleotelson narrow, much longer than wide (Fig. 36A)	***Munnachromatocephala***
33	Frontal margin of head with ~ 8 stout, 2-pointed spines; lateral margins of pereonites 1–6 with stout spines; pleotelson without subanal shelf (Fig. 36E)	***Munnaspinifrons***
–	Margins of head and pereonites without spines, although setae may be present; pleotelson with subanal shelf visible in dorsal view	**34**
34	Eyes small, occupying ca. half of short ocular lobes; body surfaces smooth, not covered with setae; uropods tiny, hidden within short, broad subanal shelf (Fig. 36C)	***Munnahalei***
–	Eyes large, occupying most of prominent ocular lobes; all dorsal surfaces covered with fine simple setae; uropods larger and clearly visible, not hidden by subanal shelf	**35**
35	Subanal shelf short and broad, reaching but not obscuring uropods; male pleopod 1 with large sensillate setae on ventral surface (Fig. 36B)	***Munnafernaldi***
–	Subanal shelf elongated posteriorly, but no wider than anus and not reaching or extending under uropods; male pleopod 1 with short simple setae on ventral surface (Fig. 36G)	***Munna* sp. A**
36	Surface of body covered with many small spines and fewer large spines; dorsal surface of pleotelson with pair of large stout posterior protuberances, each with 3 large distal spines (Fig. 37)	***Haplomunnacaeca*** [Haplomunnidae]
–	Surface of body and pleotelson not as above	**37**
37	Pereopods 5–7 expanded and natatory, with enlarged, paddle-like carpi and propodi; posterior body arranged as distinctive natasome, consisting of partially or completely fused pereonites 5–7 and the pleotelson	**38** [Munnopsidae]
–	Pereopods 5–7 without enlarged, paddle-like carpi and propodi; posterior part of body without distinct natasome	**43**
38	Head with bilobed or pointed rostrum projecting anteriorly between antennules	**39**
–	Head without rostrum extending between antennules	**40**
39	Head with broad, indented (bilobed) rostrum; natasome flattened, tapering; pereonites 5 and 6 fused dorsally; pereonite 7 smaller and narrower than pereonites 5 and 6 (Fig. 38A)	***Belonectes* sp. A**
–	Head with narrow, roundly pointed rostrum, not bilobed; natasome robust and deep; pereonites 5 and 6 not fused dorsally; pereonite 7 larger than pereonites 5 and 6 (Fig. 38B)	***Eurycopecaliforniensis***
40	Natasome not triangular, pleotelson inflated dorsally with relatively straight lateral margins; pereonites 5–7 subequal in size; uropodal protopod small, tubular	**41**
–	Natasome triangular, lateral margins of pleotelson taper to pointed apex; pereonite 7 distinctly narrower than pereonites 5 and 6; uropodal protopod large, flattened	**42**
41	Head subequal in width to pereonite 1; small species, with body length of adults < 2 mm (Fig. 38E)	***Munnopsurus* sp. A**
–	Head very large, distinctly broader than pereonite 1; much larger species than above, with body length of adults reaching 7 mm (Fig. 38F)	***Munnopsurus* sp. B**
42	Anterior margins of pereonites 1–4 with large pedestal setae (Fig. 38C)	***Ilyarachnaacarina***
–	Anterior margins of pereonites 1–4 with small, non-pedestal setae (Fig. 38D)	***Ilyarachnaprofunda***
43	Body with conspicuous setal fringe on lateral margins; head trilobate anteriorly; pleotelson wider than long with concave posterior margins at uropodal insertions and blunt median apex; basal article of antennules greatly expanded; article 3 of antennae with elongate, setiferous scale; small intertidal isopods that live in burrows in wood excavated by limnoriid isopods (Fig. 39A)	***Caecijaerahorvathi*** [Janiridae, in part]
–	Body and habitat not as above	**44**
44	Body greatly elongated, > 6 × longer than wide; lateral margins generally parallel with head, pereonal, and pleonal segments subequal in width	**45**
–	Body not as long as above, ≤ 4.5 × longer than wide; lateral margins distinctly not parallel	**47**
45	Antennae with strong scale on peduncular article 3; uropods long and distinct, protopod subequal in length to pleotelson and half as wide, sides parallel until pinching off slightly towards distal medial tip; uropodal endopod ca. one-third length of protopod and attached subapically; uropodal exopod ca. half-length of endopod and attached more laterally near where protopod begins to curve inwards (Fig. 39C)^Endnote 27^	***Microcharon* sp. A** [Lepidocharontidae]
–	Antennae without scale on peduncle; uropods styliform, not as above (Fig. 39B) [Janiridae, in part]	**46**
46	Integument of body with complete and prominent scaling; peduncular article 2 of antennules with ≥ 3 strong longitudinal ridges; mandibles whitish, not appearing sclerotized; setal comb present on posteroventral margin of basis of anterior pereopods^Endnote 28^	***Caecianiropsis* sp. LA1**
–	Body with very light and intermittent scaling; peduncle of antennules without longitudinal ridges; mandibles brownish, heavily sclerotized; setal comb absent on posteroventral margins of basis of anterior pereopods^Endnote 28^	***Caecianiropsis* sp. LA2**
47	Pereonite 7 fused dorsally with pleotelson; body widest at posterior third of pleotelson; posterior margins of pleotelson with large bilobed medial extension and large tooth on each lateral edge (Fig. 40D)	***Nannonisconuslatipleonus*** [Nannoniscidae]
–	All pereonites dorsally free; body widest at anterior half of pereon; pleotelson not as above	**48**
48	Body long and narrow (L:W ratio ~ 4.5); pereonites 1–3 only slightly wider than head and pleotelson	**49** [Desmosomatidae]
–	Body short and broad (L:W ratio ≤ 2.0); pereonite 1 and usually pereonites 2–6 much wider than head and pleotelson	**51** [Paramunnidae, in part]
49	Pereonite 1 distinctly larger than pereonite 2, with large ventral median spine; posteriorly curving ventral median spines also present on pereonites 3 and 7; pleotelson posteriorly rounded, with large subdistal ventrolateral spines (Fig. 40C)	***Prochelator* sp. A**
–	Pereonite 1 subequal or smaller than pereonite 2; ventral median spines absent on all pereonites	**50**
50	Pereonite 1 subequal to pereonite 2; head with anterolateral spines; pleotelson with posterolateral spines or angles and a rounded posterior medial border; uropods biramous (Fig. 40B)	***Momedossasymmetrica***
–	Pereonite 1 smaller than pereonite 2; head without anterolateral spines; pleotelson without posterolateral spines; uropods uniramous, lacking exopods (Fig. 40A)	***Desmosoma* sp. A**
51	Lateral margins of pleotelson serrate with numerous denticulations or spines; coxae of pereonites not visible dorsally	**52**
–	Lateral margins of pleotelson smooth, without denticulations or spines; coxae visible dorsally on pereonites 2–7, most or all with single large projecting spine	**54**
52	Anterior margin of head broadly quadrate with rounded anterolateral angles; lateral margins of pereonites heavily denticulate; pleotelson ca. as wide as long, lateral margins completely serrate; uropods inserted on posterolateral margins of pleotelson (Fig. 41A)	***Paramunnaquadratifrons***
–	Anterior margin of head with strongly produced and rounded anterior margin; lateral margins of pereonites with or without denticulations or spines; uropods inserted slightly dorsally on pleotelson	**53**
53	Head with 2 granulate tubercles above antennal bases; body widest at pereonite 1; lateral margins of pereonites serrate, anterior-most tooth enlarged and pointed forward on pereonites 1–3; pleotelson dentate along basal two-thirds of lateral margins, with 2 strong teeth located on rounded end where uropods would typically be inserted (Fig. 41B)	***Paramunna* sp. A**
–	Head without granulate tubercles; body widest at pereonites 2 and 3; lateral margins of pereonites without denticulations or spines; pleotelson with serrated lateral margins, although the details are undocumented^Endnote 29^	***Paramunna* sp. SD1**
54	Basal articles of antennules with large curved anterolateral projections; body surface rough with apparent microscales; projecting spine on each coxa of pereonite 7 typically large and conspicuous (Fig. 41D)	***Pleurogonium* sp. A**
–	Antennules without anterolateral projections on basal articles; surface of body smooth to slightly scaled; coxal spines of pereonite 7 reduced or absent (Fig. 41C)	***Pleurogoniumcaliforniense***

### ﻿Key J. Suborder Oniscidea, Superfamily Oniscoidea

Figs 42, 43

**Figure 42.**
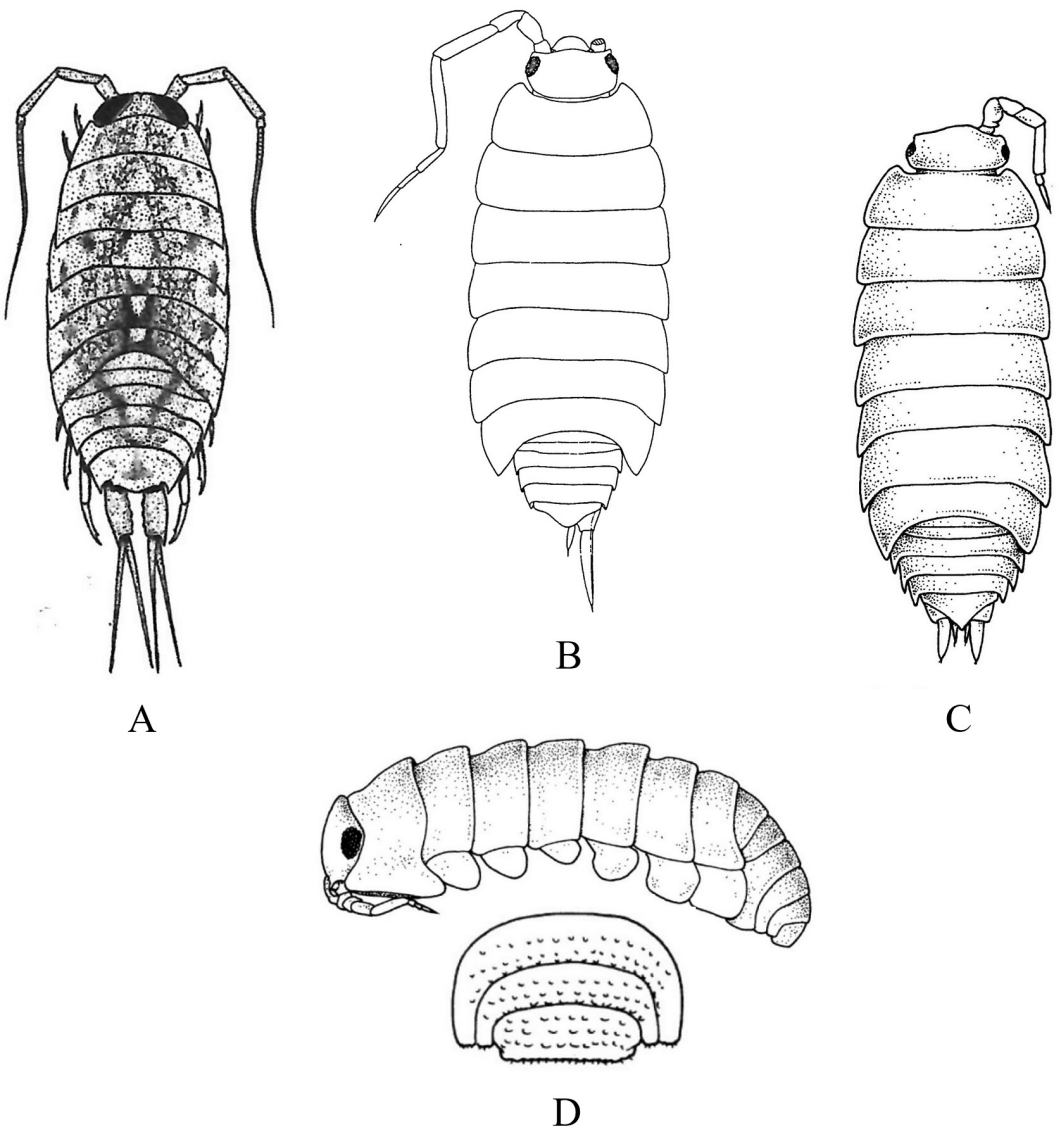
Isopoda, Oniscidea, Oniscoidea, Ligiidae: **A***Ligiaoccidentalis* (representative species for Ligiacf.occidentalis; after Allen 1976). Halophilosciidae: **B***Littorophilosciarichardsonae* (after Brusca et al. 2007). Platyarthridae: **C***Niambiacapensis* (after Brusca et al. 2007). Tylidae: **D***Tylospunctatus*, lateral view of whole body with posterior view of pleonites 4 and 5 plus pleotelson (after Brusca et al. 2007).

**Figure 43.**
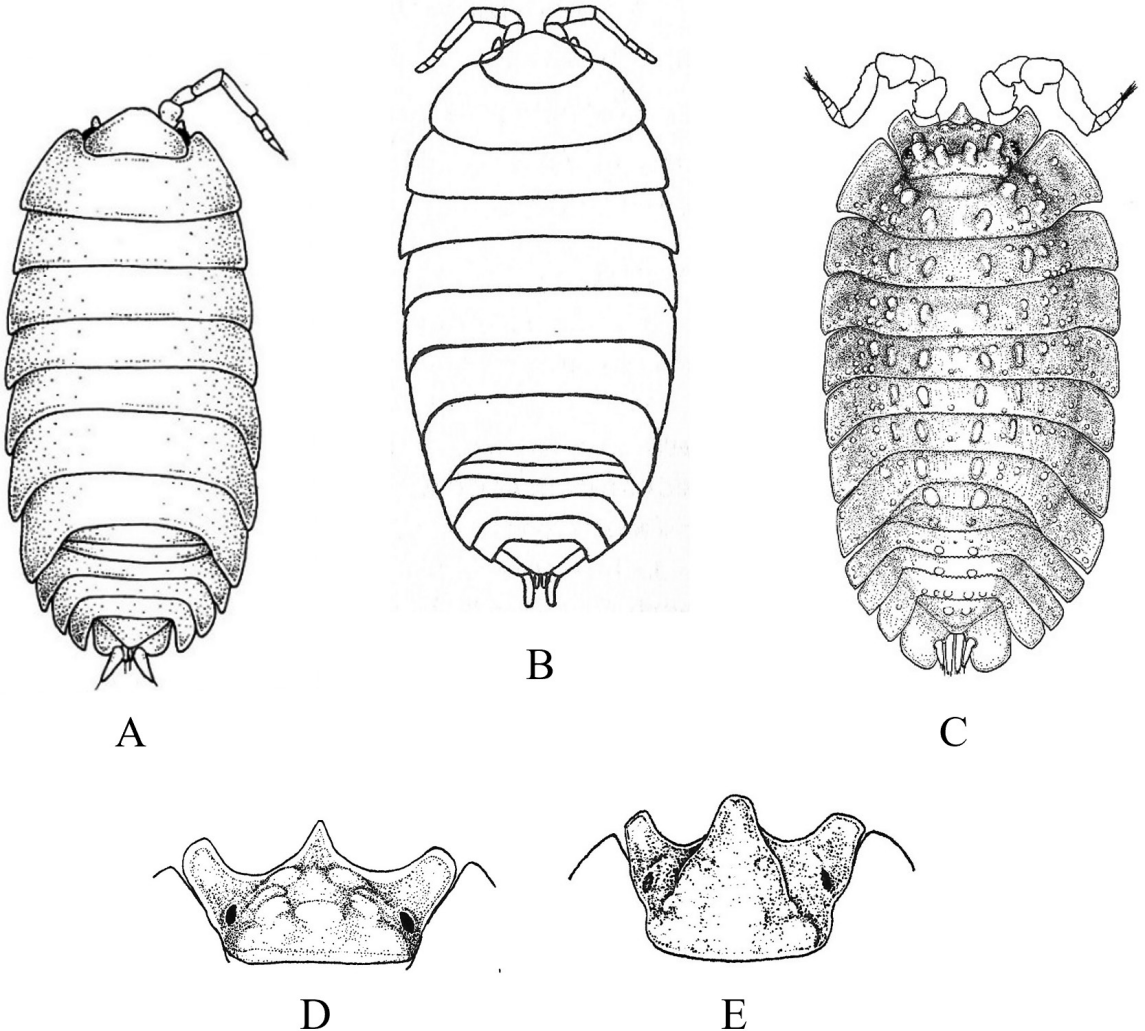
Isopoda, Oniscidea, Oniscoidea, Alloniscidae: **A***Alloniscusmirabilis* (after Brusca et al. 2007) **B***Alloniscusperconvexus* (after Richardson 1905a). Detonidae: **C***Armadilloniscuscoronacapitalis* (after Menzies 1950c) **D***Armadilloniscusholmesi* (after Menzies 1950c) **E***Armadilloniscuslindahli* (after Menzies 1950c).

**Table:** 

1	Uropods ventral and operculate covering pleopods, not visible in dorsal view; body able to roll up into a ball (conglobate) (Fig. 42D)	***Tylospunctatus***
–	Uropods terminal, visible in dorsal view; body may or may not be able to conglobate	**2**
2	Flagellum of antenna with > 10 articles; eyes large with > 50 ommatidia each, and wrapping around lateral margins of head, distance between eyes equal to length of 1 eye; isopods unable to conglobate (Fig. 42A)^Endnote 30^	**Ligiacf.occidentalis**
–	Flagellum of antenna with 2–4 articles; eyes not as above, much smaller (< 30 ommatidia) and not wrapping around lateral margins of head; isopods may or may not be able to conglobate	**3**
3	Flagellum of antenna with 2 or 3 articles; head without large anterolateral lobes	**4**
–	Flagellum of antenna with 4 articles; head with very large, distinctly produced anterolateral lobes, each lobe broad and truncate at tip	**7**
4	Flagellum of antenna with 2 articles; dorsal surface of body covered with fine, but distinct scales (Fig. 42C)	***Niambiacapensis***
–	Flagellum of antenna with 3 articles; dorsum without distinctly visible scales	**5**
5	Head with small, forward protruding cone-shaped lobes situated just anterior to eyes; frontal margin of head produced into blunt median lobe; pleon not abruptly narrower than pereon	**6**
–	Head without cone-shape lateral lobes anterior to eyes; frontal margin of head slightly sinuous, but not produced into median lobe; pleon abruptly narrower than pereon (Fig. 42B)	***Littorophilosciarichardsonae***
6	Uropodal protopod with rounded and expanded posterolateral margins (Fig. 43A)	***Alloniscusmirabilis***
–	Uropodal protopod with oblique posterolateral margins, not produced (Fig. 43B)	***Alloniscusperconvexus***
7	Anterior margin of head produced into strong, truncate median lobe; body markedly convex and capable of conglobation (Fig. 43E)	***Armadilloniscuslindahli***
–	Anterior margin of head produced into sharply acute median lobe; body not markedly convex and not capable of conglobation	**8**
8	Dorsal surface of body covered with large, elevated tubercles best developed on head; posterior border of all body segments except pleotelson appear beaded with minute, evenly spaced tubercles; antennal peduncle article 4 with spur-like process on lateral margin (Fig. 43C)	***Armadilloniscuscoronacapitalis***
–	Dorsal surface of body rough, with low, rounded tubercles; posterior border of all body segments smooth; antennal peduncle article 4 without spur-like process on lateral margin (Fig. 43D)	***Armadilloniscusholmesi***

### ﻿Endnotes

1. The provisional species Sphaeromatidae sp. A listed in SCAMIT (2021) was reported based on immature female specimens collected off Catalina Island at a depth of 12–16 m. Although these isopods co-occurred with two other sphaeromatids, *Dynamenellaglabra* and *Paracerceissculpta*, they differed from females of both species as well as other known members of those two genera (but see Endnote 17 below regarding the correct generic assignment for some SCB *Dynamenella*). Although the voucher sheet developed for Sphaeromatidae sp. A included a note that these isopods could be immature *Paracerceisgilliana*, this hypothesis has not been confirmed. Consequently, this provisional species is not included in Key G because its correct generic assignment is not known. The second species listed in SCAMIT (2021) not included in this review is the oniscid *Armadillidiumvulgare*, which is a strictly terrestrial species with a worldwide distribution.
2. No illustrations exist for the provisional anthuroid *Eisothistos* sp. A collected from Santa Barbara to Catalina Island at depths of 5–77 m, although Brusca and Cadien (1993) stated that the species is similar in gross morphology to *Eisothistosantarcticus* (see Wägele 1984a: fig. 2). However, neither *E.antarcticus* nor any of the other 30 species of *Eisothistos* currently recognized by WoRMS (Boyko et al. 2008 onwards) are known to occur near the SCB. For additional information on the genus see Wägele (1979, 1984a), Müller (1990), Poore (2001a), and Poore and Lew Ton (2002).
3. The aegid *Aegiochusplebeia* has not yet been reported from the SCB. However, the species is included in this guide since its known geographic range spans the region. See Brusca (1983a) and Bruce (2009a) for additional information.
4. It is currently unclear how to clearly distinguish juveniles of most cymothoid species from each other. Although Brusca (1978a, 1978b) provided detailed descriptions of the life histories of *Elthusavulgaris* (as *Lironecavulgaris*) and *Nerocilaacuminata* (as *N.californica*), such information is not readily available for other local cymothoid species. Consequently, all juvenile cymothoids are treated as unidentified Cymothoidae in Key C.
5. Since its description in 1980, the cymothoid *Renocilathresherorum* has been considered primarily a Gulf of California species with only a few known records from elsewhere in the Eastern Pacific (see Brusca 1981). These include one SCB record of a female and an associated juvenile collected off Corona del Mar in southern California plus a couple of records from Magdalena Bay on the southwest coast of Baja California. The species, however, has also been reported recently from the Suez Canal area of Egypt (e.g., Eman et al. 2014; Mahmoud et al. 2016; Ali and Aboyadak 2018). Given this disjunct distribution, it would be useful to perform a comprehensive morphological and molecular comparison of Eastern Pacific vs. Egyptian specimens to confirm whether they represent the same or perhaps two similar looking species of *Renocila*.
6. The cymothoid *Ceratothoagaudichaudii* is included in this guide based on historical distribution records (e.g., Brusca 1981). However, the species is listed as “taxon inquirendum” in WoRMS (Boyko et al. 2008 onwards) based on the review by Martin et al. (2015). Also see Hadfield et al. (2016) for a current list of accepted species in *Ceratothoa* Dana, 1852, and for information regarding taxonomic uncertainties within the genus.
7. Female and juvenile gnathiids key only to family in Key D. However, Haney (2006) provides a comprehensive discussion and guide to pairing unidentifiable females and juveniles with their male counterparts for many of the northeast Pacific species of *Gnathia* Leach, 1814 and *Caecognathia* Dollfus, 1901.
8. No illustrations exist for the three provisional species of gnathiids included in Key D (*Caecognathia* sp. A, *Caecognathia* sp. SD1, *Gnathia* sp. MBC1). However, photographs of these and all other known species of gnathiids that occur in SCB waters are available in Haney (2006).
9. The provisional species *Capitetragonia* sp. A was originally reported in 2011 as *Probopyria* sp. A (species voucher sheet available from SCAMIT Toolbox at https://www.scamit.org; T. D. Stebbins, 4 September 2011). However, the genus *Probopyria* Markham, 1985 was synonymized with *Capitetragonia* Pearse, 1953 by An et al. (2015). Because no illustrations exist for this provisional species, an image of the type species of *Capitetragonia*, *C.alphei* (Richardson, 1900), is provided as an example in Fig. 15B.
10. The bopyrid *Eremitionegiardi* is listed in SCAMIT (2021) as *Pseudionegiardi*, although this and eight other species previously included in *Pseudione* were transferred to the newly erected genus *Eremitione* by Williams et al. (2019). This species was originally added to the SCAMIT species list in 2011 (Edition 6) based on a trawl record from off the coast of Palos Verdes (D. Cadien, pers. comm.). However, the record may be questionable because *E.giardi* was not previously known from California waters (see Markham 1974b) except possibly for a report of an unconfirmed species identified as *Pseudione* sp. found infesting the hermit crab *Pagurushirsutiusculus* in central California (J. Markham, pers. comm.). The record of *E.giardi* off Palos Verdes is likely valid if the host was a hermit crab, but not if the host was another type of crustacean (J. Markham, pers. comm.).
11. Two different adult body morphologies have been described for the dajid isopod *Holophryxusalaskensis* (see Richardson 1905a, 1905b: Butler 1964; Boyko and Williams 2021b), while Coyle and Mueller (1981) provided complete descriptions of the larval and juvenile stages of the species. In the original species description based on three adult female specimens collected off Alaska without any host information, Richardson (1905b) described and illustrated a stout and somewhat irregular body form, the dorsal and ventral views which are reproduced in Fig. 16A herein. Richardson (1908) later described a second species of the genus, *H.californiensis*, based on one adult female collected off Alaska attached to the shrimp *Pasiphaeapacifica*, plus one female and one male collected off Santa Barbara, southern California. In his subsequent redescription of *H.alaskensis* based on Richardson’s type material and additional specimens of both sexes from British Columbia, Butler (1964: figs 1, 3) synonymized *H.californiensis* with *H.alaskensis* and described a more typical elongate and symmetrical body form for adult females. Additionally, Boyko and Williams (2021b) recently provided a thorough review of the above with new records that extended the southern distribution of *H.alaskensis* to deep waters off the US-Mexico border. These authors also provided color photographs of typical female and male *H.alaskensis*, as well as an in-situ photo of an adult female attached to its host shrimp (Boyko and Williams 2021b: fig. 1).
12. Specimens of unidentified dajid isopods listed as *Zonophryxus* sp. in SCAMIT (2021) were collected in SCB waters during the 1990s parasitizing two species of caridean shrimp, *Pantomusaffinis* and *Plesionikatrispinus* (see Montagne and Cadien 2001; Boyko and Williams 2021a). Whether these isopods represent one or more species awaits further investigation. Because no illustrations exist for *Zonophryxus* sp., images of a recently described species from Peruvian waters, *Zonophryxusprobisowa* Boyko & Williams, 2021, are provided in Fig. 16B as an example of the genus. Although it is not clear how closely this species resembles the SCB specimens, these images clearly show the notched appearance of the posterior end that is characteristic of species of *Zonophryxus*. In addition to describing *Z.probisowa*, Boyko and Williams (2021a) reviewed and provided a key to all seven species of *Zonophryxus* based on female characters. Finally, a couple of other eastern Pacific specimens of *Zonophryxus* recently discovered from mis-identified Gulf of California samples collected during the Talud VIII cruises in 2005 (TDS, pers. obs.) may represent an additional undescribed species of this genus (C. Boyko, pers. comm.).
13. There may be a new species of *Heteroserolis* in deeper waters of the SCB. Preliminary comparisons of specimens collected from depths > 100 m off San Diego with those from shallower waters in San Diego Bay (depth ≤ 15 m) reveal several differences in morphology. Whether these differences merit a new species requires further investigation. The differences between the shallow-water and deep-water specimens include:

- Deep-water specimens have a much larger dorsal carina than do shallow-water animals. This is like differences described by Hessler (1972) between *H.mgrayi* (like deep-water specimens) and *H.carinata* (like San Diego Bay specimens).
- Deep-water specimens have a rather shallow lateral notch on the pleotelson that does not form a well-defined tooth (like described for *H.tropica* and *H.mgrayi*), while shallow-water animals have the distinct tooth or deep notch characteristic of *H.carinata*.
- Pereonites 5 and 6 are subequal in width to pereonite 4 in the deep-water animals (the lateral margins are nearly continuous with a gradual tapering to the pleotelson). In contrast, there is a distinct narrowing of the pereon between pereonites 4 and 5 in the shallow-water animals. This difference in shape appears distinct even in juveniles.

1. Two species of the sphaeromatoid genus *Ancinus* H. Milne Edwards, 1840 are reported herein as occurring in the SCB, including *A.granulatus* Holmes & Gay, 1909 and *A.seticomvus* Trask, 1971. Although Schultz (1973) considered *A.seticomvus* a junior synonym of *A.granulatus* as it has been listed by SCAMIT (1998–2021) and Brusca et al. (2007), Glynn and Glynn (1974) disputed Schultz’s assessment and provided a detailed suite of characters by which to distinguish the two species. Consequently, most subsequent workers have recognized *A.seticomvus* as a valid species (e.g., Dexter 1976; Iverson 1982; Pires 1987; McLaughlin et al. 2005; Rocha-Ramírez et al. 2010) as currently accepted by WoRMS (i.e., Boyko et al. 2008 onwards).
2. Although the sphaeromatid *Gnorimosphaeromaoregonense* is included in the SCAMIT (2021) list of SCB species, Wetzer et al. (2021), in their recent redescription and assessment of the species and genus, could only verify its range from Alaska to San Francisco, California. Thus, the presence of *G. oregonense* south of central California is questionable, and any specimens encountered should be carefully examined. While *G. oregonense* is a fully marine species, the other species of the genus in SCB waters included in this review, *G. noblei*, occurs in brackish or freshwater habitats. In addition to habitat differences, these two species can best be distinguished by the shape of their coxal plates as indicated in the key. See Wetzer et al. (2021: fig. 15) for SEM comparisons of the coxae of *G. oregonense* and *G. noblei*. Finally, although *Gnorimosphaeromainsulare* (Van Name, 1940) has been reported from the SCB, it is not included in this guide since it is only known from a freshwater pond on San Nicolas Island and is therefore not a marine species (see Wetzer et al. 2021 for additional details).
3. Even though the couplet in Key G identifying the sphaeromatid *Paracerceissculpta* distinguishes between male and female characteristics, it does not account for differences between the three discrete male morphs of the species described in Shuster (1987). The male traits specified in the key represent those of the larger and more elaborate alpha-males that attract females to the spongocoels of calcareous sponges where mating and brooding of young occurs. Alpha-males are characterized by elongated uropods and highly sculptured pleotelsons as indicated in the key. Smaller beta-males lack such ornamentation and resemble females of *P.sculpta*, while tiny gamma-males resemble immature juveniles. All three male morphs, however, possess well-developed secondary sex characters (penes and appendix masculina) and are sexually mature. See Shuster (1987, 1992), Shuster and Wade (1991a, 1991b), and Shuster and Guthrie (1999) for additional information on the reproductive biology and ecology of *P.sculpta*, and the relationship between alpha, beta, and gamma males.
4. The sphaeromatids *Dynamenelladilatata* and *Dynamenellaglabra* as treated herein and in SCAMIT (2021) are instead classified in the genus *Dynamene* Leach, 1814 in both WoRMS (Boyko et al. 2008 onwards) and Wall et al. (2015), although listed as “taxon inquirendum” or “correct generic placement uncertain,” respectively. Thus, the correct generic assignment for these two species is currently in question. Based on molecular data, Wetzer et al. (2018) demonstrated that *Dynamene* and *Dynamenella* are not closely related (i.e., occurring in *Cerceis* and *Dynamenella* clades, respectively). Morphological revisions based on adult males are needed to be able to correctly place these species.
5. Species of the sphaeromatid genus *Exosphaeroma* Stebbing, 1900 have either a 1:1 male to female ratio in which males are observed to mate guard an individual female or a highly skewed sex ratio where adult males guard harems and are very rare relative to females and subadults. In the SCB, *E.inornata* exhibits the first of these mating patterns with individual male/female pairs. Both sexes in this species are similar in general appearance, are unornamented, and have uropodal endopods in which the posterior margin is evenly rounded. Alternatively, in the *E.amplicauda*-like species (*E.amplicauda*, *E.aphrodita*, and *E.pentcheffi*) treated herein in which males guard harems of females, the adult males are characterized by a pleotelson to overall body length ratio of 0.30 or greater, a pleotelson with an acuminate posterior border, and uropodal endopods in which the posterior margins are falcate. A review of *Exosphaeroma* from the north-eastern Pacific evaluated the status of *E.amplicauda* and *E.aphrodita* and described an additional three new species (Wall et al. 2015). A neotype of *E.amplicauda* was designated and the species redescribed based on the neotype and topotypic specimens. As a result of this study, the distribution of *E.amplicauda* was restricted to northern California along the coasts of Marin, Sonoma, and San Mateo Counties. In contrast, *E.aphrodita* originally described from La Jolla (San Diego County) and previously considered a *nomen dubium* was taken out of synonymy and revalidated by Wall et al. (2015) as a SCB species. Additionally, the new species *E.pentcheffi* was described from intertidal samples collected at Point Fermin (Los Angeles County). These authors are also aware that there are likely other undescribed species of *Exosphaeroma* occurring in the Channel Islands within the SCB, with specimens from Santa Catalina Island being genetically closely related to *E.pentcheffi* (RW, pers. obs.). The *E.amplicauda*-like species along the western coast of North America have been previously lumped and unrecognized, in part because original descriptions were not detailed and the best drawings available were those of Kussakin (1979). However, Kussakin’s material was based on specimens collected from Amchitka Island, Alaska, some 2000 km north of the type locality of *E.amplicauda* near San Francisco. Consequently, Kussakin’s specimens probably also represent an undescribed species.
6. Even though the sphaeromatid *Exosphaeromarhomburum* is an accepted species in WoRMS (Boyko et al. 2008 onwards) and is reported in SCAMIT (2021), Wall et al. (2015) designated the species incertae sedis in their recent treatment of north-eastern Pacific *Exosphaeroma*. This was due in part to the brief original description provided by Richardson (1899: as *Sphaeromarhomburum*), in which she only described and figured the pleon, pleotelson, and uropods in any detail. Although a figure of the maxilliped was also presented, Richardson (1899) provided no accompanying description of the structure. Richardson (1899) also did not specify the sex of either of the two specimens she examined, both of which were collected in central California (Monterey Bay). Because of the above and the lack of confirmed males known to them at the time, Wall et al. (2015) were unable to further evaluate the status of the species, thus resulting in their incertae sedis designation. As part of the present study, however, we were able to examine specimens of both male and female isopods attributed to *E.rhomburum* and confirm its validity as a distinct species without any significant sexual dimorphism. On the other hand, we question whether the species actually belongs in *Exosphaeroma* because it appears to differ noticeably in morphology from other species of the genus. In her original description and subsequent work, Richardson (1899, 1905a) also noted that the species was “similar to *S.egregium* Chilton” (= *Sphaeromaegregia* Chilton, 1892), a species now placed in the southern hemisphere genus *Cymodocella* Pfeffer, 1887 (see Harrison and Holdich 1982; Bruce 1995). Based on a cursory review of some *Cymodocella* specimens available to us, we agree that *E.rhomburum* appears to resemble that genus more closely than it does *Exosphaeroma*. However, it will require further morphological and preferably molecular studies to confirm whether the species should be reassigned to *Cymodocella* or another existing or perhaps new genus. Finally, we extend the southern distribution of *E.rhomburum* to NW Baja California based on our examination of specimens collected by the City of San Diego’s Ocean Monitoring Program, while the northern range limit is extended herein to Washington State, USA based on observations reported by Lafferty and Suchanek (2016).
7. Three species of the valviferan genus *Idarcturus* Barnard, 1914 are reported herein as occurring in the SCB. Of these, *I.allelomorphus* is the most common, occurring in soft-bottom continental shelf sediments at depths < 100 m from central California to Baja California Norte (see Menzies and Barnard 1959; Wetzer and Brusca 1997; Campos and Villarreal 2008). A second species, *I.hedgpethi*, was long known from only northern California where it was collected in 1948 living on hydroids in the low intertidal (see Menzies 1951b). Although *I.hedgpethi* was added to the SCAMIT species list in 2008 (Edition 5), we have been unable to confirm the source of that record. One record of *I.hedgpethi* was reported from Port Hueneme (Ventura County, southern California) during the 2000 and 2001 surveys of California bays and harbors (Fairey et al. 2002), but it is unknown whether that record was the source of the listing in SCAMIT (2008). The species was also reported from much further north in the Strait of Georgia, British Columbia by Macdonald et al. (2010). If accurate, these last two records expand the geographic range of *I.hedgpethi* from the northern California type locality to span ~ 1700 km from British Columbia to the SCB. Although *I.hedgpethi* was also recently reported from soft-bottom benthic sites located near the northern Channel Islands during the Bight 2013 regional monitoring project (Gillett et al. 2017), those specimens likely represent an undescribed species of *Idarcturus* that was first observed in the region during the previous Bight 2008 regional project (TDS, pers. obs.), but which was lumped together with *I.allelomorphus* in the project’s final report (i.e., Ranasinghe et al. 2012). Although the Bight 2008 specimens were initially thought to resemble *I.hedgpethi* in terms of their pronounced spination pattern, subsequent observations demonstrated that they clearly do not represent that species. Although it is possible that the Bight 2008 and Bight 2013 specimens of *Idarcturus* collected near the Channel Islands may turn out to be a more spinous form of *I.allelomorphus*, this appears unlikely based on preliminary results of ongoing investigations. Consequently, we have designated these specimens as *Idarcturus* sp. A in this review.
8. *Edotia* sp. B is currently being described by TD Stebbins and R Wetzer (unpublished results, manuscript in preparation). This paper also includes a redescription of *E.sublittoralis* with a global review of the distribution and ecology of the genus.
9. The three species of *Synidotea* characterized by an apically rounded or spatulate pleotelson (*S.calcarea*, *S.magnifica*, and *S.media*) that occur in SCB waters are difficult to distinguish using the existing literature (e.g., Menzies and Barnard 1959; Schultz 1966; Iverson 1972; Menzies and Miller 1972; Wetzer and Brusca 1997). The problem is primarily due to an incomplete original description of *S.magnifica*. For a more detailed discussion of the issue and its resolution, see Stebbins (2012a).
10. The idoteid isopods *Idoteafewkesi*, *I.rufescens*, and *I.urotoma* included in the SCB fauna were listed in the genus *Pentidotea* in SCAMIT (2021). Although Poore and Lew Ton (1993) provided a more restrictive diagnosis of the genus *Idotea* while also raising *Pentidotea* to full generic status, they did not recommend that species previously assigned to *Idotea* be transferred to *Pentidotea* or vice versa. Consequently, species formerly assigned to the subgenera *Idotea* and *Pentidotea* were placed in the genera *Idotea* and *Pentidotea*, respectively, as is currently accepted in WoRMS (see Boyko et al. 2008 onwards). Although the proper affinities of some species of *Idotea* such as *I.fewkesi* and *I.urotoma* are uncertain at this time due to the more restrictive generic diagnosis as pointed out by Poore and Lew Ton (1993), reassignment of any of these species to *Pentidotea* or another existing or new genus awaits further investigation.
11. *Janiralatarajata* and *Janiralata* sp. D may be difficult to distinguish unless comparing actual specimens of both species. For example, Wilson (1997) stated that *Janiralata* sp. D differs from *J.rajata* as follows: “the eyes are smaller, the body has numerous dense chromatophores, the antennula is longer, and the propodus of pereopod I has more proximoventral denticles (18 instead of 12 or 13).” Another possible difference is that *Janiralata* sp. D has only been reported from continental shelf waters deeper than 90 m in the Santa Maria Basin, while *J.rajata* is reported from shallower waters < 40 m deep from central to southern California.
12. Although no illustrations exist of the provisional asellote species *Pleurocope* sp. A, the SCAMIT voucher sheet for the species compares it to another provisional species of the same name in manuscript (i.e., *Pleurocope* sp. A Wilson MS), as well as to three of the other four recognized species of the genus (voucher sheet for *Pleurocope* sp. IS1 available from SCAMIT Toolbox at https://www.scamit.org; D. B. Cadien, 4 March 2012). These three species include *P.dasyura* from the Mediterranean, *P.floridensis* from the tropical western Atlantic, and *P.wilsoni* from Thailand and Aldabra. The fourth species, *P.iriomotensis*, is from Japan. An image of *P.floridensis* modified after Kensley and Schotte (1989: fig. 43B) is provided in Fig. 35E herein as an example of the genus.
13. Just and Wilson (2021) recently redescribed the SCB asellote *Munnogoniumtillerae* (Menzies & Barnard, 1959) in their review of several species in the ‘ *Austrosignum-*’ complex sensu Just and Wilson (2007). In doing so, these authors compared *M.tillerae* collected off San Diego to *Munnogoniumerratum* (Schultz, 1964) known only from the type locality off Santa Barbara County, as well as to specimens identified as *Munnogonium* cf. *tillerae* in Wilson (1997) that were also collected off Santa Barbara in the Santa Maria Basin. As a result of their comparisons, Just and Wilson (2021) concluded that the San Diego and Santa Barbara specimens represent distinct species, and they therefore restricted *M.tillerae* to occurring just off San Diego. Although these authors reported that “the type of *M.erratum* has been destroyed,” they also stated that the specimens of *Munnogonium* cf. *tillerae* studied by Wilson (1997) were collected near that type locality and at around the same depth. Thus, Just and Wilson (2021) used the features of *M.* cf. *tillerae* described in Wilson (1997) as *M.erratum* in their accompanying key to species of *Munnogonium* (G Wilson, pers. comm.). This treatment is followed in this paper, with *M.tillerae* restricted to the San Diego region and *M.erratum* considered synonymous with *M.* cf. *tillerae* from the Santa Maria Basin (i.e., Fig. 35C herein was reproduced from Wilson 1997: fig. 1.28).
14. The provisional asellote species *Microcharon* sp. A was originally assigned to the family Microparasellidae as listed in SCAMIT (2021). However, this genus was transferred to the family Lepidocharontidae by Galassi et al. (2016). Although no complete illustrations exist for *Microcharon* sp. A, the species is similar in general appearance to the Caribbean species *Microcharonsabulum* Kensley, 1984. Thus, an image of *M.sabulum* is provided in Fig. 39C as an example of the genus.
15. No illustrations exist for the SCB asellotes *Caecianiropsis* sp. LA1 and *Caecianiropsis* sp. LA2. However, these two provisional species are similar in general morphology to the type species of the genus from northern California, *Caecianiropsispsammophila* Menzies & Petit, 1956. Thus, an image of *C.psammophila* is provided in Fig. 39B herein as an example of the genus.
16. No illustrations exist for the provisional species *Paramunna* sp. SD1 collected during the Bight 2003 regional monitoring project (see Ranasinghe et al. 2007). Thus, the species can presently be distinguished from the other SCB species of *Paramunna* only by the characters in Key I.
17. Hurtado et al. (2010) and Eberl et al. (2013) presented fine-scale phylogeographic studies of *Ligia* populations distributed from central California to central Mexico, including the Gulf of California, describing several highly divergent lineages. Their conclusions suggest the presence of multiple *Ligia* species spanning this region, including at least one unnamed species that ranges throughout the SCB from south of Point Conception to NW Baja California, including the Channel Islands, as well as perhaps further south along the Baja peninsula. This species corresponds to the “Baja Pacific-Southern California” clade of Hurtado et al. (2010) and is referred to in this guide as *Ligia* cf. *occidentalis*.

### ﻿Annotated list of species

**Suborder Cymothoida**

**Superfamily Anthuroidea**

#### ﻿Family Antheluridae

*Ananthuraluna* (Schultz, 1966) [Fig. 6E] Continental shelf, slope, and submarine canyon benthos (78–1298 m); Santa Monica Bay, Tanner Canyon, La Jolla, and Coronado canyons, southern California, USA. Type locality: USA, California, Tanner Canyon (R/V Velero IV Station No. 6832-60; 32°33'36"N, 118°55'40"W). Body length to ~ 21 mm. See Schultz (1966 original description as *Bathuraluna*; 1969, 1977), Kensley (1978), Poore and Lew Ton (1988d), Wetzer et al. (1991), Cadien and Brusca (1993), McLaughlin et al. (2005), Espinosa-Pérez and Hendrickx (2006), Ranasinghe et al. (2007), SCAMIT (2008–2021), Espinosa-Pérez et al. (2009), and Gillett et al. (2019).

#### ﻿Family Anthuridae

*Amakusanthuracaliforniensis* (Schultz, 1964) [Fig. 6A] Continental shelf (depth to at least 80 m); Santa Maria Basin/Western Santa Barbara Channel, southern California, USA to Guadalupe Island off western Baja California Norte, Mexico. Type locality: USA, California, Los Angeles County, 9.9 mi SSW of Santa Monica Pier (R/V Velero IV Station No. 2998-55; 33°53'22"N, 118°34'40"W). Body length to ~ 11 mm. See Schultz (1964 original description as *Apanthuracaliforniensis*; 1969), Lissner et al. (1986), Poore and Lew Ton (1988b), Wetzer et al. (1991), Cadien and Brusca (1993), SCAMIT (1994–2021), Wetzer and Brusca (1997), Ranasinghe et al. (2003, 2007, 2012), McLaughlin et al. (2005), and Espinosa-Pérez and Hendrickx (2006).

*Cyathuramunda* Menzies, 1951 [Fig. 6B] Low intertidal to continental shelf, on kelp holdfasts to deep-water rocks (0–132 m); Marin County, northern California, USA to NW Baja California, Mexico, and the Gulf of California. Type locality: USA, California, Marin County, Tomales Point, Tomales Bluff (reef). Body length to ~ 6 mm. See Menzies (1951b), Menzies and Barnard (1959), Schultz (1964, 1969), Miller (1975), Kussakin (1982), Lissner et al. (1986), Wetzer et al. (1991), Cadien and Brusca (1993), SCAMIT (1994–2021), Chess and Hobson (1997), Wetzer and Brusca (1997), Espinosa-Pérez and Hendrickx (2001a, 2006), McLaughlin et al. (2005), and Gillett et al. (2017).

*Haliophasmageminatum* Menzies & Barnard, 1959 [Fig. 6C] Continental shelf, slope, and submarine canyon benthos (9–512 m); Strait of Georgia, British Columbia, Canada to San Quintin Bay, Baja California Norte, Mexico. Type locality: USA, California, off San Mateo Point (R/V Velero IV Station No. 4771-56; 33°21'40"N, 117°35'50"W). Body length to ~ 8 mm. See Menzies and Barnard (1959 original description as *Haliophasmageminata*; gender of specific name corrected to *geminatum* in Boyko et al. 2008 onwards), Menzies (1962a), Schultz (1964, 1966, 1969), Lie (1968), Iverson (1974), Poore (1975), Gillard (1978), Lissner et al. (1986), Poore and Lew Ton (1988a), Wetzer et al. (1991), Cadien and Brusca (1993), Thompson et al. (1993), SCAMIT (1994–2021), Wetzer and Brusca (1997), Bergen et al. (1998, 2001), Smith et al. (2001, 2003), Ranasinghe et al. (2003, 2007, 2012), McLaughlin et al. (2005), Espinosa-Pérez and Hendrickx (2006), Campos and Villarreal (2008), Macdonald et al. (2010), Puget Sound Institute (2012–2023), Gillett et al. (2017, 2019, 2022), and Henkel et al. (2020).

*Mesanthuraoccidentalis* Menzies & Barnard, 1959 [Fig. 6D] Low intertidal to shallow water, on kelp and rocks (0–20 m); Point Conception, southern California, USA to Baja California and the Gulf of California, Mexico, and to the Gulf of Nicoya, Costa Rica. Type locality: California, 11 miles east of Point Conception (R/V Velero IV Station No. 4822-57; 34°27'15"N, 120°14'45"W). Body length to ~ 7 mm. See Menzies and Barnard (1959), Schultz (1964, 1969), Allen (1976), Wägele (1984b), Wetzer et al. (1991), Cadien and Brusca (1993), Chess and Hobson (1997), Espinosa-Pérez and Hendrickx (2001a), McLaughlin et al. (2005), Campos and Villarreal (2008), and SCAMIT (2012–2021).

#### ﻿Family Expanathuridae

*Eisothistos* sp. A [No figure] Inner to mid shelf offshore habitats collected off rocks and the coral *Coenocyathusbowersi* (5–77 m); southern California, USA from Tajiguas, Santa Barbara County and Catalina Island. Body length of juveniles to ~ 2.5 mm (i.e., adults not known). See Cadien and Brusca (1993) and SCAMIT (2013–2021). Note: See Endnote 2.

#### ﻿Family Hyssuridae

*Kupellonura* sp. A [Fig. 6F] Continental shelf, hard bottom benthos (105–117 m); Santa Maria Basin offshore of Purisima Point, Santa Barbara County, California, USA (~ 35 km north of SCB northern boundary). Body length = no information available. See Wetzer and Brusca (1997: 19–20, fig. 1.7, description and figure) and SCAMIT (2008–2021).

#### ﻿Family Paranthuridae

*Califanthurasquamosissima* (Menzies, 1951) [Fig. 5C] Intertidal to offshore continental shelf and bays, in kelp and eelgrass beds, and on muddy or sandy sediments (0–142 m); Dillon Beach, northern California, USA to Oaxaca, Mexico, and the Gulf of California. Type locality: USA, California, Marin County, Tomales Point, Tomales Bluff. Body length to ~ 5.6 mm. See Menzies (1951b original description as *Colanthurasquamosissima*), Menzies and Barnard (1959), Schultz (1969), Miller (1975), Brusca (1980), Kussakin (1982), Poore (1984b), Wetzer et al. (1991), Cadien and Brusca (1993), SCAMIT (1994–2021), Espinosa-Pérez and Hendrickx (2001a, 2006), McLaughlin et al. (2005), Brusca et al. (2007), Moore and Hovel (2010), Ranasinghe et al. (2012), Bastida-Zavala et al. (2013), Cruz-García et al. (2013), Gillett et al. (2017), and García-Madrigal et al. (2022).

*Colanthurabruscai* Poore, 1984 [Fig. 5D] Intertidal to shallow subtidal (0–27 m); San Clemente, southern California, USA to Salinas Bay, Costa Rica. Type locality: Mexico, Sonora, Puerto Peñasco, Station Beach Reef (on *Sargassum*; 31°20'N, 113°35'W). Body length to ~ 5.4 mm. See Poore (1984b), Wetzer et al. (1991), Cadien and Brusca (1993), Espinosa-Pérez and Hendrickx (2001a, 2006), McLaughlin et al. (2005), and Brusca et al. (2007).

*Paranthuraelegans* Menzies, 1951 [Fig. 5A] Intertidal to subtidal, in low rocky intertidal, eelgrass beds, algal mats, muddy sediments, and pier pilings (0–55 m); Marin County, northern California, USA to San Quintin Bay, Baja California Norte, Mexico, and the Gulf of California. Type locality: USA, California, Marin County, Tomales Point, Tomales Bluff (reef). Body length to ~ 9.1 mm. See Menzies (1951b, 1962a), Menzies and Barnard (1959), Miller (1975), Brusca (1980), Kussakin (1982), Lissner et al. (1986), Wetzer et al. (1991), Cadien and Brusca (1993), SCAMIT (1994–2021), Bergen et al. (1998), Espinosa-Pérez and Hendrickx (2001a, 2006), Ranasinghe et al. (2003, 2007, 2012), Smith et al. (2003), Healey and Hovel (2004), McLaughlin et al. (2005), Reed and Hovel (2006), Sirota and Hovel (2006), Brusca et al. (2007), and Melwani and Kim (2008).

*Paranthurajaponica* Richardson, 1909 [Fig. 5B] Introduced Asian species that occurs in fouling communities and shallow-water marinas; worldwide distribution, including northern and southern Japan, eastern Russia, eastern China, southwestern France, and from northern to southern California, USA in North America. Type locality: Japan, Mororan. Body length to ~ 8.5 mm. See Richardson (1909), Kussakin (1982), Cohen and Carlton (1995), Cohen et al. (2005), Brusca et al. (2007), Ruiz et al. (2011), Lavesque et al. (2013), SCAMIT (2013–2021), and Gillett et al. (2017, 2022).

**Superfamily Cymothooidea**

#### ﻿Family Aegidae

*Aegalecontii* (Dana, 1854) [Fig. 8E] Continental shelf and submarine canyons (depth to at least 218 m), collected from fishes or soft-bottom habitats; central to southern California, USA. Type locality: USA, California. Body length to ~ 20 mm. See Dana (1854 original description as *Aegacyllalecontii*), Stimpson (1857), Richardson (1899, 1900b, 1905a), Hewatt (1946), Schultz (1966, 1969), Miller (1975), Kussakin (1979), Brusca (1983a), SCAMIT (2001–2021), McLaughlin et al. (2005), Espinosa-Pérez and Hendrickx (2006), Brusca et al. (2007), and Bruce (2009a). Note: Although Dana (1854) did not designate a specific type specimen or locality for *A.lecontii*, the specimen on which his original description was based is believed to be in the invertebrate zoology collections of the Yale Peabody Museum of Natural History (Catalog No. YPM IZ 000303.CR).

*Aegiochusplebeia* (Hansen, 1897) [Fig. 8F] Deep water off oceanic islands (688–2534 m); eastern Pacific (Aleutian Islands to Peru), western Pacific (Indonesia to Japan), and New Caledonia. Type locality (syntypes): Eastern Pacific off Cocos Island and Panama (Albatross Station No. 3363). Body length to ~ 37 mm. See Hansen (1897 original description as *Aegaplebeia*), Richardson (1909 as *Aegamagnoculis*), Birstein (1973), Brusca (1983a), Brusca and Iverson (1985), Bruce (2009a), Azofeifa-Solano and Cortés (2020), and Zúñiga Delgado et al. (2021). Note: See Endnote 3.

*Rocinelaangustata* Richardson, 1904 [Fig. 9A] Continental shelf, slope, and basins (30–2534 m), collected from fishes or soft-bottom habitats; Bering Sea, Alaska, USA to Baja California, Mexico, and the Gulf of California. Type locality (lectotype): Bering Sea, USA, Alaska, NW of Unimak Island (54°48'50"N, 165°42'W). Body length to ~ 37.4 mm. See Richardson (1904a, 1905a), Hatch (1947), George and Strömberg (1968), Schultz (1969), Birstein (1973), Miller (1975), Lissner et al. (1986), Brusca and France (1992), SCAMIT (1994–2021), Wetzer and Brusca (1997), Smith et al. (2001), Espinosa-Pérez and Hendrickx (2002, 2006), McLaughlin et al. (2005), Moles (2007), Ranasinghe et al. (2007), Bruce (2009a), Macdonald et al. (2010), Gillett et al. (2017, 2019), and Henkel et al. (2020).

*Rocinelabelliceps* (Stimpson, 1864) [Fig. 9B] Continental shelf and slope (shallow subtidal to 284 m), collected from fishes or soft-bottom habitats; Alaska, USA to western Baja California, Mexico, and the Gulf of California. Type locality (neotype): USA, Washington, San Juan Island. Body length to ~ 29.1 mm. See Stimpson (1864 original description as *Aegabelliceps*), Richardson (1898, 1899, 1905a), Fee (1927), Hatch (1947), Schultz (1966, 1969), George and Strömberg (1968), Miller (1975), Allen (1976), Kussakin (1979), Brusca and France (1992), SCAMIT (1994–2021), Chess and Hobson (1997), Espinosa-Pérez and Hendrickx (2001a, 2002, 2006), McLaughlin et al. (2005), Moles (2007), Macdonald et al. (2010), Puget Sound Institute (2012–2023), and Ranasinghe et al. (2012).

*Rocinelalaticauda* Hansen, 1897 [Fig. 9C] Continental shelf and slope (120–906 m), collected from fishes or soft bottom habitats; San Simeon, California, USA and Acapulco, Mexico. Type locality (lectotype): Mexico, off Acapulco. Body length to ~ 41.4 mm. See Hansen (1897), Richardson (1898, 1899, 1900b, 1905a), Schultz (1969), Brusca and France (1992), Espinosa-Pérez and Hendrickx (2001a, 2006), Bruce (2009a), and García-Madrigal et al. (2022).

*Rocinelamurilloi* Brusca & Iverson, 1985 [Fig. 9D] Continental slopes and basins (700–1866 m), collected from fishes or soft-bottom habitats; Point Sur, California, USA to Arica, Chile. Type locality: Costa Rica, 14 miles 233°T from Point Guiones at the mouth of Golfo de Nicoya (9°45'N, 85°55'W). Body length to ~ 35 mm. See Brusca and Iverson (1985), Wetzer et al. (1991), Brusca and France (1992), Espinosa-Pérez and Hendrickx (2001a), Hendrickx (2008, 2018a), Bruce (2009a), and Azofeifa-Solano and Cortés (2020). Note: Although *R.murilloi* has also been listed as occurring in mangrove lagoons or estuaries in Baja California Sur (table 15.1 in Whitmore et al. 2005), we suspect that record may be erroneous as the species is otherwise known only from offshore waters of 700 m or deeper.

*Rocinelasignata* Schioedte & Meinert, 1879 [Fig. 9E] Mostly intertidal to shallow subtidal in embayments and on the offshore continental shelf (0–68 m), collected from fishes or soft-bottom habitats; Humboldt Bay, northern California, USA to Baja California and the Gulf of California, Mexico, and south to Costa Rica, Panama, Colombia and Ecuador, including the Galapagos Islands in the eastern Pacific, and in the western Atlantic, Caribbean, and Gulf of Mexico from Florida, USA to northeastern Brazil. Type locality (lectotype): West Indies, St. Croix Island. Body length to ~ 20.6 mm. See Schioedte and Meinert (1879), Richardson (1898, 1905a), Menzies and Glynn (1968), Bowman (1977), Brusca and Iverson (1985), Garzón-Ferreira (1990), Brusca and France (1992), Espinosa-Pérez and Hendrickx (2001a, 2002, 2006), Brusca et al. (2004), McLaughlin et al. (2005), Bunkley-Williams et al. (2006), Lazarus-Agudelo and Cantera-Kintz (2007), Bruce (2009a), Cavalcanti et al. (2012), Bastida-Zavala et al. (2013), Pires-Vanin et al. (2014), Fogg et al. (2016), Cardoso et al. (2017), Shaughnessy et al. (2017), Aguilar-Perera et al. (2018), Ortiz and Lalana (2018), Alves et al. (2019), Silva et al. (2019), Aguilar-Perera (2022), Aguilar-Perera and Nóh-Quiñones (2022), and García-Madrigal et al. (2022).

#### ﻿Family Cirolanidae

*Cirolanadiminuta* Menzies, 1962 [Fig. 7A] Intertidal to subtidal, on rocks, sandy silt sediments, algae, and member of the demersal zooplankton community (0–50 m); Point Conception, southern California, USA to western Baja California and Gulf of California, Mexico, and the Galapagos Islands. Type locality: Mexico, Baja California, Bahia de San Quintin. Body length to ~ 10.5 mm. See Menzies (1962a), Schultz (1969), Bruce and Bowman (1982), Stepien and Brusca (1985), Bruce (1986a), SCAMIT (1994–2021), Brusca et al. (1995, 2007), Chess and Hobson (1997), Espinosa-Pérez and Hendrickx (2001a), and McLaughlin et al. (2005).

*Cirolanaharfordi* (Lockington, 1877) [Fig. 7B] Intertidal to shallow subtidal, abundant in mussel beds on rocky shores, also occurs in mangrove lagoons or estuaries at southern end of the range; Vancouver Island, Canada to Magdalena Bay, Baja California Sur, Mexico. Type locality (probable syntypes; see Brusca et al. 1995): USA, California, Santa Rosa Island. Body length to ~ 20 mm. See Lockington (1877b original description as *Aegaharfordii*), Richardson (1899, 1900b, 1905a), Stafford (1913a), Shearer (1942), Hewatt (1946), Hatch (1947), George and Strömberg (1968), Schultz (1969), Miller (1975), Allen (1976), Johnson (1976a, 1976b), Gillard (1978), Kussakin (1979), Lee and Miller (1980), Bruce (1981), Ricketts et al. (1985), Brusca et al. (1995, 2007), Espinosa-Pérez and Hendrickx (2001a, 2002, 2006), McLaughlin et al. (2005), Whitmore et al. (2005), Campos and Villarreal (2008), SCAMIT (2011–2021), Roletto et al. (2014), Bugnot et al. (2015), Lafferty and Suchanek (2016), and Morales-Zarate et al. (2016).

*Eurydicecaudata* Richardson, 1899 [Fig. 7C] Intertidal to continental shelf (0–160 m), and occasionally on fishes (e.g., California scorpionfish and longfin sanddab); San Diego, southern California, USA to western Baja California Sur and the Gulf of California, Mexico, and south to Ecuador, including the Galapagos and other offshore islands of the tropical eastern Pacific. Type locality: USA, California, Catalina Island, Isthmus Cove. Body length to ~ 9 mm. See Richardson (1899, 1900b, 1905a), Menzies and Barnard (1959 as *E.branchuropus*), Schultz (1966 as *E.branchuropus*; 1969 as both *E.branchuropus* and *E.caudata*), Bowman (1977), Brusca and Iverson (1985), Bruce (1986a), Hobson and Chess (1986), SCAMIT (1994–2021), Brusca et al. (1995, 2004, 2007), Espinosa-Pérez and Hendrickx (2001a, 2002, 2006), Smith et al. (2001, 2003), Ranasinghe et al. (2003, 2007, 2012), McLaughlin et al. (2005), Kalman (2006), Campos and Villarreal (2008), and Gillett et al. (2017, 2019, 2022). Note: If confirmed, the Gillett et al. (2019) record extends the maximum depth range of this species slightly from 160 m to the upper slope between 200–500 m.

*Excirolanachiltoni* (Richardson, 1905) [Fig. 7D] Intertidal on sandy beaches; British Columbia, Canada to southern California, USA, as well as Japan, Taiwan, and Hong Kong. Type locality: USA, California, San Francisco. Body length to ~ 13.2 mm. See Richardson (1905a original description as *Cirolanachiltoni*), Richardson (1912), Schultz (1969), Klapow (1972), Iverson (1974), Miller (1975), Kussakin (1979), Bruce and Jones (1981), Dexter (1983), Bruce (1986a), Brusca et al. (1995, 2007), Kaneko and Omori (2005), McLaughlin et al. (2005), Espinosa-Pérez and Hendrickx (2006), Ranasinghe et al. (2007, 2012), SCAMIT (2011–2021), Gillett et al. (2017), and Shaughnessy et al. (2017).

*Excirolanalinguifrons* (Richardson, 1899) [Fig. 7E] Intertidal on sandy beaches; Coos Bay, Oregon to southern California, USA. Type locality: USA, California, Monterey Bay. Body length to ~ 3.8 mm. See Richardson (1899 original description as *Cirolanalinguifrons*; 1900b, 1905a, 1912), Shearer (1942), Schultz (1969), Miller (1975), Kussakin (1979), Bruce (1986a), Brusca et al. (1995, 2007), SCAMIT (2001–2021), Ranasinghe et al. (2003), McLaughlin et al. (2005), Espinosa-Pérez and Hendrickx (2006), and Campos and Villarreal (2008).

*Metacirolanajoanneae* (Schultz, 1966) [Fig. 7F] Offshore submarine canyons and basins (218–500 m); Strait of Georgia, British Columbia, Canada to southern California, USA, including Santa Cruz Canyon. Type locality: USA, California, Santa Cruz Canyon (R/V Velero IV Station No. 6805-59; 33°35'59"N, 119°15'11"W). Body length to ~ 5 mm. See Schultz (1966 original description as *Cirolanajoanneae*, but with prior listing in Schultz 1964 designated a *nomen nudum*; 1969), Bruce (1981, 1986a), Lissner et al. (1986), Wetzer et al. (1991), Brusca et al. (1995), Wetzer and Brusca (1997), McLaughlin et al. (2005), Macdonald et al. (2010), and SCAMIT (2011–2021).

*Natatolanacaliforniensis* (Schultz, 1966) [Fig. 7G] Offshore submarine canyons and basins, on fine sand and muddy bottoms (792–1250 m); primarily southern California, USA (e.g., Coronado Canyon, Tanner Canyon), with a few records from the Gulf of California, Mexico and further south (Costa Rica and Peru-Chile Trench). Type locality: USA, California, Coronado Canyon offshore southern San Diego (R/V Velero IV Station No. 6851-60; 32°37'54"N, 118°55'40"W). Body length to ~ 13.4 mm. See Schultz (1966 original description as *Cirolanacaliforniensis*; 1969), Brusca and Ninos (1978), Bruce (1981, 1986a), Brusca and Iverson (1985), Wetzer et al. (1991), Brusca et al. (1995), Espinosa-Pérez and Hendrickx (2001a, 2006), McLaughlin et al. (2005), Keable (2006), Espinosa-Pérez et al. (2009), SCAMIT (2011–2021), Ranasinghe et al. (2012), Gillett et al. (2019), and Zúñiga Delgado et al. (2021).

#### ﻿Family Corallanidae

*Excorallanatricornisoccidentalis* Richardson, 1905 [Fig. 8A] Intertidal to continental shelf, on rocks, sandy beaches, in mangrove habitats, and as a commensal in sponges or temporary fish parasite (0–138 m); Catalina Island, southern California, USA to western Baja California Sur and the Gulf of California, Mexico, and south to Costa Rica and Panama. Type locality: Mexico, Gulf of California. Body length to ~ 11 mm. See Richardson (1905a), Bowman (1977), Delaney (1984, 1989, 1993), Brusca and Iverson (1985), Guzman et al. (1988), Espinosa-Pérez and Hendrickx (2001a, 2006), and Brusca et al. (2007).

*Excorallanatruncata* (Richardson, 1899) [Fig. 8B] Intertidal to continental shelf (0–183 m); Point Conception, southern California, USA to Galapagos Islands, including Gulf of California (Bahia Concepcion). Type locality: USA, California, Catalina Island. Body length to ~ 21.5 mm. See Richardson (1899 original description as *Corallanatruncata*; 1900b, 1905a), Delaney (1982, 1984, 1989), SCAMIT (1994–2021), Espinosa-Pérez and Hendrickx (2001a, 2002), McLaughlin et al. (2005), Brusca et al. (2007), Melwani and Kim (2008), and Gillett et al. (2019). Note: If confirmed, the Gillett et al. (2019) record extends the maximum depth range of this species slightly from 183 m to the upper slope between 200 and 500 m.

#### ﻿Family Cymothoidae

*Ceratothoagaudichaudii* (H. Milne Edwards, 1840) [Fig. 11A] Parasite of many species of pelagic fishes, including striped mullet off California, and pompano and herring from Baja California; southern California, USA to western Baja California, Mexico, and southward to Cape Horn and around to southern Patagonia, South America. Type locality: Chile, Coquimbo. Body length to ~ 55 mm. See H. Milne Edwards (1840 original description as *Cymothoagaudichaudii*), Richardson (1899, 1905a as *Meinertiagaudichaudii*), Menzies (1962b as *Meinertiagaudichaudi*), Schultz (1969), Brusca (1977, 1981), Brusca and Iverson (1985), Espinosa-Pérez and Hendrickx (2001a, 2002, 2006), McLaughlin et al. (2005), Whitmore et al. (2005), Brusca et al. (2007), and Martin et al. (2015). Note: Species listed as *species inquirenda* by Martin et al. (2015), Hadfield and Smit (2020), and Zúñiga Delgado et al. (2021). Note: See Endnote 6.

*Ceratothoagilberti* (Richardson, 1904) [Fig. 11B] Parasite in mouths of mullets and an unidentified flatfish; Newport Bay, Orange County, southern California, USA to western Baja California, Mexico, and the Gulf of California to Mazatlán. Type locality: Mexico, Mazatlán. Body length to ~ 29 mm. See Richardson (1904a original description as *Meinertiagilberti*; 1905a), Schultz (1969), Brusca (1977, 1981), Espinosa-Pérez and Hendrickx (2001a, 2006), Brusca et al. (2004), McLaughlin et al. (2005), Whitmore et al. (2005), Martin et al. (2015), Hadfield et al. (2016), and Hadfield and Smit (2020).

*Elthusacalifornica* (Schioedte & Meinert, 1884) [Fig. 11C] Parasite of dwarf surfperch, shiner surfperch, surf smelt, topsmelt, arrow goby, California killifish, and California grunion (nearshore to 90 m), common in bays and lagoons; Alaska, USA to Punta Eugenio, Baja California Sur, Mexico, and off Peru (but uncommon south of San Diego, California and north of Washington State). Type locality: USA, California, near San Francisco. Body length to ~ 15 mm. See Schioedte and Meinert (1884 original description as *Livonecacalifornica*, but genus misspelled as *Lironeca*), (Richardson 1899, 1900b, 1905a), Fee (1927), Hatch (1947), Schultz (1969), Iverson (1974), Miller (1975), Brusca (1977, 1981), Kussakin (1979), Bruce (1990), SCAMIT (1998–2021), McLaughlin et al. (2005), Espinosa-Pérez and Hendrickx (2006), Brusca et al. (2007), Espinosa-Pérez et al. (2009), Shaughnessy et al. (2017), van der Wal et al. (2019), and Zúñiga Delgado et al. (2021).

*Elthusamenziesi* (Brusca, 1981) [Fig. 11D] Parasite in gill chambers of the wooly sculpin, northern clingfish and reef finspot (tidepools to 30 m); Baja California Norte (e.g., Coronado Islands, Todos Santos Bay, San Quintin Bay, and Guadalupe Island) to Baja California Sur (e.g., Alijos Rocks) and the Gulf of California, Mexico. Type locality: Mexico, Baja California Norte, Guadalupe Island, Melpomene Cove. Body length to 21 mm. See Menzies (1962a as *Lironeca* sp. nov., but undescribed), Brusca (1981 original description as *Lironecamenziesi*), Campos-González et al. (1986), Bruce (1990), Espinosa-Pérez and Hendrickx (2001a, 2002, 2006), McLaughlin et al. (2005), Whitmore et al. (2005), and van der Wal et al. (2019).

*Elthusavulgaris* (Stimpson, 1857) [Fig. 11E] Parasite in gill chambers of at least 30 species of fishes (1–311 m); Coos Bay, Oregon, USA to Colombia, South America, and common from southern California, USA and the Gulf of California, Mexico to Costa Rica. Type locality: USA, California, San Francisco Bay. Body length to ~ 43 mm. See Stimpson (1857 original description as *Livonecavulgaris*), Richardson (1899, 1900b, 1905a), Shearer (1942), Hatch (1947), Schultz (1969), Miller (1975), Brusca (1978b, 1981), Kussakin (1979), Lee and Miller (1980), Brusca and Iverson (1985), Bruce (1990), Thompson et al. (1993), SCAMIT (1994–2021), Espinosa-Pérez and Hendrickx (2001a, 2006), Brusca et al. (2004, 2007), McLaughlin et al. (2005), Whitmore et al. (2005), Kalman (2006), Ranasinghe et al. (2007), Shaughnessy et al. (2017), van der Wal et al. (2019), and García-Madrigal et al. (2022).

*Mothocyarosea* Bruce, 1986 [Fig. 10D] Parasite of California halfbeak and skipper halfbeak; San Diego, southern California, USA to Magdalena Bay, Baja California Sur, Mexico, and Nicaragua. Type locality: Mexico, Baja California Sur, San Ignacio Lagoon (~ 26°25'N, 113°13'W). Body length to ~ 8.4 mm. See Bruce (1986b), Wetzer et al. (1991), McLaughlin et al. (2005), Espinosa-Pérez and Hendrickx (2006), Brusca et al. (2007), and Espinosa-Pérez et al. (2009).

*Nerocilaacuminata* Schioedte & Meinert, 1881 [Fig. 10B] Parasite of ~ 40 species of fishes, especially common in shallow coastal lagoons and bays; southern California, USA to Peru, including the west coast of Baja California, Mexico, the Gulf of California, Costa Rica, Peru, the offshore islands of Las Tres Marias and the Galapagos, Hawaii, and in the southern Atlantic Ocean. Type locality: not specified, but the types for *N.acuminata* and its junior synonym *N.californica* as designated by Brusca (1981) are deposited at Muséum National d’Histoire Naturelle, Paris. Body length to ~ 25 mm. See Schioedte and Meinert (1881), Richardson (1900b, 1905a), Schultz (1969), Miller (1975), Brusca (1977, 1978a, 1981), Kussakin (1979), Brusca and Iverson (1985), SCAMIT (1994–2021), Espinosa-Pérez and Hendrickx (2001a, 2002, 2006), Brusca et al. (2004, 2007), McLaughlin et al. (2005), Whitmore et al. (2005), Fogg et al. (2016), Esteves-Silva et al. (2020), Zúñiga Delgado et al. (2021), Aguilar-Perera (2022), Bueno et al. (2022), and García-Madrigal et al. (2022). Note: See Brusca (1981: 157–158) for descriptions and discussion of the *acuminata* and *aster* forms of *N.acuminata*. Additionally, although Brusca (1981) synonymized *N.acuminata* with *N.californica* Schioedte & Meinert, 1881, both are currently accepted as separate species in WoRMS (Boyko et al. 2008 onwards). Also see Williams and Bunkley-Williams (2003) for additional information. However, until a rigorous morphological and molecular comparison can be made, we are continuing to treat these as the single synonymized species *N.acuminata* in agreement with Brusca (1981).

*Renocilathresherorum* Williams & Bunkley-Williams, 1980 [Fig. 10A] Parasite of barspot cardinalfish and Panamic fanged blenny in the Eastern Pacific (i.e., Corona del Mar, southern California, USA; Magdalena Bay, Baja California Sur and the central to southern Gulf of California, Mexico), and of Egyptian sole, European bass, redbelly tilapia, and thinlip grey mullet in the Suez Canal region of Egypt (e.g., Lake Qaroun). Type locality: Mexico, Baja California Sur, Loreto. Body length to ~ 30.5 mm. See Williams and Williams (1980), Brusca (1981), Williams and Williams (1987), Espinosa-Pérez and Hendrickx (2006), Kerstitch and Bertsch (2007), Eman et al. (2014), Mahmoud et al. (2016), Ali and Aboyadak (2018), and Aneesh et al. (2020, 2021). Note: See Endnote 5.

*Smenispaconvexa* (Richardson, 1905) [Fig. 10C] Parasite in mouths or gill chambers of Pacific bumper, pompanos, serranos, and unidentified carangids; southern California, USA to the Gulf of Guayaquil, Ecuador, and Peru. Type locality (neotypes; see Brusca 1981): Mexico, Nayarit, Playa Noviella, west of Tecuelao. Body length to ~ 15 mm. See Richardson (1905c original description as *Livonecaconvexa*), Menzies et al. (1955), Schultz (1969), Brusca (1977, 1981), Brusca and Iverson (1985), Wetzer et al. (1991), SCAMIT (1998–2021), Espinosa-Pérez and Hendrickx (2001a, 2006), McLaughlin et al. (2005), Whitmore et al. (2005), Kalman (2006), Brusca et al. (2007), Özdikem (2009), and Zúñiga Delgado et al. (2021).

#### ﻿Family Gnathiidae

*Caecognathiacrenulatifrons* (Monod, 1926) [Fig. 12A] Continental shelf, slope, and submarine canyons, benthic sediments (9–1376 m); Strait of Georgia, British Columbia, Canada to Todos Santos Bay, Baja California Norte, Mexico. Type locality: USA, California, La Jolla. Male body length to ~ 4.3 mm. See Monod (1926 original description as *Gnathiacrenulatifrons*), Menzies and Barnard (1959), Schultz (1964, 1966, 1969), Iverson (1974), Miller (1975), Lissner et al. (1986), Thompson et al. (1993), SCAMIT (1994–2021), Wetzer and Brusca (1997), Bergen et al. (1998), Smit and Davies (2004), McLaughlin et al. (2005), Espinosa-Pérez and Hendrickx (2006), Haney (2006), Ranasinghe et al. (2007, 2012), Campos and Villarreal (2008), Espinosa-Pérez et al. (2009), Macdonald et al. (2010), and Gillett et al. (2017, 2019, 2022).

*Caecognathiasanctaecrucis* (Schultz, 1972) [Fig. 12B] Continental shelf, slope, and submarine canyons, benthic sediments (76–218 m); Strait of Georgia, British Columbia, Canada to Todos Santos Bay, Baja California Norte, Mexico. Type locality: USA, California, Santa Cruz Canyon (R/V Velero IV Station No. 6805-59; 33°56'03"N, 119°52'03"W). Male body length ~ 2.6 mm. See Schultz (1966 original description as *Gnathiahirsuta*; 1972 for “substitution for preoccupied name”), Lissner et al. (1986), Wetzer et al. (1991), SCAMIT (1994–2021), Wetzer and Brusca (1997), Bergen et al. (1998), Smit and Davies (2004), McLaughlin et al. (2005), Espinosa-Pérez and Hendrickx (2006), Haney (2006), Campos and Villarreal (2008), Macdonald et al. (2010), and Gillett et al. (2017).

*Caecognathia* sp. A [No figure] Continental shelf (110–126 m); Western Santa Barbara Channel off Point Conception, southern California, USA. Male body length to ~ 4.3 mm. See Haney (2006) and SCAMIT (2008–2021). Note: See Endnote 8.

*Caecognathia* sp. SD1 [No figure] Continental shelf, soft-bottom benthos (116–153 m); San Diego, southern California, USA. Male body length ~ 3.4 mm. See Haney (2006), SCAMIT (2008–2021), Ranasinghe et al. (2012), and Gillett et al. (2022). Note: See Endnote 8.

*Gnathiaclementensis* Schultz, 1966 [Fig. 12C] Continental shelf, benthic sediments with manganese nodules (162 m); San Clemente Canyon, southern California, USA (known only from type locality). Type locality: USA, California, San Clemente Canyon (R/V Velero IV Station No. 6840-60; 32°44'35"N, 118°12'45"W). Male body length to ~ 8.5 mm. See Schultz (1966, 1969), Wetzer et al. (1991), Smit and Davies (2004), McLaughlin et al. (2005), Espinosa-Pérez and Hendrickx (2006), and Haney (2006).

*Gnathiacoronadoensis* Schultz, 1966 [Fig. 12D] Offshore submarine canyons, benthic sediments (344–812 m); Coronado Canyon, southern California, USA. Type locality: USA, California, Coronado Canyon (R/V Velero IV Station No. 6851-60; 32°30'42"N, 117°21'37"W). Male body length to ~ 3.5 mm. See Schultz (1966, 1969), Wetzer et al. (1991), Smit and Davies (2004), McLaughlin et al. (2005), and Haney (2006).

*Gnathiaproductatridens* Menzies & Barnard, 1959 [Fig. 12E] Continental shelf, benthic sediments (20–164 m); Point Conception, Santa Barbara County, southern California, USA. Type locality: USA, California, off Summerland (R/V Velero IV Station No. 5173-57; 34°14'50"N, 119°32'25"W). Male body length to ~ 3.2 mm. See Menzies and Barnard (1959), Schultz (1969), Lissner et al. (1986), Wetzer et al. (1991), SCAMIT (1996–2021), Wetzer and Brusca (1997), Smit and Davies (2004), McLaughlin et al. (2005), Espinosa-Pérez and Hendrickx (2006), Haney (2006), Ranasinghe et al. (2012), and Gillett et al. (2019). Note: If confirmed, the Gillett et al. (2019) record extends the maximum depth range of this species slightly from 164 m to the upper slope between 200–500 m.

*Gnathiasteveni* Menzies, 1962 [Fig. 12F] Intertidal; Strait of Georgia, British Columbia, Canada to San Quintin Bay, Baja California Norte, Mexico. Type locality: Mexico, Baja California, Bahia de San Quintin. Male body length to ~ 2.3 mm. See Menzies (1962a), Schultz (1969), Wetzer et al. (1991), Fairey et al. (2002), Smit and Davies (2004), McLaughlin et al. (2005), Espinosa-Pérez and Hendrickx (2006), Haney (2006), Brusca et al. (2007), SCAMIT (2008–2021), and Macdonald et al. (2010).

*Gnathiatridens* Menzies & Barnard, 1959 [Fig. 12G] Shallow subtidal (11–27 m); Strait of Georgia, British Columbia, Canada to San Clemente Island, southern California, USA. Type locality: USA, California, 11 miles east of Point Conception (R/V Velero IV Station No. 4822-57; 34°27'15"N, 120°14'45"W). Male body length to ~ 3 mm. See Menzies and Barnard (1959), Schultz (1969), Lissner et al. (1986), Wetzer et al. (1991), SCAMIT (1994–2021), Wetzer and Brusca (1997), Smit and Davies (2004), McLaughlin et al. (2005), Espinosa-Pérez and Hendrickx (2006), Haney (2006), Espinosa-Pérez et al. (2009), Macdonald et al. (2010), and Henkel et al. (2020).

*Gnathiatrilobata* Schultz, 1966 [Fig. 12H] Continental shelf and submarine canyons, collected from polychaete tubes and benthic sediments (98–976 m); Strait of Georgia, British Columbia, Canada to Point Loma, San Diego, southern California, USA. Type locality: USA, California, Coronado Canyon (R/V Velero IV Station No. 6851-60; 32°30'42"N, 117°21'37"W). Male body length to ~ 5 mm. See Schultz (1966, 1969), Wetzer et al. (1991), SCAMIT (1994–2021), Bergen et al. (1998), Smit and Davies (2004), McLaughlin et al. (2005), Espinosa-Pérez and Hendrickx (2006), Haney (2006), Ranasinghe et al. (2007), and Macdonald et al. (2010).

*Gnathia* sp. MBC1 [No figure] Shallow rocky reef, collected from a white sponge (~ 11 m); Pequenot’s Reef, Corona del Mar, southern California, USA. Male body length ~ 2.2 mm. See Haney (2006) and SCAMIT (2008–2021). Note: See Endnote 8.

#### ﻿Family Tridentellidae

*Tridentellaglutacantha* Delaney & Brusca, 1985 [Fig. 8C] Continental shelf and upper slope, on green muds or rocky bottoms (128–360 m); Farallon Islands, central California to Catalina Island and Los Angeles, southern California, USA. Type locality: USA, California, west of North Farallon Islands (37°43.8'N; 123°11.3'W). Body length to ~ 18 mm. See Delaney and Brusca (1985), Wetzer et al. (1991), Kensley and Heard (1997), Wetzer and Brusca (1997), Brandt and Poore (2001), McLaughlin et al. (2005), Espinosa-Pérez and Hendrickx (2006), Espinosa-Pérez et al. (2009), and SCAMIT (2013–2021).

*Tridentellaquinicornis* Delaney & Brusca, 1985 [Fig. 8D] Continental shelf, on “rock” substrates (depth of at least 53 m); Santa Barbara Island to near Catalina Island, southern California, USA. Type locality: USA, California, Los Angeles County, Farnsworth Bank near Catalina Island (R/V Velero IV Station No. 1903-49). Body length to ~ 11 mm. See Delaney and Brusca (1985), Wetzer et al. (1991), SCAMIT (1996–2021), Kensley and Heard (1997), Brandt and Poore (2001), McLaughlin et al. (2005), and Espinosa-Pérez and Hendrickx (2006).

**Infraorder Epicaridea**

**Superfamily Bopyroidea**

#### ﻿Family Bopyridae

*Anathelgeshyphalus* (Markham, 1974) [Fig. 13C] Branchial parasite of hermit crabs *Parapaguroideslaurentae* and *P.makarovi*, deep water (69–319 m); Carmel, California, USA to western Baja California Norte, Mexico. Type locality: USA, California, Santa Barbara, Channel Islands (R/V Velero III Station No. 1026-39; 33°00'N; 118°38'W) infesting *P.makarovi*. Female body length to ~ 6.1 mm (male ~ 2.6 mm). See Markham (1974a original description as *Stegophryxushyphalus*; 1992, 2003, 2020), Wetzer et al. (1991), SCAMIT (2001–2021),Boyko and Williams (2003), McLaughlin et al. (2005), Espinosa-Pérez and Hendrickx (2006), and Stebbins (2012b).

*Aporobopyrusmuguensis* Shiino, 1964 [Fig. 14B] Branchial parasite of the porcelain crabs *Pachychelesholosericus*, *P.pubescens*, and *P.rudis*, shallow water (10–12 m); Bodega Bay, northern California, USA to central western Baja California, Mexico. Type locality: USA, California, Point Mugu, off Mugu Pier infesting *P.rudis*. Female body length to ~ 4 mm (male ~ 2.7 mm). See Shiino (1964), Miller (1975), Lee and Miller (1980), Campos-González and Campoy-Favela (1987), Markham (1992, 2008), McLaughlin et al. (2005), Espinosa-Pérez and Hendrickx (2006), Brusca et al. (2007), Stebbins (2012b), and Aguilar-Perera (2022).

*Aporobopyrusoviformis* Shiino, 1934 [Fig. 14C] Branchial parasite of the porcelain crabs *Pachychelespubescens* and *Petrolisthespubescens*, shallow water (10–12 m); Point Mugu, California, USA, and Seto, Japan. Type locality: Japan, Seto, Yusaki infesting *Petrolisthespubescens*. Female body length to ~ 5.8 mm (male ~ 2.5 mm). See Shiino (1934), McLaughlin et al. (2005), Espinosa-Pérez and Hendrickx (2006), Brusca et al. (2007), and Stebbins (2012b).

*Argeiapugettensis* Dana, 1853 [Fig. 14A] Branchial parasite of multiple species of crangonid and hippolytid shrimp (intertidal to 188 m); Bering Sea to southern California, and the US-Mexico border, Japan, and Korea. Type locality: USA, Washington, Puget Sound infesting *Metacrangonmunitus*. Female body length to ~ 14 mm (male ~ 5.5 mm). See Dana (1853), Richardson (1899, 1900c, 1905a), Fee (1927), Shearer (1942), Hatch (1947), George and Strömberg (1968), Miller (1975), Markham (1977b, 1992), Higley and Holton (1978), Ricketts et al. (1985), Jay (1989), SCAMIT (2001–2021), McLaughlin et al. (2005), Espinosa-Pérez and Hendrickx (2006), Brusca et al. (2007), Espinosa-Pérez et al. (2009), Puget Sound Institute (2012–2023), and Stebbins (2012b).

*Asymmetrioneambodistorta* Markham, 1985 [Fig. 14D] Branchial parasite of the hermit crab *Isochelespilosus*, shallow water (3 m): southern California, USA. Type locality: USA, California, Newport Beach, Corona del Mar State Beach. Female body length to ~ 5.4 mm (male ~ 2.8 mm). See Markham (1985b, 2003), McLaughlin et al. (2005), Espinosa-Pérez and Hendrickx (2006), Brusca et al, (2007), and Stebbins (2012b).

*Bathygygegrandis* Hansen, 1897 [Fig. 13B] Branchial parasite of deep-water shrimp of the genus *Glyphocrangon* (1792–1896 m in SCB); worldwide distribution, including San Clemente Basin, southern California, USA. Type locality: Mexico, off Acapulco (21°15'N, 106°23'W) infesting *G. spinulosa*. Female body length to ~ 12.7 mm (male ~ 6.8 mm). See Hansen (1897), Richardson (1899, 1905a), Espinosa-Pérez and Hendrickx (2001a), Stebbins (2012b), Markham (2016, 2020), and García-Madrigal et al. (2022).

*Bopyrellacalmani* (Richardson, 1905) [Fig. 15A] Branchial parasite of alpheid snapping shrimps *Alpheopsisequidactylus* and *Synalpheuslockingtoni*, (intertidal to 9 m); Central to southern California, USA. Type locality: USA, California, between Santa Barbara and San Nicolas Island. Female body length to ~ 8 mm (male ~ 2.2 mm). See Richardson (1905a original description as *Bopyriscuscalmani*), Sassaman et al. (1984), McLaughlin et al. (2005), Espinosa-Pérez and Hendrickx (2006), and Stebbins (2012b).

*Capitetragonia* sp. A [Fig. 15B, image of representative species *C.alphei*] Branchial parasite of the undescribed alpheid shrimp *Automate* sp. A (30 m depth); collected offshore of Palos Verdes, Los Angeles County, southern California, USA. No body size information available, but total length for the type species of the genus, *Capitetragoniaalphei* (Richardson, 1900a), measured 5.1 mm (see Markham 1985a, p. 43). See SCAMIT (2012–2021) and Stebbins (2012b). Note: Provisional species originally described as *Probopyria* sp. A (see Endnote 9).

*Eremitionegiardi* (Calman, 1898) [Fig. 15C] Branchial parasite of hermit crabs of the genus *Pagurus*; Bering Sea to Palos Verdes, southern California, USA. Type locality: USA, Washington, Puget Sound infesting *P.armatus*. Female body length to ~ 12 mm (male ~ 3 mm). See Calman (1898 original description as *Pseudionegiardi*), Richardson (1899, 1900c, 1905a), Hatch (1947), George and Strömberg (1968), Markham (1974b), Boyko and Williams (2004), McLaughlin et al. (2005), Espinosa-Pérez and Hendrickx (2006), SCAMIT (2011–2021), Stebbins (2012b), and Williams et al. (2019). Note: Record of species in California waters questionable (see Endnote 10).

*Leidyainfelix* Markham, 2002 [Fig. 13D] Branchial parasite of the grapsid crab *Pachygrapsuscrassipes* (intertidal); southern California, USA to Baja California Norte, Mexico. Type locality: Mexico, Baja California Norte, San Quintín (30°28'N; 115°58'W). Female body length to ~ 7.9 mm (male ~ 1 mm). See Markham (2002), Boyko (2003), and Stebbins (2012b).

*Munidionpleuroncodis* Markham, 1975 [Fig. 13E] Branchial parasite of the pelagic galatheid crab *Pleuroncodesplanipes*; Central California, USA to central Mexico, typically occurring in southern California during warm water years (e.g., El Niños). Type locality: Mexico, Baja California (26°22'N, 115°05'W). Female body length to ~ 9.8 mm (male ~ 4.4 mm). See Markham (1975), Lissner et al. (1986), Wetzer and Brusca (1997), SCAMIT (2001–2021), McLaughlin et al. (2005), Espinosa-Pérez and Hendrickx (2006), Brusca et al. (2007), and Stebbins (2012b).

*Orthionegriffenis* Markham, 2004 [Fig. 14E] Branchial parasite of mud shrimp *Upogebiapugettensis* and *U.macginitieorum*; introduced species from Asia (China, Japan), occurring from British Columbia, Canada to southern California, USA along west coast of North America. Type locality: USA, Oregon, Yaquina Bay, Idaho Inlet mudflats (44°35.4'N, 124°01.5'W) infesting *U.pugettensis*. Female body length to ~ 24 mm (male to ~ 10.3 mm). See Markham (2004), Williams and An (2009), Dumbauld et al. (2011), Chapman et al. (2012), Puget Sound Institute (2012–2023), Stebbins (2012b), SCAMIT (2013–2021), Hong et al. (2015), and Whalen et al. (2020).

*Phyllodurusabdominalis* Stimpson, 1857 [Fig. 13F] Abdominal parasite of the mud shrimp *Upogebialepta*, *U.macginitieorum*, and *U.pugettensis* (intertidal): British Columbia, Canada to Baja California Norte, Mexico. Type locality (see Markham 1977a): USA, Washington, Puget Sound and California, Tomales Bay. Female body length to ~ 14 mm (male ~ 6 mm). See Stimpson (1857), Richardson (1899, 1900c, 1905a), Fee (1927), Hatch (1947), George and Strömberg (1968), Miller (1975), Markham (1977a, 1992), Lee and Miller (1980), Campos-González and Campoy-Favela (1987), SCAMIT (1994–2021), Espinosa-Pérez and Hendrickx (2006), Brusca et al. (2007), Stebbins (2012b), and Aguilar-Perera (2022).

*Progebiophilusbruscai* Salazar-Vallejo and Leija-Tristán, 1990 [Fig. 14F] Branchial parasite of the mud shrimp *Upogebiadawsoni*, *U.spinigera*, *U.macginitieorum*, and *Pomatogebiarugosa*; Todos Santos Bay, Ensenada, Baja California Norte, Mexico to Tortugas Bay, Baja California Sur, the Gulf of California, and Nicaragua. Type locality: Mexico, Baja California Sur, Bahía de la Paz, Laguna de La Paz, El Comitán infesting *U.dawsoni*. Female body length to ~ 12 mm (male ~ 3.9 mm). See Salazar-Vallejo and Leija-Tristán (1990), Campos and de Campos (1998), Espinosa-Pérez and Hendrickx (2001a, 2006), Markham (2005), Boyko et al. (2017), and Aguilar-Perera (2022).

*Pseudionegalacanthae* Hansen, 1897 [Fig. 15D] Branchial parasite of the squat lobsters *Galacanthadiomedeae* and *Munidaquadrispina*, deep water (> 50 m); British Columbia, Canada to Gulf of California, Mexico. Type locality: Mexico, central Gulf of California (Albatross Station No. 3435; 26°48'00"N, 110°45'20"W) infesting *Galacanthadiomedeae*. Female body length to ~ 11 mm (male ~ 4.8 mm). See Hansen (1897), Richardson (1899, 1905a), Fee (1927), Hatch (1947), George and Strömberg (1968), Markham (1974b), Espinosa-Pérez and Hendrickx (2001a, 2006), Boyko and Williams (2004), McLaughlin et al. (2005), and Stebbins (2012b). Note: Record of species in California waters questionable (J Markham, pers. comm.), nor is this species listed within SCB waters in Markham (2020).

*Schizobopyrinastriata* (Nierstrasz & Brender à Brandis, 1929) [Fig. 15E] Branchial parasite of the alpheoid shrimps *Hippolytecaliforniensis* in San Diego Bay and *Thoralgicola* in the Gulf of California, shallow water; California, USA to Baja California, Mexico, and the Gulf of California. Type locality: USA, California, San Diego, San Diego Bay. Female body length to ~ 4 mm. See Nierstrasz and Brender à Brandis (1929 original description as *Bopyrinastriata*), Ricketts et al. (1985), Campos and de Campos (1990), Markham (1992), Espinosa-Pérez and Hendrickx (2001a, 2006), McLaughlin et al. (2005), Brusca et al. (2007), and Stebbins (2012b).

#### ﻿Family Ionidae

*Ionecornuta* Spence Bate, 1863 [Fig. 13A] Branchial parasite of ghost shrimps of the genera *Callianassa* and *Neotrypaea* in the eastern Pacific, and *Nihonotrypaea* and *Upogebia* in Asia, intertidal to shallow water; British Columbia, Canada to California, USA, the Gulf of California, Mexico, Japan, Korea, and China. Type locality: Canada, British Columbia, Vancouver Island, infesting *Neotrypaeagigas* (originally as *Callianassalongimana*). Female body length to ~ 12 mm. See Bate (1863), Richardson (1899, 1900c, 1905a), Hatch (1947), Miller (1975), Markham (1992), Nobuhiro and Kyoko (2004), McLaughlin et al. (2005), Espinosa-Pérez and Hendrickx (2006), Brusca et al. (2007), An et al. (2009), Espinosa-Pérez et al. (2009), Stebbins (2012b), and Boyko et al. (2017).

**Superfamily Cryptoniscoidea**

#### ﻿Family Dajidae

*Holophryxusalaskensis* Richardson, 1905 [Fig. 16A] Ectoparasite on carapace of the Pacific glass shrimp *Pasiphaeapacifica*, mostly deep waters (44–1200 m); Prince William Sound, Alaska, USA to offshore of the US-Mexico border. Type locality: USA, Alaska, Behm Canal, vicinity of Yes Bay (~ 55°51'05"N, 131°49'43"W). Adult female body length to ~ 13.6 mm (male ~ 2.0 mm). See Richardson (1905a, 1905b, 1908), Butler (1964), Coyle and Mueller (1981), McLaughlin et al. (2005), Espinosa-Pérez and Hendrickx (2006), SCAMIT (2012–2021), Stebbins (2012b), Markham (2020), and Boyko and Williams (2021b). Note: See Endnote 11.

*Zonophryxus* sp. [Fig. 16B, image of representative species *Z.probisowa*] Ectoparasite on carapace of the caridean shrimps *Pantomusaffinis* and *Plesionikatrispinus*, offshore (98–305 m); southern California, USA between Point Dume and Imperial Beach. Body length = no information available. See Montagne and Cadien (2001), SCAMIT (2012–2021), Markham (2020), and Boyko and Williams (2021a). Note: See Endnote 12.

#### ﻿Family Hemioniscidae

*Hemioniscusbalani* Buchholz, 1866 [Fig. 16C] Parasite on barnacles of genera *Balanus* and *Chthamalus*, intertidal; worldwide distribution, including from at least British Columbia, Canada to Baja California, Mexico along the west coast of North America. Type locality: Norway, Kristiansand. See Buchholz (1866), Nierstrasz and Brender à Brandis (1926), Crisp (1968), Campos-González and Campoy-Favela (1987), McLaughlin et al. (2005), Espinosa-Pérez and Hendrickx (2006), SCAMIT (2012–2021), and Stebbins (2012b).

**Suborder Limnoriidea**

**Superfamily Limnorioidea**

#### ﻿Family Limnoriidae

*Limnoriaalgarum* Menzies, 1957 [Fig. 17A] Burrowing into algal holdfasts, intertidal to subtidal (0–15 m); Strait of Georgia, British Columbia, Canada to San Diego, southern California, USA. Type locality: USA, California, Marin County, Dillon Beach. Body length to ~ 5.5 mm. See Menzies (1957), Menzies and Barnard (1959), Schultz (1969), Miller (1975), Kussakin (1979), Lee and Miller (1980), Ricketts et al. (1985), Cookson (1991), SCAMIT (1996–2021), McLaughlin et al. (2005), Espinosa-Pérez and Hendrickx (2006), Brusca et al. (2007), Espinosa-Pérez et al. (2009), Macdonald et al. (2010), and Roletto et al. (2014).

*Limnoriaquadripunctata* Holthuis, 1949 [Fig. 17B] Burrowing into wood, intertidal to subtidal (0–30 m); worldwide cool temperate distribution, including Humboldt Bay, northern California to San Diego, southern California, USA. Type locality: North Sea coast of Dutch province of Zuid-Holland between villages of Katwijk and Noordwijk, not far from Leiden. Body length to ~ 3.4 mm. See Holthuis (1949), Menzies and Mohr (1952), Menzies (1957), Schultz (1969), Miller (1975), Kussakin (1979), Lee and Miller (1980), Ricketts et al. (1985), Cookson (1991, 1997), McLaughlin et al. (2005), Espinosa-Pérez and Hendrickx (2006), Brusca et al. (2007), Espinosa-Pérez et al. (2009), SCAMIT (2012–2021), Borges and Costa (2014), and Borges et al. (2014).

*Limnoriatripunctata* Menzies, 1951 [Fig. 17C] Burrowing into wood, intertidal to subtidal (0–7 m); temperate and tropical locations worldwide, including Puget Sound, Washington, USA to Mazatlán, Mexico, and the Gulf of California in the eastern Pacific. Type locality: USA, California, San Diego County, Mission Bay. Body length to ~ 4 mm. See Menzies (1951a, 1957), Menzies and Glynn (1968), Schultz (1969), Bastida and Torti (1972), Miller (1975), Allen (1976), Lee and Miller (1980), Brusca and Iverson (1985), Ricketts et al. (1985), Cookson (1991), Wetzer et al. (1991), Espinosa-Pérez and Hendrickx (2001a, 2006), Cohen et al. (2005), McLaughlin et al. (2005), Brusca et al. (2007), SCAMIT (2008–2021), Puget Sound Institute (2012–2023), Borges and Costa (2014), Borges et al. (2014), Shaughnessy et al. (2017), Ortiz and Lalana (2018), and Abdelsalam and Ramadan (2019).

**Suborder Sphaeromatidea**

**Superfamily Seroloidea**

#### ﻿Family Serolidae

*Heteroseroliscarinata* (Lockington, 1877) [Fig. 18A] Intertidal to continental shelf, soft bottom benthos (0–98 m); Santa Monica Bay, southern California, USA to San Quintin Bay, Baja California Norte, Mexico, and the Gulf of California. Type locality: USA, California, San Diego. Body length to ~ 6.4 mm. See Lockington (1877a original description as *Seroliscarinatus*), Richardson (1899, 1900b, 1905a), Menzies and Barnard (1959), Menzies (1962a), Schultz (1969), Hessler (1972), Glynn (1976), Dexter (1983), Lissner et al. (1986), SCAMIT (1994–2021), Bergen et al. (1998), Espinosa-Pérez and Hendrickx (2001a, 2006), Smith et al. (2001, 2003), Ranasinghe et al. (2003, 2007, 2012), McLaughlin et al. (2005), Reed and Hovel (2006), Sirota and Hovel (2006), Brusca et al. (2007), Campos and Villarreal (2008), Melwani and Kim (2008), Bruce (2009b), and Gillett et al. (2017). Note: See Endnote 13.

**Superfamily Sphaeromatoidea**

#### ﻿Family Ancinidae

*Ancinusgranulatus* Holmes & Gay, 1909 [Fig. 19A] Intertidal to shallow subtidal (0–10 m); Santa Barbara, southern California, USA to the Gulf of California, Mexico. Type locality: USA, California, near Coronado Island from depth of 3 fathoms. Body length to ~ 9.5 mm. See Holmes and Gay (1909), Loyola e Silva (1971), Schultz (1973), Glynn and Glynn (1974), Pires (1987), SCAMIT (1998–2021), Espinosa-Pérez and Hendrickx (2001a, 2006), Ranasinghe et al. (2003, 2007), Brusca et al. (2004, 2007), McLaughlin et al. (2005), Campos and Villarreal (2008), and Rocha-Ramírez et al. (2010). Note: See Endnote 14.

*Ancinusseticomvus* Trask, 1971 [Fig. 19B] Low intertidal on sandy beaches; Santa Barbara, southern California, USA to Mazatlán, Mexico, and the Gulf of California. Type locality: USA, California, Santa Barbara County, Coal Oil Beach (~ 34°28'N; 119°50'W). Body length to 10.5 mm. See Trask (1971), Schultz (1973), Glynn and Glynn (1974), Dexter (1976), Iverson (1982), Iverson and Chivers (1984), Pires (1987), Wetzer et al. (1991), McLaughlin et al. (2005), and Rocha-Ramírez et al. (2010). Note: *Ancinusseticomvus* is listed as a junior synonym of *A.granulatus* in SCAMIT (1998–2021) and Brusca et al. (2007). See Endnote 14 for a summary of the taxonomic history of *A.seticomvus*.

*Bathycopeadaltonae* (Menzies & Barnard, 1959) [Fig. 19C] Sandy subtidal sediments (17–137 m); Columbia River Estuary, Washington State to San Miguel Island and White’s Point, Los Angeles County, southern California. USA. Type locality: USA, California, off Point Conception (R/V Velero IV Station No. 4819-57; 34°26'30"N, 120°28'10"W). Body length to ~ 4 mm. See Menzies and Barnard (1959 original description as *Ancinusdaltonae*), Loyola e Silva (1971), Schultz (1973), Iverson and Chivers (1984), Miller (1975), Higley and Holton (1978), Kussakin (1979), Wetzer et al. (1991), SCAMIT (2001–2021), Ranasinghe et al. (2003), McLaughlin et al. (2005), Espinosa-Pérez and Hendrickx (2006), Melwani and Kim (2008), Shimomura (2008), and Henkel et al. (2020).

#### ﻿Family Sphaeromatidae

*Discerceisgranulosa* (Richardson, 1899) [Fig. 20C] Subtidal (37 m); southern California, USA to Cedros Island, Baja California Norte, Mexico. Type locality: Mexico, Baja California, Cedros Island. Body length to ~ 9 mm. See Richardson (1899 original description as *Cilicaeagranulosa*; 1905a, 1906), Schultz (1969), SCAMIT (1994–2021), McLaughlin et al. (2005), Schotte (2005), and Espinosa-Pérez and Hendrickx (2006).

*Dynamenelladilatata* (Richardson, 1899) [Fig. 22C] Intertidal; Tatoosh Island, Washington to southern California, USA. Type locality: USA, California, Monterey Bay. Body length to ~ 3 mm. See Richardson (1899 original description as *Dynamenedilatata*; 1900b, 1905a), Shearer (1942), Hewatt (1946), Hatch (1947), Schultz (1969), Miller (1975), Kussakin (1979), Lissner et al. (1986), McLaughlin et al. (2005), Espinosa-Pérez and Hendrickx (2006), Brusca et al. (2007), Espinosa-Pérez et al. (2009), SCAMIT (2011–2021), and Lafferty and Suchanek (2016). Note: See Endnote 17 regarding generic assignment of this species.

*Dynamenellaglabra* (Richardson, 1899) [Fig. 22D] Intertidal; Coos Bay, Oregon to San Diego, southern California, USA. Type locality: USA, California, Monterey Bay. Body length to ~ 3 mm. See Richardson (1899 original description as *Dynameneglabra*; 1900b, 1905a), Shearer (1942), Hewatt (1946), Hatch (1947), Schultz (1969), Miller (1975), Kussakin (1979), Chess and Hobson (1997), McLaughlin et al. (2005), Espinosa-Pérez and Hendrickx (2006), Brusca et al. (2007), Espinosa-Pérez et al. (2009), and SCAMIT (2011–2021). Note: See Endnote 17 regarding generic assignment of this species.

*Dynamenellasheareri* (Hatch, 1947) [Fig. 22E] Intertidal to shallow subtidal; San Juan Archipelago, Washington to southern California, USA. Type locality: USA, Oregon, Coos Bay. Body length to ~ 3.3 mm. See Hatch (1947 original description as *Dynamenesheareri*), George and Strömberg (1968), Schultz (1969), Miller (1975), Kussakin (1979), McLaughlin et al. (2005), Espinosa-Pérez and Hendrickx (2006), Toft and Cordell (2006), Brusca et al. (2007), Toft et al. 2008, 2010), Espinosa-Pérez et al. (2009), SCAMIT (2011–2021), and Lafferty and Suchanek (2016).

*Dynoideselegans* (Boone, 1923) [Fig. 22A] Intertidal; southern California, USA from Santa Barbara County to San Diego, and possibly further south to Cedros Island, Baja California, Mexico. Type locality: USA, California, San Diego County, La Jolla, Scripps Institution of Oceanography (~ 32.27°N, 117.61°W). Body length to ~ 7.4 mm. See Boone (1923 original description as *Clianellaelegans*), Hewatt (1946), Li (2000), McLaughlin et al. (2005), Espinosa-Pérez and Hendrickx (2006), Wetzer and Mowery (2017), SCAMIT (2018–2021), and Khalaji-Pirbalouty and Gagnon (2021). Note: SCB records of *Dynoideselegans* identified as *D.saldani* in SCAMIT (2012–2016).

*Exosphaeromaamplicauda* (Stimpson, 1857) [Fig. 23A] Intertidal; Marin, Sonoma, and San Mateo Counties, northern California, USA (as redefined by Wall et al. 2015). Type locality (neotype): USA, California, Marin County, Tomales Bay (38.201°N, 122.922°W). Body length to ~ 8.4 mm. See Stimpson (1857 original description as *Sphaeromaamplicauda*), Richardson (1900b, 1905a), Hewatt (1946), George and Strömberg (1968), Miller (1975), Allen (1976), Kussakin (1979), Lee and Miller (1980), Bruce (2003), McLaughlin et al. (2005), Espinosa-Pérez and Hendrickx (2006), Brusca et al. (2007), Espinosa-Pérez et al. (2009), SCAMIT (2011–2021), Wall et al. (2015), Lafferty and Suchanek (2016), and Gillett et al. (2022). Note: See Endnote 18 regarding restricted range of *E.amplicauda* based on review by Wall et al. (2015).

*Exosphaeromaaphrodita* Boone, 1923 [Fig. 23B] Intertidal to shallow subtidal, on pier pilings and amongst detritus at the base of pilings; La Jolla, southern California, USA. Type locality (lectotype): USA, California, San Diego County, La Jolla, Scripps Institution of Oceanography pier pilings. Body length to ~ 8.7 mm. See Boone (1923), Bruce (2003), McLaughlin et al. (2005), Espinosa-Pérez and Hendrickx (2006), Wall et al. (2015), and SCAMIT (2016–2021). Note: See Endnote 18.

*Exosphaeromainornata* Dow, 1958 [Fig. 23C] Intertidal to shallow subtidal, in holdfasts of the kelp *Macrocystispyrifera*; Puget Sound, Washington, USA to Todos Santos Bay, Baja California Norte, Mexico. Type locality: USA, California, Los Angeles County, San Pedro, Point Fermin (~ 33°40'N, 118°20'W). Body length to ~ 6 mm. See Dow (1958), Schultz (1969), Iverson (1974, 1978, 1982), Miller (1975), Wetzer et al. (1991), Bruce (2003), McLaughlin et al. (2005), Espinosa-Pérez and Hendrickx (2006), Brusca et al. (2007), Campos and Villarreal (2008), Espinosa-Pérez et al. (2009), SCAMIT (2011–2021), Puget Sound Institute (2012–2023), Roletto et al. (2014), and Wall et al. (2015).

*Exosphaeromapentcheffi* Wall, Bruce & Wetzer, 2015 [Fig. 23D] High to low rocky intertidal, amongst barnacles, algal turf, and *Phragmatopoma* worm tubes on undersides of rocks; Palos Verdes, southern California, USA. Type locality: USA, California, Los Angeles County, Palos Verdes Peninsula, Point Fermin (33.71°N, 118.3°W). Body length to ~ 6.8 mm. See Wall et al. (2015) and SCAMIT (2015–2021). Note: Records of *E.pentcheffi* reported as provisional species *Exosphaeroma* sp. A in SCAMIT (2012–2014).

*Exosphaeromarhomburum* (Richardson, 1899) [Fig. 23E] Intertidal to 26 m; Tatoosh Island, Washington, USA to Baja California Norte, Mexico (~ 4.3–6.7 km south of the US-Mexico border). Type locality: USA, California, Monterey Bay. Body length to ~ 4 mm. See Richardson (1899 original description as *Sphaeromarhomburum*; 1900b, 1905a), Shearer (1942), Schultz (1969), Miller (1975), Kussakin (1979), Hobson and Chess (1986), SCAMIT (1994–2021), Chess and Hobson (1997), Bruce (2003), Ranasinghe et al. (2003), McLaughlin et al. (2005), Espinosa-Pérez and Hendrickx (2006), Brusca et al. (2007), Wall et al. (2015), Lafferty and Suchanek (2016), and Looby and Ginsburg (2021). Note: See Endnote 19.

*Gnorimosphaeromanoblei* Menzies, 1954 [Fig. 20A] High intertidal, brackish to fully freshwater habitats; Del Norte County, northern California to Palos Verdes, Los Angeles County, southern California, USA. Type locality: USA, California, Marin County, Tomales Bay, Marshall (38.162°N, 122.89°W). Body length to ~ 2.9 mm. See Menzies (1954), Schultz (1969), Iverson (1974), Miller (1975), Kussakin (1979), Ricketts et al. (1985), McLaughlin et al. (2005), Espinosa-Pérez and Hendrickx (2006), Brusca et al. (2007), Espinosa-Pérez et al. (2009), and Wetzer et al. (2021).

*Gnorimosphaeromaoregonense* (Dana, 1853) [Fig. 20B] Intertidal to subtidal (0–21 m); Vancouver, British Columbia, Canada to San Francisco, California, USA. Type locality (neotype; see Wetzer et al. 2021): Canada, British Columbia, Stanley Park (49.294°N, 123.155°W). Body length to ~ 8.5 mm. See Dana (1853 original description as *Spheromaoregonensis*), Richardson (1899, 1900b, 1905a), Menzies (1954), George and Strömberg (1968), Miller (1968), Schultz (1969), Miller (1975), Higley and Holton (1978), Kussakin (1979), Ricketts et al. (1985), McLaughlin et al. (2005), Espinosa-Pérez and Hendrickx (2006), Brusca et al. (2007), Ranasinghe et al. (2007), Espinosa-Pérez et al. (2009), SCAMIT (2011–2021), Puget Sound Institute (2012–2023), Lafferty and Suchanek (2016), Gillett et al. (2017), and Wetzer et al. (2021). Note: See Endnote 15 regarding distribution of *G. oregonense*.

*Paracerceiscordata* (Richardson, 1899) [Fig. 21A] Intertidal to shallow subtidal, on coralline algae and kelp holdfasts; Aleutian Islands to southern California, USA. Type locality: Aleutian Islands, Popoff Island. Body length to ~ 7.1 mm. See Richardson (1899 original description as *Cilicaeacordata*; 1900b, 1905a), Hewatt (1946), Schultz (1969), Miller (1975), Kussakin (1979), Lee and Miller (1980), SCAMIT (1994–2021), Chess and Hobson (1997), O’Clair and O’Clair (1998), Fairey et al. (2002), McLaughlin et al. (2005), Espinosa-Pérez and Hendrickx (2006), Brusca et al. (2007), Espinosa-Pérez et al. (2009), and Roletto et al. (2014).

*Paracerceisgilliana* (Richardson, 1899) [Fig. 21B] Subtidal, in sandy mud, and in bays on pilings, among mussel beds, and in association with hydroids, algae and ectoprocts (shallow to 73 m); Mendocino County, northern California, USA to Catalina Island, southern California, and south to Todos Santos Bay, Baja California Norte, Mexico. Type locality: USA, California, Catalina Island. Body length to ~ 4.8 mm. See Richardson (1899 original description as *Cilicaeacaudatagilliana*; 1905a), Schultz (1969), Allen (1976), McLaughlin et al. (2005), Espinosa-Pérez and Hendrickx (2006), Campos and Villarreal (2008), and Espinosa-Pérez et al. (2009).

*Paracerceissculpta* (Holmes, 1904) [Fig. 21C] Intertidal to shallow subtidal; Morro Bay, central California to San Diego, southern California, USA, and south to San Juan Alima, Michoacan, Mexico, including the entire Gulf of California, and also distributed throughout the world via oceanic shipping. Type locality: USA, California, from pieces of sponge dredged in shallow water at San Clemente Island. Body length to ~ 7.5 mm. See Holmes (1904 original description as *Dynamenesculpta*), Richardson (1905a), Menzies (1962a), Miller (1968), Schultz (1969), Brusca (1980), Shuster (1987, 1992), Shuster and Wade (1991a, 1991b), Rodríguez et al. (1992), SCAMIT (1994–2021), Shuster and Guthrie (1999), Espinosa-Pérez and Hendrickx (2001a, 2001b, 2006), Hewitt and Campbell (2001), Fairey et al. (2002), Smith et al. (2003), Ariyama and Otani (2004), Ranasinghe et al. (2003, 2005, 2007, 2012), Brusca et al. (2004, 2007), Healey and Hovel (2004), McLaughlin et al. (2005), Whitmore et al. (2005), Reed and Hovel (2006), Sirota and Hovel (2006), Montelli and Lewis (2008), Cruz-García et al. (2013), Munguia and Shuster (2013), Morales-Zarate et al. (2016), Gillett et al. (2017, 2022), Ramalhosa et al. (2017), Marchini et al. (2018), Rumbold et al. (2018), and Wetzer et al. (2018).

*Paracerceis* sp. A [Fig. 21D] Intertidal; La Jolla, southern California, USA to the central Gulf of California, Mexico. Body length = no information available. See Brusca (1980: species listed as *Paracerceis* sp.), SCAMIT (2008–2021), and Gillett et al. (2022). Note: Provisional species voucher sheet available at https://www.scamit.org (SCAMIT Newsletter, Vol. 15, No. 8, December 1996).

*Paradelladianae* (Menzies, 1962) [Fig. 22B] Intertidal to shallow subtidal; Ventura County, southern California, USA to San Juan de Alima, Michoacan, Mexico, including the Gulf of California, and widely introduced to ports around the world by international shipping. Type locality: Mexico, Baja California, Bahia de San Quintin. Body length to ~ 4 mm. See Menzies (1962a original description as *Dynamenopsisdianae*), Menzies and Glynn (1968), Schultz (1969), Iverson (1974), Allen (1976), Pires (1980), Kensley and Schotte (1989), Rodríguez et al. (1992), Espinosa-Pérez and Hendrickx (2001a, 2006), Bruce and Wetzer (2004), McLaughlin et al. (2005), Brusca et al. (2007), Wetzer and Bruce (2007), Campos and Villarreal (2008), and SCAMIT (2008–2021).

*Pseudosphaeroma* sp. [No figure] Intertidal, bays and harbors; San Francisco and central California Coast regions, and Salinas de San Pedro, Los Angeles County, southern California, USA. Body length to 5.2 mm. See Bruce and Wetzer (2008) and references therein (e.g., Hurley and Jansen 1977; Harrison 1984), and additional information available from R Wetzer (unpublished results). Note: Species probably translocated to the SCB from the southern hemisphere (e.g., likely New Zealand). The most similar species is *P.campbellense*, but the present species concept includes more than one species (see Bruce and Wetzer 2008).

*Sphaeromaquoianum* H. Milne Edwards, 1840 [Fig. 20D] Intertidal to shallow subtidal, boring in wood, mud, and soft rocks; Humboldt County, northern California, USA to San Quintin Bay, Baja California Norte, Mexico, and in Australia and New Zealand (i.e., probably introduced to western North America in the late 1800’s on ships from Australia). Type locality: Uncertain (i.e., not indicated in original description), but listed as Tasmania, Australia in Boyko et al. (2008 onwards). Body length to ~ 9.9 mm. See H. Milne Edwards (1840), Menzies (1962a, as *Sphaeromapentodon*), Rotramel (1972), Iverson (1974), Carlton (1979), Kussakin (1979), Lee and Miller (1980), Carlton and Iverson (1981), Ricketts et al. (1985), Talley et al. (2001), McLaughlin et al. (2005), Espinosa-Pérez and Hendrickx (2006), Brusca et al. (2007), Davidson and de Rivera (2010), Davidson et al. (2010), SCAMIT (2011–2021), and Brown et al. (2022).

*Sphaeromawalkeri* Stebbing, 1905 [Fig. 20E] Intertidal to shallow water, common in fouling communities in warm to warm-temperate ports and harbors, and boring into wood or soft rocks; introduced species with a worldwide distribution, but occurring in at least San Diego Bay, southern California, USA. Type locality: Sri Lanka. Body length to ~ 9.5 mm. See Stebbing (1905), Menzies and Glynn (1968), Carlton and Iverson (1981), Mak et al. (1985), Kensley and Schotte (1989), Cohen et al. (2005), McLaughlin et al. (2005), Espinosa-Pérez and Hendrickx (2006), Brusca et al. (2007), Galil (2008), Montelli and Lewis (2008), Khalaji-Pirbalouty and Wägele (2010), Ramalhosa et al. (2017), and Ortiz and Lalana (2018).

#### ﻿Family Tecticipitidae

*Tecticepsconvexus* Richardson, 1899 [Fig. 18B] Low intertidal to shallow subtidal, on sandy, rocky bottoms; southern Washington State (northwest of Columbia River mouth) to Catalina Island, southern California, USA. Type locality: USA, California, Monterey Bay. Body length to ~ 11.5 mm. See Richardson (1899, 1900b, 1905a, 1906), Iwasa (1934), Schultz (1969), Iverson (1974), Miller (1975), Kuris and Carlton (1977), Higley and Holton (1978), Kussakin (1979), Lee and Miller (1980), Hinton and Emmett (1994, 1996), Hunt et al. (1999), McLaughlin et al. (2005), Espinosa-Pérez and Hendrickx (2006), Brusca et al. (2007), Espinosa-Pérez et al. (2009), and Looby and Ginsburg (2021).

**Suborder Valvifera**

#### ﻿Family Arcturidae

*Idarcturusallelomorphus* Menzies & Barnard, 1959 [Fig. 25A] Continental shelf, soft-bottom substrates (12–94 m); Monterey, California, USA to Todos Santos Bay, Baja California Norte, Mexico. Type locality: USA, California, off Goleta (R/V Velero IV Station No. 4938-57; 34°27'25"N, 120°12'55"W). Body length to ~ 5.2 mm. See Menzies and Barnard (1959), Schultz (1969), Iverson (1974), Gillard (1978), Kussakin (1982), Dexter (1983), Lissner et al. (1986), Wetzer et al. (1991), SCAMIT (1994–2021), Wetzer and Brusca (1997), Bergen et al. (1998), Smith et al. (2001, 2003), Ranasinghe et al. (2003, 2007, 2012), McLaughlin et al. (2005), Espinosa-Pérez and Hendrickx (2006), Campos and Villarreal (2008), Espinosa-Pérez et al. (2009), Stebbins (2012a, 2012c), Gillett et al. (2017, 2022), and Kim and Yoon (2021).

*Idarcturushedgpethi* Menzies, 1951 [Fig. 25B] Intertidal, on hydroids in laminarian zone at type locality; Strait of Georgia, British Columbia, Canada to Port Hueneme, southern California, USA. Type locality: USA, California, Marin County, Tomales Point, Tomales Bluff. Body length to ~ 4.2 mm. See Menzies (1951b), Schultz (1969), Miller (1975), Kussakin (1982), Wetzer and Brusca (1997), Fairey et al. (2002), McLaughlin et al. (2005), Espinosa-Pérez and Hendrickx (2006), Brusca et al. (2007), SCAMIT (2008–2021), Macdonald et al. (2010), Stebbins (2012a, 2012c), Gillett et al. (2017), and Kim and Yoon (2021). Note: See Endnote 20.

*Idarcturus* sp. A [No figure] Continental shelf, soft bottom substrates (18–95 m); northern Channel Islands and Western Santa Barbara Channel, southern California, USA. Body length to ~ 5.5 mm. Note: See Endnote 20.

*Neastacillacalifornica* (Boone, 1918) [Fig. 25C] Continental shelf, on seaweed and soft bottom benthos (subtidal to 99 m); Point Conception, California, USA to Guerrero Negro Lagoon, Baja California Sur, Mexico, and in the Gulf of California. Type locality: USA, California, Venice. Body length to ~ 7.4 mm. See Boone (1918 original description as *Astacillacalifornica*), Menzies and Barnard (1959), Schultz (1969), Kussakin (1982), Thompson et al. (1993), SCAMIT (1994–2021), Hendrickx et al. (1997), Bergen et al. (1998), Espinosa-Pérez and Hendrickx (2001a, 2006), Smith et al. (2001, 2003), King (2003), Ranasinghe et al. (2003, 2007, 2012), McLaughlin et al. (2005), Melwani and Kim (2008), Stebbins (2012a, 2012c), Morales-Zarate et al. (2016), Gillett et al. (2017, 2022), and Mejía-Rebollo et al. (2018).

#### ﻿Family Holognathidae

*Cleantioidesoccidentalis* (Richardson, 1899) [Fig. 25E] Low intertidal to subtidal, typically on sand and muddy-sand substrates (0–50 m); southern California, USA to Baja California Sur, Mexico, throughout the Gulf of California, and south to Columbia and Ecuador, including the Galapagos Islands. Type locality: Mexico, Baja California Sur, Magdalena Bay. Body length to ~ 12.5 mm. See Richardson (1899 original description as *Cleantisoccidentalis*; 1905a), Schultz (1969), Kensley and Kaufman (1978), Brusca and Wallerstein (1979a, 1979b), Brusca and Iverson (1985), Poore and Lew Ton (1990), Espinosa-Pérez and Hendrickx (2001a, 2002, 2006), McLaughlin et al. (2005), Brusca et al. (2007), Lazarus-Agudelo and Cantera-Kintz (2007), SCAMIT (2011–2021), Ranasinghe et al. (2012), Liu and Poore (2013), and Negromonte et al. (2022).

#### ﻿Family Idoteidae

*Colidoteafindleyi* Brusca & Wallerstein, 1977 [Fig. 27C] Low intertidal to shallow subtidal, common on fronds of the brown algae *Sargassum*; San Diego, California, USA (rare) to Guadalupe Island, Baja California Norte, and the Gulf of California, Mexico. Type locality: Mexico, Sonora, 5 miles north of Cabo Tepoca (Puerto Lobos) (~ 30°17'N; 112°50'W). Body length to ~ 24.5 mm. See Brusca and Wallerstein (1977, 1979b, 1982), Brusca (1980, 1983b), Wetzer et al. (1991), Espinosa-Pérez and Hendrickx (2001a, 2002, 2006), Brusca et al. (2004, 2007), Campos and Villarreal (2008), and Stebbins (2012a, 2012c).

*Colidotearostrata* (Benedict, 1898) [Fig. 27D] Low intertidal to shallow subtidal, living as obligate commensal on sea urchins *Strongylocentrotuspurpuratus* and *Mesocentrotusfranciscanus*; Coos Bay, Oregon, USA to Baja California Norte, Mexico, but rare north of southern California. Type locality: USA, California, San Pedro. Body length to ~ 13.7 mm. See Benedict (1898 original description as *Idotearostrata*), Richardson (1899, 1900b, 1905a), Shearer (1942), Hewatt (1946), Johnson and Snook (1967), Schultz (1969), Allen (1976), Brusca and Wallerstein (1979b), Lee and Miller (1980); Ricketts et al. (1985), Stebbins (1988a, 1988b, 1989, 2012a, 2012c), McLaughlin et al. (2005), Espinosa-Pérez and Hendrickx (2006), Brusca et al. (2007), and SCAMIT (2012–2021).

*Colidoteawallersteini* Brusca, 1983 [Fig. 27E] Intertidal; known only from two localities off the coast of Baja California Norte (i.e., Punta Clara and Guadalupe Island), Mexico. Type locality: Mexico, Baja California Norte, Punta Clara, south Rio Santo Tomás. Body length to ~ 16.5 mm. See Brusca (1983b), Wetzer et al. (1991), Espinosa-Pérez and Hendrickx (2002, 2006), McLaughlin et al. (2005), and Stebbins (2012a, 2012c).

*Edotiasublittoralis* Menzies & Barnard, 1959 [Fig. 27A] Continental shelf, typically occurring in sandy-silt benthic sediments (10–46 m, and one record > 100 m off San Diego); Vancouver Island, British Columbia to western Baja California Norte, Mexico, the Gulf of California (Bahia Concepcion), and one record off Costa Rica. Type locality: USA, California, off Newport (R/V Velero IV Station No. 4720-56; 33°37'39"N, 117°58'16"W). Body length to ~ 5.8 mm. See Menzies and Barnard (1959 original description as *Edoteasublittoralis*), Schultz (1969), Iverson (1974), Miller (1975), Brusca and Wallerstein (1979b), Kussakin (1982), Vargas-Zamora et al. (1985), Rafi and Laubitz (1990), Wetzer et al. (1991), Thompson et al. (1993), Hinton and Emmett (1994, 1996), SCAMIT (1994–2021), Espinosa-Pérez and Hendrickx (2001a, 2006), Ranasinghe et al. (2003, 2007, 2012), McLaughlin et al. (2005), Campos and Villarreal (2008), Melwani and Kim (2008), Stebbins (2012a, 2012c), Lafferty and Suchanek (2016), Gillett et al. (2017, 2022), and Henkel et al. (2020). Note: See Endnote 21.

*Edotia* sp. B [Fig. 27B] Continental shelf, typically occurring in sandy-silt benthic sediments (24–62 m); southern California, USA to Baja California Norte, Mexico. Body length to ~ 4.1 mm. See Menzies and Barnard (1959, as *Edoteasublittoralis* in part), SCAMIT (1994–2021), Ranasinghe et al. (2003, 2007, 2012), Stebbins (2012a, 2012c), and Gillett et al. (2017). Note: See Endnote 21.

*Erichsonellacrenulata* Menzies, 1950 [Fig. 26B] Intertidal to shallow subtidal, typically occurring on the eelgrass *Zosteramarina*: Newport Bay, southern California to San Quintin Bay, Baja California Norte, Mexico. Type locality: USA, California, Orange County, Newport Bay. Body length to ~ 23 mm. See Menzies (1950b, 1962a), Schultz (1969), Allen (1976), Pires (1984), Wetzer et al. (1991), McLaughlin et al. (2005), Espinosa-Pérez and Hendrickx (2006), Brusca et al. (2007), Jorgensen et al. (2007), Ranasinghe et al. (2007, 2012), Campos and Villarreal (2008), SCAMIT (2008–2021), Stebbins (2012a, 2012c), and Gillett et al. (2017, 2022).

*Eusymmeruspseudoculata* (Boone, 1923) [Fig. 26C] Intertidal to subtidal (0–18 m); Point Conception, California to US-Mexico border. Type locality: USA, California, San Pedro. Body length to ~ 9 mm. See Boone (1923 original description as *Erichsonellapseudoculata*), Menzies and Bowman (1956 redescription as *Ronaleapseudoculata*), Menzies and Barnard (1959), Schultz (1969), Kussakin (1982), McLaughlin et al. (2005), Espinosa-Pérez and Hendrickx (2006), Brusca et al. (2007), Espinosa-Pérez et al. (2009), SCAMIT (2012–2021), and Stebbins (2012a, 2012c).

*Idoteafewkesi* Richardson, 1905 [Fig. 29A] Intertidal to shallow water (0–6 m); Alaska, USA to Guerrero Negro, Baja California Sur, Mexico. Type locality: USA, California, Santa Barbara. Body length to ~ 42 mm. See Richardson (1905a), Fee (1927), Shearer (1942), Hatch (1947), Menzies (1950a), Schultz (1969), Miller (1975), Brusca and Wallerstein (1979b), Lee and Miller (1980), Kussakin (1982), Rafi and Laubitz (1990), O’Clair and O’Clair (1998), SCAMIT (2001–2021), McLaughlin et al. (2005), Espinosa-Pérez and Hendrickx (2006), Brusca et al. (2007), Campos and Villarreal (2008), Espinosa-Pérez et al. (2009), Puget Sound Institute (2012–2023), Stebbins (2012a, 2012c), Roletto et al. (2014), Morales-Zarate et al. (2016), and Shaughnessy et al. (2017).

*Idoteametallica* Bosc, 1801 [Fig. 29B] Cosmopolitan pelagic species found attached to drifting seaweed, occasionally occurring in southern California and Gulf of California during warm water years. Type locality: Unknown to us (not indicated in original description). Body length to ~ 29 mm. See Bosc (1801), Miers (1881), Richardson (1900b, 1905a, 1909), Naylor (1957), Schultz (1969), Kussakin (1982), Poore and Lew Ton (1993), Franke et al. (1998), Abelló et al. (2004), McLaughlin et al. (2005), Espinosa-Pérez and Hendrickx (2006), Brusca et al. (2007), and Riera (2014). Note: Poore and Lew Ton (1993) provide a comprehensive synonymy and figures for *I.metallica*.

*Idotearufescens* Fee, 1927 [Fig. 29C] Low intertidal to subtidal (0–82 m), occurring mostly on algae; Prince Williams Sound, Alaska, USA to western Baja California, Mexico, including Catalina and the Coronado Islands. Type locality: Canada, British Columbia, Gabriola Passage. Body length to ~ 20.6 mm. See Fee (1927), Hatch (1947), Menzies (1950a), Schultz (1969), Iverson (1974), Miller (1975), Kussakin (1982), Rafi and Laubitz (1990), Wetzer and Brusca (1997), O’Clair and O’Clair (1998), McLaughlin et al. (2005), Espinosa-Pérez and Hendrickx (2006), Brusca et al. (2007), SCAMIT (2008–2021), Macdonald et al. (2010), Stebbins (2012a, 2012c), and Shaughnessy et al. (2017).

*Idoteaurotoma* Stimpson, 1864 [Fig. 29D] Intertidal to shallow subtidal (0–14 m); Alaska, USA to Todos Santos Bay, Baja California Norte, Mexico, and the Gulf of California. Type locality: USA, Washington, Puget Sound. Body length to ~ 26.5 mm. See Stimpson (1864), Richardson (1899, 1900b, 1905a), Shearer (1942), Hewatt (1946), Hatch (1947), Menzies (1950a), George and Strömberg (1968), Schultz (1969), Miller (1975), Allen (1976), Brusca and Wallerstein (1977, 1979b, 1982), Brusca (1980), Lee and Miller (1980), Kussakin (1982), Dexter (1983), Ricketts et al. (1985), Rafi and Laubitz (1990), O’Clair and O’Clair (1998), Espinosa-Pérez and Hendrickx (2001a, 2006), SCAMIT (2001–2021), McLaughlin et al. (2005), Brusca et al. (2007), Campos and Villarreal (2008), Macdonald et al. (2010), Stebbins (2012a, 2012c), and Roletto et al. (2014).

*Pentidoteaaculeata* Stafford, 1913 [Fig. 30A] Intertidal; occurring in various habitats, including encrusting red algae (e.g., *Melobesia*) on the surfgrass *Phyllospadix*: British Columbia, Canada to Cedros Island, Baja California Norte, Mexico, and the Gulf of California. Type locality: USA, California, Laguna Beach, low tide. Body length to ~ 23 mm. See Stafford (1913b), Hewatt (1946), Menzies (1950a), George and Strömberg (1968), Schultz (1969), Miller (1975), Allen (1976), Brusca and Wallerstein (1977, 1979b, 1982), Brusca (1980), Lee and Miller (1980), Kussakin (1982), Espinosa-Pérez and Hendrickx (2001a, 2006), McLaughlin et al. (2005), Brusca et al. (2007), Campos and Villarreal (2008), SCAMIT (2011–2021), and Stebbins (2012a, 2012c).

*Pentidoteakirchanskii* (Miller & Lee, 1970) [Fig. 30B] Intertidal, commonly on surfgrass *Phyllospadix*; Oregon to southern California, USA. Type locality: USA, California, Monterey County, Pebble Beach, 17 Mile Drive at Seal Rock. Body length to ~ 21 mm. See Miller and Lee (1970 original description as Idothea (Pentidotea) kirchanskii), Miller (1975), Brusca and Wallerstein (1979b), Lee and Miller (1980), Kussakin (1982), McLaughlin et al. (2005), Espinosa-Pérez and Hendrickx (2006), Brusca et al. (2007), and Stebbins (2012a, 2012c).

*Pentidoteamontereyensis* Maloney, 1933 [Fig. 30C] Intertidal to subtidal (0–109 m), common on surfgrass (*Phyllospadix* spp.) in shallow waters; Alaska, USA to western Baja California, Mexico. Type locality: USA, California, Monterey Bay. Body length to ~ 28.3 mm. See Maloney (1933), Menzies (1950a), Schultz (1969), Iverson (1974), Miller (1975), Brusca and Wallerstein (1979b), Lee and Miller (1980), Kussakin (1982), Ricketts et al. (1985), Rafi and Laubitz (1990), SCAMIT (1994–2021), O’Clair and O’Clair (1998), McLaughlin et al. (2005), Espinosa-Pérez and Hendrickx (2006), Brusca et al. (2007), and Stebbins (2012a, 2012c).

*Pentidotearesecata* (Stimpson, 1857) [Fig. 30D] Intertidal to subtidal, frequently occurring in kelp and eelgrass beds; Alaska, USA to western Baja California Sur, Mexico, and to Mazatlán in the Gulf of California. Type locality: USA, Washington, Straits of Juan de Fuca, opposite Fort Townsend. Body length to ~ 57 mm. See Stimpson (1857 original description as *Idotearesecata*), Richardson (1899, 1900b, 1905a), Fee (1927), Hewatt (1946), Hatch (1947), Menzies and Waidzunas (1948), Menzies (1950a), George and Strömberg (1968), Schultz (1969), Iverson (1974), Miller (1975), Allen (1976), Brusca and Wallerstein (1977, 1979b, 1982), Brusca (1980), Lee and Miller (1980), Kussakin (1982), Ricketts et al. (1985), Rafi and Laubitz (1990), SCAMIT (1994–2021), Chess and Hobson (1997), O’Clair and O’Clair (1998), Espinosa-Pérez and Hendrickx (2001a, 2002, 2006), McLaughlin et al. (2005), Brusca et al. (2007), Campos and Villarreal (2008), Stebbins (2012a, 2012c), Roletto et al. (2014), and Shaughnessy et al. (2017).

*Pentidoteaschmitti* (Menzies, 1950) [Fig. 30E] Intertidal to subtidal (0–73 m); Bering Sea to Punta Banda, Baja California Norte, Mexico. Type locality: USA, California, Marin County, Dillon Beach, Second Sled Road. Body length to ~ 35 mm. See Menzies (1950a original description as Idothea (Pentidotea) schmitti), Schultz (1969), Iverson (1974), Miller (1975), Brusca and Wallerstein (1979b), Kussakin (1982), Ricketts et al. (1985), Rafi and Laubitz (1990), Wetzer et al. (1991), O’Clair and O’Clair (1998), McLaughlin et al. (2005), Espinosa-Pérez and Hendrickx (2006), Brusca et al. (2007), Stebbins (2012a, 2012c), Roletto et al. (2014), and Lafferty and Suchanek (2016).

*Pentidoteastenops* (Benedict, 1898) [Fig. 30F] Intertidal to shallow subtidal, often occurring on the feather-boa kelp *Egregiamenziesii*, surfgrass (*Phyllospadix* spp.), and algae-covered rocks; Alaska, USA to San Eugenio Point, Baja California Sur, Mexico, and the Gulf of California. Type locality: USA, California, Monterey. Body length to 60 mm. See Benedict (1898 original description as *Idoteastenops*), Richardson (1899, 1900b, 1905a), Shearer (1942), Hatch (1947), Menzies (1950a), George and Strömberg (1968), Schultz (1969), Iverson (1974), Miller (1975), Allen (1976), Brusca and Wallerstein (1977, 1979b, 1982), Brusca and Brusca (1978), Brusca (1980), Lee and Miller (1980), Kussakin (1982), Ricketts et al. (1985), O’Clair and O’Clair (1998), Espinosa-Pérez and Hendrickx (2001a, 2006), McLaughlin et al. (2005), Brusca et al. (2007), Campos and Villarreal (2008), Stebbins (2012a, 2012c), and Roletto et al. (2014).

*Pentidoteawosnesenskii* (Brandt, 1851) [Fig. 30G] Intertidal to subtidal (0–16 m); Aleutian Islands, Alaska, USA to southern California, plus a single record from off La Paz, Mexico in the Gulf of California. Type locality: Listed in Brandt (1851) as more than one site, including Sea of Okhotsk, Attu, Atcha, St. Paul, Kadjak, Sitka and the northern California coast. Body length to ~ 40.2 mm. See Brandt (1851 original description as *Idoteawosnesenskii*), Richardson (1899, 1900b, 1905a), Fee (1927), Shearer (1942), Hatch (1947), Menzies (1950a), George and Strömberg (1968), Schultz (1969), Miller (1975), Brusca and Wallerstein (1977, 1979b, 1982), Brusca (1980), Lee and Miller (1980), Kussakin (1982), Ricketts et al. (1985), Rafi and Laubitz (1990), O’Clair and O’Clair (1998), Espinosa-Pérez and Hendrickx (2001a, 2006), McLaughlin et al. (2005), Brusca et al. (2007), Toft et al. (2008, 2010), Stebbins (2012a, 2012c), Roletto et al. (2014), and Lafferty and Suchanek (2016).

*Stenosomawetzerae* (Ormsby, 1991) [Fig. 26A] Subtidal, occurring on brown algae *Sargassumpalmeri* and *Cystoseiraneglecta* (13 m); Catalina Island, California, USA, and Guaymas, Mexico in the Gulf of California (unpublished data). Type locality: USA, California, Catalina Island, Isthmus Reef (33°25.4'N; 118°30.8'W). Body length to ~ 10.6 mm. See Ormsby (1991 original description as *Synisomawetzerae*), Hedo and Junoy (1999), Espinosa-Pérez and Hendrickx (2001a, 2006), Castellanos and Junoy (2005), McLaughlin et al. (2005), Santos et al. (2011), Stebbins (2012a, 2012c), Xavier et al. (2012), and Artüz and Kubanç (2015).

*Synidoteacalcarea* Schultz, 1966 [Fig. 28A] Continental shelf, slope, and submarine canyons (54–1816 m); Tanner and Santa Rosa Canyons, southern California, USA. Type locality: USA, California, Tanner Canyon (R/V Velero IV Station No. 6833-60; 32°37'54"N, 118°58'40"W). Body length to ~ 6 mm. See Schultz (1966 original description, but with prior listing in Schultz 1964 a *nomen nudum*; 1969), Iverson (1972), Menzies and Miller (1972), Lissner et al. (1986), Wetzer et al. (1991), SCAMIT (1994–2021), Poore (1996), Wetzer and Brusca (1997), Moore (2004), McLaughlin et al. (2005), Espinosa-Pérez and Hendrickx (2006), Ranasinghe et al. (2007), Stebbins (2012a, 2012c), and Gillett et al. (2019). Note: See Endnote 22.

*Synidoteaharfordi* Benedict, 1897 [Fig. 28B] Intertidal to shallow subtidal (0–12 m); Oregon, USA to western Baja California Sur, Mexico, the Gulf of California, one record from Costa Rica, and introduced to Japan. Type locality: Mexico, Baja California Sur, Magdalena Bay. Body length to ~ 17 mm. See Benedict (1897), Richardson (1899, 1905a), Schultz (1969), Menzies and Miller (1972), Miller (1975), Brusca and Wallerstein (1979b, 1982), Kussakin (1982), Vargas-Zamora at al. (1985), Poore (1996), Espinosa-Pérez and Hendrickx (2001a, 2006), SCAMIT (2001–2021), Moore (2004), McLaughlin et al. (2005), Brusca et al. (2007), Stebbins (2012a, 2012c), and Hendrickx (2018b).

*Synidoteamagnifica* Menzies & Barnard, 1959 [Fig. 28C] Continental shelf (29–98 m); San Luis Obispo, California, USA to western Baja California Norte, Mexico. Type locality: USA, California, off Oceanside (R/V Velero IV Station No. 5108-57; 33°10'30"N, 117°25'25"W). Body length to ~ 6 mm. See Menzies and Barnard (1959), Schultz (1969), Iverson (1972, 1974), Menzies and Miller (1972), Kussakin (1982), Wetzer et al. (1991), SCAMIT (1994–2021), Poore (1996), Ranasinghe et al. (2003, 2007, 2012), Moore (2004), McLaughlin et al. (2005), Espinosa-Pérez and Hendrickx (2006), Campos and Villarreal (2008), Stebbins (2012a, 2012c), and Gillett et al. (2017, 2022). Note: See Endnote 22.

*Synidoteamedia* Iverson, 1972 [Fig. 28D] Continental shelf (depths to at least 183 m): Strait of Georgia, British Columbia, Canada to Santa Maria Basin, California, USA. Type locality: USA, California, Monterey County, southwest of Point Soberanes (between 36°25'7"N–36°26'23"N). Body length to ~ 8.7 mm. See Iverson (1972), Kussakin (1982), Lissner et al. (1986), SCAMIT (1994–2021), Poore (1996), Wetzer and Brusca (1997), Ranasinghe et al. (2003, 2007), Moore (2004), McLaughlin et al. (2005), Espinosa-Pérez and Hendrickx (2006), Macdonald et al. (2010), Stebbins (2012a, 2012c), and Henkel et al. (2020). Note: See Endnote 22.

#### ﻿Family Thermoarcturidae

*Califarcturustannerensis* (Schultz, 1966) [Fig. 25D] Continental basin and canyon benthos, green mud (1197–1335 m); Tanner Basin and Tanner Canyon, southern California, USA. Type locality: USA, California, Tanner Canyon (R/V Velero IV Station No. 6832-60; 32°33'36"N, 118°55'40"W). Body length to ~ 5.5 mm. See Schultz (1966 original description as *Microarcturustannerensis*; 1969), Wetzer et al. (1991), Stebbins (2012a, 2012c), and Poore (2015: supplementary redescription). Note: The only previously known record for this species was the holotype collected in 1960 from the Tanner Canyon at depths between 1298–1320 m (see Schultz 1966 and Poore 2015). Depth range is herein slightly expanded based on additional specimens collected in 1971 and 1976, which were recently rediscovered in the Crustacea Collection of the Natural History Museum of Los Angeles County (Specimen Nos. 28303 and 28313; TDS, pers. obs.).

**Suborder Asellota**

**Superfamily Janiroidea**

#### ﻿Family Dendrotionidae

*Acanthomunnatannerensis* Schultz, 1966 [Fig. 31A] Continental slope and submarine canyon benthos (570–813 m); southeast of Catalina Island and Tanner Canyon, southern California, USA. Type locality: USA, California, Tanner Canyon (R/V Velero IV Station No. 6833-60; 32°37'54"N, 118°58'40"W). Body length to ~ 3.5 mm. See Schultz (1966, 1969), Wetzer et al. (1991), Cohen (1998), McLaughlin et al. (2005), SCAMIT (2011–2021), Ranasinghe et al. (2012), and Golovan et al. (2018).

#### ﻿Family Desmosomatidae

*Desmosoma* sp. A [Fig. 40A] Continental slope, soft bottom benthos (290 m); Santa Maria Basin off Point Buchon and Point Estero, San Luis Obispo County, California, USA (~ 89–110 km north of SCB northern boundary). Body length to ~ 2.2 mm. See Wilson (1997: 106–107, fig. 1.44, description and figures).

*Momedossasymmetrica* (Schultz, 1966) [Fig. 40B] Continental slope and submarine canyons (469–2955 m); Gulf of the Farallones, San Francisco, northern California to Tanner Canyon, southern California, USA. Type locality: USA, California, Tanner Canyon (R/V Velero IV Station No. 6836-60; 32°36'00"N, 119°05'18"W). Body length to ~ 3.2 mm. See Schultz (1966 original description as *Desmosomasymmetrica*), Lissner et al. (1986), Wetzer et al. (1991), Wilson (1997), Blake et al. (2009), and SCAMIT (2011–2021).

*Prochelator* sp. A [Fig. 40C] Continental shelf, slope, and basins (99–2955 m); Gulf of the Farallones, San Francisco, northern California to Santa Maria Basin off Purisima Point, Santa Barbara County, California, USA (~ 35 km north of SCB northern boundary). Body length to ~ 2.5 mm. See Wilson (1997: 108–109, fig. 1.45, description and figures), Blake et al. (2009), and SCAMIT (2001–2021). Note: Also see Lissner et al. (1986) for previous report of likely same species from the region.

#### ﻿Family Haplomunnidae

*Haplomunnacaeca* (Richardson, 1905) [Fig. 37] Abyssal seafloor (3998 m); ~ 210 km southwest of Santa Catalina Island, southern California, USA. Type locality: USA, southern California (Albatross Station No. 4390; 33°02'15"N, 120°42'W). Body length to ~ 7.7 mm (estimated). See Richardson (1905a original description as *Munnacaeca*; 1908), Menzies (1962b), Wolff (1962), Wilson (1976), Poore (1984c), Cunha and Wilson (2003).

#### ﻿Family Janiridae

*Caecianiropsis* sp. LA1 [Fig. 39B, image of representative species *C.psammophila*] Continental shelf benthos (95 m): off west end of San Miguel Island, southern California, USA. No body size information available, but a similar species from northern California (*C.psammophila* Menzies & Pettit, 1956) measured 1.8 mm in length. See Menzies and Pettit (1956: description of *C.psammophila*), SCAMIT (2001–2021), and Gillett et al. (2017). Note: See Endnote 28.

*Caecianiropsis* sp. LA2 [Fig. 39B, image of representative species *C.psammophila*] Continental shelf benthos, coarse sediments (27 m): offshore Palos Verdes, southern California, USA. No body size information available other than similar in size to *Caecianiropsis* sp. LA1 (see preceding species). See Menzies and Pettit (1956: description of *C.psammophila*), SCAMIT (2001–2021), Ranasinghe et al. (2012), and Gillett et al. (2017, 2022). Note: See Endnote 28.

*Caecijaerahorvathi* Menzies, 1951 [Fig. 39A] Intertidal, in burrows excavated in wood by the isopods *Limnoria* spp.; southern California, USA, and Hawaii. Type locality: USA, California, Los Angeles-Long Beach Harbor, Terminal Island. Body length to ~ 1.7 mm. See Menzies (1951c), Schultz (1969), Cooke (1977), Wetzer et al. (1991), Wilson and Wägele (1994), Espinosa-Pérez and Hendrickx (2006), Brusca et al. (2007), and Ruiz et al. (2011).

*Iaiscalifornica* (Richardson, 1904) [Fig. 31B] Shallow water bays and estuaries, commensal with the wood-boring isopod *Sphaeromaquoianum*; Humboldt Bay, northern California to San Diego, southern California, USA, introduced from Australia or New Zealand, also in Singapore. Type locality: USA, California, Sausalito. Body length to ~ 2.9 mm. See Richardson (1904a original description as *Janiropsiscalifornica*), Menzies and Barnard (1951), Schultz (1969), Rotramel (1972, 1975), Iverson (1974), Miller (1975), Kussakin (1988), Espinosa-Pérez and Hendrickx (2006), Brusca et al. (2007), Espinosa-Pérez et al. (2009), and Ruiz et al. (2011).

*Ianiropsisanaloga* Menzies, 1952 [Fig. 34A] Intertidal, under rocks or amongst *Laminaria* holdfasts; San Juan Archipelago, Washington to Los Angeles Harbor, southern California, USA. Type locality: USA, California, Marin County, Tomales Point (bay side). Body length to ~ 3.7 mm. See Menzies (1952), George and Strömberg (1968), Schultz (1969), Miller (1975), Kussakin (1988), Wetzer et al. (1991), Fairey et al. (2002), Espinosa-Pérez and Hendrickx (2006), Brusca et al. (2007), SCAMIT (2008–2021), Doti and Wilson (2010), Lafferty and Suchanek (2016), and Gillett et al. (2017, 2022).

*Ianiropsisderjugini* Gurjanova, 1933 [Fig. 34B] Intertidal, under rocks covered by algae; Bering Sea to southern California, USA, and Korea (Korea Strait and East Sea). Type locality: Bering Sea. Body length to ~ 4.8 mm. See Gurjanova (1933 original description as *Janiropsisderjugini*), Miller (1975), Kussakin (1988), Jang and Kwon (1990), Wilson (1994), Espinosa-Pérez and Hendrickx (2006), Brusca et al. (2007), Doti and Wilson (2010), and SCAMIT (2011–2021).

*Ianiropsiskincaidi* Richardson, 1904 [Fig. 34C] Intertidal; Bering Sea to Catalina Island and Los Angeles Harbor, southern California, USA. Type locality: USA, Alaska, Yakutat Bay. Body length to ~ 3.8 mm. See Richardson (1904b), George and Strömberg (1968), Schultz (1969), Miller (1975), Kussakin (1988), Jang and Kwon (1990), Wilson (1994), Fairey et al. (2002), Espinosa-Pérez and Hendrickx (2006), Brusca et al. (2007), Espinosa-Pérez et al. (2009), Doti and Wilson (2010), Macdonald et al. (2010), Roletto et al. (2014), Lafferty and Suchanek (2016), and Looby and Ginsburg (2021).

*Ianiropsisminuta* Menzies, 1952 [Fig. 34D] Intertidal, under rocks or sand; Marin County, northern California to southern California, USA. Type locality: USA, California, Marin County, Dillon Beach, Second Sled Road. Body length to ~ 1.3 mm. See Menzies (1952), Schultz (1969), Miller (1975), Lissner et al. (1986), Kussakin (1988), Espinosa-Pérez and Hendrickx (2006), Brusca et al. (2007), Doti and Wilson (2010), and SCAMIT (2011–2021).

*Ianiropsismontereyensis* Menzies, 1952 [Fig. 34E] Intertidal to shallow subtidal, under rocks or in *Macrocystis* holdfasts; Marin County, northern California to southern California, USA. Type locality: USA, California, Monterey County, Pescadero Point. Body length to ~ 3.6 mm. See Menzies (1952), Schultz (1969), Miller (1975), Kussakin (1988), Wetzer et al. (1991), Espinosa-Pérez and Hendrickx (2006), Brusca et al. (2007), Espinosa-Pérez et al. (2009), Doti and Wilson (2010), and SCAMIT (2011–2021).

*Ianiropsistridens* Menzies, 1952 [Fig. 34F] Intertidal, on algae or occasionally in sponges; Strait of Georgia, British Columbia, Canada to Oceanside, southern California, USA, Peru, northern Chile, and Korea (Korea Strait and East Sea). Type locality: USA, California, Marin County, Tomales Point (bay side). Body length to ~ 3 mm. See Menzies (1952, 1962b), George and Strömberg (1968), Schultz (1969), Miller (1975), Kussakin (1988), Jang and Kwon (1990), Wetzer et al. (1991), Fairey et al. (2002), Cohen et al. (2005), Espinosa-Pérez and Hendrickx (2006), Brusca et al. (2007), SCAMIT (2008–2021), Doti and Wilson (2010), Macdonald et al. (2010), Gillett et al. (2017), and Zúñiga Delgado et al. (2021).

*Janiralatadavisi* Menzies, 1951 [Fig. 33A] Low intertidal, under rocks; Monterey County, California, USA to Todos Santos Bay, Baja California Norte, Mexico. Type locality: USA, California, Monterey County, Carmel Cove. Body length to ~ 4.7 mm. See Menzies (1951b), Miller (1975), Kussakin (1988), Wetzer et al. (1991), Espinosa-Pérez and Hendrickx (2006), Brusca et al. (2007), and Campos and Villarreal (2008).

*Janiralataoccidentalis* (Walker, 1898) [Fig. 33B] Intertidal, under rocks; Strait of Georgia, British Columbia, Canada to Orange County, southern California, USA. Type locality: USA, Washington, Puget Sound. Body length to ~ 6 mm. See Walker (1898 original description as *Janiraoccidentalis*), Richardson (1899, 1905a), Stafford (1913b), Hatch (1947), Menzies and Barnard (1959), George and Strömberg (1968), Schultz (1969), Miller (1975), Kussakin (1988), SCAMIT (1994–2021), Bergen et al. (1998), Espinosa-Pérez and Hendrickx (2006), Brusca et al. (2007), Ranasinghe et al. (2007), and Macdonald et al. (2010).

*Janiralatarajata* Menzies, 1951 [Fig. 33C] Subtidal (1–36 m); Monterey Bay, California to Anacapa and Catalina Islands, southern California, USA. Type locality: USA, California, Monterey Bay (from eggcase of the skate *Rajabinoculata*). Body length to ~ 4 mm. See Menzies (1951b), Miller (1968), Schultz (1969), Miller (1975), Kussakin (1988), and Espinosa-Pérez and Hendrickx (2006). Note: See Endnote 24.

*Janiralatasolasteri* (Hatch, 1947) [Fig. 33D] Subtidal (50–218 m): Dall Island, Gulf of Alaska to southern California, USA. Type locality: USA, Washington, Hood Canal, on the sea star *Solasterstimpsoni*. Body length to ~ 5 mm. See Hatch (1947 original description as *Janirasolasteri*), Menzies and Barnard (1959), Schultz (1966, 1969), George and Strömberg (1968), Miller (1975), Lissner et al. (1986), Kussakin (1988), SCAMIT (1994–2021), Bergen et al. (1998), Espinosa-Pérez and Hendrickx (2006), Ranasinghe et al. (2007), Espinosa-Pérez et al. (2009), Macdonald et al. (2010), and Puget Sound Institute (2012–2023).

*Janiralata* sp. A [Fig. 33E] Continental shelf (160–168 m); Santa Maria Basin, southern California, USA. Body length ~ 4.4 mm. See Wilson (1997: 80–82, fig. 1.31, description and figures), SCAMIT (2011–2021), and Ranasinghe et al. (2012).

*Janiralata* sp. B [Fig. 33F] Continental shelf and upper slope (168–237 m); Santa Maria Basin, southern California, USA. Body length ~ 4.8 mm. See Wilson (1997: 82–84, fig. 1.32, description and figures), Ranasinghe et al. (2007), and SCAMIT (2008–2021).

*Janiralata* sp. C [Fig. 33G] Continental shelf, hard bottom benthos (91–123 m); Santa Maria Basin offshore of Purisima Point, Santa Barbara County, California, USA (~ 35 km north of SCB northern boundary). Body length ~ 2.4 mm. See Wilson (1997: 84–85, fig. 1.33, description and figures).

*Janiralata* sp. D [Fig. 33H] Continental shelf, hard bottom benthos (91–123 m); Santa Maria Basin offshore of Purisima Point, Santa Barbara County, California, USA (~ 35 km north of SCB northern boundary). Body length ~ 3.5 mm. See Wilson (1997: 86–87, fig. 1.34, description and figures) and SCAMIT (2008–2021). Note: See Endnote 24.

#### ﻿Family Joeropsididae

*Joeropsisconcava* Schultz, 1966 [Fig. 32A] Continental shelf and submarine canyons, coarse sediments (60–221 m); Central California to San Diego, southern California, USA. Type locality: USA, California, Santa Cruz Canyon (R/V Velero IV Station No. 6806-59; 33°56'06"N, 118°52'17"W). Body length to ~ 4.2 mm. See Schultz (1966, 1969), Wetzer et al. (1991), Wilson (1997), SCAMIT (1994–2021), Ranasinghe et al. (2003, 2007, 2012), Espinosa-Pérez and Hendrickx (2006), Espinosa-Pérez et al. (2009), and Gillett et al. (2017, 2022).

*Joeropsisdubiadubia* Menzies, 1951 [Fig. 32B] Low intertidal to offshore shelf and submarine canyons, often occurring under rocks, in algal holdfasts, and on bryozoans, tunicates, hydroids and barnacles (0–116 m); Strait of Georgia, British Columbia, Canada to San Quintin Bay, Baja California Norte, the Gulf of California, and western Mexico (i.e., Bahia de Banderas, Nayarit). Type locality: USA, California, Marin County, Dillon Beach, First Sled Road north of Pacific Marine Station. Body length to ~ 2.8 mm. See Menzies (1951b original description as *Jaeropsisdubia*; 1962a), Menzies and Barnard (1959), Schultz (1966, 1969), Miller (1968, 1975), Carvacho (1983), Lissner et al. (1986), Wetzer et al. (1991), Thompson et al. (1993), SCAMIT (1994–2021 as *J.dubia*), Kussakin (1999), Espinosa-Pérez and Hendrickx (2001a, 2006), Smith et al. (2001, 2003), Ranasinghe et al. (2003, 2007, 2012), Brusca et al. (2007), Campos and Villarreal (2008), Macdonald et al. (2010), Puget Sound Institute (2012–2023), Cruz-García et al. (2013), Lafferty and Suchanek (2016), and Gillett et al. (2017, 2019, 2022). Note: If confirmed as *J.dubiadubia*, the Gillett et al. (2019) record extends the maximum depth range of this species from 116 m to the lower slope between 500–1000 m.

*Joeropsisdubiapaucispinis* Menzies, 1951 [Fig. 32C] Intertidal to offshore shelf and submarine canyons (0–116 m); Marin County, northern California to Santa Monica Canyon, southern California, USA. Type locality: USA, California, Marin County, Dillon Beach. Body length < 3 mm. See Menzies (1951b original description as *Jaeropsisdubiapaucispinis*; 1962a), Menzies and Barnard (1959), Schultz (1966, 1969), Miller (1968, 1975), Kussakin (1999), Espinosa-Pérez and Hendrickx (2006), Brusca et al. (2007), and Espinosa-Pérez et al. (2009).

*Joeropsislobata* Richardson, 1899 [Fig. 32D] Intertidal to shallow water; Coos Bay, Oregon to southern California, USA, plus one recent record from Namhyeongjeseom Islet, Busan, Republic of Korea (see Hwang et al. 2014) that should be further studied to verify. Type locality: USA, California, Monterey Bay. Body length to ~ 3.5 mm. See Richardson (1899, 1900c, 1905a), Shearer (1942), Hatch (1947), Schultz (1969), Miller (1975), Bergen et al. (1998), Kussakin (1999), Espinosa-Pérez and Hendrickx (2006), Brusca et al. (2007), SCAMIT (2008–2021), and Espinosa-Pérez et al. (2009).

*Joeropsis* sp. A [Fig. 32E] Continental shelf, hard bottom benthos (54–131 m); Santa Maria Basin offshore of Point Arguello and Purisima Point, Santa Barbara County, California, USA (~ 30–35 km north of SCB northern boundary). Body length ~ 2.8 mm. See Wilson (1997: 90–91, fig. 1.36, description and figures).

#### ﻿Family Lepidocharontidae

*Microcharon* sp. A [Fig. 39C, image of representative species *M.sabulum*] Continental shelf benthos (75 m); Channel Islands, southern California, USA. No body size information available, but a similar species from the Caribbean listed in Kensley and Schotte (1989), *M.sabulum*, measured ~ 1.5 mm in length. See Kensley and Schotte (1989), Ranasinghe et al. (2012), and SCAMIT (2012–2021). Note: See Endnote 27.

#### ﻿Family Munnidae

*Munnachromatocephala* Menzies, 1952 [Fig. 36A] Intertidal, on red algae and encrusting organisms on rocks; Strait of Georgia, British Columbia, Canada to central and southern California, USA. Type locality: USA, California, Marin County, Dillon Beach, First Sled Road. Body length to ~ 2.2 mm. See Menzies (1952), George and Strömberg (1968), Schultz (1969), Miller (1975), Poore (1984c), Kussakin (1988), Wetzer et al. (1991), McLaughlin et al. (2005), Espinosa-Pérez and Hendrickx (2006), Brusca et al. (2007), SCAMIT (2008–2021), Espinosa-Pérez et al. (2009), Macdonald et al. (2010), and Lafferty and Suchanek (2016).

*Munnafernaldi* George & Strömberg, 1968 [Fig. 36B] Intertidal, from stones and algae; San Juan Archipelago, Washington to southern California, USA. Type locality: USA, Washington, San Juan Archipelago, San Juan Island, Friday Harbor. Body length to ~ 1.5 mm. See George and Strömberg (1968), Schultz (1969), Poore (1984c), Kussakin (1988), McLaughlin et al. (2005), Espinosa-Pérez and Hendrickx (2006), Puget Sound Institute (2012–2023), and SCAMIT (2012–2021).

*Munnahalei* Menzies, 1952 [Fig. 36C] Intertidal to shallow subtidal, under rocks, in *Macrocystis* holdfasts, and among spines of the purple sea urchin *Strongylocentrotuspurpuratus*; Tomales Bay, northern California to southern California, USA. Type locality: USA, California, Marin County, Tomales Point, Tomales Bluff. Body length to ~ 1.5 mm. See Menzies (1952), Schultz (1969), Iverson (1974), Miller (1975), Harty (1979), Poore (1984c), Kussakin (1988), McLaughlin et al. (2005), Espinosa-Pérez and Hendrickx (2006), Brusca et al. (2007), and SCAMIT (2011–2021).

*Munnamagnifica* Schultz, 1964 [Fig. 36D] Offshore continental slope, black mud, nodules, and flat shaley rocks (500 m); southern California, USA. Known only from the type material. Type locality: USA, California, 13.1 miles WNW of Santa Barbara Island (R/V Velero IV Station No. 2969-54; 33°35'59"N, 119°15'11"W). Body length to ~ 2 mm. See Schultz (1964, 1969), Wilson (1980), Poore (1984c), Wetzer et al. (1991), and McLaughlin et al. (2005).

*Munnaspinifrons* Menzies & Barnard, 1959 [Fig. 36E] Offshore continental shelf; 12–218 m: Point Conception to Point Loma, San Diego, southern California, USA. Type locality: USA, California, 11 miles east of Point Conception (R/V Velero IV Station No. 4822-57; 34°27'15"N, 120°14'45"W). Body length to ~ 1.5 mm. See Menzies and Barnard (1959), Schultz (1966, 1969), Poore (1984c), Kussakin (1988), Wetzer et al. (1991), SCAMIT (1998–2021), McLaughlin et al. (2005), Espinosa-Pérez and Hendrickx (2006), and Espinosa-Pérez et al. (2009).

*Munnastephenseni* Gurjanova, 1933 [Fig. 36F] Intertidal to shallow subtidal (0–18 m); Bering Sea to central and southern California, USA, and Japan. Type locality: Bering Sea, Komador Islands. Body length to ~ 3 mm. See Gurjanova (1933), Schultz (1969), Miller (1975), Poore (1984c), Kussakin (1988), SCAMIT (1994–2021), Shimomura and Mawatari (2001), McLaughlin et al. (2005), Espinosa-Pérez and Hendrickx (2006), and Brusca et al. (2007). Note: See Shimomura and Mawatari (2001) for a brief discussion of differences between California and Japanese specimens of *M.stephenseni*.

*Munna* sp. A [Fig. 36G] Continental shelf and upper slope, hard substrates (105–237 m); Santa Maria Basin, southern California, USA. Body length to ~ 1.5 mm. See Wilson (1997: 71–72, fig. 1.27, description and figures), Espinosa-Pérez and Hendrickx (2006), Ranasinghe et al. (2007), and SCAMIT (2008–2021). Note: See Lissner et al. (1986) for previous report of likely same species from the region.

*Uromunnaubiquita* (Menzies, 1952) [Fig. 36H] Intertidal to shallow subtidal; Strait of Georgia, British Columbia, Canada to San Quintin Bay, Baja California Norte, and the Gulf of California, Mexico. Type locality: USA, California, Marin County, Tomales Bay. Body length to ~ 1.2 mm. See Menzies (1952 original description as *Munnaubiquita*; 1962a), Menzies and Barnard (1959), George and Strömberg (1968), Schultz (1969), Miller (1975), Carvacho (1983), Poore (1984c), Kussakin (1988), Wetzer et al. (1991), SCAMIT (1994–2021), Chess and Hobson (1997), Espinosa-Pérez and Hendrickx (2001a, 2006), Ranasinghe et al. (2003, 2012), McLaughlin et al. (2005), Espinosa-Pérez and Hendrickx (2006), Brusca et al. (2007), Macdonald et al. (2010), Puget Sound Institute (2012–2023), Esquete and Wilson (2016), and Gillett et al. (2017, 2022).

#### ﻿Family Munnopsidae

*Belonectes* sp. A [Fig. 38A] Continental slope and submarine canyons (305–401 m); Santa Maria Basin to La Jolla Canyon, southern California, USA. Body length to ~ 2.1 mm. See SCAMIT (1994–2021), Wilson (1997: 94–95, fig. 1.38, description and figures), and Gillett et al. (2017, 2019). Note: If confirmed, the Gillett et al. (2019) record extends the maximum depth range of this species slightly from 401 m to the lower slope between 500 and 1000 m.

*Eurycopecaliforniensis* Schultz, 1966 [Fig. 38B] Continental slope (478–930 m); Santa Maria Basin to Newport Canyon, southern California, USA. Type locality: USA, California, Newport Canyon (R/V Velero IV Station No. 7032-60; 33°31'28"N, 117°54'58"W). Body length to ~ 3.5 mm. See Schultz (1966, 1969), Gillard (1978), Lissner et al. (1986), Wetzer et al. (1991), Wilson (1997), McLaughlin et al. (2005), Ranasinghe et al. (2007, 2012), SCAMIT (2008–2021), and Gillett et al. (2017, 2019, 2022).

*Ilyarachnaacarina* Menzies & Barnard, 1959 [Fig. 38C] Continental shelf, slope, and submarine canyons (73–1118 m, and possibly > 2100 m per ‘Note’ below); San Francisco, northern California to San Diego, southern California, USA (including Santa Maria Basin, and Coronado, Redondo, San Pedro, Santa Cruz, and Santa Monica canyons). Type locality: USA, California, off Santa Barbara (R/V Velero IV Station No. 4980-57; 34°15'50"N, 119°34'28"W). Body length to ~ 4.3 mm. See Menzies and Barnard (1959), Schultz (1964, 1966, 1969), Hessler and Thistle (1975), Gillard (1978), Thistle (1979), Lissner et al. (1986), Wetzer et al. (1991), SCAMIT (1994–2021), Wilson 1997), Bergen et al. (1998), Smith et al. (2001, 2003), Kussakin (2003), McLaughlin et al. (2005), Espinosa-Pérez and Hendrickx (2006), Ranasinghe et al. (2007, 2012), Blake et al. (2009), Espinosa-Pérez et al. (2009), and Gillett et al. (2017, 2019, 2022). Note: San Francisco specimens identified as Ilyarachnacf.acarina were collected from a subset of lower slope stations ranging between 2160–3140 m in depth (see Blake et al. 2009).

*Ilyarachnaprofunda* Schultz, 1966 [Fig. 38D] Continental slope, basins, and submarine canyons (461–1670 m, and possibly > 2100 m per ‘Note’ below), extremely abundant on whale falls (skeletons); San Francisco, northern California to San Diego, southern California, USA (including Santa Cruz Basin, La Jolla, San Pedro, Santa Catalina and Tanner canyons). Type locality: USA, California, La Jolla Canyon (R/V Velero IV Station No. 7047-60; 32°54'21"N, 117°29'53"W). Body length to ~ 3.8 mm. See Schultz (1966, 1969), Hessler and Thistle (1975), Thistle (1979), Wetzer et al. (1991), Wilson (1997), Smith and Baco (2003), McLaughlin et al. (2005), Ranasinghe et al. (2007, 2012), SCAMIT (2008–2021), Blake et al. (2009), Bernardino et al. (2010), and Gillett et al. (2019, 2022). Note: San Francisco specimens identified as Ilyarachnacf.profunda were collected from a subset of lower slope stations ranging between 2160–3140 m in depth (see Blake et al. 2009).

*Munnopsurus* sp. A [Fig. 38E] Continental slope and submarine canyons (393–582 m); Santa Maria Basin to La Jolla Canyon, southern California, USA. Body length to ~ 1.8 mm. See Wilson (1997: 98–99, fig. 1.40, description and figures), SCAMIT (1998–2021), Ranasinghe et al. (2012), and Gillett et al. (2019). Note: See Lissner et al. (1986) for previous report of likely same species from the region.

*Munnopsurus* sp. B [Fig. 38F] Continental slope, soft bottom benthos (930 m); Santa Maria Basin west of Point Conception, Santa Barbara County, California, USA. Body length to ~ 7 mm. See Wilson (1997: 100–101, fig. 1.41, description and figures).

#### ﻿Family Nannoniscidae

*Nannonisconuslatipleonus* Schultz, 1966 [Fig. 40D] Continental slope and submarine canyons (294–465 m); Western Santa Barbara Channel off Point Conception to Redondo Canyon, southern California, USA. Type locality: USA, California, Redondo Canyon (R/V Velero IV Station No. 2793-54; 33°48'00"N, 118°32'00"W). Body length to ~ 2.8 mm. See Schultz (1966, 1969), Siebenaller and Hessler (1981), Lissner et al. (1986), Wetzer et al. (1991), Wilson (1997, 2008b), and McLaughlin et al. (2005). Note: Wilson (1997) briefly discusses that *N.latipleonus* may be conspecific with the more northern *N.carinatus* Mezhov, 1986. If true, this will extend the geographic range of *N.latipleonus* to Alaska Bay in the northeast Pacific.

#### ﻿Family Paramunnidae

*Austrosignumlatum* Just & Wilson, 2021 [Fig. 35A] Subtidal benthos (20 m); La Jolla Canyon, southern California, USA. Type locality: USA, California, La Jolla (32°52.09'N, 117°15.69'W). Body length to ~ 2 mm. See Just and Wilson (2021) for original description and comparison with other species of the genus.

*Boreosignum* sp. A [Fig. 35B] Shallow subtidal rocks (9–16 m); Point La Jolla to Pin Rock, Catalina Island, southern California, USA. Body length < 1 mm. See Cadien (2008: voucher sheet for *Boreosignum* sp. IS1 available from SCAMIT Toolbox at https://www.scamit.org; specimens examined 5 April 2008, D. B. Cadien), Maloney et al. (2008), and SCAMIT (2012–2021).

*Munnogoniumerratum* (Schultz, 1964) [Fig. 35C] Continental shelf (98–158 m); Morro Bay, central California to Santa Maria Basin, southern California, USA. Type locality: USA, California, Santa Barbara County, 4.6 miles 137 degrees from Gaviota Pier (R/V Velero IV Station No. 6003-58; 34°24'45"N, 120°08'40"W). Body length to ~ 1.8 mm. See Schultz (1964 original description as *Austrosignumerratum*; 1969), Bowman and Schultz (1974), Lissner et al. (1986), Wetzer et al. (1991), SCAMIT (1994–1996), Wilson (1997, as Munnogoniumcf.tillerae), McLaughlin et al. (2005), Espinosa-Pérez and Hendrickx (2006), Just and Wilson (2021: comparison to redescribed *M.tillerae*), and Golovan and Malyutina (2022).

*Munnogoniumtillerae* (Menzies & Barnard, 1959) [Fig. 35D] Continental shelf (101–127 m); San Diego, southern California, USA. Type locality: USA, California, off Point Loma (R/V Velero IV Station No. 4753-56; 32°41'50"N, 117°20'25"W). Body length to ~ 1.3 mm. See Menzies and Barnard (1959 original description as *Austrosignumtillerae*), Schultz (1969), Bowman and Schultz (1974), Lissner et al. (1986), Kussakin (1988), Wetzer et al. (1991), SCAMIT (1994–2021), Wilson (1997: but not M.cf.tillerae [see *M.erratum* above]), Bergen et al. (1998), Smith et al. (2001, 2003), Ranasinghe et al. (2003, 2007, 2012), McLaughlin et al. (2005), Espinosa-Pérez and Hendrickx (2006), Brusca et al. (2007), Gillett et al. (2017, 2019, 2022), Just and Wilson (2021: redescription, range restriction, and comparison to *M.erratum*), and Golovan and Malyutina (2022).

*Paramunnaquadratifrons* Iverson & Wilson, 1981 [Fig. 41A] Continental shelf (197 m); southern Channel Islands and Tanner bank, southern California, USA. Type locality: USA, California, Tanner Bank, ~ 160 kilometers southwest of Los Angeles (33°53.24'N; 119°23.35'W). Body length to ~ 1.3 mm. See Iverson and Wilson (1981), Wetzer et al. (1991), McLaughlin et al. (2005), Espinosa-Pérez and Hendrickx (2006), SCAMIT (2011–2021), Ranasinghe et al. (2012), and Golovan and Malyutina (2022).

*Paramunna* sp. A [Fig. 41B] Continental shelf (100 m); off Del Mar, San Diego County, southern California, USA. Body length to ~ 1 mm. See Cadien (1996: voucher sheet available [in part] in SCAMIT Newsletter Vol. 15, No. 2 at https://www.scamit.org; specimens examined 30 May 1996, D. Cadien) and SCAMIT (2013–2021).

*Paramunna* sp. SD1 [No figure] Channel Island shelf (75 m); NE Anacapa Island, southern California, USA. No body size information available. See Ranasinghe et al. (2007) and SCAMIT (2008–2021). Note: Provisional species designation based on diagnostic notes for “Asellota sp. SD1” drafted by D. Pasko in 2004 (see Endnote 29).

*Pleurogoniumcaliforniense* Menzies, 1951 [Fig. 41C] Continental shelf, slope and submarine canyons, benthic sediments (93–150 m, and possibly > 2100 m as per ‘Note’ below); Strait of Georgia, British Columbia, Canada to Point Loma, San Diego, southern California, USA. Type locality: USA, California, Sonoma County, 3 miles west of mouth of Russian River. Body length to ~ 1.3 mm. See Menzies (1951b), Menzies and Barnard (1959), Schultz (1966, 1969), Lissner et al. (1986), Kussakin (1988), SCAMIT (1994–2021), Bergen et al. (1998), Smith et al. (2001, 2003), McLaughlin et al. (2005), Espinosa-Pérez and Hendrickx (2006), Ranasinghe et al. (2007, 2012), Blake et al. (2009), Macdonald et al. (2010), Gillett et al. (2017, 2019, 2022), and Golovan and Malyutina (2022). Note: If confirmed, the Gillett et al. (2019) record extends the maximum depth range of this species slightly from 150 m to the upper slope between 200–500 m. Additionally, San Francisco specimens identified as Pleurogoniumcf.californiense were collected from a subset of lower slope stations ranging between 2160–3140 m in depth as reported by Blake et al. (2009), thus extending this bathymetric range into even deeper waters.

*Pleurogonium* sp. A [Fig. 41D] Continental shelf benthos (90–154 m); Santa Maria Basin to San Diego, southern California, USA. Body length to ~ 1.6 mm. See Wilson (1997: 78–79, fig. 1.30, description and figures), Bergen et al. (1998), SCAMIT (1994–2021), Ranasinghe et al. (2007, 2012), and Gillett et al. (2019, 2022). Note: If confirmed, the Gillett et al. (2019) record extends the maximum depth range of this species slightly from 154 m to the upper slope between 200–500 m.

#### ﻿Family Pleurocopidae

*Pleurocope* sp. A [Fig. 35E] Shallow water fouling communities (0.5 m); Central California to Avalon Harbor, Catalina Island and Point Loma, San Diego, southern California, USA. Body length to ~ 1.2 mm. See Cadien (2012: voucher sheet for original provisional species designation *Pleurocope* sp. IS1 available from TD Stebbins; specimens examined 4 March 2012, DB Cadien), SCAMIT (2012–2021), and prior SCB reports of *P.floridensis* in Maloney et al. (2006, 2008). Note: See Endnote 25.

**Superfamily Stenetrioidea**

#### ﻿Family Stenetriidae

*Stenetrium* sp. A [Fig. 31C] Continental shelf, hard bottom benthos (90–131 m); Santa Maria Basin offshore of Purisima Point, Santa Barbara County, California, USA (~ 35 km north of SCB northern boundary). Body length to ~ 7.3 mm. See Wilson (1997: 69–70, fig. 1.26, description and figures). Note: See Lissner et al. (1986) for previous report of likely same species from the region.

**Suborder Oniscidea**

**Superfamily Oniscoidea**

#### ﻿Family Alloniscidae

*Alloniscusmirabilis* (Stuxberg, 1875) [Fig. 43A] Littoral halophilic species common on sandy beaches, burrows into sand under driftwood above high tide line (may co-occur with *A.perconvexus*); San Mateo County, northern California, USA to Magdalena Bay, Baja California Sur, Mexico. Type locality: USA, California. Body length to ~ 9.4 mm. See Stuxberg (1875 original description as *Rhinoryctesmirabilis*), Richardson (1899, 1900c, 1905a), Stafford (1913a as A.cornutusvar.lagunae), Schultz (1984), Garthwaite and Lawson (1992), Leistikow and Wägele (1999), Jass and Klausmeier (2000, 2001), Schmalfuss (2003), McLaughlin et al. (2005), Espinosa-Pérez and Hendrickx (2006), Brusca et al. (2007), and Campos-Filho et al. (2018).

*Alloniscusperconvexus* Dana, 1854 [Fig. 43B] Littoral halophilic species common on sandy beaches, burrows into sand under driftwood above most recent high tide line (may co-occur with *A.mirabilis* or *Tylospunctatus* in southern California); British Columbia, Canada to Magdalena Bay, Baja California Sur, Mexico. Type locality: USA, California. Body length to ~ 12 mm. See Dana (1854), Richardson (1899, 1900c, 1905a), Shearer (1942), Hewatt (1946), Hatch (1947), Brusca (1966), George and Strömberg (1968), Miller (1975), Lee and Miller (1980), Schultz (1984), Ricketts et al. (1985), Garthwaite and Lawson (1992), Leistikow and Wägele (1999), Jass and Klausmeier (2000, 2001), Espinosa-Pérez and Hendrickx (2001a, 2006), Schmalfuss (2003), McLaughlin et al. (2005), Brusca et al. (2007), SCAMIT (2011–2021), Hubbard et al. (2014), Campos-Filho et al. (2018), and Wright and Harris (2020).

#### ﻿Family Detonidae

*Armadilloniscuscoronacapitalis* Menzies, 1950 [Fig. 43C] Littoral halophilic species, occurring under stones, driftwood, decaying eelgrass, or other debris along high tide line of marshes, bays, and estuaries; Marin County, northern California to San Miguel and Anacapa Islands, southern California, USA. Type locality: USA, California, Marin County, Tomales Bay, Tomales Point. Body length to ~ 4.6 mm. See Menzies (1950c), Miller (1975), Taiti and Ferrara (1989), Garthwaite and Lawson (1992), Garthwaite et al. (1992), Leistikow and Wägele (1999), Jass and Klausmeier (2000, 2001), Schmalfuss (2003), McLaughlin et al. (2005), and Espinosa-Pérez and Hendrickx (2006).

*Armadilloniscusholmesi* Arcangeli, 1933 [Fig. 43D] Littoral halophilic species, occurring under stones, driftwood, decaying eelgrass, or other debris along high tide line of marshes, bays and estuaries, and in the spray zone on rocky beaches: British Columbia, Canada to Magdalena Bay, Baja California Sur, Mexico. Type locality: USA, California, San Diego County, San Diego. Body length to ~ 3.9 mm. See Arcangeli (1933), Menzies (1950c), Miller (1975), Taiti and Ferrara (1989), Garthwaite and Lawson (1992), Garthwaite et al. (1992), Leistikow and Wägele (1999), Jass and Klausmeier (2000, 2001), Schmalfuss (2003), McLaughlin et al. (2005), Espinosa-Pérez and Hendrickx (2006), Wright and Harris (2020), and SCAMIT (2021).

*Armadilloniscuslindahli* (Richardson, 1905) [Fig. 43E] Littoral halophilic species, occurring under stones, driftwood, decaying eelgrass, or other debris along high tide line of marshes, bays and estuaries, and in the spray zone on rocky beaches; Tomales Bay, northern California to San Diego, southern California, USA. Type locality: USA, California, Oakland. Body length to ~ 4.5 mm. See Richardson (1905a original description as *Actoniscuslindahli*), Menzies (1950c), Miller (1975), Taiti and Ferrara (1989), Garthwaite and Lawson (1992), Garthwaite et al. (1992), Leistikow and Wägele (1999), Jass and Klausmeier (2000, 2001), Schmalfuss (2003), McLaughlin et al. (2005), Espinosa-Pérez and Hendrickx (2006), and Wright and Harris (2020).

#### ﻿Family Halophilosciidae

*Littorophilosciarichardsonae* (Holmes & Gay, 1909) [Fig. 42B] Littoral halophilic species common above the high tide line in marshes, bays and estuaries, and in the spray zone on rocky beaches; Vancouver Island, British Columbia, Canada to Cedros Island, Baja California Norte. Mexico. Type locality: USA, California, San Diego, on moist swampy ground. Body length to ~ 5 mm. See Holmes and Gay (1909 original description as *Philosciarichardsonae*), Hatch (1947), George and Strömberg (1968), Miller (1975), Taiti and Ferrara (1986), Garthwaite and Lawson (1992), Leistikow and Wägele (1999), Jass and Klausmeier (2000, 2001), Schmalfuss (2003), McLaughlin et al. (2005), Espinosa-Pérez and Hendrickx (2006), Brusca et al. (2007), Whitcraft et al. (2008), Roletto et al. (2014), Shaughnessy et al. (2017), and Wright and Harris (2020).

#### ﻿Family Ligiidae

Ligiacf.occidentalis (Dana, 1853) [Fig. 42A, image of representative species *L.occidentalis*] Spray zone of high intertidal rocky shores; Refugio (south of Point Conception), southern California, USA to Puerto San Carlos, Baja California Sur, Mexico. Type locality: not applicable, but near the Sacramento River, California, USA for *L.occidentalis* [overall range: Oregon, USA to Chamela Bay, Jalisco, Mexico, and Gulf of California]. Body length to ~ 25 mm (without uropods). See Dana (1853 original description as *Lygiaoccidentalis*), Richardson (1899, 1900c, 1905a), Hewatt (1946), Brusca (1966), Miller (1975), Allen (1976), Lee and Miller (1980), Ricketts et al. (1985), Garthwaite and Lawson (1992), Leistikow and Wägele (1999), Jass and Klausmeier (2000, 2001), Espinosa-Pérez and Hendrickx (2001a, 2002, 2006), Schmalfuss (2003), Brusca et al. (2004, 2007), McLaughlin et al. (2005), Campos and Villarreal (2008), Hurtado et al. (2010), Eberl (2012), SCAMIT (2012–2021), Eberl et al. (2013), Roletto et al. (2014), Santamaria et al. (2016), and Wright and Harris (2020). Note: References cited above cover *L.occidentalis* in general (including L.cf.occidentalis as recognized herein); see Endnote 30.

#### ﻿Family Platyarthridae

*Niambiacapensis* (Dollfus, 1895) [Fig. 42C] Supralittoral and riparian species, occurring on sandy beaches and the edges of marshes in beach wrack, holdfasts, and under driftwood, logs, and rocks; introduced species from Namibia and South Africa, occurring along Pacific coast of North America from southern Washington State to southern California, USA, and in continental Chile and on Saint Helena Island in the South Atlantic Ocean. Type locality: Africa, Namibia. Body length to ~ 5.4 mm. See Dollfus (1895 original description as *Metoponorthuscapensis*), Miller (1936 as *Porcelliolittorinus*), Leistikow and Wägele (1999), Jass and Klausmeier (2000, 2001), Schmalfuss (2003), McLaughlin et al. (2005), Espinosa-Pérez and Hendrickx (2006), Brusca et al. (2007), Maloney et al. (2008), Pérez-Schultheiss et al. (2018), and Pérez-Schultheiss and Urra (2020).

#### ﻿Family Tylidae

*Tylospunctatus* Holmes & Gay, 1909 [Fig. 42D] Littoral halophilic species on sandy beaches, burrows in sand above the most recent high tide line during the day and is active on the surface at night (may co-occur with *Alloniscusperconvexus* in southern California); San Diego, California, USA to Ensenada, Baja California Norte, Mexico, and the Gulf of California. Type locality: USA, California, San Diego, in sand near the beach. Body length to ~ 10 mm. See Holmes and Gay (1909), Stafford (1913b), Hewatt (1946), Schultz (1970), Brusca (1980), Ricketts et al. (1985), Leistikow and Wägele (1999), Jass and Klausmeier (2000, 2001), Espinosa-Pérez and Hendrickx (2001a, 2006), Schmalfuss (2003), Brusca et al. (2004, 2007), McLaughlin et al. (2005), SCAMIT (2011–2021), Hubbard et al. (2014), and Wright and Harris (2020).

## ﻿Discussion

The marine habitats of the Southern California Bight (SCB) are characterized by a diverse isopod fauna with species occurring from the upper intertidal of rocky shores and sandy beaches, to the shallow waters of local bays and estuaries, and in the deeper offshore benthos of the continental shelf, slope, basins, and submarine canyons (Table 2). The offshore shelf, composed of soft and hard bottom substrates at depths to ~ 200 m, houses the greatest diversity of SCB isopods (119 species), while the various intertidal habitats have the second highest diversity (89 species). Fewer species have been reported from the shallow subtidal waters of SCB bays and estuaries (13 species) as well as deeper waters of the continental slope to ~ 1000 m (38 species) and below (15 species). However, this pattern may be due in part to less intensive sampling that has been conducted in the deeper areas compared to the shelf and intertidal habitats.

**Table 2.** Diversity of isopods by habitat type in the Southern California Bight. Data are expressed as the number of species per taxon occurring in each habitat. Intertidal habitats include outer coast rocky shores and sandy beaches, as well as soft and hard substrates along the edges of marshes, bays, and estuaries. Subtidal habitats include shallow bays and estuaries, plus shallow to deep waters of the continental shelf, slope, basins, and submarine canyons.

Taxon	Intertidal	Bays & estuaries	Shelf, slope, basins, canyons		
			< 200 m	200–1000 m	> 1000 m
**Suborder Cymothoida**	**20**	**7**	**47**	**19**	**8**
Superfamily Anthuroidea	4	2	10	2	1
Superfamily Cymothooidea	8	1	24	14	5
Infraorder Epicaridea					
Superfamily Bopyroidea	7	4	11	1	1
Superfamily Cryptoniscoidea	1	–	2	2	1
**Suborder Limnorioidea**	**3**	–	**1**	–	–
**Suborder Sphaeromatidea**	**22**	**4**	**16**	–	–
Superfamily Seroloidea	1	1	1	–	–
Superfamily Sphaeromatoidea	21	3	15	–	–
**Suborder Valvifera**	**18**	**1**	**22**	**1**	**2**
**Suborder Asellota**	**17**	**1**	**33**	**18**	**5**
Superfamily Janiroidea	17	1	32	18	5
Superfamily Stenetrioidea	–	–	1	–	–
**Suborder Oniscoidea**	**9**	–	–	–	–
**Total No. Species**	**89**	**13**	**119**	**38**	**15**

Overall, 190 species of isopods are recognized in this review as likely to occur in the SCB. However, seven of these species represent provisional taxa confirmed from just a few sites located ~ 30–90 km north of the SCB northern boundary (i.e., between Point Conception and San Luis Obispo) that were sampled during the benthic survey of the Santa Maria Basin and Western Santa Barbara Channel in the 1980s (see Wetzer and Brusca 1997; Wetzer et al. 1997; Wilson 1997). These species include the anthuroid *Kupellonura* sp. A, and the asellotes *Desmosoma* sp. A, *Janiralata* sp. C, *Janiralata* sp. D, *Joeropsis* sp. A, *Prochelator* sp. A, and *Stenetrium* sp. A that occurred at depths between ~ 50–300 m. Of these species, *Prochelator* sp. A has also been reported from much deeper waters (2955 m) located further north in the Gulf of the Farallones off San Francisco, northern California (Blake et al. 2009). It seems reasonable to expect that each of the above seven species may also range at least a little further south into northern SCB waters and therefore be encountered in future surveys of the region.

Approximately 22% of the isopods treated herein (41 species) appear restricted to SCB waters at this time. However, fewer than half of these isopods have been formally described, while the remainder represent distinct provisional species of which many are known from only one or a few samples. Thus, it seems likely that some or all these provisional species may have broader geographic distributions than summarized in this review. In contrast, most of the species covered in this paper (~ 76%) have broader eastern Pacific distributions. For example, 111 of the species covered (~ 58%) also occur north of the SCB ranging variously along the coasts of central and northern California to Oregon, Washington, British Columbia, Alaska, and into the Bering Sea. Additionally, 73 species (~ 38%) also range south of the SCB in the eastern Pacific, occurring along the southwestern coast of Baja California, throughout the Gulf of California, and off the western coasts of Mexico, Central and South America, and the Galapagos Islands.

Twenty-seven of the SCB isopod species also occur in other areas of the world beyond the eastern Pacific, some of which have been introduced to new regions or distributed globally by international shipping. These wide-ranging species include one paranthurid (*Paranthurajaponica*), two aegids (*Aegiochusplebeia* and *Rocinelasignata*), one cirolanid (*Excirolanachiltoni*), two cymothoids (*Ceratothoagaudichaudii* and *Renocilathresherorum*), six epicarideans (*Aporobopyrusoviformis*, *Argeiapugettensis*, *Bathygygegrandis*, *Orthionegriffenis*, *Ionecornuta*, and *Hemioniscusbalani*), two limnoriids (*Limnoriaquadripunctata* and *L.tripunctata*), five sphaeromatids (*Paracerceissculpta*, *Paradelladianae*, *Pseudosphaeroma* sp., *Sphaeromaquoianum*, and *S.walkeri*), one idoteid (*Synidoteaharfordi*), five asellotes (*Caecijaerahorvathi*, *Iaiscalifornica*, *Ianiropsisderjugini*, *Ianiropsistridens*, and *Munnastephenseni*), and one oniscid (*Niambiacapensis*). Also occasionally occurring in SCB waters during warm water years is the cosmopolitan pelagic species *Idoteametallica*. See the annotated species list provided in this review for additional details and references regarding each of the above species.

Some species may have been introduced multiple times, and others have become established residents in their new localities with expanding ranges. For example, based on mitochondrial data, the sphaeromatid *Pseudosphaeroma* sp. appears to have been introduced multiple times from the southern hemisphere (RW, pers. obs.). In Morro Bay located just north of the SCB, *Pseudosphaeroma* sp. is now known to be associated with dead barnacle tests found attached to dock pilings. Additionally, the species is known to co-occur with the sacoglossan sea slug *Alderia* sp. in San Francisco Bay on high intertidal mudflats, tolerating freshwater immersion following rain events. Most recently in 2018, a single specimen identified as *Pseudosphaeroma* sp. was collected in the Salinas de San Pedro (Los Angeles County), thus extending the range of this introduced species to the SCB (RW, pers. obs.; specimen in Natural History Museum of Los Angeles County collections, Catalog No. LACM:DISCO:9917).

Another sphaeromatid, *Paracerceissculpta*, best known for its multiple male morphs (designated alpha, beta, and gamma) in the northern Gulf of California (Shuster 1987, 1992), is expanding its range not only in SCB bays and harbors, but also on rocky outer coast habitats. In its newly adopted habitats, *P.sculpta* readily occurs on non-native bryozoans, algae, and assorted fouling organisms. Although originally described from pieces of a sponge dredged from shallow water off San Clemente Island in the SCB, *P.sculpta* is now widely distributed in coastal waters around the globe (Marchini et al. 2018). On the other hand, it is possible that *P.sculpta* as currently recognized represents more than one species. For example, the smaller beta and gamma males are known to co-occur with the larger alpha males only in northern Gulf of California populations, while populations outside of the Gulf include only alpha males. Unfortunately, there are no genetic barcode sequences available for northern Gulf of California specimens (RW and S Shuster, pers. comm.). In contrast, genetic sequences for *P.sculpta* occurring in the central and southern Gulf of California are similar to those from specimens collected in San Diego bays and harbors and northwards where beta and gamma males have never been noted. In other words, it is not out of the question that specimens from the northern Gulf of California are an unnamed *Paracerceis* species, and that the species name *P.sculpta* has been attributed incorrectly to isopods from that region. In fact, taxa attributed to broad geographic ranges are likely to be found to be different species upon closer examination unless it is a species with a propensity to be readily relocated by anthropogenic means. Another sphaeromatid example of such a pattern is the “*Exosphaeromaamplicauda*” clade referred to previously in this review (see Endnote 18), which in the past was reported as a single species ranging from the Aleutian Islands to northern Baja California. However, Wall et al. (2015) demonstrated that between Alaska and southern California there are at least five species comprising this group, and perhaps more, as these authors did not consider specimens from the Channel Islands where additional species are likely to occur. Because of such uncertainties, we acknowledge that unknown numbers of undescribed taxa are likely to become evident in the future as morphological taxonomic revisions are undertaken and new genetic data are added.

We also recognize the shortcomings of reproducing dorsal view line drawings for most species yet are grateful to previous authors for their efforts. Although dorsal views have long been the accepted standard depiction for isopods, such views are often not especially useful for identifying some species. For example, identification of some asellotes and arcturid valviferans would greatly benefit from lateral views. Ventral views depicting the pleotelson features of valviferans and sphaeromatids would also improve and simplify many identifications. However, such views do not exist for most taxa. High quality color photographs of live specimens would also be a vast improvement in facilitating accurate species identifications. Fortunately, with the increasing popularity of community platforms such as iNaturalist, in-situ color photographs of live animals are helping to expand our knowledge of species distributions, as well as contributing new and valuable habitat information. As videos of living specimens become more common, insights into the behavior and habitat use of species should also improve.

Several other active projects are also moving forward that should further our understanding of the biodiversity and taxonomy of many groups. For example, the National Science Foundation’s digitization efforts of natural history museum collections, such as the current 4-year effort underway at the Natural History Museum of Los Angeles County in southern California (DigIn: https://www.digin-tcn.org), involving 19 US collections, are making biological specimens that have been silently sitting on basement shelves for the past hundred years available on the web. Some collections such as the National Museum of Natural History (Smithsonian Institution) already have most of their collections digitized. Similarly, many collections in Europe, Australia, and New Zealand have all or most of their collections publicly available or are similarly working on capturing specimen metadata and publishing it. Consequently, these data are now becoming publicly available through existing portals, including the Integrated Digitized Biocollections (iDigBio.org) and Global Biodiversity Information Facility (GBIF), using standardized data formats. Genetic sequence data are also accumulating in the NIH genetic sequence database (GenBank) and the Barcode of Life Data Systems (BOLD). Electronic specimen data portals, such as InvertEBase https://invertebase.org/portal/ (based on Symbiota, open-source software for managing and mobilizing biodiversity data), are supporting a growing network of natural history collections, but will need continuing refinements in order to support regional needs and improve user accessibility and applicability. The Biodiversity Heritage Library (BHL) has made enormous amounts of previously inaccessible primary taxonomic literature readily available. Together these resources are game changers for accessibility to worldwide marine biodiversity data and our understanding of the changing marine environment around us.

The next step, just now beginning, is to knit the systems above (and others like them) into an integrated architecture of biodiversity knowledge. The goal is to realize the ideals expressed in the emerging “Extended Specimen” concept (see Hedrick et al. 2020; Lendemer et al. 2020; Miller et al. 2020; NASEM 2020; Heberling et al. 2021; Hardisty et al. 2022). In this view, specimens (e.g., type specimens) stand at the center of an interlinked cloud of digitally accessible information, enabling taxonomic understanding of biodiversity by combining local and non-local specimen-based data (Rocha et al. 2014; Meyer et al. 2021). Therefore, we hope that future versions of this guide to the SCB marine isopod fauna will function as a direct entry point to all available specimen-based data and taxonomic research relevant to the region’s taxonomy and biogeography.

## ﻿Appendix 1

**Table A1.** Systematic treatment and geographic distribution summary for Southern California Bight marine isopods (Crustacea: Isopoda). See annotated species list for details of geographic ranges. Distribution codes: BCN = Baja California Norte; BCS = Baja California Sur; CA = California; MX = Mexico; SCB = Southern California Bight; WW = worldwide (i.e., species also reported from regions beyond Eastern Pacific).

Taxon	Eastern Pacific Distribution
**Suborder Cymothoida**	
Superfamily Anthuroidea	
Family Antheluridae	
*Ananthuraluna* (Schultz, 1966)	SCB
Family Anthuridae	
*Amakusanthuracaliforniensis* (Schultz, 1964)	SCB – Isla Guadalupe, BCN
*Cyathuramunda* Menzies, 1951	Northern CA – Gulf of CA
*Haliophasmageminatum* Menzies & Barnard, 1959	British Columbia – San Quintin Bay, BCN
*Mesanthuraoccidentalis* Menzies & Barnard, 1959	SCB – Costa Rica
Family Expanathuridae	
*Eisothistos* sp. A	SCB
Family Hyssuridae	
*Kupellonura* sp. A	~ 35 km north of SCB
Family Paranthuridae	
*Califanthurasquamosissima* (Menzies, 1951)	Northern CA – Oaxaca, MX
*Colanthurabruscai* Poore, 1984	SCB – Costa Rica
*Paranthuraelegans* Menzies, 1951	Northern CA – Costa Rica
*Paranthurajaponica* Richardson, 1909	Northern CA – SCB (+ WW)
Superfamily Cymothooidea	
Family Aegidae	
*Aegalecontii* (Dana, 1854)	Central CA – SCB
*Aegiochusplebeia* (Hansen, 1897)	Aleutian Islands – Peru (+ West Pacific)
*Rocinelaangustata* Richardson, 1904	Bering Sea – Gulf of CA
*Rocinelabelliceps* (Stimpson, 1864)	Alaska – Gulf of CA
*Rocinelalaticauda* Hansen, 1897	Central CA – Acapulco, MX
*Rocinelamurilloi* Brusca & Iverson, 1985	Central CA – Chile
*Rocinelasignata* Schioedte & Meinert, 1879	Northern CA – Ecuador + Galapagos (+ WW)
Family Cirolanidae	
*Cirolanadiminuta* Menzies, 1962	SCB – Gulf of CA + Galapagos
*Cirolanaharfordi* (Lockington, 1877)	British Columbia – Magdalena Bay, BCS
*Eurydicecaudata* Richardson, 1899	SCB – Ecuador + Galapagos
*Excirolanachiltoni* (Richardson, 1905)	British Columbia – SCB (+ WW)
*Excirolanalinguifrons* (Richardson, 1899)	Oregon – SCB
*Metacirolanajoanneae* (Schultz, 1966)	British Columbia – SCB
*Natatolanacaliforniensis* (Schultz, 1966)	SCB – Peru-Chile Trench
Family Corallanidae	
*Excorallanatricornisoccidentalis* Richardson, 1905	SCB – Panama
*Excorallanatruncata* (Richardson, 1899)	SCB – Galapagos
Family Cymothoidae	
*Ceratothoagaudichaudii* (H. Milne Edwards, 1840)	SCB – Cape Horn (+ SW Atlantic)
*Ceratothoagilberti* (Richardson, 1904)	SCB – Mazatlán, MX
*Elthusacalifornica* (Schioedte & Meinert, 1884)	Alaska – Peru
*Elthusamenziesi* (Brusca, 1981)	Coronado Islands, BCN – Gulf of CA
*Elthusavulgaris* (Stimpson, 1857)	Oregon – Columbia
*Mothocyarosea* Bruce, 1986	SCB – Nicaragua
*Nerocilaacuminata* Schioedte & Meinert, 1881	SCB – Peru
*Renocilathresherorum* Williams & Bunkley-Williams, 1980	SCB – Gulf of CA (+ Egypt)
*Smenispaconvexa* (Richardson, 1905)	SCB
Family Gnathiidae	
*Caecognathiacrenulatifrons* (Monod, 1926)	British Columbia – BCN
*Caecognathiasanctaecrucis* (Schultz, 1972)	British Columbia – BCN
*Caecognathia* sp. A	SCB
*Caecognathia* sp. SD1	SCB
*Gnathiaclementensis* Schultz, 1966	SCB
*Gnathiacoronadoensis* Schultz, 1966	SCB
*Gnathiaproductatridens* Menzies & Barnard, 1959	SCB
*Gnathiasteveni* Menzies, 1962	British Columbia – SCB
*Gnathiatridens* Menzies & Barnard, 1959	British Columbia – SCB
*Gnathiatrilobata* Schultz, 1966	British Columbia – SCB
*Gnathia* sp. MBC1	SCB
Family Tridentellidae	
*Tridentellaglutacantha* Delaney & Brusca, 1985	Central CA – SCB
*Tridentellaquinicornis* Delaney & Brusca, 1985	SCB
Infraorder Epicaridea	
Superfamily Bopyroidea	
Family Bopyridae	
*Anathelgeshyphalus* (Markham, 1974)	Central CA – SCB
*Aporobopyrusmuguensis* Shiino, 1964	Northern CA – Central Baja CA
*Aporobopyrusoviformis* Shiino, 1934	SCB (+ Japan)
*Argeiapugettensis* Dana, 1853	Bering Sea – SCB (+ Japan, Korea)
*Asymmetrioneambodistorta* Markham, 1985	SCB
*Bathygygegrandis* Hansen, 1897	SCB – MX (+ WW)
*Bopyrellacalmani* (Richardson, 1905)	Central CA – SCB
*Capitetragonia* sp. A	SCB
*Eremitionegiardi* (Calman, 1898)	Bering Sea – SCB
*Leidyainfelix* Markham, 2002	SCB – San Quintin Bay, BCN
*Munidionpleuroncodis* Markham, 1975	Central CA – Central MX
*Orthionegriffenis* Markham, 2004	British Columbia – SCB (+ Asia)
*Phyllodurusabdominalis* Stimpson, 1857	British Columbia – BCN
*Progebiophilusbruscai* Salazar-Vallejo & Leija-Tristán, 1990	SCB – Nicaragua
*Pseudionegalacanthae* Hansen, 1897	British Columbia – Gulf of CA
*Schizobopyrinastriata* (Nierstrasz & Brender à Brandis, 1929)	SCB – Gulf of CA
Family Ionidae	
*Ionecornuta* Spence Bate, 1863	British Columbia – Gulf of CA (+ Asia)
Superfamily Cryptoniscoidea	
Family Dajidae	
*Holophryxusalaskensis* Richardson, 1905	Alaska – SCB
*Zonophryxus* sp.	SCB
Family Hemioniscidae	
*Hemioniscusbalani* Buchholz, 1866	British Columbia – MX (+ WW)
**Suborder Limnoriidea**	
Superfamily Limnorioidea	
Family Limnoriidae	
*Limnoriaalgarum* Menzies, 1957	British Columbia – SCB
*Limnoriaquadripunctata* Holthuis, 1949	Northern CA – SCB (+ WW)
*Limnoriatripunctata* Menzies, 1951	Washington – Mazatlán, MX (+ WW)
**Suborder Sphaeromatidea**	
Superfamily Seroloidea	
Family Serolidae	
*Heteroseroliscarinata* (Lockington, 1877)	SCB – San Quintin Bay, BCN, + Gulf of CA
Superfamily Sphaeromatoidea	
Family Ancinidae	
*Ancinusgranulatus* Holmes & Gay, 1909	SCB – Gulf of CA
*Ancinusseticomvus* Trask, 1971	SCB – Mazatlán, MX
*Bathycopeadaltonae* (Menzies & Barnard, 1959)	Washington – SCB
Family Sphaeromatidae	
*Discerceisgranulosa* (Richardson, 1899)	SCB – Isla Cedros, BCN
*Dynamenelladilatata* (Richardson, 1899) †	Washington – SCB
*Dynamenellaglabra* (Richardson, 1899) †	Oregon – SCB
*Dynamenellasheareri* (Hatch, 1947)	Washington – SCB
*Dynoideselegans* (Boone, 1923)	SCB – Isla Cedros, BCN
*Exosphaeromaamplicauda* (Stimpson, 1857) ‡	Northern CA
*Exosphaeromaaphrodita* Boone, 1923	SCB
*Exosphaeromainornata* Dow, 1958	Washington – SCB
*Exosphaeromapentcheffi* Wall, Bruce & Wetzer, 2015	SCB
*Exosphaeromarhomburum* (Richardson, 1899)	Washington – SCB
*Gnorimosphaeromanoblei* Menzies, 1954	Northern CA – SCB
*Gnorimosphaeromaoregonense* (Dana, 1853) ‡	British Columbia – Central CA
*Paracerceiscordata* (Richardson, 1899)	Aleutian Islands – SCB
*Paracerceisgilliana* (Richardson, 1899)	Northern CA – SCB
*Paracerceissculpta* (Holmes, 1904)	Central CA – Gulf of CA (+ WW)
*Paracerceis* sp. A	SCB + Gulf of CA
*Paradelladianae* (Menzies, 1962)	SCB – Gulf of CA (+ WW)
*Pseudosphaeroma* sp.	Northern CA – SCB (+ WW)
*Sphaeromaquoianum* H. Milne Edwards, 1840	Northern CA – SCB (+ WW)
*Sphaeromawalkeri* Stebbing, 1905	SCB (+ WW)
Family Tecticipitidae	
*Tecticepsconvexus* Richardson, 1899	Washington – SCB
**Suborder Valvifera**	
Family Arcturidae	
*Idarcturusallelomorphus* Menzies & Barnard, 1959	Central CA – SCB
*Idarcturushedgpethi* Menzies, 1951	British Columbia – SCB
*Idarcturus* sp. A	SCB
*Neastacillacalifornica* (Boone, 1918)	SCB – Gulf of CA
Family Holognathidae	
*Cleantioidesoccidentalis* (Richardson, 1899)	SCB – Gulf of CA, Ecuador + Galapagos
Family Idoteidae	
*Colidoteafindleyi* Brusca & Wallerstein, 1977	SCB – Guadalupe Isla, BCN + Gulf of CA
*Colidotearostrata* (Benedict, 1898)	Oregon – SCB
*Colidoteawallersteini* Brusca, 1983	SCB – Guadalupe Isla, BCN
*Edotiasublittoralis* Menzies & Barnard, 1959	British Columbia – Gulf of CA + Costa Rica
*Edotia* sp. B	SCB
*Erichsonellacrenulata* Menzies, 1950	SCB – San Quintin Bay, BCN
*Eusymmeruspseudoculata* (Boone, 1923)	SCB
*Idoteafewkesi* Richardson, 1905	Alaska – BCS
*Idoteametallica* Bosc, 1801	WW (pelagic), including SCB
*Idotearufescens* Fee, 1927	Alaska – Baja CA
*Idoteaurotoma* Stimpson, 1864	Alaska – Gulf of CA
*Pentidoteaaculeata* Stafford, 1913	British Columbia – Gulf of CA
*Pentidoteakirchanskii* (Miller & Lee, 1970)	Oregon – SCB
*Pentidoteamontereyensis* Maloney, 1933	Alaska – Baja CA
*Pentidotearesecata* (Stimpson, 1857)	Alaska – Gulf of CA
*Pentidoteaschmitti* (Menzies, 1950)	Bering Sea – SCB
*Pentidoteastenops* (Benedict, 1898)	Alaska – Gulf of CA
*Pentidoteawosnesenskii* (Brandt, 1851)	Aleutian Islands – Gulf of CA
*Stenosomawetzerae* (Ormsby, 1991)	SCB – Gulf of CA
*Synidoteacalcarea* Schultz, 1966	SCB
*Synidoteaharfordi* Benedict, 1897	Oregon – Costa Rica (+ Japan)
*Synidoteamagnifica* Menzies & Barnard, 1959	Central CA – SCB
*Synidoteamedia* Iverson, 1972	British Columbia – SCB
Family Thermoarcturidae	
*Califarcturustannerensis* (Schultz, 1966)	SCB
**Suborder Asellota**	
Superfamily Janiroidea	
Family Dendrotionidae	
*Acanthomunnatannerensis* Schultz, 1966	SCB
Family Desmosomatidae	
*Desmosoma* sp. A	~ 89 km north of SCB
*Momedossasymmetrica* (Schultz, 1966)	Northern CA – SCB
*Prochelator* sp. A	Northern CA to ~ 35 km north of SCB
Family Haplomunnidae	
*Haplomunnacaeca* (Richardson, 1905)	SCB
Family Janiridae	
*Caecianiropsis* sp. LA1	SCB
*Caecianiropsis* sp. LA2	SCB
*Caecijaerahorvathi* Menzies, 1951	SCB (+ Hawaii)
*Iaiscalifornica* (Richardson, 1904)	Northern CA – SCB (+ WW)
*Ianiropsisanaloga* Menzies, 1952	Washington – SCB
*Ianiropsisderjugini* Gurjanova, 1933	Bering Sea – SCB (+ Korea)
*Ianiropsiskincaidi* Richardson, 1904	Bering Sea – SCB
*Ianiropsisminuta* Menzies, 1952	Northern CA – SCB
*Ianiropsismontereyensis* Menzies, 1952	Northern CA – SCB
*Ianiropsistridens* Menzies, 1952	British Columbia – Chile (+ Korea)
*Janiralatadavisi* Menzies, 1951	Central CA – SCB
*Janiralataoccidentalis* (Walker, 1898)	British Columbia – SCB
*Janiralatarajata* Menzies, 1951	Central CA – SCB
*Janiralatasolasteri* (Hatch, 1947)	Alaska – SCB
*Janiralata* sp. A	SCB
*Janiralata* sp. B	SCB
*Janiralata* sp. C	~ 35 km north of SCB
*Janiralata* sp. D	~ 35 km north of SCB
Family Joeropsididae	
*Joeropsisconcava* Schultz, 1966	Central CA – SCB
*Joeropsisdubiadubia* Menzies, 1951	British Columbia – Gulf of CA
*Joeropsisdubiapaucispinis* Menzies, 1951	Northern CA – SCB
*Joeropsislobata* Richardson, 1899	Oregon to SCB
*Joeropsis* sp. A	~ 30 km north of SCB
Family Lepidocharontidae	
*Microcharon* sp. A	SCB
Family Munnidae	
*Munnachromatocephala* Menzies, 1952	British Columbia – SCB
*Munnafernaldi* George & Strömberg, 1968	Washington – SCB
*Munnahalei* Menzies, 1952	Northern CA – SCB
*Munnamagnifica* Schultz, 1964	SCB
*Munnaspinifrons* Menzies & Barnard, 1959	SCB
*Munnastephenseni* Gurjanova, 1933	Bering Sea – SCB (+ Japan)
*Munna* sp. A	SCB
*Uromunnaubiquita* (Menzies, 1952)	British Columbia – Gulf of CA
Family Munnopsidae	
*Belonectes* sp. A	SCB
*Eurycopecaliforniensis* Schultz, 1966	SCB
*Ilyarachnaacarina* Menzies & Barnard, 1959	Northern CA – SCB
*Ilyarachnaprofunda* Schultz, 1966	Northern CA – SCB
*Munnopsurus* sp. A	SCB
*Munnopsurus* sp. B	SCB
Family Nannoniscidae	
*Nannonisconuslatipleonus* Schultz, 1966	SCB
Family Paramunnidae	
*Austrosignumlatum* Just & Wilson, 2021	SCB
*Boreosignum* sp. A	SCB
*Munnogoniumerratum* (Schultz, 1964)	Central CA – SCB
*Munnogoniumtillerae* (Menzies & Barnard, 1959)	SCB
*Paramunnaquadratifrons* Iverson & Wilson, 1981	SCB
*Paramunna* sp. A	SCB
*Paramunna* sp. SD1	SCB
*Pleurogoniumcaliforniense* Menzies, 1951	British Columbia – SCB
*Pleurogonium* sp. A	SCB
Family Pleurocopidae	
*Pleurocope* sp. A	Central CA – SCB
Superfamily Stenetrioidea	
Family Stenetriidae	
*Stenetrium* sp. A	~ 35 km north of SCB
**Suborder Oniscidea**	
Superfamily Oniscoidea	
Family Alloniscidae	
*Alloniscusmirabilis* (Stuxberg, 1875)	Northern CA – Magdalena Bay, BCS
*Alloniscusperconvexus* Dana, 1854	British Columbia – Magdalena Bay, BCS
Family Detonidae	
*Armadilloniscuscoronacapitalis* Menzies, 1950	Northern CA – SCB
*Armadilloniscusholmesi* Arcangeli, 1933	British Columbia – Magdalena Bay, BCS
*Armadilloniscuslindahli* (Richardson, 1905)	Northern CA – SCB
Family Halophilosciidae	
*Littorophilosciarichardsonae* (Holmes & Gay, 1909)	British Columbia – Isla Cedros, BCN
Family Ligiidae	
Ligiacf.occidentalis (Dana, 1853)	SCB – BCS
Family Platyarthridae	
*Niambiacapensis* (Dollfus, 1895)	Washington – Chile (+ WW)
Family Tylidae	
*Tylospunctatus* Holmes & Gay, 1909	SCB – Gulf of CA

† Taxon inquirendum (genus uncertain for *Dynamenelladilatata* and *D.glabra*). ‡ No longer considered a SCB species (see Wall et al. 2015 for *E.amplicauda*, and Wetzer et al. 2021 for *G. oregonense*).
